# 2019 HRS/EHRA/APHRS/LAHRS expert consensus statement on catheter ablation of ventricular arrhythmias

**DOI:** 10.1007/s10840-019-00663-3

**Published:** 2020-01-27

**Authors:** Edmond M. Cronin, Frank M. Bogun, Philippe Maury, Petr Peichl, Minglong Chen, Narayanan Namboodiri, Luis Aguinaga, Luiz Roberto Leite, Sana M. Al-Khatib, Elad Anter, Antonio Berruezo, David J. Callans, Mina K. Chung, Phillip Cuculich, Andre d’Avila, Barbara J. Deal, Paolo Della Bella, Thomas Deneke, Timm-Michael Dickfeld, Claudio Hadid, Haris M. Haqqani, G. Neal Kay, Rakesh Latchamsetty, Francis Marchlinski, John M. Miller, Akihiko Nogami, Akash R. Patel, Rajeev Kumar Pathak, Luis C. Saenz Morales, Pasquale Santangeli, John L. Sapp, Andrea Sarkozy, Kyoko Soejima, William G. Stevenson, Usha B. Tedrow, Wendy S. Tzou, Niraj Varma, Katja Zeppenfeld

**Affiliations:** 1 Hartford Hospital, Hartford, CT USA; 2 University of Michigan, Ann Arbor, MI USA; 3 University Hospital Rangueil, Toulouse, France; 4 Institute for Clinical and Experimental Medicine, Prague, Czech Republic; 5 Jiangsu Province Hospital, The First Affiliated Hospital of Nanjing Medical University, Nanjing, China; 6 Sree Chitra Institute for Medical Sciences and Technology, Thiruvananthapuram, India; 7 Centro Privado de Cardiología, Tucuman, Argentina; 8 Instituto Brasília de Arritmia, Brasília, Brazil; 9 Duke University Medical Center, Durham, NC USA; 10 Beth Israel Deaconess Medical Center, Boston, MA USA; 11 Heart Institute, Teknon Medical Center, Barcelona, Spain; 12 University of Pennsylvania, Philadelphia, PA USA; 13 Cleveland Clinic, Cleveland, OH USA; 14 Washington University School of Medicine, St. Louis, MO USA; 15 Hospital Cardiologico SOS Cardio, Florianopolis, Brazil; 16 Northwestern University Feinberg School of Medicine, Chicago, IL USA; 17 Ospedale San Raffaele, Milan, Italy; 18 Herz- und Gefäß-Klinik, Bad Neustadt, Germany; 19 University of Maryland, Baltimore, MD USA; 20 Hospital General de Agudos Cosme Argerich, Buenos Aires, Argentina; 21 University of Queensland, The Prince Charles Hospital, Chermside, Australia; 22 University of Alabama at Birmingham, Birmingham, AL USA; 23 Indiana University School of Medicine, Krannert Institute of Cardiology, Indianapolis, IN USA; 24 University of Tsukuba, Ibaraki, Japan; 25 University of California San Francisco Benioff Children’s Hospital, San Francisco, CA USA; 26 Australian National University, Canberra Hospital, Canberra, Australia; 27 CardioInfantil Foundation, Cardiac Institute, Bogota, Columbia USA; 28 Queen Elizabeth II Health Sciences Centre, Halifax, Canada; 29 University Hospital Antwerp, University of Antwerp, Antwerp, Belgium; 30 Kyorin University School of Medicine, Tokyo, Japan; 31 Vanderbilt University Heart and Vascular Center, Nashville, TN USA; 32 Brigham and Women’s Hospital, Boston, MA USA; 33 University of Colorado Denver, Aurora, CO USA; 34 Leiden University Medical Center, Leiden, the Netherlands

**Keywords:** Catheter ablation, Clinical document, Electrical storm, Electroanatomical mapping, Electrocardiogram, Expert consensus statement, Imaging, Premature ventricular complex, Radiofrequency ablation, Ventricular arrhythmia, Ventricular tachycardia

## Abstract

Ventricular arrhythmias are an important cause of morbidity and mortality and come in a variety of forms, from single premature ventricular complexes to sustained ventricular tachycardia and fibrillation. Rapid developments have taken place over the past decade in our understanding of these arrhythmias and in our ability to diagnose and treat them. The field of catheter ablation has progressed with the development of new methods and tools, and with the publication of large clinical trials. Therefore, global cardiac electrophysiology professional societies undertook to outline recommendations and best practices for these procedures in a document that will update and replace the 2009 *EHRA/HRS Expert Consensus on Catheter Ablation of Ventricular Arrhythmias*. An expert writing group, after reviewing and discussing the literature, including a systematic review and meta-analysis published in conjunction with this document, and drawing on their own experience, drafted and voted on recommendations and summarized current knowledge and practice in the field. Each recommendation is presented in knowledge byte format and is accompanied by supportive text and references. Further sections provide a practical synopsis of the various techniques and of the specific ventricular arrhythmia sites and substrates encountered in the electrophysiology lab. The purpose of this document is to help electrophysiologists around the world to appropriately select patients for catheter ablation, to perform procedures in a safe and efficacious manner, and to provide follow-up and adjunctive care in order to obtain the best possible outcomes for patients with ventricular arrhythmias.

**Electronic supplementary material:**

The online version of this article (10.1007/s10840-019-00663-3) contains supplementary material, which is available to authorized users.

## TABLE OF CONTENTS

1. Introduction ....................................... *In this issue*
  1.1. Document Scope and Rationale .......... *In this issue*
  1.2. Methods .............................................. *In this issue*
2. Background ...................................... *In this issue*
  2.1. History of Ventricular Arrhythmia Ablation .............................................. *In this issue*
  2.2. Mechanisms of Ventricular Arrhythmia ......................................... *In this issue*
    2.2.1.1. Mechanisms and Basis for Catheter Ablation of Ventricular Tachycardia ................................ *In this issue*
    2.2.1.2. Triggered Activity and Automaticity .............................. *In this issue*
    2.2.1.3. Scar-Related Reentry ................. *In this issue*
    2.2.1.4. Reentry in the Purkinje System and Ventricular Fibrillation ....... *In this issue*
  2.3. Definitions .......................................... *In this issue*
  2.4. Standard Anatomical Terminology ..... *In this issue*
3. Clinical Evaluation ........................... *In this issue*
  3.1. Clinical Presentation ........................... *In this issue*
  3.2. Diagnostic Evaluation ......................... *In this issue*
    3.2.1.1. Resting 12-Lead Electrocar-diogram ...................................... *In this issue*
    3.2.1.2. Assessment of Structural Heart Disease and Myocardial Ischemia ..................................... *In this issue*
    3.2.1.3. Risk Stratification in the Setting of Frequent Premature Ventri-cular Complexes ......................... *In this issue*
    3.2.1.4. Longitudinal Follow-up in the Setting of Frequent Premature Ventricular Complexes .............. *In this issue*
4. Indications for Catheter Ablation ....... *In this issue*
  4.1. Idiopathic Outflow Tract Ventricular Arrhythmia .......................................... *In this issue*
  4.2. Idiopathic Nonoutflow Tract Ventricular Arrhythmia ........................ *In this issue*
  4.3. Premature Ventricular Complexes With or Without Left Ventricular Dysfunction ........................................ *In this issue*
  4.4. Ventricular Arrhythmia in Ischemic Heart Disease ...................................... *In this issue*
  4.5. Nonischemic Cardiomyopathy ........... *In this issue*
  4.6. Ventricular Arrhythmia Involving the His-Purkinje System, Bundle Branch Reentrant Ventricular Tachycardia, and Fascicular Ventricular Tachycardia .......................................... *In this issue*
  4.7. Congenital Heart Disease .................... *In this issue*
  4.8. Inherited Arrhythmia Syndromes ....... *In this issue*
  4.9. Ventricular Arrhythmia in Hypertrophic Cardiomyopathy ............ *In this issue*
5. Procedural Planning ........................... *In this issue*
  5.1. Patient Selection and Preprocedural Risk Assessment ........... *In this issue*
    5.1.1. The PAAINESD Risk Score ......... *In this issue*
    5.1.2. The Seattle Heart Failure Model ............................................ *In this issue*
    5.1.3. Multidisciplinary Involvement ..... *In this issue*
  5.2. 12-Lead Electrocardiogram and Body Surface Mapping Before Ventricular Tachycardia Ablation ....... *In this issue*
    5.2.1. Standard 12-Lead Electrocar-diogram ......................................... *In this issue*
    5.2.2. Ventricular Tachycardia and Premature Ventricular Complex in the Absence of Structural Heart Disease ................................ *In this issue*
    5.2.3. Postinfarction Ventricular Tachycardia ................................... *In this issue*
    5.2.4. Epicardial Sources ........................ *In this issue*
    5.2.5. Ventricular Tachycardia in Nonischemic Cardiomyopathy ...... *In this issue*
    5.2.6. Bundle Branch Reentrant Ventricular Tachycardia ................ *In this issue*
    5.2.7. Body Surface Mapping ................. *In this issue*
    5.2.8. Summary ...................................... *In this issue*
  5.3. Facilities for the Procedure .................. *In this issue*
    5.3.1. Facilities ........................................ *In this issue*
    5.3.2. Laboratory Equipment .................. *In this issue*
    5.3.3. Personnel ....................................... *In this issue*
    5.3.4. Patient Safety ................................. *In this issue*
  5.4. Preprocedural Imaging ........................ *In this issue*
  5.5. Patient Preparation ............................... *In this issue*
6. Intraprocedural Patient Care ............. *In this issue*
  6.1. Anesthesia ............................................ *In this issue*
  6.2. Vascular Access ................................... *In this issue*
  6.3. Epicardial Access ................................ *In this issue*
    6.3.1. Background ................................... *In this issue*
    6.3.2. Criteria Suggesting Epicardial Substrate ........................................ *In this issue*
    6.3.3. Epicardial Access Technique ........ *In this issue*
    6.3.4. Epicardial Access Compli-cations ........................................... *In this issue*
  6.4. Intraprocedural Hemodynamic Support ................................................ *In this issue*
  6.5. Intraprocedural Anticoagulation ......... *In this issue*
  6.6. Antibiotic Prophylaxis ........................ *In this issue*
  6.7. Fluid Balance ....................................... *In this issue*
7. Electrophysiological Testing ............. *In this issue*
8. Mapping and Imaging Techniques .... *In this issue*
  8.1. Mapping Catheters .............................. *In this issue*
    8.1.1. Multielectrode Mapping ................ *In this issue*
  8.2. Activation Mapping ............................ *In this issue*
  8.3. Entrainment Mapping .......................... *In this issue*
    8.3.1. Entrainment Mapping: Overview ...................................... *In this issue*
    8.3.2. How to Perform Entrainment Mapping ........................................ *In this issue*
  8.4. Pace Mapping ...................................... *In this issue*
  8.5. Sinus Rhythm Substrate Mapping ..... *In this issue*
    8.5.1. Substrate Mapping in Sinus Rhythm ......................................... *In this issue*
    8.5.2. Summary ....................................... *In this issue*
  8.6. Intraprocedural Imaging: Intracardiac Echocardiography, Fluoroscopy, Cardiac Magnetic Resonance Imaging ............................. *In this issue*
    8.6.1. Intraprocedural Imaging During Catheter Ablation of Ventricular Arrhythmias .......... *In this issue*
    8.6.2. Summary ....................................... *In this issue*
  8.7. Electroanatomical Mapping Systems and Robotic Navigation ...................... *In this issue*
9. Mapping and Ablation ..................... *In this issue*
  9.1. Ablation Power Sources and Techniques .......................................... *In this issue*
    9.1.1. Introduction .................................. *In this issue*
    9.1.2. Unipolar Radiofrequency Catheter Ablation ........................................ *In this issue*
    9.1.3. Contact Force Sensing ................. *In this issue*
    9.1.4. Hypotonic External Irrigation ....... *In this issue*
    9.1.5. Simultaneous Unipolar or Simultaneous Bipolar Radiofreq-uency Delivery .............................. *In this issue*
    9.1.6. Needle Ablation ............................ *In this issue*
    9.1.7. Cryoablation ................................. *In this issue*
    9.1.8. Transvascular Ethanol Ablation .... *In this issue*
    9.1.9. Stereotactic Radiotherapy ............. *In this issue*
  9.2. Idiopathic Outflow Tract Ventricular Arrhythmia .......................................... *In this issue*
    9.2.1. Introduction .................................. *In this issue*
    9.2.2. General Approach ......................... *In this issue*
    9.2.3. Right Ventricular Outflow Tract and Pulmonary Artery .................. *In this issue*
    9.2.4. Aortic Sinuses of Valsalva ............. *In this issue*
    9.2.5. Left Ventricular Outflow Tract and Left Ventricular Summit ......... *In this issue*
    9.2.6. Para-Hisian Ventricular Arrhythmias ................................. *In this issue*
    9.2.7. Deep Intraseptal Sites ................... *In this issue*
  9.3. Idiopathic Nonoutflow Tract Ventricular Arrhythmia ............................ *In this issue*
    9.3.1. Ventricular Arrhythmias from the Tricuspid and Mitral Annuli ......... *In this issue*
    9.3.2. Mapping and Ablation of Ventricular Arrhythmia from the Papillary Muscles ................... *In this issue*
  9.4. Bundle Branch Reentrant Ventri-cular Tachycardia and Fascicular Ventricular Tachycardia ...................... *In this issue*
    9.4.1. Introduction ...
    9.4.2. Bundle Branch Reentrant Ventricular Tachycardia ................ *In this issue*
    9.4.3. Idiopathic Fascicular Reentrant Ventricular Tachycardia ............... *In this issue*
    9.4.4. Focal Nonreentrant Fascicular Ventricular Tachycardia and Premature Ventricular Complex ....... *In this issue*
  9.5. Postinfarction Ventricular Tachycardia ......................................... *In this issue*
    9.5.1. General Considerations ................ *In this issue*
    9.5.2. Clinical, Unknown Clinical, and Nonclinical Ventricular Tachycardia ................................... *In this issue*
    9.5.3. Mapping and Ablation Strategy ..... *In this issue*
    9.5.4. Substrate-Based Ablation Strategies Without Upfront Ventricular Tachycardia Induction ................... *In this issue*
    9.5.5. Epicardial Mapping and Ablation ........................................ *In this issue*
  9.6. Dilated Cardiomyopathy ...................... *In this issue*
  9.7. Ventricular Tachycardia Ablation in Hypertrophic Cardiomyopathy ............ *In this issue*
  9.8. Brugada Syndrome ............................. *In this issue*
    9.8.1. Introduction ................................... *In this issue*
    9.8.2. Approach to Triggering Premature Ventricular Complexes ................. *In this issue*
    9.8.3. Approach to Sustained Monomorphic Ventricular Tachycardia .................................. *In this issue*
    9.8.4. Approach to Polymorphic Ventricular Tachycardia/Ventri-cular Fibrillation ............................ *In this issue*
    9.8.5. Outcomes ...................................... *In this issue*
    9.8.6. Risks ............................................. *In this issue*
  9.9. Polymorphic Ventricular Tachyca-rdia/Ventricular Fibrillation Triggers ............................................... *In this issue*
  9.10. Arrhythmogenic Right Ventricular Cardiomyopathy ............. *In this issue*
    9.10.1. Introduction to the Specific Disease Substrate Characteri-stics ............................................. *In this issue*
    9.10.2. General Management ................. *In this issue*
    9.10.3. General Approach for Ablation ...................................... *In this issue*
    9.10.4. Risks ........................................... *In this issue*
  9.11. Mapping and Ablation in Congenital Heart Disease ................... *In this issue*
    9.11.1. Introduction ................................ *In this issue*
    9.11.2. Mapping and Ablation ................ *In this issue*
    9.11.3. Outcome After Ablation ............ *In this issue*
  9.12. Sarcoidosis ........................................ *In this issue*
  9.13. Chagas Disease .................................. *In this issue*
    9.13.1. Chagas Disease .......................... *In this issue*
    9.13.2. Ventricular Tachycardia in Chagas Cardiomyopathy ......... *In this issue*
    9.13.3. Epicardial Ablation of Sus-tained Ventricular Tachyca-rdia in Chagas Heart Disease ..... *In this issue*
  9.14. Miscellaneous Diseases and Clin-ical Scenarios With Ventricular Tach-ycardia ............................................... *In this issue*
    9.14.1. Lamin Cardiomyopathy .............. *In this issue*
    9.14.2. Left Ventricular Noncompaction e119
    9.14.3. Congenital Left Ventricular Aneurysms .................................. *In this issue*
    9.14.4. Left Ventricular Assist Devices ... *In this issue*
  9.15. Surgical Therapy ................................ *In this issue*
  9.16. Sympathetic Modulation .................. *In this issue*
  9.17. Endpoints of Catheter Ablation of Ventricular Tachycardia ................ *In this issue*
    9.17.1. Historical Perspective ................ *In this issue*
    9.17.2. Programmed Electrical Stimu-lation ........................................... *In this issue*
    9.17.3. Current Ablation Strategies and Assessment of Results ................ *In this issue*
    9.17.4. Summary .................................... *In this issue*
10. Postprocedural Care ......................... *In this issue*
  10.1. Postprocedural Care: Access, Anticoagulation, Disposition ............ *In this issue*
    10.1.1. Postprocedural Care: Access ...... *In this issue*
    10.1.2. Atrial Fibrillation After Epicar-dial Ventricular Arrhythmia Ablation ...................................... *In this issue*
    10.1.3. Postprocedural Care:Anticoa-gulation ....................................... *In this issue*
    10.1.4. Postprocedural Care: Dispo-sition ............................................ *In this issue*
  10.2. Incidence and Management of Complications .................................. *In this issue*
    10.2.1. Introduction ................................. *In this issue*
    10.2.2. Mortality ..................................... *In this issue*
    10.2.3. Acute Periprocedural Hemodynamic Decompe-nsation and Cardiogenic Shock ........................................... *In this issue*
    10.2.4. Neurological Complications ...... *In this issue*
    10.2.5. Pericardial Complica-tions: Cardiac Tamponade, Hemopericardium, and Perica-rditis ........................................... *In this issue*
    10.2.6. Vascular Injury ........................... *In this issue*
    10.2.7. Myocardial Ischemia, Coronary Artery Damage ........................... *In this issue*
    10.2.8. Valve Injury ................................ *In this issue*
    10.2.9. Atrioventricular Block ................ *In this issue*
  10.3. Hemodynamic Deterioration and Proarrhythmia .................................... *In this issue*
  10.4. Follow-up of Patients Post Catheter Ablation of Ventricular Tach-ycardia .............................................. *In this issue*
  10.5. Assessing the Outcomes of Catheter Ablation ............................................ *In this issue*
    10.5.1. Introduction ................................ *In this issue*
    10.5.2. Recurrent Arrhythmias ................ *In this issue*
    10.5.3. Arrhythmia Burden .................... *In this issue*
    10.5.4. Ventricular Tachycardia Storm .......................................... *In this issue*
    10.5.5. Hospitalizations .......................... *In this issue*
    10.5.6. Patient-Reported Outcomes ........ *In this issue*
    10.5.7. Mortality ..................................... *In this issue*
11. Training and Institutional Requi-rements and Competencies ............ *In this issue*
  11.1. Training Requirements and Compe-tencies for Catheter Ablation of Ventricular Arrhythmias ................ *In this issue*
    11.1.1. Training Requirements ................ *In this issue*
    11.1.2. Medical Knowledge ................... *In this issue*
    11.1.3. Patient Care and Procedural Skills ............................................ *In this issue*
    11.1.4. Systems-Based Practice ............. *In this issue*
    11.1.5. Practice-Based Learning and Improvement .............................. *In this issue*
    11.1.6. Professionalism .......................... *In this issue*
    11.1.7. Interpersonal and Communi-cations Skills .............................. *In this issue*
    11.1.8. Ionizing Radiation ...................... *In this issue*
  11.2. Institutional Requirements for Catheter Ablation of Ventricular Tachycardia ........................................ *In this issue*
  11.3. Ventricular Tachycardia Network and Ventricular Tachycardia Unit .................................................... *In this issue*
12. Future Directions ............................ *In this issue*
  12.1. Clinical Trials of Catheter Ablation of Ventricular Tachycardia ................ *In this issue*
    12.1.1. Introduction ................................ *In this issue*
    12.1.2. Ongoing Randomized Contro-lled Trials .................................... *In this issue*
    12.1.3. Endpoints for Prospective Clin-ical Trials of Ventricular Tach-ycardia Ablation ......................... *In this issue*
    12.1.4. Future Clinical Studies ................ *In this issue*
  12.2. Future Directions in the Treatment of Patients With Ventricular Arrhythmias ...................................... *In this issue*
    12.2.1. Introduction
    12.2.2. Advances in Mapping ................ *In this issue*
    12.2.3. Advances in Ablation ................ *In this issue*
    12.2.4. Advances in Patient Evalu-ation ............................................. *In this issue*
13. Author Disclosure Table ................ *In this issue*
14. Reviewer Disclosure Table ............ *In this issue*

## Introduction

### Document scope and rationale

The field of electrophysiology has undergone rapid progress in the last decade, with advances both in our understanding of the genesis of ventricular arrhythmias (VAs) and in the technology used to treat them. In 2009, a joint task force of the European Heart Rhythm Association (EHRA) and the Heart Rhythm Society (HRS), in collaboration with the American College of Cardiology (ACC) and the American Heart Association (AHA), produced an expert consensus document that outlined the state of the field and defined the indications, techniques, and outcome measures of VA ablation [1]. In light of advances in the treatment of VAs in the interim, and the growth in the number of VA ablations performed in many countries and regions [2, 3], an updated document is needed. This effort represents a worldwide partnership between transnational cardiac electrophysiology societies, namely, HRS, EHRA, the Asia Pacific Heart Rhythm Society (APHRS), and the Latin American Heart Rhythm Society (LAHRS), and collaboration with ACC, AHA, the Japanese Heart Rhythm Society (JHRS), the Brazilian Society of Cardiac Arrhythmias (Sociedade Brasileira de Arritmias Cardíacas [SOBRAC]), and the Pediatric and Congenital Electrophysiology Society (PACES). The consensus statement was also endorsed by the Canadian Heart Rhythm Society (CHRS).

This clinical document is intended to supplement, not replace, the *2017 AHA/ACC/HRS Guideline for Management of Patients with Ventricular Arrhythmias and the Prevention of Sudden Cardiac Death* [4] and the *2015 ESC Guidelines for the Management of Patients with Ventricular Arrhythmias and the Prevention of Sudden Cardiac Death* [5]. The scope of the current document relates to ablation therapy for VAs, from premature ventricular complexes (PVCs) to monomorphic and polymorphic ventricular tachycardia (VT) and triggers of ventricular fibrillation (VF). Due to its narrower scope, the consensus statement delves into greater detail with regard to indications and technical aspects of VA ablation than the above-mentioned guidelines.

Where possible, the recommendations in this document are evidence based. It is intended to set reasonable standards that can be applicable worldwide, while recognizing the different resources, technological availability, disease prevalence, and health care delivery logistics in various parts of the world. In addition, parts of this document, particularly Section 9, present a practical guide on how to accomplish the procedures described in a manner that reflects the current standard of care, while recognizing that some procedures are better performed, and some disease states better managed, in settings in which there is specific expertise.

### Methods

The writing group was selected according to each society’s procedures, including content and methodology experts representing the following organizations: HRS, EHRA, APHRS, LAHRS, ACC, AHA, JHRS, PACES, and SOBRAC. Each partner society nominated a chair and co-chair, who did not have relevant relationships with industry and other entities (RWIs). In accordance with HRS policies, disclosure of any RWIs was required from the writing committee members (Appendix 1) and from all peer reviewers (Appendix 2). Of the 38 committee members, 17 (45%) had no relevant RWIs. Recommendations were drafted by the members who did not have relevant RWIs. Members of the writing group conducted comprehensive literature searches of electronic databases, including Medline (via PubMed), Embase, and the Cochrane Library. Evidence tables were constructed to summarize the retrieved studies, with nonrandomized observational designs representing the predominant form of evidence (Appendix 3). Case reports were not used to support recommendations. Supportive text was drafted in the “knowledge byte” format for each recommendation. The writing committee discussed all recommendations and the evidence that informed them before voting. Initial failure to reach consensus was resolved by subsequent discussions, revisions as needed, and re-voting. Although the consensus threshold was set at 67%, all recommendations were approved by at least 80% of the writing committee members. The mean consensus over all recommendations was 95%. A quorum of two-thirds of the writing committee was met for all votes [6].

Each recommendation in this document was assigned a Class of Recommendation (COR) and a Level of Evidence (LOE) according to the system developed by ACC and AHA (Table 1) [7]. The COR denotes the strength of the recommendation based on a careful assessment of the estimated benefits and risks; COR I indicates that the benefit of an intervention far exceeds its risk; COR IIa indicates that the benefit of the intervention moderately exceeds the risk; COR IIb indicates that the benefit may not exceed the risk; and COR III indicates that the benefit is equivalent to or is exceeded by the risk. The LOE reflects the quality of the evidence that supports the recommendation. LOE A is derived from high-quality randomized controlled trials (RCTs); LOE B-R is derived from moderate-quality RCTs; LOE B-NR is derived from well-designed nonrandomized studies; LOE C-LD is derived from randomized or nonrandomized studies with limitations of design or execution; and LOE C-EO indicates that a recommendation was based on expert opinion [7].

**Table 1:**
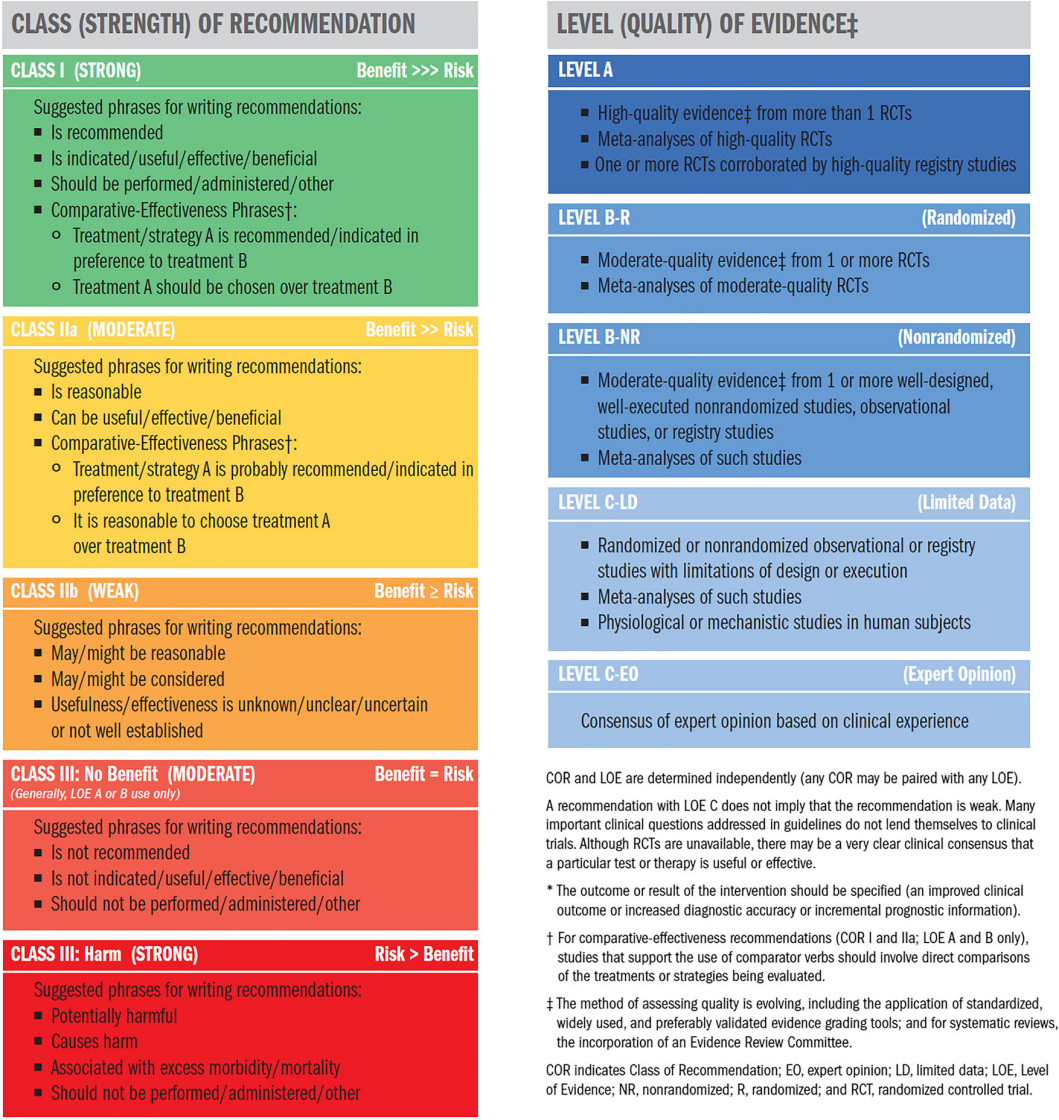
ACC/AHA Recommendation System: Applying Class of Recommendation and Level of Evidence to Clinical Strategies, Interventions, Treatments, and Diagnostic Testing in Patient Care*

Reproduced with permission of the American College of Cardiology (ACC) and the American Heart Association (AHA) [7]

**Fig. 1:**
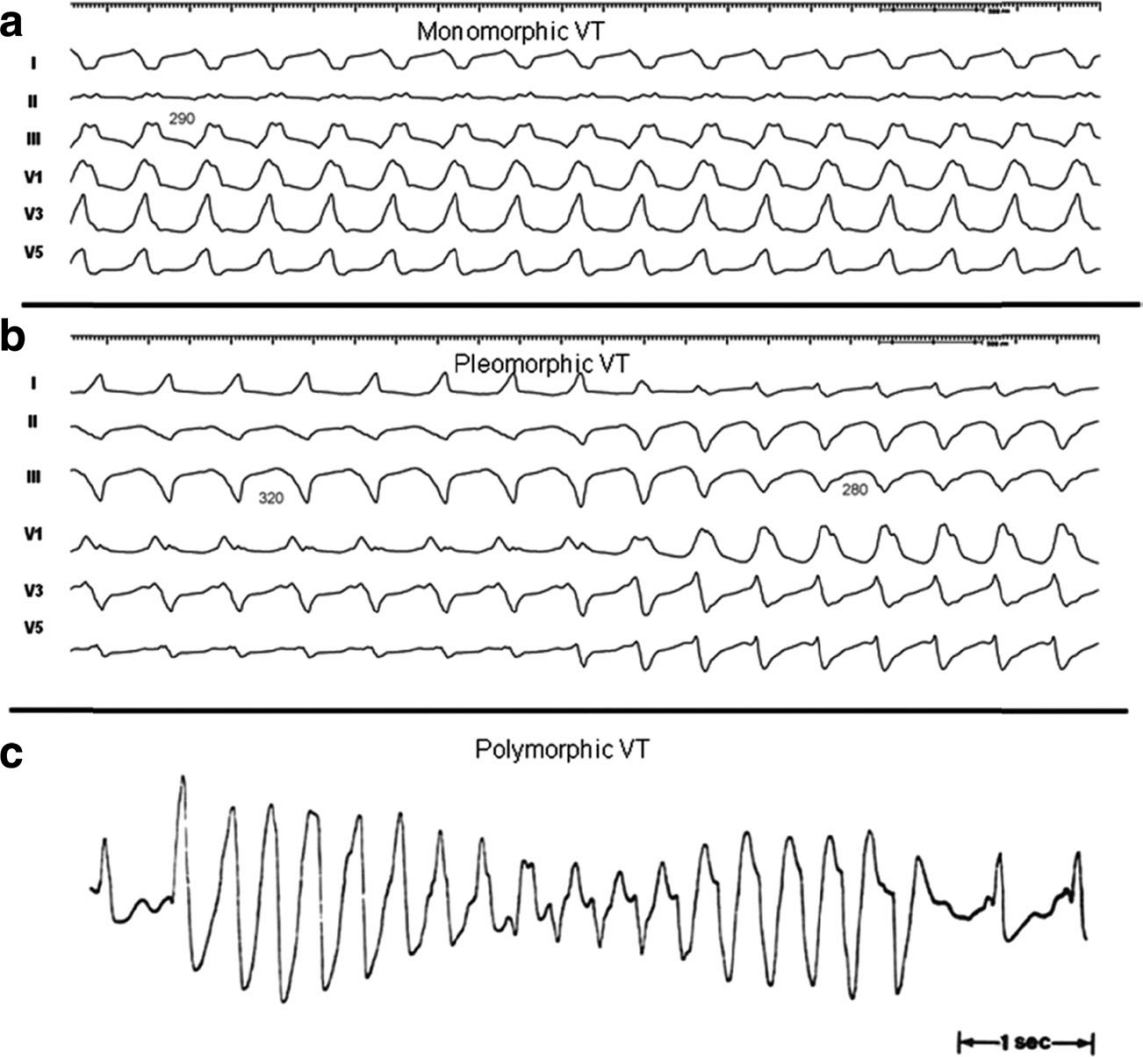
Monomorphic (**a**), pleomorphic (**b**), and polymorphic (**c**) VT. Reproduced with permission of the Heart Rhythm Society [1]. VT = ventricular tachycardia

Unique to this consensus statement is the systematic review commissioned specifically for this document as part of HRS’s efforts to adopt the rigorous methodology required for guideline development. The systematic review was performed by an experienced evidence-based practice committee based at the University of Connecticut, which examined the question of VT ablation vs control in patients with VT and ischemic heart disease (IHD) [8]. The question, in PICOT format, was as follows: In adults with history of sustained VT and IHD, what is the effectiveness and what are the detriments of catheter ablation compared with other interventions? Components of the PICOT were as follows: P = adults with history of sustained VT and IHD; I = catheter ablation; C = control (no therapy or antiarrhythmic drug [AAD]); O = outcomes of interest, which included 1) appropriate implantable cardioverter defibrillator (ICD) therapies (ICD shock or antitachycardia pacing [ATP]), 2) appropriate ICD shocks, 3) VT storm (defined as three shocks within 24 h), 4) recurrent VT/VF, 5) cardiac hospitalizations, and 6) all-cause mortality; and T = no time restrictions.

An industry forum was conducted to achieve a structured dialog to address technical questions and to gain a better understanding of future directions and challenges. Because of the potential for actual or perceived bias, HRS imposes strict parameters on information sharing to ensure that industry participates only in an advisory capacity and has no role in either the writing of the document or its review.

The draft document underwent review by the HRS Scientific and Clinical Documents Committee and was approved by the writing committee. Recommendations were subject to a period of public comment, and the entire document underwent rigorous peer review by each of the participating societies and revision by the Chairs, before endorsement.

## Background

### History of ventricular arrhythmia ablation

In 1959, Couch [9] reported the elimination of VT with the resection of a postinfarction left ventricular (LV) aneurysm. In the early to mid-1970s, standard LV aneurysmectomy was performed for patients with preoperative VT episodes in the setting of prior infarction. Unfortunately, the operative mortality rates were high and VT recurrences were frequent [10]. Endocardial encircling ventriculotomy, introduced by Guiraudon et al. [11], was designed to isolate the arrhythmogenic tissue from the remainder of the ventricle by creating a nearly transmural incision through the edge of the border zone, sparing only the epicardium. This operation was associated with marked postoperative LV dysfunction, likely due to interference with coronary arterial blood supply. Guiraudon et al. [12] also attempted to disarticulate the right ventricular (RV) free wall from the rest of the ventricles in patients with arrhythmogenic right ventricular cardiomyopathy (ARVC). Although the surgery was successful in isolating the arrhythmogenic RV free wall and in producing interesting 12-lead electrocardiogram (ECG) recordings of sinus rhythm simultaneous with persistent sustained VT in the same patient, most patients ultimately did poorly because of progressive RV failure. In the late 1970s, Josephson et al. [13] developed the technique of map-guided subendocardial resection. This procedure was based on the observation that diastolic or presystolic electrical activation could be recorded during VT on the endocardium near or within the border between the densely scarred aneurysm or infarct and more normal muscle [14–16]. The surgical procedure thus targeted areas specified by mapping. As originally practiced, subendocardial resection removed segments of endocardium approximately 3 mm thick and 5 cm^2^ [13]. These areas were almost always within regions of visibly scarred endocardium, extending from the edge of a densely scarred aneurysm. As the procedure evolved, a more extensive area of resection was typically performed because of the ease of defining a single plane of resection with the goal of eliminating other arrhythmogenic areas within the visual scar. Adjunctive cryoablation was applied to locations that were not easily resected, such as the papillary muscles or the deep myocardial layers beneath the removed subendocardium when VT was localized to these regions. Cryoablation targeting an isthmus of surviving myocardium between a more densely scarred inferior infarction and the mitral annulus improved the outcome of VT ablation associated with this substrate [17]. More extensive cryoablation of the entire visually scarred endocardial surface was also used with some success [18]. Although success rates approached 90% with surgery in terms of VT elimination, a mortality rate of 5%–15% limited the procedure to a few select patients [19].

In 1983, endocardial catheter ablation of VT using direct current energy electrical shock delivered via the distal electrode of a standard quadripolar endocardial catheter positioned in the area to be modified was first described by Hartzler [20]. One of the largest early studies was by Fontaine et al. [21], who referred to direct current shock ablation as fulguration and reported their results in 43 patients. One to 8 R-wave-synchronous shocks of preselected energy ranging from 160 to 320 joules were delivered per session, with 23 patients undergoing at least one repeat procedure. Of note, a success rate of 87% in preventing VT recurrence was achieved, and no deaths were thought to be related to the endocardial shock itself. Strategies for analyzing the 12-lead ECG during VT and pace mapping to mimic the QRS of VT were first described in the early 1980s to help to regionalize areas of interest for more detailed activation mapping for surgical or early catheter-based VT ablation [22–24].

Concern about barotrauma and the need for general anesthesia with direct current shock ablation led to the use of radiofrequency (RF) energy for catheter ablation for all arrhythmias, including VT, by the end of the 1980s [25, 26]. The safety and short-term effectiveness of RF catheter ablation for VT occurring either in the absence or presence of structural heart disease (SHD) was first reported in observational reports by Klein et al. [27] and Morady et al. [28], respectively. Activation mapping to identify diastolic activation coupled with entrainment mapping techniques to identify critical components of the VT circuit ultimately proved most useful to define a critical isthmus through which a VT circuit must pass. This isthmus identification allowed for successful targeted ablation using RF ablation techniques for hemodynamically tolerated VT [29–33]. Using both computer simulations and catheter mapping of stable VT in humans, Stevenson et al. [30] elucidated a schematic model of the postinfarction VT circuit that endures.

Unfortunately, detailed activation and entrainment mapping is not always feasible when VT is hemodynamically poorly tolerated [34, 35]. A successful substrate-based ablation strategy that did not require detailed mapping of VT was first described by Marchlinski et al. [36, 37]. Linear ablation created by sequential point lesions transected the border zone, extending into the region of dense infarction defined by detailed bipolar voltage mapping with a color-coded display on a three-dimensional (3D) mapping system. The mapping system facilitated the ability to track lesion deployment. The location of the ablation line was guided by analyzing the 12-lead QRS of VT and by pace mapping to mimic the QRS complex. Subsequent substrate-based VT targets, which were reported to be effective surrogates of the VT circuit, included 1) late potentials (LPs); 2) channels defined by high voltage surrounded by lower voltage or by areas of pace capture surrounded by myocardium that could not be captured at 10 mA pacing output; 3) local abnormal ventricular activity (LAVA) that could demonstrate more abnormality with pacing; 4) paced map QRS morphologies that matched VT and demonstrated a long stimulus to QRS duration; and 5) regions in which pace mapping demonstrates abrupt transition in paced QRS morphologies [38–49]. More recently, isolation of abnormal myocardium demonstrating critical components of the VT circuit or extensive direct ablation of all low-voltage areas have been reported as successful techniques for possibly improving substrate-based ablation outcome [50, 51]. The integration of anatomical imaging of ventricular myocardial scar by computed tomography (CT) or cardiac magnetic resonance imaging (CMR) with electroanatomical mapping (EAM) has further contributed to the ability to recognize and eliminate disrupted and potentially slowly conducting regions of myocardium that are critical to the maintenance of VT.

The documentation of basal, perivalvular, low-voltage scar serving as the substrate for VT in nonischemic LV and RV cardiomyopathy focused attention on these regions for VT localization [52–54]. The basal involvement frequently included the septum, and not uncommonly the substrate was intramurally located in the septum or midmyocardial with epicardial extension if located in the free wall [55–57].

The percutaneous technique for accessing the pericardial space to allow mapping of the epicardium as described by Sosa et al. [58] provided the opportunity to define the epicardial substrate in patients with SHD. Epicardial mapping and ablation proved particularly valuable in patients with nonischemic RV and LV cardiomyopathy, where the predominant substrate and VT circuits are frequently located [59–64]. Endocardial unipolar voltage mapping helped to identify the probable epicardial substrate when normal endocardial bipolar voltage was demonstrated in patients with VT and nonischemic cardiomyopathy (NICM) [65, 66]. The value of epicardial mapping and ablation in select patients with postinfarction and idiopathic VT has also been demonstrated [67–69].

In an attempt to overcome the biophysical limitations of lesion formation in scar, irrigated ablation for VT was introduced with closed-loop, internal irrigation in the late 1990s [70]. This was followed by reports of even more extensive experience with open irrigated catheter ablation [71, 72]. More recently, techniques have been described to further enhance lesion formation in scar and/or deep to the endocardium, including alcohol infusion in the coronary arteries or coronary veins; bipolar and simultaneous unipolar ablation at both endocardial and epicardial sites; ablation with near freezing saline; half normal saline as the irrigant; and needle electrode ablation [73–78]. Simultaneously, small, multipolar electrode recording techniques have been proven to further enhance the accuracy of activation and entrainment mapping [79–81].

Idiopathic VT ablation with RF ablation also evolved from the initial catheter ablation experience. The most common anatomical sites of origin of frequent PVCs and VF triggers were described [82]. Twelve-lead ECG QRS assessment provided reasonably precise characterization of origin for these focal arrhythmias occurring in the absence of SHD, with an emphasis on clues to identify left versus RV outflow tract (RVOT) origin and epicardial origin [83]. New techniques to overcome the challenges of idiopathic VT ablation associated with the sinuses of Valsalva (SV), the coronary venous system, the LV summit, and papillary muscle arrhythmias have been described [84–87]. The importance of PVC-induced cardiomyopathy has been recognized, and the potential for improvement in LV function with ablation has been demonstrated [88, 89].

Of note, this brief historical summary of VT ablation provides only an overview. There have been many important contributions related to VT ablation, the details of which will be further highlighted elsewhere in this document.

### Mechanisms of ventricular arrhythmia

#### Mechanisms and basis for catheter ablation of ventricular tachycardia

Catheter ablation has an important role in reducing or preventing VAs both in patients with heart disease and in those with idiopathic VTs not associated with SHD. The approach to ablation and the efficacy are determined by the characteristics of the arrhythmia and the anatomy and location of the arrhythmia substrate, which can often be anticipated from the ECG of the VT and the nature of any underlying heart disease. Focal VTs are susceptible to ablation with discrete RF lesions [90–97]. Relatively large scar substrates requiring more extensive ablation are common in VT associated with SHD; however, VT origin can appear focal if the reentry circuit is small, or if it is due to a focal endocardial breakthrough from an epicardial or intramural reentry circuit. Automatic VTs can also occur in some patients with SHD and ventricular scars.

Focal VT has a point source of earliest ventricular activation with a spread of activation away in all directions from that site. The mechanism can be automaticity, triggered activity, or microreentry. Focal origin arrhythmias should be particularly suspected in patients without SHD who have repetitive monomorphic and nonsustained VTs and PVCs or who have sustained VT from the outflow tract (OT) and other more stereotypical sites of origin [95–97]. A focal origin is confirmed by mapping that shows spread of activation away in all directions from the site of earliest activation relative to the QRS onset. Unipolar unfiltered (or minimally high pass filtered at 0.5 Hz) electrograms typically display a QS configuration at the site of origin (SOO) [98, 99]. Pacing at the origin will replicate the VT/PVC QRS morphology if the origin is on the surface; however, matching pace maps are frequently found within 1 cm of the site of earliest activation. Pace mapping is particularly unreliable for VTs originating from the aortic sinuses [100].

#### Triggered activity and automaticity

Triggered activity arises from oscillations in membrane potential during (early afterdepolarizations) or following (delayed afterdepolarizations) an action potential and can give rise to focal VA. Experimental evidence implicates early afterdepolarizations in the initiation of polymorphic tachycardias in long QT syndromes [101]. However, the mechanism of the premature ventricular beats targeted for ablation in these syndromes is unknown [102].

Delayed afterdepolarizations can be caused by intracellular calcium overload, which activates the Na^+^/Ca^2+^ exchanger, resulting in the transient inward current *I*_ti_ [103]. Factors that increase intracellular calcium include increases in heart rate, beta-adrenergic stimulation, and digitalis. Beta-adrenergic effects are mediated through a cyclic adenosine monophosphate (cAMP)-induced increase in intracellular calcium and are antagonized by adenosine, which effects a decrease in cAMP. Termination of idiopathic RVOT tachycardias by an intravenous bolus of adenosine, by infusion of calcium channel blockers, or by vagotonic maneuvers is consistent with triggered activity as the likely mechanism for some of these tachycardias [92]. These tachycardias can be difficult to induce at electrophysiology testing; rapid burst pacing and/or isoproterenol infusion is often required. Aminophylline, calcium infusion, and atropine can also be useful [91].

Less commonly, focal VT can be due to automaticity provoked by adrenergic stimulation that is not triggered [91, 103]. This type of VT can become incessant under stress or during isoproterenol administration, and it cannot be initiated or terminated by programmed electrical stimulation (PES); however, it can sometimes be suppressed by calcium channel blockers or beta blockers. In contrast to its effects on triggered RVOT tachycardia, adenosine transiently suppresses, but does not terminate, the arrhythmia. Automaticity from damaged Purkinje fibers has been suggested as a mechanism for some catecholamine-sensitive, focal origin VTs [104, 105]. Whether these VTs are due to abnormal automaticity, originating from partially depolarized myocytes, as has been shown for VTs during the early phase of myocardial infarction (MI), is not clear [106].

Although automaticity is frequently considered as a mechanism of VT in the absence of overt SHD, disease processes that diminish cell-to-cell coupling are likely to facilitate automaticity [107]. Automatic VTs can occur in SHD, and automatic premature beats can initiate reentrant VTs.

#### Scar-related reentry

Scar-related reentry is the most common cause of sustained monomorphic VT in the presence of SHD [108]. Evidence supporting reentry includes initiation and termination by programmed stimulation (although this does not exclude triggered activity), demonstrable entrainment or resetting with fusion, and continuous electrical activity that cannot be dissociated from VT by extrastimuli [14, 33]. Prior MI is the most common cause of the substrate, but scar-related VT also occurs in other myocardial diseases, including ARVC, sarcoidosis, Chagas disease (ChD), dilated cardiomyopathy (DCM) including laminopathies, and after cardiac surgery for congenital heart disease (CHD) (particularly, tetralogy of Fallot) or valve replacement [54, 109–114].

Regions of fibrosis with surviving myocyte bundles create fixed and/or functional conduction block and disrupted or slow conduction that are the substrate for reentry. Stable circuits can be modeled as having an isthmus or channel comprised of a small mass of tissue that does not contribute to the surface ECG. QRS onset occurs when the excitation wave front emerges from an exit along the border of the scar and spreads across the ventricles [30, 35]. Scars associated with VT are often close to a valve annulus and together can form the borders of the isthmus of a VT circuit [115, 116]. The 3D structure of the reentry circuit and substrate can be subendocardial, intramural, or subepicardial, or it can span the width of the entire ventricular wall [117, 118]. The entire circuit or only a portion of it might be accessible to ablation.

The substrate supporting scar-related reentry is characterized by 1) regions of slow conduction; 2) unidirectional conduction block at some point in the reentry path that allows initiation of reentry; and 3) areas of conduction block that often define parts of the reentry path. Some of the substrate might exhibit functional rather than fixed conduction block [119–121]. VT after MI has been extensively studied in canine models and in humans [119, 122]. Reentry occurs through surviving muscle bundles, commonly located in the subendocardium; however, this can also occur in the midmyocardium and epicardium. Evidence has shown ongoing ion channel remodeling within scar, at least early after MI, resulting in regional reductions in ionized sodium and ionized calcium currents [123], although action potential characteristics of surviving myocytes late after infarction can be normal or near normal [122]. Coupling between myocyte bundles and myocytes is reduced by increased collagen and connective tissue, diminished gap junction density, and alterations in gap junction distribution, composition, and function [124]. Surviving fibers can be connected by side to side connections in regions where the collagenous sheaths are interrupted, resulting in a “zig-zag” pattern of transverse conduction along a pathway lengthened by branching and merging bundles of surviving myocytes [125]. The fibrosis pattern might be important in determining the degree of conduction delay; patchy fibrosis between strands of surviving muscle produces greater delay than diffuse fibrosis [120]. These aspects of scar remodeling contribute to the formation of channels and regions in which conduction time is prolonged, facilitating reentry [126].

Unidirectional conduction block can occur after a properly timed PVC and is often functional [119, 127, 128]; it can present only during tachycardia, when the refractory period of the tissue exceeds the tachycardia cycle length (CL) or is maintained by collision of excitation waves. Regions of conduction block can also be anatomically fixed such that they are present during tachycardia and sinus rhythm; dense, nonexcitable fibrosis, calcifications, surgical scars, or valve annuli create these types of anatomical boundaries for reentry [39, 115, 116]. Multiple VTs with various QRS morphologies can be due to multiple exits from the same region of scar, or to changes in activation remote from the circuit due to functional regions of block. Ablation at one region can abolish more than one VT. Multiple reentry circuits from widely separated areas also occur.

It is possible that other reentry mechanisms cause some VTs. Spiral wave reentry can be induced in excitable tissue in the absence of tissue discontinuities and could cause VF or polymorphic VT [129]; anchoring to a discontinuity or to a region of slow conduction could theoretically cause monomorphic VT [130].

#### Reentry in the Purkinje system and ventricular fibrillation

Reentry within the Purkinje fibers and the specialized conduction system is a particular form of reentry and is covered in detail in Section 9.4. Other nonreentrant arrhythmias involving the Purkinje system can also occur, including VF and automatic rhythms [105, 131–133]. PVCs initiating VF most often originate from the Purkinje fiber system. Structural abnormalities in the vicinity of the Purkinje fibers are frequently present and facilitate the anchoring of reentry [134]. However, even in the absence of detectable structural alterations, VF can be initiated by PVCs from the Purkinje fiber system [135] and can be maintained in the complex fiber interaction between Purkinje and myocardial fibers located in the papillary muscles [136]. The latter situation can be operative in some patients who have idiopathic VF, in whom no structural abnormalities can be detected with current technology. Some structural abnormalities, however, have also recently been described in patients with idiopathic VF, when high-density mapping is performed during sinus rhythm revealing abnormal electrograms in a confined area located in the epicardium [137]. This potential substrate, although not usually detected by imaging, was reported to colocalize with areas where VF drivers were identified by mapping. Interestingly, in most of these patients with idiopathic VF, VF was still triggered by PVCs originating from the Purkinje fiber system [137].

### Definitions

The previous EHRA/HRS expert consensus on catheter ablation of VA in 2009 proposed several definitions to standardize nomenclature in the field [1]. The current consensus statement repeats the majority of these recommendations for VT ablation. In the last 10 years, knowledge and experience of PVC ablations have significantly increased. In the current report, new proposals are made to facilitate understanding of clinical characteristics and reporting of the ablation outcomes of these arrhythmias (Table 2). Note that different cutoff rates for VT and (accelerated) idioventricular rhythm could be appropriate for children, who have a higher resting sinus rate than adults: the mechanism, symptoms, and clinical setting of the VA are more important than the rate [138].

**Table 2:** Definitions

**Clinical Characteristics**	
***Clinical ventricular tachycardia (VT)***: VT that has occurred spontaneously based on analysis of 12-lead ECG QRS morphology.	
***Hemodynamically unstable VT***: causes hemodynamic compromise requiring prompt termination.	
***Idiopathic VT***: used to indicate VT that is known to occur in the absence of clinically apparent SHD.	
***Idioventricular rhythm***: three or more consecutive beats at a rate of up to 100 per minute that originate from the ventricles independent of atrial or atrioventricular (AV) nodal conduction. Although various arbitrary rates have been used to distinguish it from VT, the mechanism of ventricular rhythm is more important than the rate. Idioventricular rhythm can be qualified as “accelerated” when the rate exceeds 40 bpm.	
***Incessant VT***: continuous sustained VT that recurs promptly despite repeated intervention for termination over several hours.	
***Nonclinical VT***: VT induced by PES that has not been documented previously.	
***Nonsustained VT***: terminates spontaneously within 30 s.	
***PVC***: premature ventricular complex; it is an early ventricular depolarization with or without mechanical contraction. We recommend avoiding the use of the terms “ventricular premature depolarization” and “premature ventricular contraction” to standardize the literature and acknowledge that early electrical activity does not necessarily lead to mechanical contraction.	
***Presumptive clinical VT***: similar to a spontaneous VT based on rate, limited ECG, or electrogram data available from ICD interrogation, but without the 12-lead ECG documentation of spontaneous VT.	
***PVC burden***: the amount of ventricular extrasystoles, preferably reported as the % of beats of ventricular origin of the total amount of beats over a 24-h recording period.	
***Repetitive monomorphic VT***: continuously repeating episodes of self-terminating nonsustained VT.	
***Sustained VT***: continuous VT for 30 s, or which requires an intervention for termination (such as cardioversion).	
***VT***: a tachycardia (rate > 100 bpm) with 3 or more consecutive beats that originates from the ventricles independent of atrial or AV nodal conduction.	
***VT storm***: three or more separate episodes of sustained VT within 24 h, each requiring termination by an intervention.	
**VT Morphologies**	
***Monomorphic VT***: a similar QRS configuration from beat to beat (Fig. 1a). Some variability in QRS morphology at initiation is not uncommon, followed by stabilization of the QRS morphology.	
***Monomorphic VT with indeterminate QRS morphology***: preferred over *ventricular flutter*; it is a term that has been applied to rapid VT that has a sinusoidal QRS configuration that prevents identification of the QRS morphology.	
***Multiple monomorphic VTs***: more than one morphologically distinct monomorphic VT, occurring as different episodes or induced at different times.	
***Pleomorphic VT***: has more than one morphologically distinct QRS complex occurring during the same episode of VT, but the QRS is not continuously changing (Fig. 1b).	
***Polymorphic VT***: has a continuously changing QRS configuration from beat to beat, indicating a changing ventricular activation sequence (Fig. 1c).	
***Right bundle branch block (RBBB)- and left bundle branch block (LBBB)-like VT configurations***: terms used to describe the dominant deflection in V1, with a dominant R wave described as “RBBB-like” and a dominant S wave with a negative final component in V1 described as “LBBB-like” configurations.	
***Torsades de pointes***: a form of polymorphic VT with continually varying QRS complexes that appear to spiral around the baseline of the ECG lead in a sinusoidal pattern. It is associated with QT prolongation.	
***Unmappable VT***: does not allow interrogation of multiple sites to define the activation sequence or perform entrainment mapping; this could be due to hemodynamic intolerance that necessitates immediate VT termination, spontaneous or pacing-induced transition to other morphologies of VT, or repeated termination during mapping.	
***Ventricular fibrillation (VF)***: a chaotic rhythm defined on the surface ECG by undulations that are irregular in both timing and morphology, without discrete QRS complexes.	
**PVC Morphologies**	
***Monomorphic PVC***: PVCs felt reasonably to arise from the same focus. Slight changes in QRS morphology due to different exit sites from the same focus can be present.	
***Multiple morphologies of PVC***: PVCs originating from several different focal locations.	
***Predominant PVC morphology***: the one or more monomorphic PVC morphologies occurring most frequently and serving as the target for ablation.	
**Mechanisms**	
***Focal VT***: a point source of earliest ventricular activation with a spread of activation away in all directions from that site. The mechanism can be automaticity, triggered activity, or microreentry.	
***Scar-related reentry***: arrhythmias that have characteristics of reentry that originate from an area of myocardial scar identified from electrogram characteristics or myocardial imaging. Large reentry circuits that can be defined over several centimeters are commonly referred to as “macroreentry.”	

*AV* atrioventricular; *ECG* electrocardiogram; *ICD* implantable cardioverter defibrillator; *LBBB* left bundle branch block; *PES* programmed electrical stimulation; *PVC* premature ventricular complex; *RBBB* right bundle branch block; *SHD* structural heart disease; *VT* ventricular tachycardia

### Standard anatomical terminology

The following are the suggested anatomical terminology for use in the description of catheter ablation of VA (Table 3). While these generally represent the most commonly used terms, the writing committee recognizes that several variants or alternatives are in use and may also be valid.

**Table 3:** Anatomical terminology

Term	Definition
RV inflow	The part of the RV containing the tricuspid valve, chordae, and proximal RV.
RV outflow tract (RVOT)	The conus or infundibulum of the RV, derived from the bulbus cordis. It is bounded by the supraventricular crest and the pulmonic valve.
Tricuspid annulus	Area immediately adjacent to the tricuspid valve, including septal, free wall, and para-Hisian regions.
Moderator band	A muscular band in the RV, typically located in the mid to apical RV, connecting the interventricular septum to the RV free wall, supporting the anterior papillary muscle. It typically contains a subdivision of the right bundle branch (RBB).
RV papillary muscles	Three muscles connecting the RV myocardium to the tricuspid valve via the tricuspid chordae tendineae, usually designated as septal, posterior, and anterior papillary muscles. The septal papillary muscle is closely associated with parts of the RBB.
Supraventricular crest	Muscular ridge in the RV between the tricuspid and pulmonic valves, representing the boundary between the conus arteriosus and the rest of the RV. The exact components and terminology are controversial; however, some characterize it as being composed of a parietal band that extends from the anterior RV free wall to meet the septal band, which extends from the septal papillary muscle to meet it.
Pulmonary valves	The pulmonic valve includes three cusps and associated sinus, variously named right, left, and anterior; or anterolateral right, anterolateral left, and posterior sinuses. The posterior-right anterolateral commissure adjoins the aorta (junction of the right and left aortic sinuses). Muscle is present in each of the sinuses, and VA can originate from muscle fibers located within or extending beyond the pulmonary valve apparatus.
Sinuses of Valsalva, aortic cusps, aortic commissures	The right (R), left (L), and noncoronary aortic valve cusps are attached to the respective SV. The left sinus of Valsalva (LSV) is posterior and leftward on the aortic root. The noncoronary sinus of Valsalva (NCSV) is typically the most inferior and posterior SV, located posterior and rightward, superior to the His bundle, and anterior and superior to the paraseptal region of the atria near the superior AV junctions, typically adjacent to atrial myocardium. The right sinus of Valsalva (RSV) is the most anterior cusp and may be posterior to the RVOT infundibulum. VAs can also arise from muscle fibers at the commissures (connections) of the cusps, or from myocardium accessible to mapping and ablation from this location, especially from the RSV/LSV junction.
LV outflow tract (LVOT)	The aortic vestibule, composed of an infra-valvular part, bounded by the anterior mitral valve leaflet, but otherwise not clearly distinguishable from the rest of the LV; the aortic valve; and a supra-valvular part.
LV ostium	The opening at the base of the LV to which the mitral and aortic valves attach.
Aortomitral continuity (AMC); aortomitral curtain, or mitral-aortic intervalvular fibrosa	Continuation of the anteromedial aspect of the mitral annulus to the aortic valve; a curtain of fibrous tissue extending from the anterior mitral valve leaflet to the left and noncoronary aortic cusps. The AMC is connected by the left and right fibrous trigones to ventricular myocardium, the right fibrous trigone to the membranous ventricular septum.
Mitral valve annulus	Area immediately adjacent to the mitral valve. This can be approached endocardially, or epicardially, either through the coronary venous system or percutaneously.
LV papillary muscles	Muscles connecting the mitral valve chordae tendineae to the LV, typically with posteromedial and anterolateral papillary muscles. Papillary muscle anatomy is variable and can have single or multiple heads.
LV false tendon (or LV moderator band)	A fibrous or fibromuscular chord-like band that crosses the LV cavity, attaching to the septum, papillary muscles, trabeculations, or free wall of the LV. They may contain conduction tissue and may impede catheter manipulation in the LV.
Posterior-superior process	The posterior-superior process of the LV is the most inferior and posterior aspect of the basal LV, posterior to the plane of the tricuspid valve. VAs originating from the posterior-superior process of the LV can be accessed from the right atrium, the LV endocardium, and the coronary venous system.
Endocardium	Inner lining of the heart.
Purkinje network	The specialized conduction system of the ventricles, which includes the His bundle, RBB and left bundle branches (LBB), and the ramifications of these, found in the subendocardium. The Purkinje system can generate focal or reentrant VTs, typically manifesting Purkinje potentials preceding QRS onset.
Interventricular septum	Muscular wall between the LV and RV.
Membranous ventricular septum	The ventricular septum beneath the RSV and NCSV, through which the penetrating His bundle reaches the ventricular myocardium.
LV summit	Triangular region of the most superior part of the LV epicardial surface bounded by the left circumflex coronary artery, the left anterior descending artery, and an approximate line from the first septal coronary artery laterally to the left AV groove. The great cardiac vein (GCV) bisects the triangle. An area superior to the GCV is considered to be inaccessible to catheter ablation due to proximity of the coronary arteries and overlying epicardial fat.
Crux of the heart (crux cordis)	Epicardial area formed by the junction of the AV groove and posterior interventricular groove, at the base of the heart, approximately at the junction of the middle cardiac vein and coronary sinus (CS) and near the origin of the posterior descending coronary artery.
Epicardium	The outer layer of the heart—the visceral layer of the serous pericardium.
Epicardial fat	Adipose tissue variably present over the epicardial surface around coronary arteries, LV apex, RV free wall, left atrial appendage, right atrial appendage, and AV and interventricular grooves.
Pericardial space or cavity	The potential space between the parietal and visceral layers of serous pericardium, which normally contains a small amount of serous fluid. This space can be accessed for epicardial procedures.
Parietal pericardium	The layer of the serous pericardium that is attached to the inner surface of the fibrous pericardium and is normally apposed to the visceral pericardium, separated by a thin layer of pericardial fluid.
Fibrous pericardium	Thick membrane that forms the outer layer of the pericardium.
Subxiphoid area	Area inferior to the xiphoid process; typical site for percutaneous epicardial access.
Phrenic nerve	The right phrenic nerve lays along the right atrium and does not usually pass over ventricular tissue. The course of the left phrenic nerve on the fibrous pericardium can be quite variable and may run along the lateral margin of the LV near the left obtuse marginal artery and vein; inferior, at the base of the heart; or anterior over the sternocostal surface over the L main stem coronary artery or left anterior descending artery.
Coronary sinus (CS) and branches	The CS and its branches comprise the coronary venous system with the ostium of the CS opening into the right atrium. Tributaries of the CS, which runs along the left AV groove, may be used for mapping. These include the anterior interventricular vein (AIV), which arises at the apex and runs along the anterior interventricular septum, connecting to the GCV that continues in the AV groove to the CS; the communicating vein located between aortic and pulmonary annulus; various posterior and lateral marginal branches or perforator veins; and the middle cardiac vein that typically runs along the posterior interventricular septum from the apex to join the CS or empty separately into the right atrium. The junction of the GCV and the CS is at the vein or ligament of Marshall (or persistent left superior vena cava, when present), and the valve of Vieussens (where present).

Anatomical terminology [139–147]. See also Figs. 3, 4, 7, and 8

*AIV* anterior interventricular vein; *AMC* aortomitral continuity; *AV* atrioventricular; *CS* coronary sinus; *GCV* great cardiac vein; *LBB* left bundle branch; *LSV* left sinus of Valsalva; *LV* left ventricle; *LVOT* left ventricular outflow tract; *NCSV* noncoronary sinus of Valsalva; *RBB* right bundle branch; *RSV* right sinus of Valsalva; *RV* right ventricle; *RVOT* right ventricular outflow tract; *SV* sinus of Valsalva; *VA* ventricular arrhythmia; VT = ventricular tachycardia

## Clinical evaluation

### Clinical presentation

**Table:**
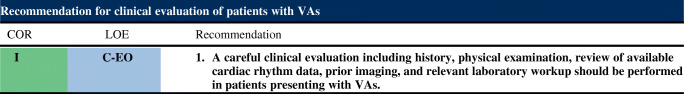

#### Recommendation-specific supportive text

1. History should identify the onset, duration, frequency, and trigger of any symptoms and should include medication use as well as comorbidities and family history. Available cardiac rhythm data include interrogation of cardiovascular implantable electronic devices (CIEDs) to assess arrhythmia burden, morphologies, and duration as well as treatment. Electrogram storage may be programmed to include far- and near-field electrograms to allow superior assessment of VA morphologies. The laboratory workup should be individualized to the patient’s presentation and may include electrolytes, troponin, brain natriuretic peptide, genetic testing, or drug screening as appropriate.

#### Synopsis

The clinical presentations of patients with VAs encompass a wide spectrum, ranging from asymptomatic to VT/VF storm or sudden cardiac death [4].

Presenting symptoms can be classified into 5 groups: due to the VA itself (eg, PVCs, VT or VF); due to a secondary disease caused by the VAs (eg, PVC-induced cardiomyopathy); due to an underlying pathology associated with the VAs (eg, ischemia); due to ICD therapy; and a combination of these causes.

Idiopathic VA is frequently asymptomatic, especially when presenting as PVCs or nonsustained VT. In those cases, VAs are commonly detected coincidentally during routine exams. If symptomatic, symptoms can often be secondary to post-PVC augmentation of contractility or a post-PVC compensatory pause, and commonly consist of palpitations, dizziness, shortness of breath, fatigue, or chest discomfort. With increasing duration or VA rate (eg, VT or VF), hemodynamic compromise can result in more severe symptoms, such as pre-syncope, syncope, or even sudden cardiac death [4, 148].

Secondary diseases caused by VA include PVC-induced cardiomyopathy, which can present with typical symptoms of heart failure and reduced ejection fraction (EF) [149, 150]. If PVCs are asymptomatic, the diagnosis is commonly made by a routine physical exam and is confirmed by a 12-lead ECG.

Underlying pathologies resulting in VA are numerous and include ischemia [151]; cardiomyopathy [152]; genetic diseases (eg, inherited arrhythmia syndromes) [153]; hypertrophic cardiomyopathy (HCM) [154]; ARVC [155]; CHD [156]; infiltrative, inflammatory, or infectious diseases [157]; and correctible causes, such as electrolyte abnormalities or medication adverse effects [158]. If VAs themselves are asymptomatic, the presenting symptoms will mostly depend on the underlying pathology and might include chest pain, heart failure, dizziness, syncope, and sudden cardiac death. A careful history and physical exam with a review of the family history, ECG, imaging, and laboratory data [159] will direct diagnosis and specific treatment (eg, immunosuppression in cardiac sarcoidosis) [157]. If inherited arrhythmia syndromes are suspected (eg, long QT syndrome), genetic testing should be considered [153].

ICD therapy including shocks is an increasingly common presentation of VAs in patients with CIEDs, and appropriate ICD therapy occurs in the first year in >50% of patients with secondary and approximately 5% of patients with primary prevention ICDs [160, 161].

Combined presentations of those scenarios are common, such as worsening heart failure status with increased arrhythmias burden [149, 150] or acute MI presenting with sudden cardiac death as a manifestation of the VAs [151].

Given that presenting symptoms of VA vary widely, careful documentation and correlation of the specific arrhythmia (ECG, telemetry, Holter or event monitor, electrograms) with the presenting symptoms is important to guide further workup and therapy. Symptoms commonly attributed to VA (eg, palpitations, dizziness, chest pain, syncope) are nonspecific and can either be due to other arrhythmias [162] (eg, supraventricular tachycardia [SVT] or bradycardia), other cardiac diseases, noncardiac conditions, anxiety, or have no clear identifiable cause [163].

### Diagnostic evaluation

#### Resting 12-lead electrocardiogram

**Table:**
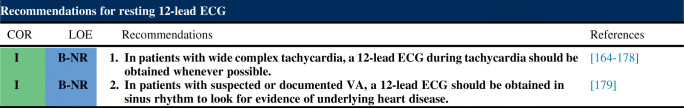

##### Recommendation-specific supportive text

1. A 12-lead ECG during tachycardia is the first diagnostic test that should be performed for any patient with a stable, wide, QRS complex tachycardia to differentiate VT from SVT prior to attempts to terminate the tachycardia. Criteria that support a diagnosis of VT include AV dissociation, a QRS complex >0.14 s, monophasic R wave in aVR, positively or negatively concordant QRS complexes in the precordial leads, the absence of an RS complex in all precordial leads, and an RS interval > 100 ms in at least 1 precordial lead [164–166]. For patients with preexisting bundle branch block, comparison of the QRS morphology during sinus rhythm with QRS morphology during wide complex tachycardia is important. Various QRS morphologies (eg, bundle branch block pattern) strongly support the diagnosis of VT. An identical QRS complex during sinus rhythm and broad QRS tachycardia, however, does not rule out the presence of bundle branch reentry (BBR) tachycardia. Patients without SHD can present with idiopathic VT (eg, fascicular VT) that can be easily recognized by 12-lead ECG [167]. For nonsustained VAs (PVCs or nonsustained VT), the 12-lead QRS morphology is critical to allow for identification of the SOO. Idiopathic VAs (eg, right and left OT VAs, PVCs from the aortic SV, papillary muscle VAs) can be recognized, given they exhibit characteristic ECG patterns (see Section 5.2) [168–173].
2. A 12-lead ECG during sinus rhythm is helpful to evaluate the presence of underlying heart disease and might be a clue for scar location and origins of related VAs, such as inferior wall or anterior wall Q waves. An inherited arrhythmia disorder can also be identified, such as ARVC (epsilon waves and/or inverted T waves in right precordial leads), long QT syndrome, Brugada syndrome (coved-type ST segment elevation in the right precordial leads), and ChD (right bundle branch block [RBBB] and/or left anterior hemiblock) [155]. In patients with SHD, QRS duration and the presence of conduction abnormalities might provide additional prognostic information [180–185].

#### Assessment of structural heart disease and myocardial ischemia

**Table:**
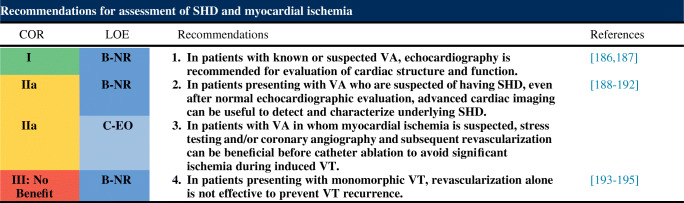

##### Recommendation-specific supportive text

1. Assessment of global and regional myocardial function, valvular structure and function, along with testing for adult CHD is required in patients with or at high risk for VA or sudden cardiac death. Echocardiography is the most readily available and commonly used imaging technique [186, 187]. Accurate assessment of LV ejection fraction (LVEF) using CMR is hindered by the presence of frequent PVCs [196]. In these patients, echocardiographic evaluation of LVEF may be superior to CMR.
2. Advanced cardiac imaging, such as cardiac CT, CMR, and fluorodeoxyglucose positron emission tomography (PET), is useful for the evaluation of SHD and assessment of LV and RV function [188–192]. CMR with assessment of late gadolinium enhancement (LGE) is the gold standard technique for determination of location and the extent of scarring. This information has implications for planning the ablation strategy (see Section 5.4) [192, 197]. The use of this imaging technique is limited in some patients with CIEDs [4] (see Section 5.4). Additional myocardial inflammation or infiltrative diseases can be detected with CMR or fluorodeoxyglucose PET [157].
3. Transient myocardial ischemia is a known cause of polymorphic rather than monomorphic sustained VT. Monomorphic VT in the setting of prior MI is typically due to scar-related reentry and not due to acute ischemia. For patients suspected to have myocardial ischemia, stress testing and/or coronary angiography and subsequent revascularization should be performed when possible before catheter ablation to avoid significant ischemia during VT induction, mapping, and ablation.
4. Revascularization alone is unlikely to reduce the recurrence of monomorphic VT [193–195]. However, revascularization might be beneficial in patients with IHD and VF, polymorphic VT, or exercise-induced arrhythmias associated with ischemia [198]. Revascularization for prognostic indications may also be indicated.

#### Risk stratification in the setting of frequent premature ventricular complexes

**Table:**
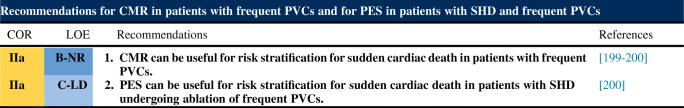

##### Recommendation-specific supportive text

1. CMR has been reported to identify patients at increased risk for adverse outcomes in the presence of frequent PVCs [199, 200]. One study assessed the value of CMR in an Italian patient population with frequent LBBB PVCs [199]. Patients without RV CMR abnormalities had better outcomes than patients with CMR abnormalities. Another study assessed the benefit of CMR for risk stratification in patients with frequent PVCs undergoing ablation procedures for PVCs [200]. Except for 1 patient who had inducible idiopathic VT, 14 of 15 patients with inducible, sustained, monomorphic VT had scarring identified by CMR. All patients with inducible VT except the patient with idiopathic VT underwent ICD implantation, and 50% had appropriate ICD therapy during follow-up.
2. Programmed stimulation has been reported to identify patients at increased risk for adverse outcomes in the presence of frequent PVCs (the PVC burden was 20% ± 13% in the cited study), but without prior documented VT, and who are undergoing PVC ablation procedures [200]. All but one patient with inducible VT had SHD prompting ICD implantation, and 50% of the patients had appropriate ICD therapy during follow-up [200].

##### Synopsis

LVEF continues to be the main prognostic clinical variable for VA. The presence and extent of myocardial fibrosis, assessed by CMR-LGE, predict ventricular tachyarrhythmias in patients with ischemic and nonischemic LV dysfunction [201–206]. In a meta-analysis including 2850 patients with IHD and nonischemic heart disease from 19 studies, the composite arrhythmic endpoint was significantly higher in the patients with LGE (annualized event rate of 8.6%) than in the patients without LGE (annualized event rate of 1.7%; *P* < .0001) [206]. In a larger meta-analysis including 7882 patients from 36 studies (both ischemic cardiomyopathy [ICM] and NICM), LGE was associated with an increase in all-cause mortality (hazard ratio [HR] 2.96; 95% CI 2.37–3.70; *P* < .001), cardiovascular mortality (HR 3.27; 95% CI 2.05–5.22; *P* < .001), VA and sudden cardiac death (HR 3.76; 95% CI 3.14–4.52; *P* < .001), and major adverse cardiovascular events (HR 3.24; 95% CI 2.32–4.52; *P* < .001) [204]. In both studies, the predictive value of LGE was independent of LVEF and whether the cardiomyopathy was of ischemic or nonischemic etiology.

Patients with PVC-induced cardiomyopathy show improvement (even normalization) of LVEF after effective PVC treatment. As opposed to patients with NICM, the absence of LGE is a common finding in these patients; thus, absence of LGE could be used to identify patients with greater chance of LVEF recovery [207]. Patients with frequent PVCs in the presence of LGE still have a possibility of LVEF improvement [208] post ablation, although the LVEF might not completely normalize [209]. An RBBB morphology of the PVC has been associated with an increased prevalence of LGE-defined fibrosis [210]. This finding has prognostic implications. CMR has been reported to identify patients at increased risk for adverse outcomes in the presence of frequent PVCs [199, 200]. In addition, CMR provides important information about the underlying fibrotic substrate and facilitates ablation procedure planning. Inducible VT can have prognostic implications in patients with frequent PVCs and LGE-CMR. Over 80% of the writing committee members perform programmed stimulation to induce VT at the time of PVC or VT ablation in patients without known SHD.

The arrhythmogenic substrate can also be recognized by voltage mapping at the time of the procedure. Low-voltage areas have been correlated with scar tissue identified as LGE-CMR [211, 212]. Although OT PVCs typically occur in patients with normal heart, identification of low-voltage areas and transitional zones could provide helpful information at the time of ablation [213]. Two-thirds of the writing committee members perform voltage mapping of the relevant ventricle at the time of PVC or VT ablation in patients without known SHD.

#### Longitudinal follow-up in the setting of frequent premature ventricular complexes

**Table:**
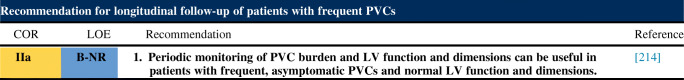

##### Recommendation-specific supportive text

1. Frequent PVCs can be associated with the development of cardiomyopathy in susceptible individuals. Despite extensive study, the predictors of future deterioration of LV function are unclear. In one study of 249 patients, all of whom were followed for at least 4 years, none developed overt congestive heart failure, but the LVEF decreased in 20% of patients with very frequent PVCs (>20,000 per 24 h) [214]. Therefore, until the development of PVC-induced cardiomyopathy can be predicted with more precision, periodic measurement of LVEF and LV end-diastolic dimensions, along with quantification of PVC burden, may be useful for patients with a high PVC burden [approximately 10% or higher [215]] to identify deteriorating LV function before symptoms appear.

## Indications for catheter ablation

Following are the consensus recommendations for catheter ablation of VAs organized by underlying diagnosis and substrate. These recommendations are each assigned a COR and an LOE according to the current recommendation classification system [216]. In drafting each of these recommendations, the writing committee took into account the published literature in the specific area, including the methodological quality and size of each study, as well as the collective clinical experience of the writing group when published data were not available. Implicit in each recommendation are several points: 1) the procedure is being performed by an electrophysiologist with appropriate training and experience in the procedure and in a facility with appropriate resources; 2) patient and procedural complexity vary widely, and some patients or situations merit a more experienced operator or a center with more capabilities than others, even within the same recommendation (eg, when an epicardial procedure is indicated and the operator or institution has limited experience with this procedure, it might be preferable to refer the patient to an operator or institution with adequate experience in performing epicardial procedures); 3) the patient is an appropriate candidate for the procedure (as outlined in Section 5.1), recognizing that the level of patient suitability for a procedure will vary widely with the clinical scenario; and 4) the patient’s (or designee’s) informed consent, values, and overall clinical trajectory are fundamental to a decision to proceed (or not) with any procedure. Therefore, in some clinical scenarios, initiation or continuation of medical therapy instead of an ablation procedure may be the most appropriate option, even when a class 1 recommendation for ablation is present. There may also be scenarios not explicitly covered in this document, and on which little or no published literature is available, in which the physician and patient must rely solely on their own judgment.

In drafting these recommendations, the writing committee also referenced several other relevant clinical documents, including the *2017 AHA/ACC/HRS Guideline for Management of Patients with Ventricular Arrhythmias and the Prevention of Sudden Cardiac Death* [4], among others. The exclusive focus of the current document on VA ablation led to the opportunity to develop more detailed and nuanced recommendations.

### Idiopathic outflow tract ventricular arrhythmia

**Table:**
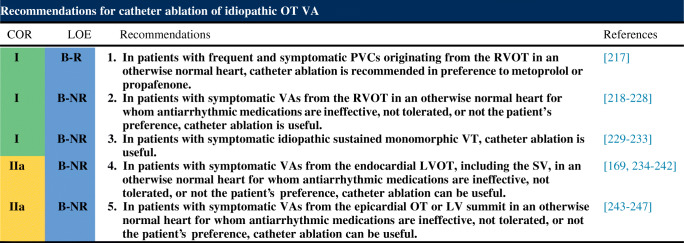

#### Recommendation-specific supportive text

1. In symptomatic patients with frequent PVCs from the RVOT, catheter ablation had a higher rate of efficacy than pharmacotherapy with either metoprolol or propafenone in an RCT [217]. Ablation success rates are reported at 80%–95%, with low complication rates [213, 217, 228, 237, 248, 249]. Catheter ablation can be considered as a preferred therapy in suitable, symptomatic patients. However, some patients with minimal or tolerable symptoms might prefer medical therapy or no therapy.
2. RVOT and LVOT are the most common SOOs for idiopathic VA in patients without SHD. VAs arising from these locations mostly present with a unique pattern on 12-lead surface ECG. The most common underlying pathophysiological mechanism is triggered activity, and RF catheter ablation is highly effective and has low complication rates [218–228]. Multiple studies have shown that for RVOT VAs, catheter ablation is effective for prevention of arrhythmia recurrences.
3. In patients with symptomatic, idiopathic, sustained monomorphic VT, catheter ablation might be preferable to medical therapy. It is a more definitive treatment option, given its high success and low recurrence rates [229–233].
4. LVOT VA is reported to account for 12%–45% of all idiopathic VAs [234–238]. Compared with VAs originating from the RVOT, ablation of LVOT VAs is more complex [234–238]. Rarely, LVOT VA sites require epicardial ablation via the GCV/AIV or subxiphoid puncture. Clinically, ablation of LVOT VA can involve greater procedural complexity as well as periprocedural risk (stroke or coronary artery injury) compared with RVOT VA. However, many studies report good results pertaining to the safety, feasibility, and potential curative ability of RF catheter ablation [234–238].
5. Although most idiopathic VAs originate from the RVOT or LVOT, in some cases, RF catheter ablation cannot successfully be performed from either site. In such cases, the VAs might originate from the LV summit (see Section 2 for definition). VAs originating from this area can present challenges for successful RF catheter ablation, and the failure rate is high due to epicardial fat and the proximity of coronary arteries if a subxiphoid epicardial access is used [230–232]. Appropriate patient selection for this approach is key, and the initial approach should focus on the endocardium and adjacent structures, including the coronary venous system, the aortic cusps, and the RVOT.

### Idiopathic nonoutflow tract ventricular arrhythmia

**Table:**
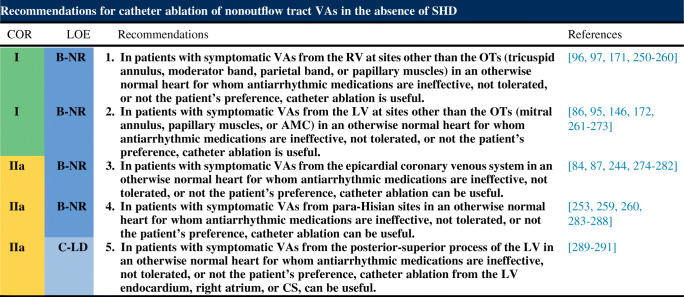

#### Recommendation-specific supportive text

1. Among idiopathic RV arrhythmias presenting for catheter ablation, approximately 10%–15% arise at sites other than the RVOT [250]. Sites that can be ablated with a high level of success include any of the three RV papillary muscles [171, 252], the parietal band [253, 255–257], the tricuspid annulus [96, 254, 258, 259], and the moderator band [251, 260]. Although frequent PVCs, nonsustained VT, and sustained monomorphic VT are the most common idiopathic VAs at these sites of origin, VF triggered by PVCs arising from the moderator band [251] can occasionally occur. Successful catheter ablation is achieved in over 90% of patients with RV VAs arising outside the RVOT, with a low risk of complications. The recurrence rate and need for repeat procedures are higher for VAs arising from the moderator, septal, and parietal bands than from other sites [251, 253]. In addition, the probability of successful ablation is higher for tricuspid annular VAs arising from the free wall than the septal regions, which are closer to the conduction system [96].
2. Idiopathic VAs arising from the LV papillary muscles account for approximately 15% of idiopathic LV arrhythmias referred for catheter ablation and are characterized by frequent PVCs or recurrent monomorphic VT with a catecholamine-sensitive focal mechanism. VT can arise from either the posteromedial papillary muscles or the anterolateral papillary muscles [86, 172, 261–267]. These arrhythmias often require several RF applications for successful ablation. A change in QRS morphology of the VA after ablative applications to either side of the involved papillary muscle is common. Due to the thickness of the papillary muscles and their vigorous contraction, catheter stability can be challenging and might be improved by the use of intracardiac ultrasound and possibly cryoablation [262, 266]. The recurrence risk after initial successful ablation of papillary muscle VAs is higher than for many other idiopathic VA sites, and repeat procedures are required in approximately 30% of patients [172, 262].
  The mitral annulus is the SOO for approximately 20% of idiopathic LV arrhythmias, with the majority being PVCs or nonsustained VT rather than sustained VT [268, 269]. Mitral annular VAs are based on a focal, catecholamine-sensitive mechanism. A superior-anterior mitral annular origin is more common than an inferior-posterior origin [95, 267–269]. Successful ablation is achieved in approximately 90% of mitral annular VAs, with a very low risk of complications [95, 146, 267–271]. The AMC is a common location of mitral annular VAs at the base of the LV ostium. Most patients with VA from the AMC have frequent PVCs rather than sustained VT [272]. Endocardial mapping demonstrates a prepotential in the majority of VAs originating in this location, with successful ablation in over 90% of patients with a low risk of complications [272, 273].
3. VAs that can be mapped and ablated within the GCV or AIV are relatively common SOOs near or within the LV summit [84, 87, 244, 274, 275]. Proximity to the coronary arteries needs to be assessed (see Sections 9.3 and 11.1) prior to ablation. If the SOO is too close (<5–10 mm) to a coronary artery, ablation from the adjacent endocardium might be successful when within 1 cm of the SOO [276]. These arrhythmias from the superior portion of the coronary venous system are usually based on a focal mechanism and typically require an irrigated-tip ablation catheter due to high impedance within the coronary vein. Mapping and ablation from the LSV or LV endocardium might also be successful, even if the local ECG in the coronary venous system is earlier [277]. For VAs with an intramural location, ablation within the perforator veins from within the LV septum [278], simultaneous bipolar or simultaneous unipolar RF energy from the coronary venous system and the LV endocardium might be required to achieve successful ablation.
  Sustained monomorphic VT arising from the crux of the heart is typically very rapid, based on a focal catecholamine-sensitive mechanism, and often produces syncope [279–282]. The QRS morphology is characterized by an abrupt transition from negative in lead V1, to positive in lead V2, to more negative in lead V3 [279]. Ablation can be achieved within the coronary venous system, including the middle cardiac vein [279, 280, 282] and the adjacent endocardium, or might require epicardial access [279–281]. The proximity of the coronary venous system to the posterior descending coronary artery requires imaging to prevent arterial injury.
4. VAs arising from the RV septum near the His bundle can be successfully ablated in approximately 70%–90% of patients, with several series reporting a higher likelihood of abandoning attempts at ablation due to concerns about inducing AV block [253, 259, 260, 283–288]. The appearance of an accelerated junctional rhythm is common during RF application in this region, though AV block is not [253]. Catheter stability and the need to prevent injury to the AV node are issues that are carefully considered for these VAs [288]. Pacing maneuvers may be beneficial to prevent damage to the AV node [292]. The careful use of cryoablation can help to prevent damage to the conduction system if ablative therapy is delivered in close proximity to the conduction system.
5. The posterior-superior process of the LV is the most inferior and posterior aspect of the basal LV, posterior to the plane of the tricuspid valve [289]. Ablation of VAs in this region can be accomplished from the LV endocardium [290]. However, VAs arising from this region can also be successfully ablated from the inferior septal surface of the right atrium, where a small atrial signal and a larger ventricular signal, earlier than the earliest site in the LV endocardium, can be recorded [289]. Reports also describe ablation from within the CS [291]. Successful ablation can be achieved with either RF or cryoablation energies. The risk of complications appears to be low, although data are limited, and caution is required due to the proximity to the AV node and the AV nodal artery [289].

### Premature ventricular complexes with or without left ventricular dysfunction

**Table:**
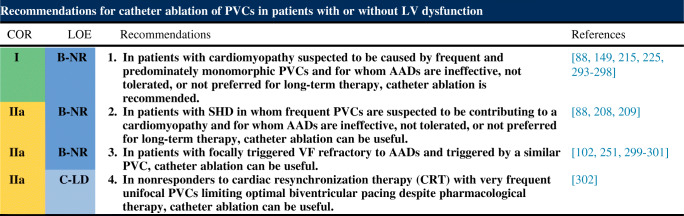

#### Recommendation-specific supportive text

1. In patients with a suspected PVC-induced cardiomyopathy, catheter ablation can be considered an alternative to long-term AAD therapy, particularly for patients with monomorphic PVCs, or PVCs of an RVOT origin [293]. PVC-induced cardiomyopathy should be suspected when cardiomyopathy and frequent PVCs are present and a comprehensive cardiac evaluation fails to identify alternate etiologies. Several studies have confirmed a correlation between a higher PVC burden and development of cardiomyopathy, although no precise burden of PVCs consistently predicts the development of a cardiomyopathy. Several studies have demonstrated a higher incidence of cardiomyopathy with PVC burdens >15%–25% [88, 215, 288, 303]; however, patients with similarly high PVC burdens can also maintain normal cardiac function. Reversible PVC-induced cardiomyopathy has also been reported in patients with PVC burdens as low as 4%–5% [149, 304]. When PVC-induced cardiomyopathy is suspected in these patients with relatively low PVC burdens, other etiologies for cardiomyopathy should be thoroughly investigated and addressed, and the persistence of the PVC burden established. Other risk factors for the development of a PVC-induced cardiomyopathy to consider include epicardial origin of PVCs, PVCs with a longer QRS duration, longer exposure to PVCs, asymptomatic PVCs, interpolated PVCs, and male sex [293, 296, 305, 306]. In patients with PVC-induced cardiomyopathy, PVC ablation has an overall reported success rate of 65%–90%, with a low complication rate [88, 149, 225, 293, 295–298]. Predictors of ablation success include an RVOT PVC origin and a uniform PVC morphology. Following successful ablation, the majority of patients with PVC-induced cardiomyopathy experience significant improvement and, possibly, normalization of their LVEF. Based on a review of current data, routine catheter ablation of frequent PVCs in asymptomatic patients without evidence of LV dysfunction is not presently recommended. In some asymptomatic patients with very frequent PVCs and preserved cardiac function who express a strong preference for ablation, approximately half of the writing committee members agreed that an ablation can be considered after adequate counseling on the risks, benefits, and alternatives to ablation, whereas half would not offer ablation. Additional clinical features that may portend worsening LV function that should be considered include LV dilation or a relative decrease in EF that may still be considered in the normal range.
2. In patients with underlying SHD, the presence of frequent PVCs can contribute to a cardiomyopathy, and successful PVC elimination by ablation can improve cardiac function [88, 208, 209]. Detailed cardiac imaging (such as CMR to quantify scarring) in conjunction with a comprehensive cardiac history, including the time course of cardiomyopathy development and onset of PVCs, can help to estimate the contribution of the PVCs to the cardiomyopathy and guide treatment decisions targeting the PVCs.
3. In patients with recurrent VF refractory to antiarrhythmic medications and triggered by PVCs from a potentially identifiable site, successful ablation of the PVC can lead to VF suppression [299–301]. The triggering PVC is often located in the Purkinje system and can exhibit slight variations in morphology. It is important to recognize that despite an initially successful ablation, PVC and VF recurrences are possible, and recurrent PVCs can arise from an alternate focus [300]. Successful ablation, therefore, often does not eliminate the need for an ICD.
4. In patients who are clinical nonresponders to CRT, with limited biventricular pacing due to frequent PVCs despite pharmacological therapy, successful PVC ablation has demonstrated an improvement in heart failure class and a modest improvement in LVEF. In one prospective multicenter study in which CRT nonresponders with frequent and primarily monomorphic PVCs were ablated, the improvements in LV function and New York Heart Association (NYHA) class were proportional to the preprocedural PVC burden, and the greatest improvement was noted in patients with a preablation PVC burden >22% [102].

### Ventricular arrhythmia in ischemic heart disease

**Table:**
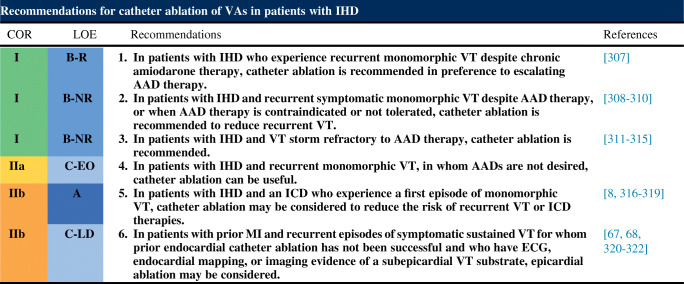

#### Recommendation-specific supportive text

1. For patients presenting with VT despite AAD therapy, which often results in ICD therapies (shocks or ATP), therapeutic options include escalating AAD therapy by increasing the dose, changing the drug, adding a new drug, and catheter ablation. The VANISH trial [307] compared the strategies of escalating AAD therapy according to a predefined protocol versus catheter ablation in 259 patients and followed them for a mean (± standard deviation [SD]) of 27.9 ± 17.1 months. The composite primary endpoint of death, VT storm, or appropriate ICD shock was reduced by catheter ablation. Benefit was observed in the subgroup on amiodarone at baseline, for whom escalated drug therapy was an increase in amiodarone or an addition of mexiletine (HR 0.55; 95% CI 0.38–0.80; *P* = .001), but not in the subgroup of patients who were on sotalol at baseline, for whom escalation of drug therapy was initiation of amiodarone (HR 1.14; 95% CI 0.65–2.02; *P* = .64) [307]. Further analysis found that catheter ablation was markedly superior to the addition of mexiletine in a small subgroup of patients with VT refractory to high dose (≥300 mg daily) amiodarone [323]. There were no procedure-related deaths.
2. In addition to the VANISH trial described above [307], 3 large, prospective, multicenter cohort studies have examined the role of catheter ablation in reducing recurrent VT in patients with IHD, most of whom had recurrent VT despite AAD therapy [308–310]. In these trials, the patient served as his/her own control, and VT episodes were reported for the 6 months before and the 6 months after ablation. In each study, a consistent reduction in VT episodes was observed post ablation. In the Euro-VT study, VT recurrence occurred in 49% of the patients [309]. A reduction in device therapies (ATP and shocks) was observed in 79% of these patients, with the mean number of therapies falling from 60 ± 70 in the 6 months prior to ablation to 14 ± 15 in the same period of time after ablation (*P* = .02). In the Multicenter Thermocool VT Ablation Trial, and the similarly designed Post-Approval THERMOCOOL VT Trial, the number of VT episodes in the 6 months before and after ablation was reduced from a median of 11.5 to 0 episodes (*P* < .0001), and from 13 to 0 episodes (*P* < .0001), respectively [308, 310].
3. There are limited comparative data on catheter ablation vs other therapies in the management of VT storm. AAD therapy is almost universally used as first-line therapy for these patients; however, catheter ablation is a particularly important therapy when VA recurs. Two small, single-center, nonrandomized studies found that patients treated with ablation had a lower risk of recurrence than those treated medically [324], or they had a similar risk of recurrence but lower mortality [325]; however, these studies were limited by their retrospective study design and the small number of patients. Several other large series [311–314] and a systematic review and meta-analysis [315] have shown a reduction in ICD therapies after catheter ablation and reasonable success in controlling arrhythmia, with a nonetheless significant mortality. A common finding is that unsuccessful ablation (persistent VT inducibility) is associated with poor outcomes.
4. Patients who do not tolerate or are unsuitable for AAD therapy have not generally been included in RCTs. However, the writing committee felt it was reasonable to recommend catheter ablation in such patients over no therapy, in view of the reduction in recurrent VT demonstrated in multiple clinical trials. Examples of contraindications to AAD therapy include significant renal dysfunction or QT prolongation in the case of sotalol, and severe pulmonary disease, which might be worsened by amiodarone.
5. Four RCTs have examined the role of catheter ablation in patients who have experienced their first episode of VT [8, 316–319]. In these studies, an ICD has been a mandatory part of the protocol. Two of these trials have shown a reduction in the primary endpoint with catheter ablation [316, 317]. The CALYPSO pilot study found an increased time to first VT recurrence with ablation versus AADs [318]. The Substrate Modification Study (SMS) failed to meet its primary endpoint of time to first VT/VF recurrence; however, catheter ablation reduced the total number of ICD interventions [319]. A meta-analysis of these trials, commissioned to guide recommendations in this consensus document, showed a reduction in the risk of appropriate ICD therapies with catheter ablation [8]. However, in drafting this recommendation, the writing committee also considered that only the CALYPSO pilot study randomized patients to either catheter ablation or AAD therapy [318]. The other trials [316, 317, 319] compared catheter ablation without specifying antiarrhythmic therapy in the control group. Prospective trials have not shown that catheter ablation reduces mortality, and the risks of the procedure must be carefully weighed against the benefits in this population. In a large study using the US National Inpatient Sample database, the overall rate of any in-hospital complication was 11.2% in patients with prior MI undergoing catheter ablation, and in-hospital mortality was 1.6%, without changes over a decade [326]. The VANISH2 trial is currently comparing these two strategies in patients with prior MI and VT while not on AAD therapy [327]. There are limited data exploring the feasibility of managing VT with catheter ablation without an ICD in selected patients with IHD and relatively preserved LV function [328–330]. Of 302 patients with IHD who underwent a catheter ablation procedure sufficiently successful to make ICD implantation unnecessary, approximately 3% experienced sudden death during 3–4 years of follow-up [328–330]. The writing group did not feel there was sufficient evidence to make a recommendation regarding ablation as a stand-alone therapy without an ICD for patients with IHD and sustained VT.
6. It has long been recognized that the epicardial surface can contain critical elements of VT circuits in patients with IHD [67, 320]. However, the VT substrate can be accessed from the endocardium in most cases, and endocardial mapping and ablation have been the mainstay of therapy. A number of studies have shed light on the incidence of epicardial substrate in IHD. In the initial description of epicardial mapping and ablation in patients with inferior wall MI, an epicardial circuit was found in 7 of 30 VTs [67]. In another series of 11 patients with 1 to 4 prior unsuccessful endocardial ablation procedures, epicardial access was successful in 10, and 7 required epicardial ablation, which abolished the clinical VT in all patients. However, 40% had recurrence during follow-up, and there was one periprocedural death [321]. Two larger, single-center observational studies have reported similar or improved VT-free survival in patients with IHD undergoing combined endocardial and epicardial ablation [331, 332]. A prospective study of patients undergoing catheter ablation for electrical storm, in which epicardial access was obtained in all patients in one arm being treated with a scar homogenization technique (*n* = 43), found that ablation was performed in one-third. However, whether the epicardial substrate participated in clinical or inducible VT was not assessed [322]. There are limited data on the use of combined endocardial and epicardial ablation as a first-line approach in patients with IHD and VT [68]; an RCT is currently examining this question [333]. Preprocedural imaging can be useful to determine the presence of epicardial substrate, and therefore the likelihood of requiring epicardial access for complete substrate ablation [334].

### Nonischemic cardiomyopathy

**Table:**
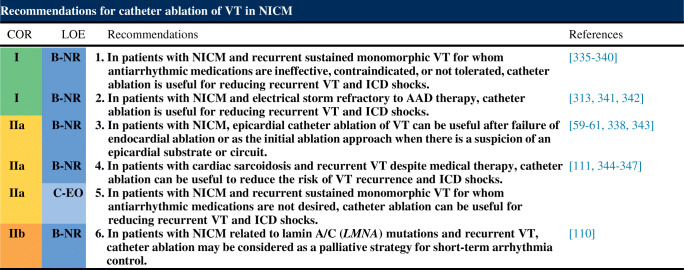

#### Recommendation-specific supportive text

1. Patients with NICM represent a heterogeneous population with diverse etiologies, including undifferentiated dilated NICM and valvular, hypertensive, hypertrophic, toxic, genetic, inflammatory, tachycardia-induced, peripartum, and infiltrative cardiomyopathy. Several retrospective and prospective cohort studies have shown VT-free survival of 40.5% to 70% at 1 year post ablation [335–340].

VTs in the context of NICM are more often pleomorphic and polymorphic; they are less hemodynamically tolerated, are more difficult to induce, and are faster compared with postinfarction VT [348, 349]. Differences in the results of VT catheter ablation between NICM and ICM could be due to a substrate that is less favorable to ablation with NICM [350]. The substrate is often intramurally located and can involve the epicardium; it is typically located at the basal perivalvular regions and at the interventricular septum. The intramural substrate makes the arrhythmogenic substrate less accessible for identification of optimal ablation target sites based on pace mapping and identification of abnormal electrograms.

Although sustained VT occurs in only 5% of patients with NICM, it is an important cause of sudden cardiac death [351]. Recurrent VT is associated with significantly increased mortality in NICM [352].

2. Electrical storm with recurrent VT/VF episodes in patients with NICM and ICD can adversely affect hospitalization rates and long-term survival [352, 353]. Catheter ablation of patients with NICM and electrical storm refractory to, or intolerant of, AADs eliminates recurrent VT in most cases [313, 341, 342]. Whether ablation reduces mortality in this population is unclear at present.
3. In multicenter registries, 30% of epicardial VT catheter ablations were performed on patients with NICM [60, 61]. Epicardial mapping and catheter ablation can significantly reduce VT recurrence in patients with NICM [337, 338] and long-term success rates reach 55% to 70% after epicardial ablation [59, 61]. However, due to an increased risk of complications or late adhesions preventing future pericardial accesses, epicardial ablation may be reserved to first-line endocardial approach failures [335], except when ECG or imaging suggests a predominant epicardial substrate [335]. Consideration should be given to the ability to proceed to epicardial access during the initial procedure.
4. Cardiac sarcoidosis is associated with a poor prognosis. VT is frequently resistant to AADs and immunosuppression, requiring ICDs. Recurrent VT is common in cardiac sarcoidosis and is associated with a poorer prognosis. Catheter ablation decreases VT recurrence and ICD shocks in cardiac sarcoidosis [111, 344–347].
5. AADs are not as effective as the ICD at decreasing mortality in patients with SHD and sustained VAs. Even if successful at acutely terminating VT or VF, ICD shocks have been associated with increased heart failure and mortality in NICM [354, 355]. To date, there has been no RCT comparing catheter ablation with AADs for preventing VT recurrences in NICM. Indirect comparisons have found similar reductions in VT recurrences with catheter ablation or amiodarone in patients with SHD [356]. Amiodarone might increase mortality in patients with cardiomyopathy [356, 357] and can cause serious long-term adverse effects. Catheter ablation of VT appears to be safe and effective in NICM, achieving long-term arrhythmia control in 50% to 70% [335, 340, 351, 358] (with AADs in one-third), with a survival rate of 70% [335, 340]. Retrospective series show that acute success of catheter ablation and freedom from recurrent VT after catheter ablation are associated with a significant reduction in mortality and heart transplantation in NICM [335, 340, 359, 360].
6. Mutations in the lamin A/C (*LMNA*) gene are an important cause of NICM, with a disease course more aggressive than other forms of idiopathic NICM in terms of progression to end-stage heart failure or the tendency toward malignant VAs [361, 362]. Catheter ablation can be beneficial as a palliative strategy to allow transient arrhythmia control in patients with NICM related to *LMNA* mutations before aggressive heart failure management is proposed [110]. However, acute and chronic success are low, due to frequent intramural substrate, and complication rates are high in the published literature [110]. Disease progression to end-stage heart failure requiring mechanical support or transplant is also common, and VT ablation might be best targeted toward those with relatively earlier stage disease, rather than being a means to delay progression in those approaching end-stage heart failure.

### Ventricular arrhythmia involving the His-Purkinje system, bundle branch reentrant ventricular tachycardia, and fascicular ventricular tachycardia

**Table:**
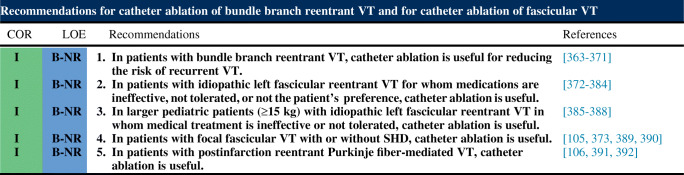

#### Recommendation-specific supportive text

1. Bundle branch reentrant VT (BBRVT) is curable by catheter ablation, which is successful in almost all patients [363–366]. However, for patients with SHD or conduction system disease, long-term outcome depends on the type of underlying cardiac disease. Despite successful BBRVT ablation, many patients remain at risk of sudden death due to concomitant scar-related VTs and/or LV dysfunction. An ICD with or without cardiac resynchronization should be considered [366–368]. On the other hand, for patients with BBRVT who have unacceptably frequent shocks from their ICD, catheter ablation can be helpful in preventing or reducing the shocks [365]. Even for patients with terminal heart failure and incessant BBRVT, catheter ablation can function as a bridge to cardiac transplantation [365].

BBRVT can be cured by catheter ablation for patients with normal heart structure and function [365–367, 369, 370]. Although data are limited, some reports suggest that patients with preserved LV systolic function and no other inducible VT might not further benefit from ICD insertion after successful ablation. This subgroup had excellent long-term outcomes for VT-free survival without deterioration of conduction properties during follow-up. However, further study is required before a specific recommendation can be formulated [366].

For patients with BBRVT who have a normal HV interval, catheter ablation is also a curative approach. Approximately 22%–46% of patients with BBRVT had a normal baseline HV interval, and significant prolongation of the HV interval only developed during tachycardia. This result suggests that either a functional or fixed conduction block in the His-Purkinje system could be sufficient to maintain a BBRVT mechanism. Long-term outcome depends on the underlying cardiac disease [366, 368]. Patients with normal LV systolic function and HV interval have excellent long-term outcomes after ablation [366].

Myocardial VT is the most common type of inducible sustained monomorphic VT in patients with valvular heart disease. The majority of these patients have underlying coronary artery disease and significant LV dysfunction. However, in almost one-third of the patients, sustained BBRVT is the only type of inducible VT. This type of VT is facilitated by the valve procedure occurring within 4 weeks after surgery in most patients. In these patients, LV function is relatively well preserved, and the RBBB-type of BBRVT is frequently induced. Because a curative intervention can be offered to these patients (eg, bundle branch ablation), BBRVT should be seriously considered as the VT mechanism in patients with valvular heart disease, particularly if the arrhythmia occurs soon after valve surgery [367].

Catheter ablation of the RBB or LBB interrupts the circuit and is usually curative. However, severely impaired antegrade conduction in the remaining bundle is often present, requiring permanent pacing, which can have hemodynamic consequences. Consideration should be given to future CIED requirements when selecting the ablation target. In patients with baseline complete or incomplete LBBB, anterograde slow conduction over the LBB is present, and ablation of the LBB might leave adequate residual anterograde conduction over the right bundle. Ablation targeting the portion of the distal LBB (left posterior or anterior fascicle) that has slow conduction can potentially preserve the residual AV conduction. Close follow-up for recurrent arrhythmias, progressive conduction deterioration, or LBBB-induced ventricular dysfunction is important [371].

2. In the published literature, the overall success rate of ablation for idiopathic left fascicular reentrant VT is >95%. Although complications related to left heart catheterization might be encountered, no serious complications were reported. Left posterior hemiblock was observed when a line of RF lesions was applied through the posterior septal region [372–384].

Left upper septal fascicular VT often presents as narrow QRS complex tachycardia, but some left upper septal fascicular VTs occur after catheter ablation targeting left posterior fascicular VT. Such VTs can be managed successfully by focal ablation at the left upper septum with some risk of fascicular injury [384].

3. In pediatric patients with fascicular reentrant VT (age 10.0 ± 5.1 years, 62% males), nondihydropyridine calcium channel blockers were effective at terminating and preventing VT in 80% of the patients; however, 21% of the patients experienced recurrence during chronic oral therapy. Catheter ablation was successful in 71% of fascicular VTs. After a follow-up period of 2 years (1 day to 15 years), 72% of all patients with fascicular VT were off medications, with no tachycardia recurrence [385].

Two small series reported an acute success rate for catheter ablation for fascicular VT of 100%. However, the overall recurrence rate was 0%–50% over 2–3 years’ follow-up. Major complications included 1 case of complete AV block and 1 case of LBBB [386, 387].

In smaller patients (less than 15 kg) with fascicular reentrant VT that either is controlled medically or is hemodynamically well tolerated without ventricular dysfunction, ablation is generally not pursued [138, 388].

As with many other forms of arrhythmia, infants with this VT frequently experience resolution of the VT with time; such resolution is much less common in older children and adolescents. Idiopathic VAs are often well tolerated, minimally symptomatic, and are not associated with a risk of sudden death [138].

A case series of 2 patients showed that oral verapamil suppressed VTs in newborns. Following discontinuation of verapamil at 1 year of age, both children remained free of tachycardia recurrence at 3 and 4 years of age [393].

4. Among idiopathic VT cases referred for ablation, 2.8% were focal nonreentrant fascicular tachycardia, which had distinct clinical characteristics and usually originated from the left posterior fascicle (LPF), and less commonly from the left anterior fascicle (LAF) and RV Purkinje network. Catheter ablation guided by activation mapping is effective, whereas a pace map–guided approach is less effective. VT recurrence was observed in 27% of patients, and among them, pace mapping was used to target VT during the initial ablation attempt [389].

Another small series reported two focal Purkinje VT cases with IHD in whom complete AV block occurred after the successful ablation of the VT. This outcome suggests that ablation often is not sufficient as the sole therapy due to other induced VTs and conduction abnormalities, requiring pacemaker and/or defibrillator implantation for these VT patients with SHD [105].

5. The Purkinje system might be part of the reentry circuit in patients with postinfarction monomorphic VT, resulting in a type of VT with a relatively narrow QRS complex that mimics fascicular VT. Catheter ablation is effective [138, 392].

### Congenital heart disease

**Table:**
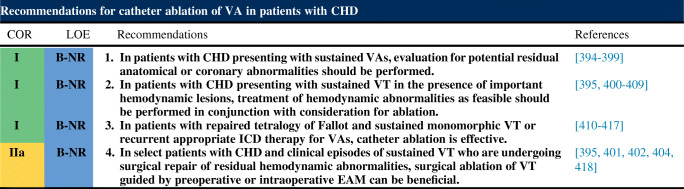

#### Recommendation-specific supportive text

**Fig. 2:**
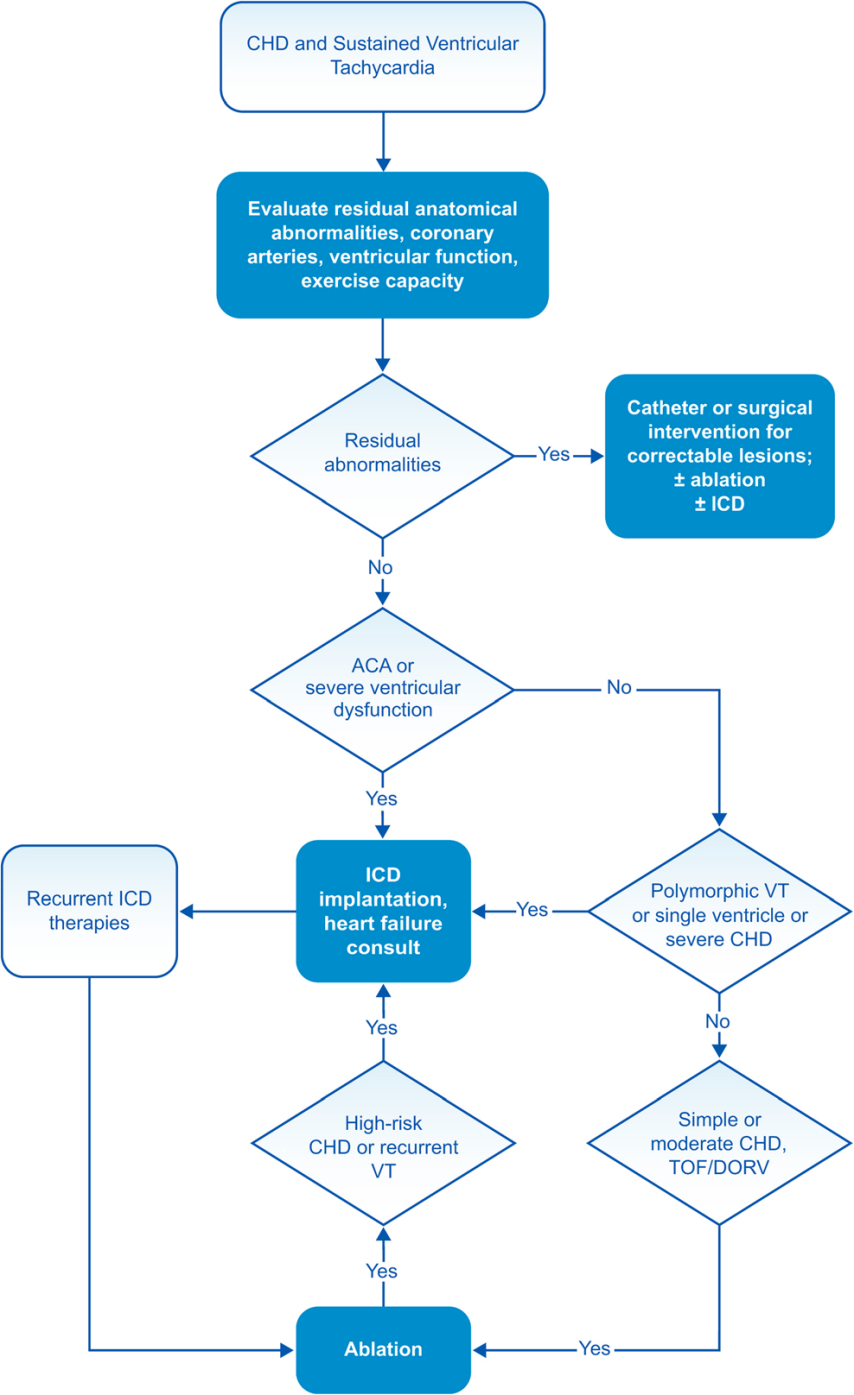
Congenital heart disease and sustained VT. For further discussion of ICD candidacy, please see *PACES/HRS Expert Consensus Statement on the Recognition and Management of Arrhythmias in Adult Congenital Heart Disease* [407] and *2012 ACCF/AHA/HRS Focused Update of the 2008 Guidelines for Device-Based Therapy of Cardiac Rhythm Abnormalities* [419]. ACA = aborted cardiac arrest; CHD = congenital heart disease; DORV = double outlet right ventricle; ICD = implantable cardioverter defibrillator; TOF = tetralogy of Fallot; VT = ventricular tachycardia

1. The substrate for VAs in patients with repaired CHD is typically reentry related to ventricular hypertrophy and areas of scar from prior incisions, patches, and conduit placement. The development of VAs in patients with CHD can be a manifestation of hemodynamic abnormalities, including valvar obstruction or regurgitation, ventricular dysfunction, or coronary abnormalities. Identification of the underlying abnormality contributing to ischemia, ventricular dysfunction, and VAs is important to therapeutic planning, given that interventions for the substrate may reduce the frequency of recurrent VAs and/or improve the hemodynamic status.
2. Most data regarding reoperation for patients with CHD and sustained VAs are from patients with tetralogy of Fallot. Treatment of underlying hemodynamic abnormalities may reduce the incidence of recurrent VAs and allow for ventricular remodeling or improvement in ventricular function. In studies of patients with tetralogy of Fallot and sustained VT undergoing reoperation for hemodynamic abnormalities without specific arrhythmia intervention, late postoperative VT was reduced to 11%–33% during mid-term follow-up [395, 400–402, 406]. Given the incomplete protection from recurrent VA, consideration should also be given to catheter and surgical ablation and/or ICD therapy as indicated (Fig. 2).
3. The majority of reports of successful VT ablation in adults with repaired CHD include patients with tetralogy of Fallot, with small numbers of patients with transposition of the great arteries, ventricular septal defects (VSDs), and other lesions [339, 410, 411, 420]. VT circuits tend to be multiple and related to prior ventriculotomies and VSD patches in patients with complex CHD [412–415, 417, 420, 421]. In patients with tetralogy of Fallot, anatomical isthmuses (AIs) critical to the reentrant circuit have been identified between the septal defect patch or ventriculotomy and the pulmonary valve, and between the tricuspid annulus and the OT patch or septal defect patch [412, 415, 417]. EAM to identify and ablate reentrant channels, with verification of conduction block and occasionally ablation from the left side of the ventricular septum, have procedural success rates of approximately 80% [414, 417, 421]. Patients with procedural success from catheter ablation and with preserved biventricular ventricular function have not experienced recurrent VT or sudden cardiac death in up to 10-year follow-up [412, 415].
4. Several small studies have demonstrated the effectiveness of map-guided surgical ablation of VT during concomitant repair of SHD, with recurrent VT reported in 15%–20% of patients [395, 401, 402, 404]. To date, in patients with tetralogy of Fallot empiric surgical cryoablation lesions for sustained VT without electrophysiological mapping of the tachycardia circuit have shown recurrent VT in 18%–45% of patients [418, 422]. The role of prophylactic empiric cryoablation of the OT in selected high-risk patients with tetralogy of Fallot is evolving [409, 418].

### Inherited arrhythmia syndromes

**Table:**
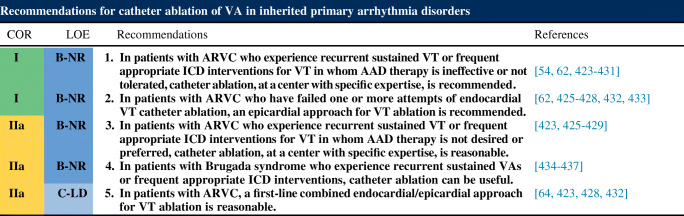

#### Recommendation-specific supportive text

1. In patients with ARVC, recurrent VT or frequent appropriate ICD interventions for VT can be seen despite the use of multiple AAD therapies, including amiodarone and sotalol. Catheter ablation has demonstrated an acceptable acute success rate and over time with development of different techniques (substrate vs. conventional mapping) and approaches (endocardial, epicardial, or combined endo-epicardial) has reduced recurrence rates and appropriate ICD interventions [54, 62, 423–431]. The writing committee felt that earlier consideration could be given to ablation, as opposed to further AAD therapy, in centers with specific expertise in VA ablation in ARVC, including epicardial access. Reference should also be made to the *2019 HRS Expert Consensus Statement on Evaluation, Risk Stratification, and Management of Arrhythmogenic Cardiomyopathy* [438].
2. In patients with ARVC, scar location is heterogeneous based on the chamber (mainly RV dominant but can involve the LV), the number of foci, and depth (epicardial and/or endocardial) [62]. Over time, the need for repeat catheter ablation is common and due to the scar location, and an epicardial approach is often necessary. Long-term freedom from VA is achieved in approximately one-third of patients with an endocardial approach [426]. An endocardial followed by an epicardial ablation approach if the patient remains inducible for VT may avoid unnecessary epicardial access and associated procedural risk. However, strong consideration should be given to performing the procedure in a setting where it is possible to proceed to epicardial ablation during the same procedure, if endocardial ablation fails to terminate the clinical VA, or if the patient remains inducible.
3. In patients with ARVC, recurrent VT or frequent appropriate ICD interventions for VT can be seen despite the use of multiple AAD therapies [439]. The majority of patients undergoing catheter ablation are on antiarrhythmic therapy, including beta blockade before and after ablation. However, some patients in the published series were not on AAD therapy at the time of ablation [423, 425–429]. The overall acute success rates are acceptable, especially when an epicardial approach is used, and indeed are superior to that reported for other substrates like NICM [424]. Patients are often younger and might not be on other medications, which could influence the choice between AAD therapy and ablation. Two studies have assessed whether the substrate underlying VAs in ARVC progressed over time. In the first, scar progression, as assessed by bipolar voltage mapping in patients undergoing repeat ablation, was observed in 2 of 11 patients over a mean follow-up of approximately 6 years [440]. In the second, similar progression occurred in 2 of 7 patients over a 30-month follow-up, and recurrent VTs were ablated within the previously documented scar, suggesting incomplete ablation at the index procedure [441]. This outcome suggests that with a comprehensive initial procedure that achieves noninducibility, substrate progression and recurrent VA are not inevitable, at least over the medium term. Strong consideration should be given to performing such a procedure in a center that can proceed to an epicardial ablation during the same procedure should an endocardial ablation be insufficient to eliminate VT. However, to achieve long-term freedom from AADs, patients might require repeated ablation procedures and rely on ICD interventions for treatment of hemodynamically unstable VT. The writing committee felt that stronger consideration could be given to catheter ablation as a first-line approach without preceding AAD therapy by experienced operators and centers, and with fully informed patients. Reference should also be made to the *2019 HRS Expert Consensus Statement on Evaluation, Risk Stratification, and Management of Arrhythmogenic Cardiomyopathy* [438].

The role of catheter ablation in new-onset single morphology VT without ICD therapy prior to or after ablation was examined only in a small number of patients [439]. Catheter ablation has not been shown to reduce sudden cardiac death in patients with ARVC; thus, ICD therapy remains the mainstay for sudden cardiac death prevention [439, 442–444].

4. Patients with Brugada syndrome and a type I Brugada ECG pattern (spontaneous or drug induced) have an arrhythmogenic substrate located in the anterior epicardial RVOT that can be further unmasked with provocative drug testing [436]. Targeted catheter ablation can normalize the electrographic abnormalities and reduce VA inducibility during programmed stimulation [434–437]. Use of ablation with substrate modification in the most symptomatic patients (recurrent VF/VT, VF storm, or frequent ICD interventions) with Brugada syndrome and a type I Brugada ECG pattern (spontaneous or drug induced) has demonstrated a reduction in arrhythmic events [434–437]. Long-term follow-up has been limited, and generalizability is unknown, given that procedures have been performed at highly specialized centers with expertise in Brugada syndrome and epicardial ablations [434–437]. Drug therapy with quinidine should be considered as a first-line alternative [445].
5. In patients with ARVC, combined endocardial and epicardial substrate catheter ablation has improved acute success and lowered recurrence rates compared with endocardial-only catheter ablation. A combined endocardial-epicardial approach might be a reasonable first-line approach, although not all patients require epicardial catheter ablation for elimination of VT. Risks associated with epicardial access and ablation should be considered, and these procedures should be performed at centers with expertise [59, 60].

### Ventricular arrhythmia in hypertrophic cardiomyopathy

**Table:**
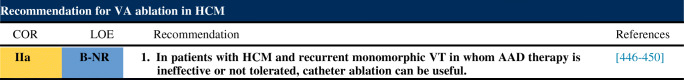

#### Recommendation-specific supportive text

1. Although a less common arrhythmia in HCM, the reported ablation experience is largely limited to monomorphic VT [446–450]. Most patients in these series had already failed AAD therapy. Ablation can be challenging due to the thickness of the myocardium, and epicardial access has been required in the majority of patients [446–449], along with adjunctive techniques such as intracoronary ethanol ablation and surgical epicardial cryoablation [448, 449]. Satisfactory results have been reported, although multiple procedures were required in some patients [446–450]. VAs associated with apical aneurysms are mostly ablated from the LV endocardium.

## Procedural planning

### Patient selection and preprocedural risk assessment

RF catheter ablation is an important therapeutic option in patients with different forms of VA. Over the last decade, significant improvements in the techniques and technologies available for catheter ablation have been paralleled by an increasing number of procedures performed for high-risk and complex patient subsets [359, 451–453]. In these cases, the competing risks associated with the concomitant presence of advanced heart failure syndromes and high burden of associated comorbidities pose substantial periprocedural and postprocedural management challenges. A proper preprocedural risk stratification is crucial to minimize the risk of adverse periprocedural outcomes such as acute hemodynamic decompensation (AHD), which can have devastating consequences [451, 454, 455].

#### The PAAINESD risk score

The PAAINESD risk score (chronic obstructive Pulmonary disease [5 points], Age > 60 years [3 points], General anesthesia [4 points], Ischemic cardiomyopathy [6 points], New York Heart Association class III or IV [6 points], Ejection fraction <25% [3 points], presentation with VT Storm [5 points], Diabetes mellitus [3 points]) has been demonstrated to be helpful to identify patients undergoing scar-related VT ablation who are at increased risk of adverse periprocedural outcomes, and represents the most studied risk stratification tool in this context (Table 4) [451, 452, 454–457]. In a single center, the risk of acute hemodynamic deterioration increased across tertiles of the score, with values ≥17 (or ≥ 15 when “general anesthesia” is excluded) being associated with a risk of 24%. Another single-center report found that, when excluding general anesthesia, a score between 9 and 14 points was associated with a 6% risk of acute hemodynamic collapse, and that risk increased to 24% in patients with a score ≥ 15 [455]. A multicenter cohort found the score (excluding general anesthesia) was higher in those who died early post procedure versus those who survived to the end of follow-up (16 ± 7 vs 9 ± 6; *P* < .001), although chronic obstructive pulmonary disease was not included in the calculation [452]. These findings were mirrored in another single-center cohort, which found a higher score (excluding general anesthesia) in those who died early post ablation (17.4 ± 6.3 vs 11.6 ± 7.6; *P* = .012) and in patients who experienced complications (16.4 ± 6.7 vs 11.6 ± 7.6; *P* < .001) [456]. Of note, intraprocedural mechanical hemodynamic support (HS) was commonly used in two of these series, which could limit the generalizability of these findings [455, 456].

**Table 4:** The PAAINESD Score, developed to predict the risk of periprocedural hemodynamic decompensation

Variable	Points
**P**ulmonary disease (COPD)	5
**A**ge > 60	3
General **a**nesthesia	4
**I**schemic cardiomyopathy	6
**N**YHA class III/IV	6
**E**F <25%	3
VT **s**torm	5
**D**iabetes mellitus	3

The PAAINESD Score, developed to predict the risk of periprocedural hemodynamic decompensation, has values that range from 0 to 35 points (or 0 to 31 [PAINESD] when the modifiable intraprocedural variable “general anesthesia” is excluded) [451]

*COPD* chronic obstructive pulmonary disease; *EF* ejection fraction; *NYHA* New York Heart Association; *VT* ventricular tachycardia

The cumulative evidence arising from published studies suggests that a PAAINESD score ≥ 15–17 (depending on whether the variable “general anesthesia” is included in the risk score calculation or not) identifies patients with SHD and VT at particularly high risk of adverse periprocedural and postprocedural outcomes [451, 452, 455, 457]. Careful consideration of the optimal sedation or anesthesia strategy could further help to prevent AHD in patients with multiple other risk factors.

#### The Seattle Heart Failure Model

Another potentially useful risk stratification tool is the Seattle Heart Failure Model (SHFM), given its established role in predicting mortality in patients with heart failure. The major limitation of the SHFM is that it has been developed from cohorts of patients with heart failure without recurrent VAs, and it remains a complex risk score to calculate due to the number of variables included. Evidence from a single observational study has shown that the SHFM may help to identify patients with scar-related VT undergoing catheter ablation at high risk of midterm (ie, 6 months) postprocedural mortality [458]. Other institutional risk assessment protocols combining variables included in the PAAINESD and SHFM with other risk features, such as hemodynamic status during recurrent VT episodes, have also been proposed to determine which patients are best managed in the setting of dedicated intensive care units or with the aid of HS devices, and whether prolonged postablation surveillance may be helpful [359, 459].

#### Multidisciplinary involvement

The optimal management of high-risk patients extends well beyond the treatment of recurrent VAs and typically involves shared treatment plans and close collaboration between multiple disciplines, including interventional electrophysiologists, cardiologists, heart failure specialists, cardiac anesthesiologists, cardiothoracic surgeons, and pediatric electrophysiologists or those with expertise in CHD (especially in young patients <16 years of age or those with moderate/complex CHD) as appropriate. As such, it is preferable that high-risk patients with recurrent VAs for whom a catheter ablation procedure is planned are managed in centers with proficiency in these procedures, and that they receive prompt access to advanced therapies, including percutaneous HS devices, permanent ventricular assist devices (VADs), and/or heart transplantation. Ongoing heart failure management is a critical component of the management of such patients both immediately after VA ablation and in the longer term.

### 12-Lead electrocardiogram and body surface mapping before ventricular tachycardia ablation

#### Standard 12-lead electrocardiogram

The standard 12-lead ECG has been shown to be a valuable tool in planning catheter ablation procedures for treatment of VAs, including VT and PVC, and in the case of hemodynamically tolerated VAs, every effort should be taken to record the 12-lead ECG. In cases of focal VA in the absence of SHD, the 12-lead ECG is a relatively accurate indicator of the source location, whereas in the presence of myocardial scarring from whatever cause (in which reentry is the predominant mechanism of arrhythmia), the ECG reflects the exit site from the reentrant circuit, rather than the diastolic corridor that typically represents the best ablation target. In some cases, the distance between these two locations can be several centimeters.

#### Ventricular tachycardia and premature ventricular complex in the absence of structural heart disease

Features of the 12-lead ECG during VT or PVC occurring in the absence of SHD have been reported, which suggest endocardial catheter-accessible origins in the RV vs LV outflow regions (including the aortic SV), idiopathic fascicular (verapamil-sensitive) VT related to the LV Purkinje system, and other LV sources, including anterior and posterior papillary muscle VT, para-Hisian VT, mitral annular VT, AMC VT, and GCV/AIV VT; and other RV sources, including tricuspid annular VT, moderator band VT, and papillary muscle VT. Certain ECG patterns can indicate these regions of origin. An LBBB inferior axis morphology with late transition (>lead V3) indicates an origin in the RVOT. Lead I indicates whether the origin is anteriorly (negative) or posteriorly (positive) located [168]. Notching in the inferior leads indicates a lateral (free wall) position in the OT [168]. A Q wave in lead V1, combined with an R wave in lead aVL and an R wave in lead I, together with smaller R waves in the inferior leads, has been found for VAs originating from close to the conduction system [286]. An early transition (≤lead V3) in the presence of an LBBB pattern could indicate a left-sided origin [460], and a broader and taller R wave in V1 or V2 could indicate an origin from the SV [241].

Epicardial origins from the LVOT, in addition to displaying a broad QRS complex with a pseudodelta wave (see below), often have a Q wave in lead I. An epicardial origin close to the AIV, however, can closely resemble the ECG pattern of an origin from the RVOT. An RBBB and inferior axis pattern with positive concordance is the hallmark for VAs originating from the anterior mitral annulus [95]. A transition to a precordial rS pattern, usually in lead V3–V4, has been observed for origins from the papillary muscles with a leftward axis, indicating an origin from the posteromedial, or with a rightward axis, indicating an origin from the anterolateral papillary muscle [461]. Papillary muscle sources often produce a small Q wave in lead V1 [86, 267]. In contradistinction to papillary muscle origins, a fascicular origin has a much narrower QRS complex [461].

**Fig. 3:**
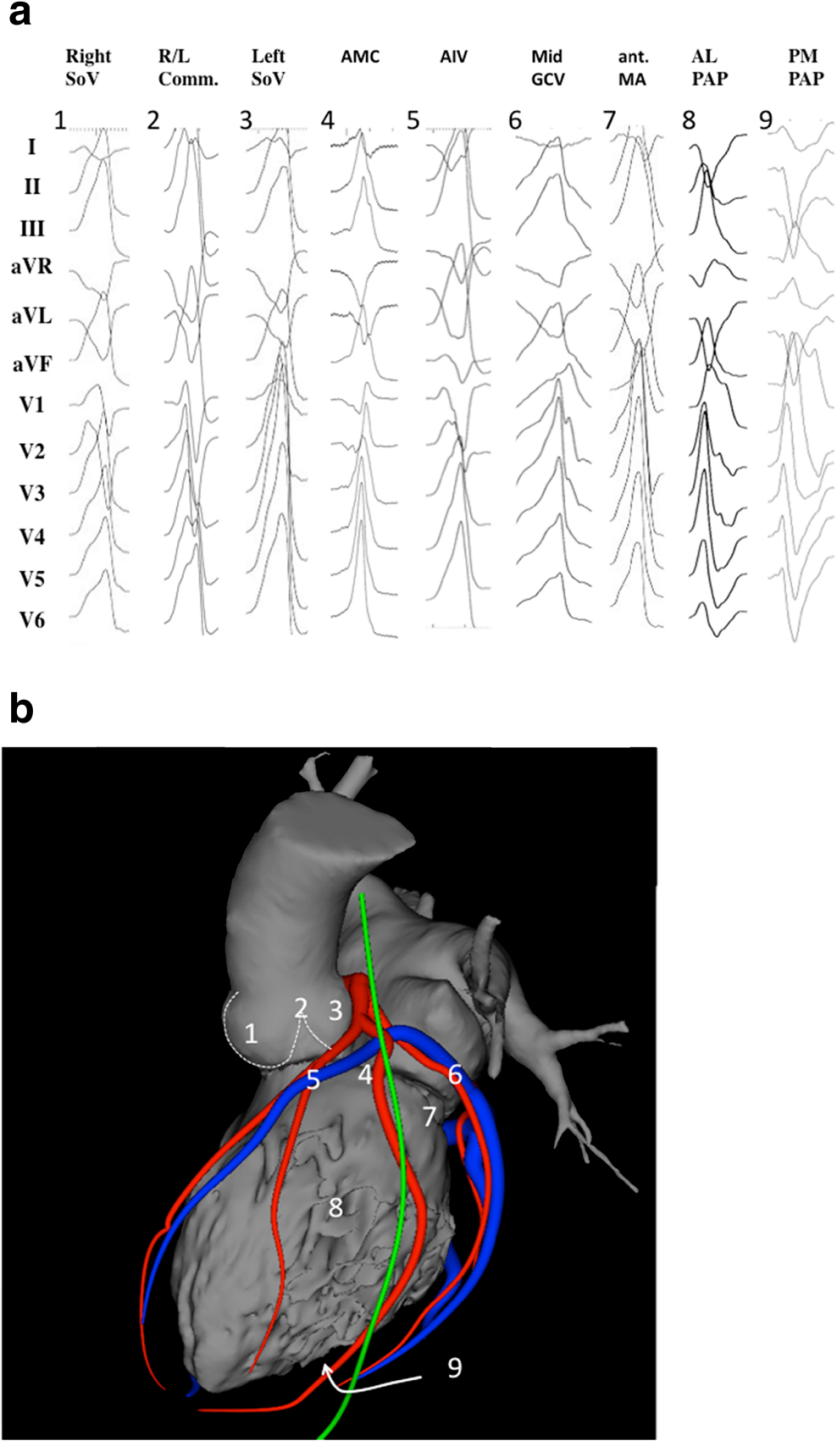
Examples of 12-lead ECGs of premature ventricular complexes from different LV sites, as corroborated by successful focal ablation. (A) shows 12-lead ECG patterns of common ventricular arrhythmia origins in patients without SHD [1–9] from the left ventricle. All leads are displayed at the same amplification and sweep speed. These locations are illustrated in (B) based on 3D reconstruction of a cardiac computed tomography using the MUSIC software that was developed at the University of Bordeaux. The reconstruction shows an anterolateral view of the left ventricle, aorta, and left atrium. Also shown are the coronary arteries (red), the coronary venous system (blue), and the phrenic nerve (green). AIV = anterior interventricular vein; AL PAP = anterolateral papillary muscle; AMC = aortomitral continuity; GCV = great cardiac vein; ant. MA = anterior mitral valve annulus; PM PAP = posteromedial papillary muscle; R/L = right-left; SHD = structural heart disease; SoV = sinus of Valsalva

**Fig. 4:**
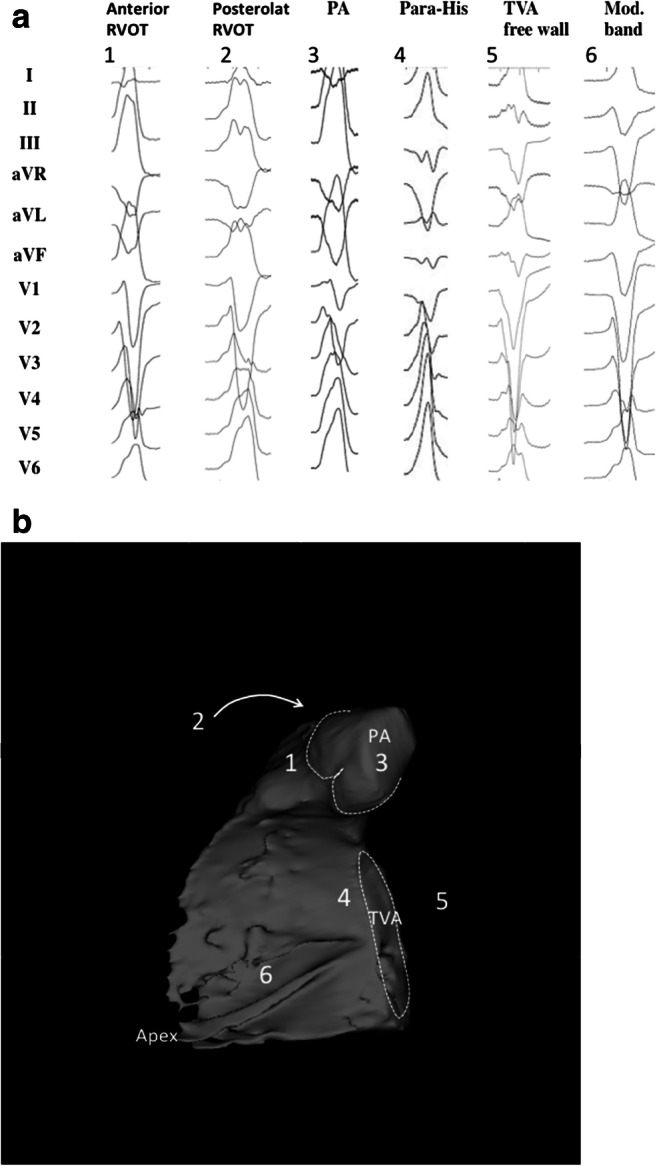
Examples of 12-lead ECGs of premature ventricular complexes from different right ventricular sites, as corroborated by successful focal ablation. All leads are displayed at the same amplification and sweep speed. (A) shows the 12-lead ECG pattern of common origins of right ventricular arrhythmias in patients without SHD [1–6]. The locations are detailed in a 3D reconstruction of the computed tomography using the MUSIC software that was developed at the University of Bordeaux. The reconstruction shown in (B) illustrates the septal view of the right ventricle. Indicated are the pulmonary artery, the tricuspid valve annulus, and the right ventricular apex. ECGs = electrocardiograms; PA = pulmonary artery; RVOT = right ventricular outflow tract; SHD = structural heart disease; TVA = tricuspid valve annulus

Despite the range of heart positions in the chest and body habitus, and variations of ECG lead positions, these ECG features can be remarkably accurate at indicating the SOO to within a 1- to 2-cm radius. Refinement of the exact site of impulse formation using activation or pace mapping is still needed prior to ablation [238, 260, 281, 462, 463]. Examples of PVC morphologies from several common SOOs in the RV and LV are shown in Fig. 3 and Fig. 4.

#### Postinfarction ventricular tachycardia

Several algorithms have been devised to predict the exit site in post-MI VT. As noted above, these all have the important limitation that (to the degree they can reveal localizing information about the arrhythmia) they indicate the exit site of the circuit, not the location of the midportion of the diastolic corridor [24, 464–466]. Although the distance from the exit site to a vulnerable portion of the diastolic corridor can be 1 cm or less, it could be more. In addition, the existing algorithms are not applicable in all cases, and in those cases in which they are, only approximately 75% of VTs can be regionalized to a 2- to 5-cm^2^ area. In general, correlation of exit sites with VT morphology conforms to rather intuitive principles:

1. The majority of post-MI VT exit sites are LV endocardial.
2. LBBB VTs tend to have exit sites on, or within 1 cm of, the intraventricular septum.
3. Patients with inferior infarctions often have Q waves in the inferior leads, indicating inferior wall scarring and an inferior wall exit site, which tend to be located on the inferobasal septum or the inferolateral free wall, with a common diastolic corridor on the inferobasal free wall along the mitral annulus.
4. VTs with a predominant inferior axis tend to have exit sites on the anterior wall (cranial half of the LV).
5. VTs with a predominant superior axis tend to have exit sites on the inferior half of the LV.
6. VTs with a leftward axis tend to have exit sites on or within 1 cm of the septum.
7. VTs with concordant positive precordial QRS complexes tend to have basal exit sites.
8. VTs with concordant negative precordial QRS complexes tend to have apical exit sites.

There can sometimes be disagreement among observers about specific features of ECGs, such as whether an axis is leftward or rightward (ie, discordance between vector of leads I and aVL/lateral precordial leads). Automated algorithms can remove some of the subjectivity of VT ECG analysis [467–469].

#### Epicardial sources

Several ECG criteria have been proposed that indicate an epicardial source of VT in patients without SHD or with NICM. These include several interval measurements that reflect slow, muscle-to-muscle propagation at the beginning of the QRS complex, the maximum deflection index in precordial leads, and presence of Q waves where they would not ordinarily be expected (indicating propagation away from the epicardial surface of the ventricular wall recorded by that chest lead) [174, 176–178]. Of these, the raw interval measurements are subject to false-positive errors in that they can be nonspecifically prolonged by sodium-channel blocking effects of AADs or hyperkalemia as well as by CL (less accurate with faster VTs) [470]. The maximum deflection index (time to earliest peak in any precordial lead divided by the total QRS duration [175], at least in principle, prevents this problem by indexing the intrinsicoid deflection to the total QRS duration, which will be subject to the same effects.

#### Ventricular tachycardia in nonischemic cardiomyopathy

The 12-lead ECG of a VT could be less helpful in NICM to direct ablation; however, target areas can be estimated in a fashion comparable to post-MI VT. In many cases of NICM, successful ablation sites are on the epicardial surface; however, NICM VTs originating from anteroseptal scar typically have an LBBB inferior axis morphology, and these patients (who might also present with AV conduction disturbances due to septal scarring in the region of the bundle branches) might not benefit from an epicardial approach for ablation [471].

#### Bundle branch reentrant ventricular tachycardia

Patients with BBRVT typically have some form of SHD (nonischemic more than ICM), with an LBBB pattern in the baseline conducted rhythm (sinus or atrial fibrillation [AF]), although RBBB or nonspecific interventricular conduction delay can also occur. During BBRVT, the QRS generally closely resembles the baseline QRS, with characteristically rapid initial forces (in contrast to the delayed upstrokes in most other myocardial VTs). This is an important entity to recognize because of its prevalence as well as its curability with a rather simple ablation procedure (targeting RBBs or LBBs).

Many patients with SHD have an ICD in place that terminates the majority of VT episodes with pacing or shock, thus precluding recording of a full 12-lead ECG of the arrhythmia. Only when the device is disabled, or when the VT rate or episode duration is below the device’s programmed detection levels, is it possible to record the ECG during VT. In patients with SHD, having the 12-lead ECG of all spontaneously occurring VTs is especially important during procedures in which multiple morphologies of VT are induced and the operator must decide which morphology (or morphologies) are most important to target for ablation. In the absence of a 12-lead ECG, ICD electrograms of the recorded VT episodes have been particularly helpful to identify the clinical VT when multiple VTs are induced during an ablation procedure [472].

#### Body surface mapping

Body surface mapping has been used for many years as a predictor of VA exit site, starting with skin surface potential minima and more recently as electrocardiographic imaging (ECGI). The latter integrates unipolar electrograms obtained during the arrhythmia while the patient is wearing a 256-electrode vest, with ventricular anatomy derived from a CT or CMR scan with the vest in place. An activation map during the arrhythmia is then mathematically derived using the inverse solution and is plotted on the epicardial surface as designated by the CT or CMR scan. Although experience with this modality in VAs is limited, ECGI maps have shown good correlations with endo- and epicardial mapping results in a variety of settings, in patients with and without SHD [473–476]. Correlations appear to be very good for OT arrhythmias (right vs left) and for those with epicardial sources, and poorer for reentrant VTs (many of which have septal diastolic corridors with variable epicardial breakthrough sites that can be detected by ECGI), and with origin from sites close to scar [477].

#### Summary

In most settings, ECG tracings provide important insights regarding location of either the source of focal arrhythmias or the exit site for reentrant VTs; when possible, a 12-lead ECG should be obtained of the target VA(s) (PVC or VT) and be used as a guide for where to concentrate mapping efforts based on published algorithms. However, no matter how specific the ECG algorithm or ECGI map is, it remains as a guide to where mapping efforts should begin or focus, rather than pinpointing an actual site at which ablation will be certain to eliminate the arrhythmia. Careful mapping is still needed in all situations.

### Facilities for the procedure

#### Facilities

VT ablation can be a complex electrophysiological procedure, especially in patients suffering from deterioration of LV function or from cardiogenic shock. In addition, many patients with scar-related VT have comorbidities that impact on procedural planning. Adequate preprocedural planning and standardization of procedural steps are crucial in this population with advanced heart disease. Preprocedural planning should involve communication with other subspecialties, including cardiothoracic surgeons and physicians with expertise in heart failure management, to optimize patient safety. In general, VT ablation in patients with advanced heart disease should only be performed by experienced operators in centers with expertise for complex electrophysiological procedures with onsite cardiosurgical backup and expertise in HS.

All personnel and equipment should meet institutional requirements and training requirements for treating patients with complex cardiac pathologies. Integrity and functionality of the equipment should be regularly maintained as required by institutional standards.

#### Laboratory equipment

The electrophysiology laboratory for VT ablation should meet standard requirements for any type of catheter ablation procedure, with availability of emergent echocardiography to rule out cardiac tamponade. Laboratory specifications include a dedicated work space and fluoroscopy system (mono- or biplane, capability of cinefluoroscopy or angiography), a programmed stimulator, an electrophysiology recording system with EAM ability, as well as the possibility to perform cardiac catheterization procedures with hemodynamic monitoring capabilities. Institutional and societal requirements for electrophysiology laboratories should be met, and regular maintenance is imperative [478]. The electrophysiology lab should be equipped with a biphasic defibrillator and an ECG monitor, and a backup defibrillator should be immediately accessible. Continuous connection of the defibrillator to the patient (eg, using attached defibrillator pads) is used in many labs for fast and easy defibrillation. A code cart with standard advanced cardiac life support medications is mandatory. For surgical access, such as epicardial surgical windows, institutional hygienic standards should be maintained.

Programming devices for the patient’s specific ICDs should be available in the electrophysiology lab to allow pre- and intraprocedural programming and device interrogation as well as emergency internal shock delivery if required. For patients with implanted left or right VADs, the appropriate equipment and personnel should be available as required.

Epicardial access for mapping and ablation might be needed in a subgroup of patients with VT. Epicardial instrumentation is a complex part of VT ablation and requires high expertise and experience with epicardial puncture or surgical access. Continuous availability of an ICD programmer and the capability of delivering internal shocks can be crucial to terminate VAs in case of external defibrillation failure due to pericardial fluid and/or air and in cases of suboptimal defibrillator pad location.

Patient monitoring is crucial for safety during ablation of VT, independent of the underlying cardiac disease. A physiological recorder capable of monitoring pulse oximetry, noninvasive and invasive arterial blood pressure, and ECG is recommended. Vital signs in addition to oxygen saturation should be displayed for the operator and assisting staff throughout the procedure. For longer procedures involving patients with SHD, invasive intra-arterial blood pressure monitoring might be superior to noninvasive noncontinuous measurement. Noninvasive monitoring of cerebral tissue oxygen saturation has been used to reflect end organ perfusion when longer episodes of VT were required, to allow for adequate mapping [479, 480].

#### Personnel

Credentialing of personnel involved in VT ablation procedures is specific to institutions and can vary by country. The personnel in the electrophysiology lab typically consist of a nurse and a technician involved in patient care and monitoring; possibly a person handling the ICD programmer (approximately 15% of the writing committee have a representative from the device company assisting with intraprocedural management of the ICD) during the ablation; an expert operator for manipulation and placement of catheters; and one or two operators managing the electrophysiology recording system, the stimulator, and the mapping system. When a 3D EAM system is used, as is the case in most VA ablation procedures, a highly trained professional (eg, a manufacturer’s representative, a technician, or a physician) is usually present to operate this system and assist with acquisition, editing, and interpretation of data. With respect to sedation, different requirements exist in different countries and societies. An anesthesiologist, a nurse anesthetist, or an equivalent person experienced with sedation and intubation should be present if deeper sedation is required. In this regard, sedation during VT ablation by trained cardiologists has been shown to be safe [481–483].

Personnel involved in VT ablations should be trained for complex electrophysiology maneuvers and mapping maneuvers for monitoring severely impaired patients under sedation and administration of antiarrhythmic as well as inotropic drugs [481–483]. Recognizing any potential complication or prolonged hemodynamic compromise is a key feature to patient safety. Personnel involved in VT ablation should be proficient in using cardiac defibrillators and in performing advanced life support and cardiopulmonary resuscitation.

Operators performing VT ablation procedures should be certified interventional electrophysiologists with expertise for RV and LV mapping and ablation. Coronary angiography by the operator him- or herself or by another physician might be needed during epicardial or aortic SV ablation procedures to delineate anatomy of the coronary arteries. In addition, physicians capable of inserting percutaneous HS devices should be available in case of prolonged hemodynamic compromise. A cardiac surgeon should be available in case cardiopulmonary bypass, surgical HS, or sternotomy is required.

Epicardial VT procedures can be more complex than endocardial VT ablation procedures; these are discussed in more detail in Section 6.3. For adequate treatment of possible complications and also for surgical epicardial access, cardiac surgical backup is warranted.

#### Patient safety

Due to procedural complexity and risks, catheter ablation for VAs should have appropriate patient safety and pertinent laboratory protocols in place. Electrophysiology laboratory equipment should undergo routine maintenance by a biomedical engineering department to ensure appropriate functionality and should be available for troubleshooting. In particular, routine assessment for external defibrillator functionality should be performed according to regulatory, manufacturer, and institutional policies. Radiation exposure should be minimized to the operator and patient with the use of digital-pulsed, low pulse rate fluoroscopy if feasible, and implementation of ALARA (as low as reasonably achievable) principles. Safety protocols should be in place for management of emergent complications, with a focus on those pertaining to higher-risk VA ablation (eg, epicardial access with surgical backup, hemodynamically unstable VAs requiring HS, acute coronary injury requiring coronary intervention).

### Preprocedural imaging

**Table:**
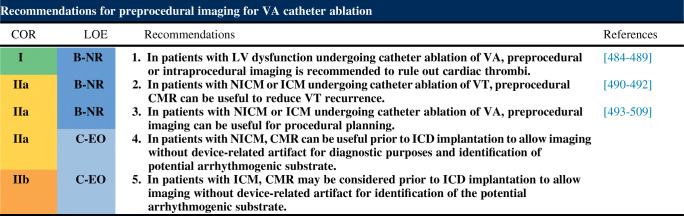

#### Recommendation-specific supportive text

1. In patients with LV dysfunction, recent preprocedural imaging should be performed if LV catheter manipulation is anticipated to assess for LV thrombi to prevent thromboembolic events. Typically, a transthoracic echocardiogram is performed to assess for intracardiac thrombi [510, 511], and contrast echocardiography has further been shown to improve the yield of echocardiography to detect cardiac thrombi, especially in high-risk patients [487, 512] and in patients with poor echocardiographic windows. CMR has been shown to be superior to transthoracic echocardiography for the detection of intracardiac thrombi [488, 489, 513], especially in the presence of small and mural thrombi. To further minimize the possibility of thromboembolic events, for patients with concomitant AF, it might be prudent to also obtain a preprocedural transesophageal echocardiogram to rule out left atrial thrombi if defibrillation or cardioversion might be required during the ablation procedure [510]. An intracardiac echocardiography (ICE) can alternatively be performed prior to LV catheter manipulation if no recent imaging is available to rule out LV thrombi.
2. Preprocedural imaging with CMR and use of CMR for targeting the arrhythmogenic substrate has resulted in reduced VT recurrence and reduced mortality in a few observational studies [490–492]. Patients with contraindications to CMR were excluded, and in one study, only patients with good image quality benefited from the preprocedural CMR [492]. CIEDs have long been considered as being contraindicated for magnetic resonance imaging (MRI). However, provided that appropriate precautions are taken, MRIs in patients with CIEDs have been demonstrated to be safe in multiple studies and registries [514–516], and a recent HRS expert consensus statement details specific recommendations for MRIs in patients with CIEDs [517].
3. By demonstrating location [495, 508, 509] and extent [509] of delayed enhancement, CMR helps to identify the location of the arrhythmogenic substrate and therefore is beneficial in periprocedural planning regarding the need for obtaining epicardial access. Other imaging modalities, such as CT and nuclear imaging, have been correlated with electroanatomical voltage mapping data to identify scarring by detecting areas of delayed enhancement [493–500, 502], wall thinning [504], hypoperfusion [503], and lack of metabolic activity [507]. Contrary to CMR, CT and nuclear imaging do not provide specific information about the precise extent of scarring within the myocardial wall; these imaging techniques might be preferable for preprocedural imaging in case of contraindications to CMR.
4. Prior to implantation of a cardioverter defibrillator, performing a CMR might be beneficial in patients with NICM and ICM. Especially for patients with NICM, the writing committee felt that in addition to localizing scar in the event of future VT development, a CMR will be beneficial for diagnostic reasons to clarify the etiology of NICM. In the *2013 ACC/AHA Guideline for Management of Heart Failure*, CMR is considered a class IIa recommendation to assess for infiltrative disorders [518]. In general, it was felt that the higher the odds that patients might have recurrent VTs, the stronger the indication for a CMR prior to ICD implantation (for example, in patients with a secondary prevention indication for ICD implantation). Concerns about cost implications were the main reason why the writing committee issued a class IIb recommendation for patients with ICM.

#### Synopsis

Although randomized studies that demonstrate that the use of imaging results in improved procedural outcomes are lacking, pre-, intra- and postprocedural imaging is routinely used for VA ablation procedures.

Advances in imaging technology, image integration, and image analysis have paved the way for CMR and CT to take the lead as imaging modalities to define the presence and extent of SHD as well as to refine the characteristics of the arrhythmogenic substrate.

Due to the high spatial resolution of ex vivo MRI, myocardial scarring can be characterized with an almost histological precision [519, 520]. Therefore, LGE-CMR is often referred to as the gold standard for scar assessment. Unfortunately, a clinical CMR study obtained for a patient has a much lower spatial resolution due to the motion of the beating heart compared with an ex vivo MRI. Several studies have reported on the accuracy of CMR-defined scar by correlating CMR-defined scar with areas of low voltage from EAM data [493–500]. Although differences exist between these studies, there is general agreement between imaging defined scar and the abnormal substrate defined by EAM. Important limitations of the EAM-defined substrate, however, need to be kept in mind, and there is mounting evidence for improved procedural outcomes with an imaging-guided ablation compared with an EAM-guided ablation approach [490–492]. EAM-derived data reflect the 3D surface of the myocardium and lack information about the 3D anatomical and pathological integrity of the heart. Hence, CMR offers a more comprehensive assessment of the entire heart, especially of the intramural and epicardial myocardium, which is difficult, if not impossible, to assess with endocardial voltage mapping alone. Lack of adequate catheter contact with the myocardium can further impact on accuracy of EAM. These limitations can be overcome by integrating the CMR data into the EAM. Accurate registration of the CMR with the electroanatomical map is of critical importance to have reliable information about location and extent of myocardial scarring. The information about scar location has been supplemented by further characterization of the tissue heterogeneity within the scar that contains the surviving myofiber bundles as the arrhythmogenic substrate [521–523].

Two methods have been described to characterize tissue heterogeneity: the SD method, which uses the remote, unenhanced myocardium as a reference, defining scar if the signal intensity is above normal myocardium (usually >2–3 SD) [494, 495, 524]; and the signal intensity method (full-width at half maximum technique), which uses >50% of the maximal signal intensity within a region of interest to define scar. Various cutoff values have been used to distinguish the border zone from the scar core and have been correlated with outcomes [521–523]. Controversy exists, however, about which of the methods and which of the cutoff values is preferable for assessment of tissue heterogeneity. It is important to note that quantification of the border zone still awaits histologic validation. Limitations of LGE-CMR include the limited spatial resolution that is in the 1- to 2-mm range, making it difficult to characterize tissue heterogeneity if the wall thickness is in this range. This, among others, is one of the reasons why the assessment for LGE in the thinner RV is challenging when using standard LGE-CMR techniques. In the past, CIEDs have been considered to be contraindications for CMR. This is no longer the case, and provided that certain precautions are followed, CMR has been shown to be safe in several studies [516] and registries [514, 515]. However, in the presence of a CIED, artifacts from the device generator, especially from ICDs, can obscure the myocardium and render a CMR study completely or partially nondiagnostic [525]. It is therefore recommended to perform CMRs prior to ICD implantation. The prevalence and extent of artifact can be minimized by wideband CMR sequences [526]. CMR in patients with NICM has been particularly helpful despite the presence of ICDs, and improved outcomes have been described in patients who underwent CMR prior to VT ablation procedures [490–492].

The advantage of multidetector cardiac CT (MDCT) over LGE-CMR is the higher spatial resolution that is in the submillimeter range. Various MDCT characteristics indicative of scarring have been described, including a degree of wall thinning with a cutoff wall thickness of <5 mm [505], hypoattenuation [503], and delayed enhancement [502]. More recently, thanks to the higher spatial resolution of MDCT, thicker ridges of tissue separating areas of thinning have been described within the myocardial scar harboring the majority of VT target sites in patients post infarction [501]. Hence, MDCT imaging has been found to be beneficial as an alternative to CMR to indicate the location of myocardial scarring, especially in the presence of contraindications for CMR. Although assessment of wall thickness in MDCT imaging has been used successfully in patients with prior infarctions, the ability to identify scarring based on wall thickness alone has been less successful [504, 527] in patients with NICM, and might require a different approach [502]. MDCT is the imaging technique of choice for preprocedural imaging of the coronary arteries, the coronary veins, and the phrenic nerve. The ability to image the coronary arteries in conjunction with the ability to image epicardial fat thickness has been especially valuable for epicardial ablation procedures to enhance safety and to quantify epicardial fat thickness covering potential epicardial VT target sites [528, 529].

Nuclear imaging for the purpose of facilitating VT ablation procedures has been described for PET and single-photon emission computerized tomography (SPECT) scanning, using CT for image integration. Although there is a correlation between PET/CT defined scar and EAM [506, 507], the correlation of SPECT perfusion imaging with low-voltage areas on EAM is lower [530]. It is intriguing that metabolically active areas corresponding to critical VT sites have been identified; however, they might not correspond to areas of low voltage on the EAM [506]. Furthermore, sympathetic denervation can be imaged using tracers, such as 11C-meta-hydroxyepinephrine and iodine-123-metaiodobenzylguanidine. Regional sympathetic denervation has been recognized as an imaging marker for an increased risk of sudden cardiac death [531]. Viable but denervated myocardium has been shown to be particularly sensitive to development of arrhythmias [532, 533]. Klein et al. [534] have demonstrated that critical VT ablation sites were located in denervated areas, some of which were located in areas with preserved bipolar voltage.

Finally, echocardiography is typically used to rule out the presence of cardiac thrombi [510, 511] prior to an ablation in patients with SHD in whom catheter manipulation in the LV is anticipated. Although transthoracic echocardiography has been beneficial in identifying intracardiac thrombi [484–486], the use of contrast has been shown to further increase the yield of cardiac thrombi [487, 512] and should be used if the LV endocardium is not well visualized. Although ablation procedures might be safe in the presence of laminated thrombi [535], it would be prudent in the absence of an urgent indication for VT ablation, and especially in the presence of a mobile thrombus, to anticoagulate the patient for a period of time and reassess for LV thrombus prior to the ablation procedure. Not every patient has optimal echocardiographic windows that allow for comprehensive assessment of the entire endocardium to rule out LV thrombi, even if sonographic contrast is used. LGE-CMR and Cine-CMR are alternative imaging techniques that have a higher accuracy in identifying myocardial thrombi [488, 489, 513], which should be kept in mind, particularly in patients for whom transthoracic echocardiography is considered insufficient to rule out an LV thrombus. The sensitivity of ICE for detection of LV thrombus has not been systematically studied, but it can provide real-time assessment immediately before catheter placement. To minimize the risk of thromboembolic events for patients with concomitant AF, a preprocedural transesophageal echocardiogram will be helpful to exclude a left atrial thrombus if a cardioversion is required during the ablation procedure [510].

### Patient preparation

Heart failure, electrolyte abnormalities, and myocardial ischemia should be adequately treated and controlled, if possible, before the patient is subjected to an invasive electrophysiology study. Similarly, associated SHDs should be clearly defined, given that patients with conditions such as severe LV systolic dysfunction, severe coronary artery disease, critical aortic stenosis, and advanced renal impairment might not tolerate induction of arrhythmia, prolonged procedures, or fluid overload resulting from the use of saline-irrigated catheters. Although some of these conditions can be optimized prior to an elective procedure, this is often not possible for more emergent procedures. In these cases, a substrate-based ablation approach without repeated VT inductions might be preferable.

AADs, with the exception of amiodarone, should, if at all possible, be discontinued for at least 5 half-lives prior to the ablation procedure. This is almost mandatory for cases in which the primary strategy of mapping is not substrate-based, but it might not be possible for emergency cases. Oral anticoagulants are generally discontinued prior to the ablation to achieve an international normalized ratio < 1.5 at the time of the study. Direct oral anticoagulants should be withheld for 24–48 h before the procedure, depending on anticipated access, and bridging with heparin can be used for patients with mechanical heart valves or other features that place them at high risk of thrombosis. The majority of the writing committee (60%) usually use bridging in patients with mechanical valves, where possible, but many distinguish between mechanical aortic valves, in which transseptal access with continued anticoagulation can be used, and mechanical mitral valves, in which retrograde access is used. For elective procedures, imaging with ultrasound can help to define the feasibility of an arterial closure device, and therefore continued anticoagulation. Routine perioperative bridging in patients with AF who interrupted warfarin for procedures, including percutaneous transvascular procedures, was noninferior to no bridging in a large RCT with respect to the occurrence of thromboembolic events [536]. Approximately half of the writing committee do not use bridging in similar patients, and a further 20% do not use bridging in patients taking direct oral anticoagulants.

Informed consent needs to be obtained from the patient or his or her surrogate after discussing the risks and benefits involved in the procedure, as well as alternatives. The patient should fast overnight or for at least for 6 h prior to the procedure [537, 538].

## Intraprocedural patient care

### Anesthesia

**Table:**
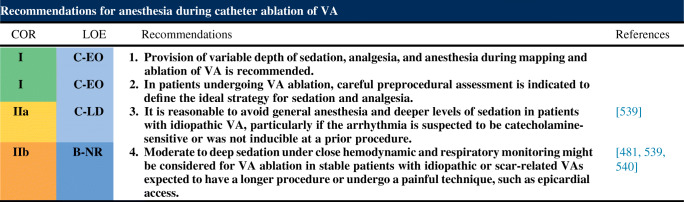

#### Recommendation-specific supportive text

1. The American Society of Anesthesiologists defines levels of sedation in a continuum, varying from minimal sedation (anxiolysis), to moderate sedation or analgesia (conscious sedation), to deep sedation or analgesia, to general anesthesia [541]. Careful preprocedural assessment is indicated to define the ideal strategy for sedation and analgesia based on age and comorbidities, targeted arrhythmia, planned procedure, risk for airway obstruction, and patient wishes. General anesthesia ensures patient comfort that facilitates vascular and especially epicardial access, and also ensures immobility during catheter manipulation for mapping and ablation, especially for procedures of long duration. However, a major disadvantage of general anesthesia is its potential suppression of VA. Elimination of mental stress and the related changes in the autonomic tone during general anesthesia or deep sedation can potentially decrease the spontaneous manifestation of catecholamine-sensitive VA and the induction/maintenance of reentrant VT [542–545]. Inhaled anesthetics that prolong action-potential duration and ventricular refractoriness (sevoflurane and isoflurane) and intravenous anesthetics that reduce sympathetic tone (dexmedetomidine) are commonly avoided for ablation procedures [542, 543]. Additionally, most of the anesthetic agents used for sedation and analgesia reduce myocardial contractility and systemic vascular resistance, causing hypotension that could be exacerbated during VT, requiring pharmacological or mechanical HS. Those cardiovascular effects are more intense at the anesthetic doses needed for general anesthesia, which additionally produce higher attenuation of sympathetic tone. General anesthesia or the use of cardiodepressive medications, such as propofol for anesthesia in patients with severely compromised LV function, can result in acute hemodynamic compromise during the ablation procedure, and its use needs to be carefully considered for these patients. If general anesthesia is used during epicardial ablation procedures, the concomitant use of muscle relaxants could preclude identification of the phrenic nerve during epicardial ablation. Use of a short-acting muscle relaxant during induction of anesthesia is acceptable, without redosing; however, it could necessitate a deeper level of anesthesia with resultant exaggeration of the above-mentioned hypotensive effects. Nevertheless, the effects of propofol on cardiac electrophysiological proprieties are diverse and could include arrhythmia suppression. In fact, propofol has been associated with suppression of VT and VT storm [544].

The need for sedation and analgesia varies throughout the procedure, being higher during vascular and epicardial access and cardioversion or defibrillation. On the other hand, a more superficial level of sedation (or any) is desirable to prevent VA suppression and to assure VT induction and maintenance for mapping. Short-acting agents are considered more suitable to provide those rapid changes in the requirements of sedation level during mapping and ablation of VAs [546]. Intravenous boli of midazolam and fentanyl at repeated doses as needed was reported by a single-center observational study as a feasible and safe strategy for minimal sedation in patients with idiopathic PVC or VT planned for shorter procedures [539]. However, even shorter-acting sedatives such as midazolam have the potential for suppressing idiopathic VA for prolonged periods of time and need to be used with caution during procedures targeting idiopathic VAs, such as frequent PVCs. A strategy to use shorter-acting sedatives such as propofol for allowing temporary deeper sedation is frequently used initially, and if VAs are suppressed, sedation can be reduced or discontinued to allow for arrhythmias to reappear as necessary. For patients with scar-related VTs, sedatives such as midazolam, which has the additional benefit of amnesia, do not impact on inducibility of VT and can be used in conjunction with analgesics and/or other sedatives such as propofol to achieve the desired level of sedation. Continuous infusion of propofol, alone or complemented with repeated boli of fentanyl (as needed), has been proposed for deep sedation of stable patients during ablation of idiopathic or scar-related VT that are expected to have a longer duration or that require more painful and challenging techniques, such as epicardial access. Two single-center observational studies reported that this strategy can be safely performed in this subset of patients under close hemodynamic monitoring [481, 539]. Propofol had to be discontinued (switched to midazolam) in 11.7% of the procedures, predominantly due to hypotension presenting in elderly patients and prolonged procedural duration [481]. Respiratory depression resulting in sustained oxygen saturation of <90% requiring reduction of propofol and transitory mechanical maneuvers to assist ventilation was present in 1.5% of the patients in the same study.

2. The selection of sedation strategy for patients undergoing ablation of VAs requires a balance between the desire of assuring a comfortable and safe procedure and the need to induce the targeted arrhythmia [546]. Proactive communication between the operator and the anesthesia team and with the patient about the patient’s needs and desires, as well as the electrophysiological requirements, helps to select a sedation strategy that is in the best interest of the patient.
3. In the prospective arm of a large observational study, in which patients with sustained, monomorphic VT and SHD underwent noninvasive programmed stimulation (NIPS) under minimal sedation, followed by invasive programmed stimulation under general anesthesia using propofol or isoflurane, >90% remained inducible under general anesthesia, although in half, a different VT was induced [547]. General anesthesia was associated, however, with less hemodynamic stability and greater use of vasopressor support with phenylephrine, without an adverse effect on procedural outcomes. More aggressive stimulation was often required with general anesthesia than with conscious sedation [547]. In another cohort of 25 patients referred for epicardial ablation in the setting of NICM, a strategy of initial programmed stimulation under sedation with midazolam, fentanyl, or remifentanil led to induction of tolerated VT in 15 patients, and avoidance of epicardial access (which was performed under general anesthesia) altogether occurred in 10 (40%) [540].

On the basis of the cited single-center observational studies [481, 539, 540, 542–548] about the various sedative and analgesic strategies for ablation of VAs, it is reasonable to avoid general anesthesia and deeper levels of sedation in patients with idiopathic VAs (PVC or VT) planned for shorter procedures, particularly when the arrhythmia is suspected to be catecholamine-sensitive (typically, automatic and triggered arrhythmias) or was not inducible during a prior procedure. In these patients, a minimal sedation strategy with short-acting sedatives and analgesics, with repeated doses as needed, can be useful to ensure adequate sedation without VA suppression.

4. Epicardial scar-related VT mapping and ablation under deep sedation with sufentanil or remifentanil and intermittent boli of midazolam was reported as a feasible and safe strategy in a single-center prospective series and a case report [546, 548]. Interestingly, no respiratory failure resulting in endotracheal intubation or prolonged periods of hypotension requiring HS were observed (except during periods of unstable VT) in these cases (combined 79 procedures in 73 patients). Importantly, this strategy allowed epicardial access with comfort, preventing the need for general anesthesia and muscle relaxants (facilitating the identification of the phrenic nerve during epicardial ablation), and providing rapid recuperation of consciousness with no recall of punctures or cardioversions. Remifentanil is an opioid with an analgosedative effect that can have advantages for ablation of VAs, including a lack of negative inotropic effect, comparatively little effect on arrhythmogenicity, time-to-peak effect of approximately 90 s and a half-life of 3–4 min [546]. This agent is also associated with hypotension likely secondary to vasodilatation and bradycardia.

Moderate to deep sedation with continuous infusion of propofol and repeated boli of fentanyl under close hemodynamic and respiratory monitoring might be considered for stable patients with ablation of idiopathic or scar-related VA with no indication for general anesthesia who are expected to undergo a procedure of longer duration or for patients who require more painful techniques, such as epicardial access. Another short-action anesthetic, such as remifentanil or sufentanil, could also be considered. Of note, patients with severe hemodynamic compromise, severe life-threatening comorbidities or acute illness, respiratory failure, high risk for airway obstruction, or who were intubated before the procedure for arrhythmia storm or cardiogenic shock were excluded in the cited studies and are also traditionally considered candidates for general anesthesia. Hospital and regulatory guidance varies widely on the training and specialization required for different levels of sedation and administration of various drugs (see Section 11).

### Vascular access

**Table:**
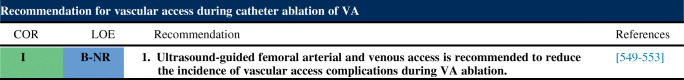

#### Recommendation-specific supportive text

1. Significant vascular complications occur in approximately 2% of VT ablation procedures [554], which are further detailed in Section 10. Ultrasound-guided femoral arterial and venous access has been widely implemented in electrophysiological procedures in an effort to reduce vascular complications. Several observational studies of electrophysiological procedures, mostly examining AF ablation, one RCT, and a meta-analysis have shown an association of ultrasound guidance with a reduction in major and minor vascular access complications and bleeding [549–552]. One observational study reported the rate of major complications in the subgroup undergoing VA ablation, which was 8.9% in the conventional group and 0% in the ultrasound-guided group [549]. In a single RCT in patients undergoing AF ablation, ultrasound guidance reduced time, additional punctures, arterial puncture, and unsuccessful access. Although it increased first pass success, however, it was underpowered for the primary endpoint of major vascular access complications due to a lower than expected observed complication rate [551]. Similarly, a meta-analysis of nonrandomized controlled trials of ultrasound- vs fluoroscopy- or palpation-guided femoral arterial access found it was associated with a reduction in the number of attempts and vascular complications [552]. An RCT of ultrasound- vs fluoroscopic-guided retrograde femoral arterial access in patients undergoing angiography or interventional procedures found that ultrasound guidance reduced the number of attempts, time to access, risk of venipuncture, and vascular complications [553].

#### Synopsis

For VA ablation, standard percutaneous vascular access techniques are used. The choice of peripheral vascular access depends upon several factors, including the arrhythmia’s SOO, the location of any substrate identified on pre- or intraprocedural imaging, the patient’s anatomy, and the presence of peripheral vascular disease or venous occlusion or agenesis. LV access can be achieved through either retrograde transaortic or antegrade transseptal approaches, depending on patient specifics and operator preference, the latter especially in the presence of mechanical aortic valve replacement or severe aortic or peripheral vascular disease. Although most areas of the endocardial LV can be accessed by either approach, the degree of contact force can vary (eg, contact force was significantly higher transseptally in the mid-anteroseptum, mid-lateral, and apical segments, and significantly higher with a retrograde approach in the basal-anteroseptum, basal-inferoseptum, basal-inferior, and the basal-lateral segments) [555]. If a retrograde access to the LV endocardium has been chosen, the use of long sheaths can be helpful in the presence of tortuosity of the iliac arteries or the distal aorta. Once vascular access has been obtained, heparin can be administered to prevent clotting of the sheaths.

### Epicardial access

**Table:**
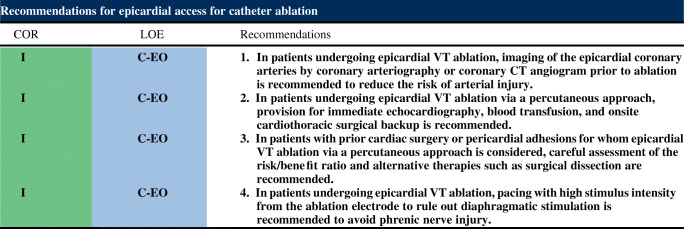

#### Recommendation-specific supportive text

1. Inadvertent injury to an epicardial coronary artery can occur by puncture or laceration with a needle and is recognized by aspiration of arterial blood from the pericardial space. Arterial injury can require arterial stenting or surgical repair. In addition, application of RF current within 5 mm of an epicardial coronary artery can result in stenosis, spasm, or occlusion of the vessel [556–558]. Because of the risk of arterial injury, imaging of the coronary arteries with arteriography is essential. The use of other coronary artery imaging techniques assumes that an adequate registration of imaging data has been accomplished. Hence, coronary angiography is the preferred technique.
2. Epicardial ablation requires considerable preparation prior to the procedure. Major complications occur in approximately 5% of epicardial ablation procedures [60]. Unintended puncture of the RV can occur in up to 17% of cases [60], although bleeding is usually self-limited provided that a sheath has not been introduced through the RV free wall. The patient’s blood should be typed and cross-matched for immediate transfusion should significant bleeding occur. Because bleeding within the pericardial space is common, intracardiac or transthoracic echocardiography should be immediately available. In addition, cardiothoracic surgical backup should be readily available. A potentially serious complication of percutaneous subxiphoid transpericardial puncture is laceration of the liver or intra-abdominal arteries. Thus, unexplained hypotension should prompt imaging of these structures to evaluate possible intra-abdominal bleeding that could necessitate surgical repair.
3. The presence of pericardial adhesions can severely restrict the ability to maneuver a guidewire or catheter within the pericardial space. Thus, patients with prior cardiac surgery or a history of pericarditis can present significant challenges for epicardial ablation. Although dissection of pericardial adhesions using a guidewire, deflectable catheter, or deflectable sheath can be useful to allow epicardial mapping [559–561], access to the entire epicardial surface might not be possible, and loculated pericardial effusions that are challenging to drain percutaneously can occur in this setting. A planned surgical dissection of pericardial adhesions can allow dense adhesions to be safely lysed and allow epicardial ablation in selected cases [562, 563].
4. Damage to the phrenic nerves can occur if the ablation electrode lies adjacent to these structures [564] and can be minimized by pacing from the ablation electrode using high stimulus intensity to identify phrenic nerve stimulation. If phrenic nerve stimulation is observed during pacing, mechanical means to protect the phrenic nerve might be required, such as inflation of an intrapericardial balloon [565].

#### Background

VA can originate from the endocardium, the midmyocardium, or might involve the epicardium. The latter is particularly true for patients with NICMs, including idiopathic DCM [61, 65, 495], ARVC [62, 64, 566–568], myocarditis [569], sarcoidosis [347, 570], and ChD [58, 571–573], as well as Brugada syndrome [435, 574–576]. Also, for patients with VT following MI, the arrhythmogenic substrate can be located in the midmyocardium or in the subepicardial region [35, 320, 577–580]. Finally, idiopathic VA can originate from the LV epicardium [87, 175, 275, 279]. The percutaneous technique for epicardial mapping and ablation in patients with Chagas cardiomyopathy (CCM) was described by Sosa et al. in 1996 [58]. Since that initial description, epicardial ablation has become an important technique to effectively treat VA in a variety of diseases. Despite mapping data implicating an epicardial origin of postinfarction VT in up to one-third of cases [577, 578, 581], epicardial ablation procedures are typically performed in only a small minority of patients post infarction. This is most likely due to the predominant endocardial involvement of postinfarction scarring and the three-dimensionality of reentry circuits that can include the endocardium. The anatomical distribution of post-MI epicardial VTs is highly associated with infarctions in the distribution of the left circumflex or right coronary arteries [582]. The prevalence of epicardial VT in patients with NICMs appears to be significantly higher than for VT following MI [59, 61, 349]. The site of scarring and slowed conduction supporting VT in NICM is most often located on the epicardial surface of the basal lateral LV [59, 349]. Thus, epicardial ablation is an important technique to address VT in a variety of SHDs, particularly in those with NICM.

#### Criteria suggesting epicardial substrate

Several factors suggest an epicardial substrate for VT, including the anatomical location of scar on CMR or CT imaging [495, 583, 584], unipolar voltage mapping from the endocardium [65], and the lack of identifiable scarring or regions of slow conduction with endocardial mapping. The presence of a subepicardial or midmyocardial scar with contrast-enhanced CT or CMR is a valuable tool to identify potential epicardial substrates for VT and correlates with voltage mapping [583, 584]. Preprocedural imaging is therefore useful to select patients for epicardial mapping and ablation and to guide the operator to the regions of interest. The surface ECG [174, 176–178, 585] during VT provides important clues to an epicardial origin, including the presence of a pseudo-delta wave (≥34 ms in duration) in the precordial leads, an intrinsicoid deflection to the peak of the R wave in lead V2 ≥ 85 ms, and an RS duration of ≥121 ms, although with limited specificity in patients with ICM [174, 585]. An ECG algorithm to identify epicardial VT in patients with NICM includes the absence of Q waves in the inferior leads with either pseudo-delta waves (≥75 ms), a maximum deflection index ≥0.59, or a Q wave in lead I [177]. The reported sensitivity and specificity of these criteria to predict successful epicardial ablation in NICM can exceed 90% [177]. ICE has also been shown to be useful for identifying epicardial scar as VT substrate with increased echogenicity strongly correlating with the results of EAM [586]. Although the origin of epicardial VTs in patients with SHD depends on scar location, in patients without SHD, the SOO is often in close proximity to the mitral annulus and the LV summit. This area can be reached and mapped via the coronary venous system. Scar-related VTs are often beyond the reach of the coronary venous system and hence require different access to the pericardial space.

#### Epicardial access technique

Access to the epicardium is most often achieved with a subxiphoid, transpericardial puncture using either a 17–18-gauge, 6-in. Tuohy or similar needle with a beveled tip [58], a thinner 21-gauge needle [587], or a needle-in-needle technique [587, 588]. Three-quarters of the writing committee who perform epicardial access use a Tuohy needle, whereas the rest use the needle-in-needle technique. The latter approach uses a 7-cm, 18-gauge needle for support while puncturing the skin and subcutaneous tissues, while a longer (15–20-cm) 21-gauge micropuncture needle is inserted through the 18-gauge needle to puncture the parietal pericardium [588]. With either approach, small amounts of radiographic contrast are injected to confirm that the needle is within the pericardial space and that contrast moves freely within this space. A long guidewire is then advanced through the needle, with care taken to ensure that it moves freely to surround the cardiac silhouette. The angle of entry into the pericardial space can be either anterior, in which case the guidewire travels superiorly over the free wall of the RV, or posterior (inferior approach), in which the guidewire travels beneath the LV inferior wall before traveling superiorly toward the posterobasal LV. Transpericardial access should be guided by fluoroscopy, including a steep left anterior oblique or lateral view; the latter being useful for an anterior access. Once successful entry within the pericardial space is achieved, a sheath is advanced over the guidewire, allowing the mapping and ablation catheter to be moved across the epicardial surface. Percutaneous subxiphoid access to the pericardial space might not be feasible in patients with dense pericardial adhesions, including patients with prior cardiac surgery, previous pericarditis, or prior epicardial ablation procedures [559–561]. For these patients, a limited thoracotomy with a small subxiphoid or larger left lateral thoracotomy might be required that allows for manual lysis of adhesions and control of bleeding [562, 563]. Thus, a collaborative approach with a cardiothoracic surgeon and an electrophysiologist might be required for patients in whom a percutaneous approach is not feasible because of pericardial adhesions. The presence of less extensive pericardial adhesions can be approached percutaneously, using a deflectable catheter and sheath for adhesion lysis. Insufflation of carbon dioxide or contrast via intentional exit into the pericardial space from the right atrium [589] or coronary venous system [590] could have a role in determining the extent of adhesions in patients with prior cardiac surgery [589] and can facilitate epicardial access; however, further study is required.

Catheter ablation on the epicardial surface is usually performed with irrigated RF current, though cryoablation has also been reported [591–595]. Use of a low irrigation flow rate (5–7 mL/min) appears to result in a similar lesion size as the higher flows used in endocardial ablation while limiting intrapericardial fluid accumulation, which reduces lesion size [592]. Frequent aspiration of irrigant is also important to prevent hemodynamic compromise. The use of contact force-sensing catheters can improve orientation of the ablation electrode so that current is directed toward the epicardial surface rather than toward the parietal pericardium [596–598]. Epicardial fat >5 mm in thickness results in reduced electrogram voltage and increased stimulation threshold, and could limit the depth of RF lesions [528, 581, 599–603]. Epicardial fat is typically clustered along the course of epicardial coronary arteries, within the AV grooves, and along the free wall of the RV.

#### Epicardial access complications

The complications of epicardial ablation are important to consider. Major complications have been reported in approximately 5% of patients [59, 60]. The most common complication of epicardial mapping and ablation is pericarditis, which can occur in over 20% of patients [59]. The use of systemic or intrapericardial steroids can decrease the risk and severity of pericarditis (see Section 10.1). Inadvertent puncture of the RV can occur in up to 17% of cases and usually results in self-limited bleeding of <80 cc venous blood [60]. Avoidance of dual antiplatelet therapy can reduce periprocedural bleeding during epicardial ablation. Inadvertent puncture of the RV can be recognized if a guidewire can be advanced into the pulmonary artery. This is managed by withdrawing the guidewire from the RV, withdrawing the needle, and readvancing the wire until the pericardial space is accessed. If the needle enters and then exits the RV before pericardial access is obtained, a “through and through” puncture of the RV can result. In this case, hemodynamic collapse might occur only on removal of the sheath at the end of the procedure. This complication can be detected when bleeding occurs after withdrawal of the pericardial sheath with the guide wire still in place. It is recommended to advance a guide wire into the pericardial space prior to sheath removal and to observe the puncture site for bleeding before removing the guide wire. Other complications of percutaneous, subxiphoid pericardial access include laceration or puncture of an epicardial coronary artery or vein (which could necessitate coronary stenting or surgical repair), RV pseudoaneurysm, and coronary spasm [604]. RF current that is applied within 5 mm of an epicardial coronary artery can produce occlusion, spasm, or stenosis of the artery [556–558]. Because of this risk, imaging of the coronary arteries with arteriography or preprocedural CT angiography is essential to accurately localize the ablation electrode relative to the position of the coronary arteries. The left phrenic nerve has a variable course and can be damaged by RF or cryoablation energy [564, 565]. Preprocedural imaging (see Section 5) has been particularly helpful to display the anatomical course of the coronary arteries and the phrenic nerve, thereby preventing damage to these structures. Reliance on imaging only, however, might cause damage to these structures in case of imperfect image registration. Also, pacing from the ablation electrode with high stimulus intensity prior to ablation should be performed to exclude phrenic nerve stimulation. In some cases in which the ablation target is in close proximity to the phrenic nerve, a balloon or steerable catheter can be advanced into the pericardial space to shield the nerve from ablation-related injury [564, 605]; the use of air and fluid in the pericardial space has also been reported to reduce the risk of phrenic nerve damage [565]. Of note, the presence of air in the pericardial space can increase the defibrillation threshold, requiring emergent decompression or internal defibrillation if defibrillation is required [606].

Esophageal injury can also occur if ablation is performed in the posterior LV [607–609]. Although the exact mechanism and risk factors are unclear, esophageal temperature monitoring might be warranted before ablating near the esophagus. A significant complication of subxiphoid transpericardial access is bleeding within the abdomen from laceration of the liver or an intra-abdominal artery [604]. Unexplained hypotension during or after epicardial ablation should lead to prompt investigation of possible intra-abdominal bleeding, which could require surgical repair.

Because of the many important complications that can occur with epicardial ablation, extensive experience with this procedure is required. Careful preprocedural planning is essential, including immediate access to echocardiography, imaging of the coronary arteries, provision for immediate blood transfusion of cross-matched blood, and backup cardiothoracic and general surgical support.

### Intraprocedural hemodynamic support

**Table:**
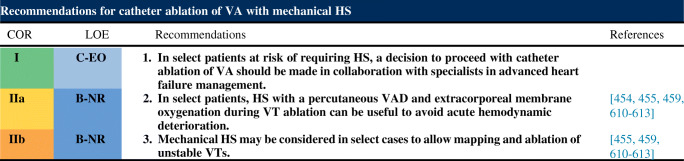

#### Recommendation-specific supportive text

1. In patients with VAs and severe comorbidities, incessant or recurrent VTs treated by multiple ICD shocks might cause end organ hypoperfusion and further deterioration in cardiac function [359, 451]. Planning for a VA ablation procedure, in which there is a significant risk that it will be complicated by hemodynamic deterioration, should involve specialists in heart failure. The patient’s values regarding the various temporary and permanent HS options and their feasibility should be considered.
2. Data about strategies for HS during VT ablation are available from single and multicenter, nonrandomized, retrospective, and observational trials, which have demonstrated feasibility and safety [455, 459, 610–613]. Mapping of nontolerated VTs can be performed for longer periods of time when HS with either percutaneous left ventricular assist device (pLVAD) (Tandem Heart, CardiacAssist, Pittsburgh, PA; and Impella, Abiomed, Danvers, MA) or extracorporeal membrane oxygenation is performed as opposed to no support or intra-aortic balloon pump (IABP) [610, 611]. A large retrospective multicenter report, however, has demonstrated that the use of HS was associated with higher acute ablation failure rates, increased periprocedural complications, higher mortality, and a higher rate of VT recurrence [613]. It is possible that HS was used in patients with more severe disease in this study, and outcome data were similar between the HS and no HS groups compared with patients with severely compromised EF (≤20%) and advanced heart failure (NYHA class III and IV). Hence, the use of HS in these patients needs to be carefully considered. Of note, data supporting HS as a bailout strategy during or right after VT ablation are poor and do not demonstrate a benefit for procedural outcomes and survival [454, 455, 459].
3. The benefit of HS to allow mapping of unstable VTs needs to be weighed against the potential detrimental effects of HS, depending on the type of support that is used, including vascular damage due to large-bore venous and arterial access sites, among others [610]. Prevention of hemodynamic deterioration during the ablation procedure by cautious use of cardiodepressive medications such as propofol could obviate the necessity for HS; also, the use of general anesthesia has been identified as a factor associated with hemodynamic deterioration and needs to be carefully considered in patients with severely impaired myocardial function. Adequate patient selection using risk assessment algorithms (see Section 5) could help to identify patients for whom HS might be beneficial.

### Intraprocedural anticoagulation

**Table:**
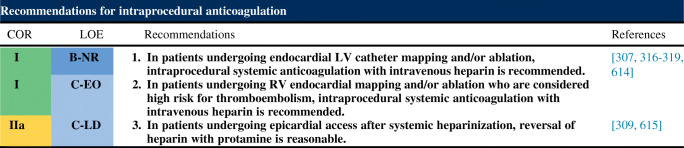

#### Recommendation-specific supportive text

1. Catheter ablation of VA can be associated with risk of thrombus formation and thromboembolism. Although the individual risk varies with type and site of ablation and patient factors, the risk of clinically apparent stroke or thromboembolism in patients with SHD undergoing VT ablation in RCTs has ranged from 0% to 1.9% [307, 316–319] and is lower in patients without SHD [616]. A small study with diffusion-weighted magnetic resonance brain imaging demonstrated the presence of a new brain lesion in 58% of patients undergoing LV endocardial ablation [617]. These lesions were detected in the absence of clinically apparent stroke; no brain lesion was identified in patients undergoing RV ablation. Although systemic heparinization has been routine for LV endocardial ablation since the earliest large-scale investigations, few studies have compared different approaches to anticoagulation before, during, or after the VT ablation procedure [614, 615] (Table 9). Systemic anticoagulation with heparin is recommended for all procedures that last more than several minutes involving left heart catheterization. Intraprocedural anticoagulation schemes differ between centers. Unfractionated heparin is commonly administered after sheath insertion as an initial bolus (empirical dose 5000–10,000 IU or 50–100 IU/kg) followed by intermittent boli and/or continuous infusion to maintain a target activated clotting time (ACT) longer than 250–350 s [307, 309, 614, 615, 617, 618]. In a survey regarding intraprocedural anticoagulation among the writing committee members, for idiopathic VA, 48% of the responders use ACT levels longer than 250 s, 39% longer than 300 s, and 13% longer than 350 s. For patients with SHD, 25% of the committee members use an ACT target of 250 s, 58% use a target longer than 300 s, and 17% use a target longer than 350 s. The ACT level should be checked at 15-min intervals until therapeutic anticoagulation is achieved, and then at 15- to 30-min intervals for the duration of the procedure. Approximately 90% of the surveyed committee members routinely check for ACT during VT ablation procedures. Most members use either a Hemochron ACT (Instrumentation Laboratory, Bedford, MA) or an i-STAT (Abbott Point of Care Inc., Princeton, NJ) device for ACT monitoring. For transseptal access, heparin should be administered prior to or immediately following the transseptal puncture. More than 70% of the writing committee members surveyed routinely administer heparin before the transseptal puncture, and approximately 20% use a higher target ACT when a transseptal approach is used. Among the writing committee members, 85% use continuous flushing for sheaths, especially if a transseptal approach is used. Heparin infusion can be discontinued once all catheters are removed from left sided chambers. In the event of significant persistent bleeding or cardiac tamponade, protamine should be administered to reverse heparin. In patients with a history of heparin-induced thrombocytopenia or allergy, a direct thrombin inhibitor, such as bivalirudin or argatroban, can be considered as an alternative for intraprocedural anticoagulation; however, limited experience exists in patients undergoing VA ablation [618].
2. During RV endocardial mapping, systemic anticoagulation with heparin is not necessary unless other factors are present that increase the thromboembolic risk. The use of heparin can, however, prevent deep venous thrombosis or pulmonary embolism, especially when a prolonged procedure with multiple venous catheters and extensive ablation is anticipated. Similarly, patients with a history of deep venous thrombosis, pulmonary embolism, hypercoagulable state (eg, factor V Leiden), right-to-left cardiac shunt, severe RV dilatation, and advanced heart failure should undergo systemic anticoagulation. Among the writing committee members, 65% reported routine use of heparin for VT ablation procedures even in the absence of risk factors for thromboembolism.
3. Anticoagulation is not required solely for epicardial mapping and/or ablation. If LV mapping is planned, epicardial access can be obtained prior to LV instrumentation and systemic anticoagulation (see Section 6.3). When epicardial access is required after therapeutic heparinization, reversal of heparin anticoagulation with protamine is typically performed [51, 619]. Two small studies reported epicardial access in fully anticoagulated patients without a major increase in risk of bleeding complications [620, 621]. When the writing committee was surveyed about epicardial access after full heparinization, half of the writing committee would administer protamine for heparin reversal even when extensive endocardial ablation had been performed, 20% would administer protamine only after limited LV endocardial ablation, and another 20% of the writing committee members would terminate the procedure and schedule an epicardial ablation as a separate procedure.

### Antibiotic prophylaxis

Prophylactic antibiotics are not generally indicated for sterile procedures such as VA ablation [622]. However, patients with ICDs undergoing VA ablation, in which catheters might be in direct contact with the intravascular leads, could present a special circumstance. No data exist to support this practice, nor is a specific antibiotic preferred over another. Approximately 40% of the writing committee members administer prophylactic antibiotics to patients with a pacemaker or ICD undergoing VA ablation, whereas a quarter never do, and the remaining do in select patients based on potential risk factors for device infection, such as a prosthetic valve or use of a Foley catheter. Thirty percent of the writing committee use antibiotic prophylaxis for patients undergoing epicardial access.

### Fluid balance

Careful monitoring of fluid balance is essential in patients undergoing VA ablation, especially given that many have impaired ventricular function, putting them at risk of volume overload, and they might have renal impairment. Various irrigated catheters are currently in use, allowing for different flow rates, and hence, different volume loads. A urinary catheter might not be required for cases performed under sedation when the anticipated procedure duration is short, but for longer ablation procedures, it should be considered. This permits more precise assessment of the fluid balance, which becomes particularly important for patients in whom irrigated-tip RF catheters are used. However, urinary catheters can cause urinary tract infection and bleeding; thus, a sterile technique is essential during insertion and after-care. After measurement of volume infused and eliminated (“ins and outs”), an intravenous loop diuretic should be considered, especially with a large infused volume, a significantly positive procedural fluid balance, and impaired LV function.

## Electrophysiological testing

PES is a key component of the VT ablation procedure, allowing for mapping of induced arrhythmias. In addition, a baseline assessment of arrhythmia induction can be useful by comparison for the eventual assessment of procedural outcome (see Section 9.17). Given the majority of idiopathic VAs are caused by cAMP-mediated delayed afterdepolarizations, they can be provoked with catecholamine infusion and burst pacing [623, 624]. Although purely substrate-based ablation (without arrhythmia induction) can be performed, many laboratories perform programmed stimulation in select patients with reentrant scar-related VT for several reasons: 1) to perform activation and entrainment mapping in tolerated VT; 2) to identify the clinical VT morphologies to better focus substrate-based ablation; and 3) to limit the extent of ablation delivery. Although “legacy” protocols for programmed stimulation have been proposed [625, 626], most laboratories that perform programmed stimulation would hold to a minimal standard of at least 1 site and 2 drive CLs, with the delivery of 1–4 ventricular extrastimuli at coupling intervals limited by local refractoriness or a minimum of 180–200 ms. Given known site dependence for induction of reentrant VT, additional sites of stimulation (particularly the LV) can be useful. The use of long–short sequences during programmed stimulation has been particularly helpful to induce BBRVT that can easily be addressed with an ablation procedure.

The most important objective of the ablation procedure is to identify and eliminate the clinical VT. Prior studies have demonstrated that programmed stimulation results in VT induction in 93%–95% of patients with healed MI and a history of sustained VT [626, 627]. Importantly, these studies were concerned with induction of any VT, but not necessarily the clinical VTs. Limited data are available to address the question of how reliable PES is in reproducing all clinically pertinent VT morphologies. Given that 12-lead ECGs of the clinical VT are frequently not available, ICD electrograms have been described to reliably identify the clinical VT when induced in the electrophysiology lab [628]. Unfortunately, the clinical VT is not always inducible: 7%–24% of patients are completely noninducible [629–631], and the clinical VT cannot be induced in another 13%–30% of patients [628, 629, 632]. Furthermore, induction of VTs that have not been previously documented occurs frequently, depending on whether the PES protocol is completed, until all extrastimuli are delivered from multiple sites and refractoriness has been reached. Elimination of all inducible VTs has been found to result in improved outcomes [633, 634]; hence, this is the ideal objective of the ablation procedure. Inducibility of nonclinical VTs post ablation has been found to be associated with a high VT recurrence rate [635]. Risks and benefits will need to be weighed, however, given it might take performance of longer procedures to reach this objective. A major concern about the reliability of PES is that VTs often recur even when patients have been rendered completely noninducible post ablation [634]. Edema formation in a critical area can render a targeted VT temporarily noninducible; however, incomplete lesions can also form a new or modified substrate, generating new VTs. The former can be addressed with NIPS a few days after the ablation procedure, which has been found to predict VT recurrence in patients who were noninducible at the conclusion of the ablation procedure [636]. PES is typically performed at the onset of the procedure, but it might need to be deferred to the conclusion of the procedure if there is concern about the patient’s hemodynamic status.

Despite there being less complete characterization of the effectiveness of PES to guide ablation of VT in nonischemic substrates, noninducibility has also been demonstrated to result in superior outcomes post ablation [637]. Only limited data and anecdotal reports exist on the use of other agents (aminophylline, epinephrine, calcium, dobutamine, caffeine) to induce idiopathic arrhythmias when isoproterenol and burst pacing fail [638].

A survey of the writing committee is summarized here to give some perspective. For induction of VT in SHD, all the members routinely stimulate from the RV apex, 66% from the RVOT and 59% from the LV if LV access has already been obtained. Up to 3 extrastimuli are routinely performed in 76%, and up to 4 in 24%. For induction of idiopathic arrhythmias, the majority of the committee uses isoproterenol frequently (93%), followed by epinephrine (21%), phenylephrine (14%), atropine (11%), and calcium (7%). In addition, some members of the committee at least considered dobutamine and caffeine.

At this point, PES remains a key tool of the mapping and ablation procedure. The use of PES, however, often needs to be complemented by alternative strategies, given its many limitations.

## Mapping and imaging techniques

### Mapping catheters

#### Multielectrode mapping

The use of multielectrode mapping catheters has gained popularity over the past several years with the introduction of mapping systems capable of acquiring data from multiple sites for each beat. These catheters offer several advantages, particularly in respect to mapping density, resolution, and speed. The ability to record electrograms from multiple sites at each beat increases the number of data points and can shorten the duration of mapping.

Multielectrode catheters often use small electrodes with short interelectrode spacing, thereby increasing mapping resolution, which can be advantageous for substrate mapping [639–641], facilitating identification of surviving myocardial bundles within heterogeneous scar tissue that may escape detection when mapping with standard ablation catheters with larger electrodes and spacing is performed [641–643]. Multielectrode catheters allow pacing from multiple electrodes positioned in and around areas of scar. The increased current density during pacing from smaller electrodes can achieve capture at relatively low pacing stimulus strength [639]. In addition, pacing from one site while recording from surrounding sites allows investigation of propagation in multiple directions that can identify anisotropy and areas prone to slow conduction and/or block [644, 645]. These catheters can also be useful for activation mapping during VT, given they allow rapid acquisition of multiple sites at high spatial resolution that can facilitate identification of a reentry isthmus or VT focus [643].

Currently, several multielectrode catheters are available. The Pentaray catheter (Biosense Webster, Diamond Bar, CA) is shaped like a flower and has 5 splines, each with 4 electrodes (a total of 20 electrodes), which can be used with the CARTO EAM system. This catheter frequently causes ectopic beats in areas of contracting myocardium, and spatial sampling is nonuniform due to variable spread of the splines and limited contact with the myocardium. A linear catheter with 20 electrodes is also available for use with this mapping system. This catheter has uniform spatial spread of electrodes and a relatively large area of contact, although during endocardial mapping the proximal electrodes are often not in contact. It is well suited for mapping of the epicardial surface. A duodecapolar catheter with tighter interelectrode spacing (Livewire, Abbott Laboratories, Abbott Park, IL) has been used to map endocardial and epicardial ventricular surfaces in vivo and has been validated against histology in an animal model [646, 647]. The Advisor HD Grid catheter (Abbott Laboratories, Abbott Park, IL) is a novel 4-by-4 unipolar electrode array with 1-mm (diameter) electrodes, equidistantly spaced 3 mm apart from each other, which can be used in conjunction with the EnSite Precision system [648]. A potential advantage is the uniform spatial sampling; however, collection of ventricular mapping data in humans in vivo is currently not available. The fourth catheter is a small basket catheter with 64 very small electrodes arranged on 8 splines that can be used with the Rhythmia EAM system (Orion, Boston Scientific, Marlborough, MA) [643, 649]. This catheter has a low noise level, facilitating recording of low amplitude signals. However, the basket is not well suited for mapping the papillary muscles, the RV, or the epicardium.

Common limitations of multielectrode mapping catheters include mechanical trauma with frequent ectopy, transient injury of the superficial conduction bundles, limited maneuverability, lack of tissue contact information, and the potential for thrombus formation with the need for careful anticoagulation. In addition, the potential benefit of mapping with smaller electrodes in patients with NICM and high prevalence of intramyocardial substrate is unclear. Currently, ablation is performed with a second catheter, necessitating integration of the anatomy and physiology acquired with the multielectrode catheter in a mapping system that can also support data acquisition with an ablation catheter. Newer catheter designs integrating very small electrodes positioned at the circumference of a standard ablation catheter could allow combining high-resolution mapping with ablation on a single catheter [640].

### Activation mapping

Mapping of the electrical activation sequence during VT is a valuable mapping strategy for patients with hemodynamically stable monomorphic tachycardia [650]. It can localize the origin for focal tachycardias and potentially reentry circuits, depending on their anatomy [644].

Activation mapping is performed by recording of local electrograms from multiple sites during VT and is facilitated by the use of 3D mapping systems, allowing display of the position and relative timing on the EAM system. For focal VTs, the earliest site of activation identifies the SOO and is the target of ablation. At this site, the local bipolar electrogram precedes the QRS onset, and the unipolar signal (with high-pass filter setting <1 Hz) demonstrates a QS configuration, consistent with a centrifugal spread of activation away from the SOO. In intramural focal tachycardias, the bipolar electrogram often inscribes with or after the onset of the QRS and the unipolar electrogram demonstrates an rS configuration, consistent with an initial activation propagating toward the recording electrode. In a true intramyocardial origin, mapping from all opposing surfaces demonstrate relatively late bipolar activations with rS unipolar configurations.

The most common VT mechanism in patients with SHD is scar-related reentry with continuous excitation of the circuit throughout the tachycardia CL [650]. The QRS onset typically occurs when the impulse reaches the exit from the scar to activate the contractile myocardium [651]. In “exit sites,” electrograms can be fractionated and immediately precede the onset of the QRS complex. Ablation at exit sites can terminate the tachycardia; however, it can also result in a change of the tachycardia configuration and/or CL in which the diastolic pathway exits at different locations from the scar [643]. Ablation of the diastolic pathway “isthmus” is therefore a more desirable target, given it can eliminate the machinery required for reentry. Electrograms at isthmus sites occur earlier during diastole, are typically of very low-voltage amplitude (<0.5 mV), and can have multiple potentials. Mapping of these circuits with multielectrode mapping catheters can be helpful for identifying low amplitude signals, for differentiating near-field from far-field potentials, and for shortening the time required for mapping (see Section 8.1). It should be noted that the mere presence of diastolic potentials does not suggest an isthmus location. These can also be recorded in dead-end pathways and adjacent bystanders unrelated to the circuit. It is therefore important to carefully review and interpret the activation map in order to understand the location and complexity of the circuit. In principle, an activation map of a macroreentrant VT circuit should demonstrate distinct entrance, isthmus, and exit sites that all serve as obligatory parts of the circuit, such that it cannot continue without all of these elements. However, scar-related circuits (particularly in patients with a nonischemic substrate) can have intramural component(s) that might not be recorded on the surface. These usually exhibit a “gap” in the activation sequence, such that part of the circuit is “concealed” from the surface map, residing deep in the myocardium or on the opposing surface.

Activation mapping of scar-related VTs can be highly valuable for identifying the isthmus. However, it is often limited to patients with hemodynamically stable VT. In patients with less tolerated VTs, limited activation mapping can be performed in conjunction with substrate, entrainment, and/or pace mapping. The utility of HS to allow extended mapping during VT can improve the acute procedural end-point of noninducibility; however, it has limited impact on the long-term outcome (see Section 6.4) and has not reduced VT recurrence [610, 613].

### Entrainment mapping

#### Entrainment mapping: Overview

**Fig. 5:**
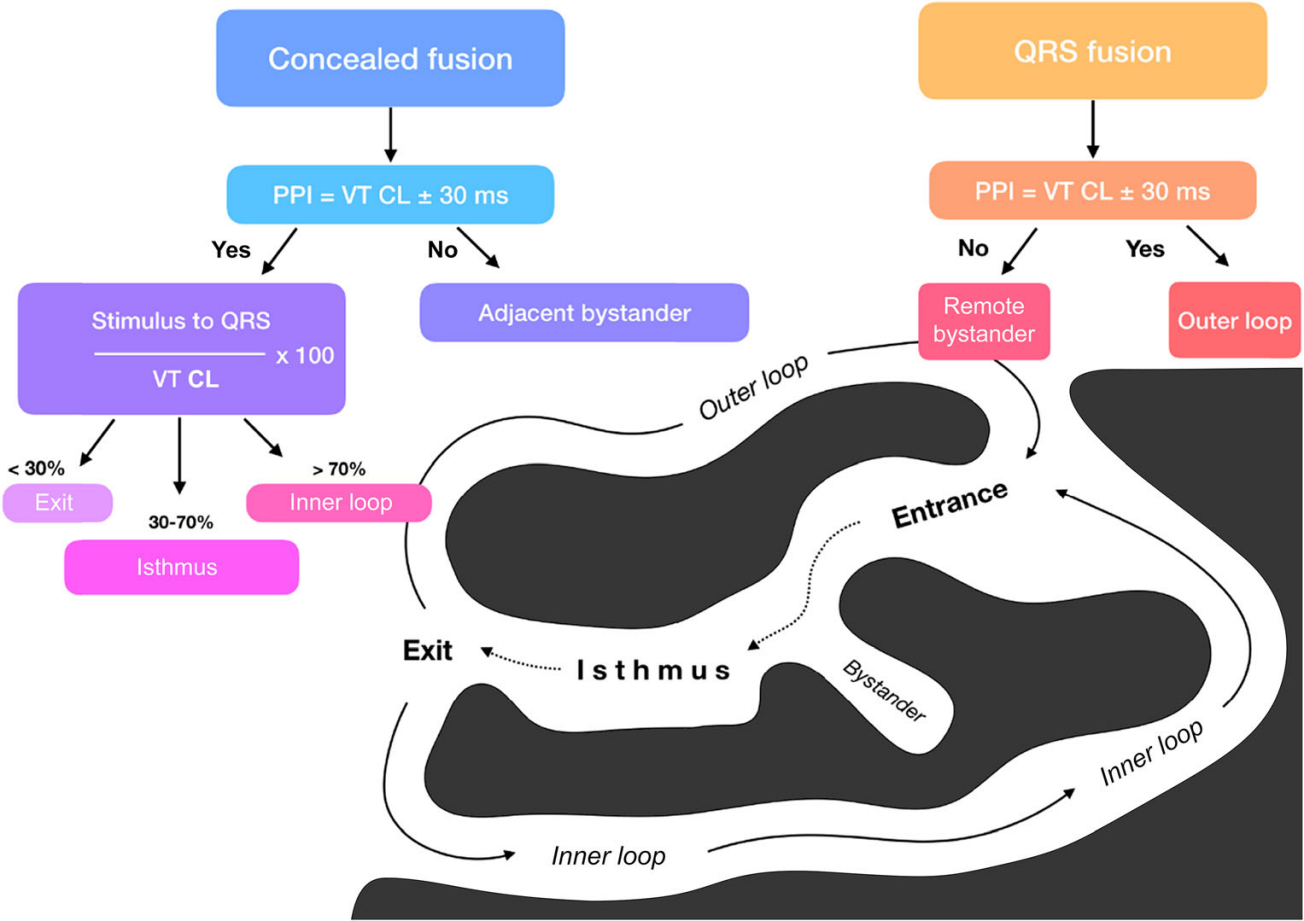
Entrainment responses from components of reentrant VT circuit. CL = cycle length; PPI = postpacing interval; VT = ventricular tachycardia. Adapted with permission from Elsevier [31]

**Fig. 6:**
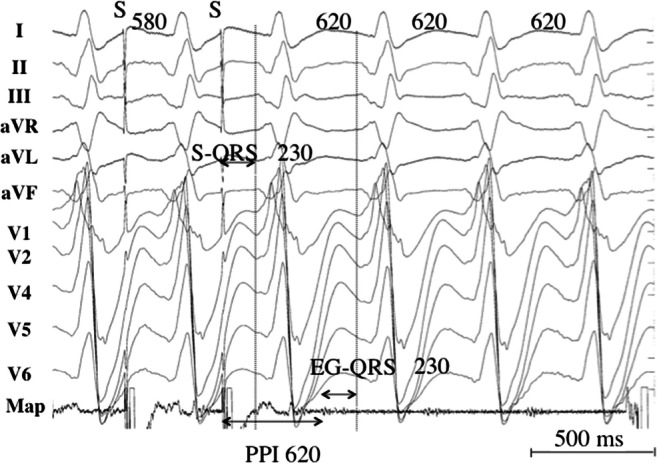
Pacing from the protected isthmus of a VT circuit. Entrainment mapping during VT. The VT CL is 620 ms, and pacing is performed at a CL of 580 ms. A low-voltage electrogram is located in diastole on the recordings of the ablation catheter (Map). The stimulus-QRS interval is 230 ms and matches with the electrogram-QRS interval. The postpacing interval (PPI) is equal to the VT CL. The stimulus-QRS/VT CL ratio is 0.37, indicating that the catheter is located in the common pathway. CL = cycle length; PPI = postpacing interval; VT = ventricular tachycardia

Entrainment is a pacing maneuver that helps to distinguish reentrant from nonreentrant arrhythmias and can be used as a mapping tool to target ablation to critical parts of the reentry circuit. Entrainment involves the continuous resetting of a reentry circuit during pacing at sites that are either within or outside the reentry circuit. This technique is used to identify critical sites of the arrhythmia circuit, through the analysis of the QRS morphology, the measured intervals, and the recorded electrograms [31]:

1. The QRS configuration during entrainment provides information about whether the pacing site is within or outside the protected zone of the reentry circuit. With the pacing site being located outside the reentry circuit, during entrainment, the stimulated wavefronts that propagate out from the pacing site collide with the orthodromically propagating wavefront of the reentry circuit and fuse; therefore, the QRS complex is due to the fusion of wavefronts propagating directly away from the pacing site with those emerging from the tachycardia circuit (classic entrainment). During pacing from within a protected region in or near the reentry circuit, pacing entrains VT without changing the QRS configuration (concealed entrainment or entrainment with concealed fusion) [29, 652, 653].
2. The stimulus-QRS interval during entrainment with concealed fusion: Entrainment with concealed fusion indicates that the pacing site is within a protected region of the reentry circuit, and can be located in the common pathway of the reentry circuit (also referred to as a critical isthmus or channel), or in a bystander site that communicates with a critical isthmus. If pacing is performed from a site in the circuit, the stimulus-QRS interval should be equal to the electrogram-QRS interval during VT. On the other hand, if stimulation is performed from a bystander site, the stimulus-QRS interval is longer than the electrogram-QRS interval during tachycardia [30, 654]. A difference in stimulus-QRS and electrogram-QRS up to 30 ms was superior to other criteria for distinguishing bystander sites from critical sites in one study [655]. The stimulus-QRS interval indicates the conduction time from the pacing site to the VT exit site [656]. Similarly, the stimulus-QRS/VT CL ratio is a reflection of the pacing site location within the critical zone of the reentry circuit (Figs. 5 and 6). The exit site is defined as a stimulus-QRS/VT CL ratio < 0.3, the common pathway and entry sites have a stimulus-QRS/VT CL ratio 0.3–0.7, and the inner loop sites have a stimulus-QRS/VT CL ratio > 0.7. There is, however, an important exception to keep in mind: a longer stimulus-QRS/VT CL ratio of >0.7 can occur at critical sites within the reentry circuit. Discrete potentials located in electrical systole rather than diastole that match with the stimulus-QRS interval are present in these situations and reflect that the catheter is within the VT entrance zone [657].
3. The postpacing interval (PPI): The PPI is also an indication of the proximity of the pacing site to the reentry circuit [30]; the PPI measures the interval from the pacing stimulus to the following nonstimulated depolarization recorded at the pacing site. The PPI can be used during entrainment to verify whether the pacing site is within the circuit or is in a bystander area. At a site with concealed entrainment, a PPI that matches the VT CL (±30 ms) is consistent with a site where delivery of RF energy is likely to terminate VT; a PPI >30 ms is often an adjacent bystander [31] when ablation fails to terminate VT. The PPI includes the conduction time for a full revolution of the propagating wavefront through the reentry circuit. Several factors can impact PPI accuracy. A faster pacing rate can result in slower conduction and a prolongation of the PPI. The measurement of the PPI assumes that the recorded electrogram indicates a depolarization at the pacing site. Electrograms generated by remote tissue represent far-field electrograms and can result in erroneous measurements of the PPI. Distinction of local electrograms from far-field electrograms is hence key to accurately measuring the PPI [658].

Both the PPI and the comparison of the stimulus-QRS interval to the electrogram intervals are methods to differentiate bystander sites from critical ablation sites. The method using stimulus-QRS and electrogram-QRS interval assesses a smaller part of the reentry circuit (pacing site to exit site); thus, it could be less susceptible to error than the PPI that assesses a full revolution of the reentry circuit.

Entrainment mapping is most easily used to select ablation sites during mapping of hemodynamically tolerated VTs and is particularly useful in patients with incessant VTs [29]. Unfortunately, the majority of inducible VTs in patients with SHD are not hemodynamically tolerated, and entrainment mapping can only be used for short time periods to confirm that a particular site is critical.

#### How to perform entrainment mapping

Ideally, the VT is hemodynamically tolerated in order to perform entrainment mapping. Furthermore, the VT must have regular RR intervals to prevent erroneous PPI measurements. Pacing is performed at a CL faster than the VT, with care taken to ensure that all QRS complexes and electrograms are accelerated to the pacing rate. Pacing at faster rates can facilitate detection of QRS fusion; however, it can further slow conduction in the circuit, resulting in termination of VT or resulting in acceleration to a different VT. A pacing interval 10–50 ms shorter than the VT CL is frequently used for pacing. Pacing can be performed either in a unipolar manner from the tip electrode or in a bipolar manner from the 2 distal electrodes. Theoretically, the optimal pacing output is slightly above threshold to limit capture to the tissue immediately beneath the distal electrode, which is also the source of the near-field signal. Determining threshold at each site is not practical, however, and some centers use an initial output of 10 mA and a pulse width of 2 ms, which can increase until capture is achieved or which can decrease if capture of a large area is suspected [659]. Pacing for a sufficient duration to be certain that reliable capture is achieved is important.

- Limitations of entrainment mapping are as follows:
  1. Inducibility of sustained and stable VT is required if pacing maneuvers are to be attempted.
  2. Pacing during VT can either interrupt, accelerate, or modify the ongoing VT.
  3. AADs can decrease conduction velocity during pacing, increasing the postpacing and stimulus-QRS intervals [654].
  4. Electrograms indicating local depolarization need to be distinguished from far-field signals, and this is subject to error in regions of fractionated, complex electrograms [30, 31, 652, 660].
  5. Incomplete ablation can create areas where conduction slows or blocks during pacing, creating misleading, increased postpacing and stimulus-QRS intervals.

The advantage of entrainment mapping over a substrate mapping approach is that individual VTs can be reliably and permanently eliminated with few RF ablation lesions when reentry circuit targets are identified [33, 655]. An approach targeting only VT exit sites in the border zone based on QRS morphology and presystolic activity in VT, for example, can fail to eliminate VT if the exit site is deep to the endocardium. Entrainment mapping can potentially identify critical components of the reentry circuit other than the exit site that might be within the reach of the ablation catheter. Unfortunately, most patients with heart disease have multiple VTs that are not hemodynamically tolerated; hence, entrainment mapping as the only mapping technique is insufficient to eliminate most of the VTs encountered in a given patient. In the modern era of VT ablation in the setting of complex VAs and multiple etiologies with different substrates, in which 3D mapping is routinely adopted, entrainment mapping is only one component of the ablation strategy. Ablation guided by entrainment and activation mapping of hemodynamically tolerated VT has not been shown to be superior to substrate-based ablation [661]. Therefore, entrainment mapping is typically combined with substrate mapping in patients with VT associated with scars and SHD.

### Pace mapping

Pace mapping is a technique used to locate the origin of a PVC or VT by stimulating the myocardium to reproduce the clinical 12-lead morphology in the absence of VT; it is particularly useful if the targeted arrhythmia is difficult to induce or is hemodynamically unstable. The optimal site should exactly match the tachycardia QRS, including individual notches as well as major deflections. Comparison of the 12-lead ECG during pace mapping and VT is usually expressed with a scale from 0 to 12; however, computer-assisted comparison allows its quantification [662]. Manufacturer-specific algorithms (Labsystem Pro, Boston Scientific, Arden Hills, MN [formerly Bard]; PaSo CARTO module, Biosense Webster, Diamond Bar, CA; Rhythmia, Boston Scientific, Marlborough, MA; and EnSite Precision, Abbott Laboratories, Abbott Park, IL) exist that use template-matching algorithms to generate a correlation coefficient between VT morphology and pace maps [49, 663]. These algorithms allow computational comparisons between pace maps; however, careful external validation is currently lacking.

Pace mapping indicates the location of the origin of focal VAs [229], although activation sequence mapping is more accurate [664]. Furthermore, pace mapping is used to identify the exit of the VT in reentrant VTs [665–667]. Critical sites of the reentry circuit and regions of slow conduction can be identified based on pace mapping and electrogram characteristics [39]. Pacing in noncritical areas adjacent to the exit can generate an adequate pace match that is similar in morphology to a pace map from the VT exit. On the other hand, pacing at sites close to the entrance of the reentry circuit during sinus rhythm often generates markedly different QRS complexes, given the stimulated wavefront propagates away from the pacing site via paths that are blocked during VT [656, 666, 668]. Thus, sites without matching pace maps could still be critical for the reentry circuit, and identification of a transition from an adequate to a poor matching pace map might indicate the isthmus orientation [49]. Importantly, the pacing rate has been reported to alter the QRS morphology; therefore, a pacing rate close to the VT rate should be used [669].

Within scar, the spatial resolution of pace mapping is heterogeneous and can indicate a region of interest that measures up to 18 cm^2^ [670]. Even in patients without SHD, a perfect pace map can be observed at sites up to 2 cm away from the VT origin [664, 671]. Most electrophysiologists of the writing committee perform pacing in a bipolar manner, starting with an output of 10 mA at 2 ms pulse width. Limitations to bipolar pacing, including anodal capture, and the limitations to pacing with a minimal fixed output, including generation of a virtual electrode that captures more tissue with higher stimulus strength, need to be recognized [42]. Pacing at stimulus strengths only slightly greater than threshold is desirable to avoid capture over a large area, which can reduce accuracy; however, checking the threshold at each mapping point is time consuming and might not be practical.

Smaller interelectrode spacing, smaller electrode size, and unipolar pacing could increase the accuracy of pace mapping (see Section 8.1) [672].

### Sinus rhythm substrate mapping

#### Substrate mapping in sinus rhythm

**Table:**
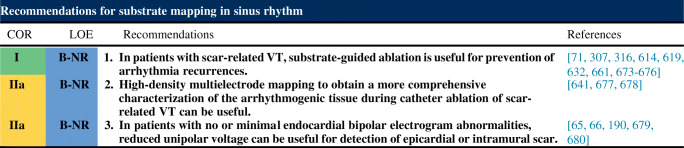

##### Recommendation-specific supportive text

1. Substrate-guided mapping and ablation during sinus rhythm are intended to overcome the limitations of conventional mapping and ablation. They allow ablation of multiple VT morphologies irrespective of inducibility or hemodynamic tolerance. Multiple studies have shown that substrate-guided ablation is effective for prevention of arrhythmia recurrences in scar-related unmappable VT [71, 307, 316, 614, 619, 632, 661, 673–676].
2. Multielectrode mapping has been shown to increase mapping density and to shorten mapping and RF delivery time [641, 677, 678]. Although in retrospective studies the use of multielectrode mapping has been associated with better outcome, no effect on acute and long-term outcome has yet been prospectively demonstrated [677, 678].
3. Bipolar electrograms have a limited field of view to detect epicardially or intramurally located scar. Unipolar voltage mapping can be used to extend field of view of endocardial mapping, and low unipolar voltages can indicate regions of epicardial or intramural involvement in patients with no or minimal endocardial abnormalities evident on bipolar electrogram analysis; however, there is substantial overlap of unipolar low voltage between scar zones and regions without scar [65, 66, 190, 679, 680].

#### Summary

The majority of patients with SHD presenting for catheter ablation have hemodynamically unstable VTs that prevent accurate delineation of the critical part of the reentrant circuit with activation or entrainment mapping [35]. Substrate mapping is an approach to characterize areas likely to support reentry based on electrophysiological characteristics that can be determined during stable sinus or paced rhythm. It allows for elimination of VT, irrespective of inducibility or hemodynamic tolerance. Even for hemodynamically stable VTs, substrate mapping is often used to limit activation mapping or entrainment to a region of interest [661, 673, 676].

The concept of substrate mapping has developed from the success of surgical subendocardial resection for postinfarction VT, which has established the physical link between the VT circuit and the infarction scar [19]. Current criteria to define the abnormal arrhythmogenic substrate rely on a combination of lower bipolar or unipolar voltage and abnormal electrogram characteristics (eg, fragmented, split, and late electrograms). These abnormal electrogram features can represent slow or delayed activation that constitute surrogate markers for potential VT circuits [125, 681].

*Voltage criteria for scar identification*: Scar tissue can be identified based on bipolar electrogram amplitude. Using a 4-mm tip mapping catheter and 1-mm ring interelectrode spacing with a 2-mm ring filtered at 10–400 Hz, 95% of normal LV endocardial electrograms have a peak-to-peak amplitude >1.55 mV [36]. Conversely, bipolar voltage <0.5 mV has been designated as “dense scar,” but it is important to recognize that these regions can still contain viable myocytes [42] that can be captured by pacing. Use of cutoff values has important limitations as shown by recent work integrating voltage mapping and full human heart histology [117]. This is particularly relevant in patients with NICM with patchy scar, in whom larger bundles of surviving myocytes are mixed with fibrotic tissue. Bipolar voltage and electrogram characteristics can be further affected by different electrode size and/or interelectrode distance of the mapping catheter [641], direction of activation wavefront [682], and wall thickness [117].

Bipolar voltage mapping can have a limited field of view to detect epicardially or intramurally located scar, typically observed in patients with NICM. Under these circumstances, unipolar voltage mapping can be used to extend the field of view of endocardial mapping [65, 66, 190]. Endocardial low unipolar voltages could indicate regions of epicardial or intramural involvement in patients with no or minimal endocardial abnormalities evident on bipolar electrogram analysis. Although various studies have reported different cutoff values to identify deeper scar tissue, validation studies with CMR-defined scar have demonstrated that there is a substantial overlap of unipolar low voltage between scar zones and regions without scar. CMR-defined scar remains the gold standard for precise demarcation of scar and is preferable over unipolar voltage mapping for defining scar. Furthermore, cutoff values are impacted by electrode size, wall thickness, surrounding anatomical structures, and ventricular hypertrophy, or are region specific [66, 117, 680, 683].

*Other electrogram characteristics as ablation targets*: Low-voltage areas in patients with SHD are often large, and not all are related to VT. To limit the extent of ablation, several other electrogram features obtained during spontaneous rhythm or elicited by pacing maneuvers have been studied to identify areas within the scar potentially related to VT. In the seminal study of de Bakker et al. [125], the authors have demonstrated the presence of inhomogeneous “zig-zag” conduction within the infarcted area, which was related to the presence of poor cell-to-cell coupling of surviving myocyte bundles interspersed among fibrous tissue. In these regions, abnormal fractioned and LPs were recorded. Although fragmented electrograms can be typically recorded throughout the whole scar, isolated and LPs have been demonstrated to be a more specific marker of the VT circuit [38, 39, 41, 45, 431]. However, clinical studies have adopted heterogeneous definitions of abnormal electrograms, with nonuniform use of terms such as “fragmented,” “split,” and “late,” and the relationship between these surrogate markers and the VT circuits remains to be thoroughly explored [644]. In this respect, LAVA has been proposed as a global term that incorporates all abnormal ventricular signals that represent near-field signals from poorly coupled fibers within scar, representing potential VT isthmus sites [684].

Importantly, the lines of block delimiting the channels responsible for arrhythmias can be both fixed and functional [644], and the participation of functional barriers in VT circuits challenges the identification of potential VT circuits during substrate mapping. Abnormal local electrograms can be hidden in far-field signals, preventing their recognition during sinus rhythm or RV pacing. Identification of such electrograms might require pacing maneuvers to demonstrate their poorly coupled nature [127, 684, 685]. This could be relevant in the presence of smaller, nontransmural scars, in which subepicardial viable myocardium can overlay the subendocardial scar, creating far-field obscuration of the local signal [686].

Some abnormal electrograms can be disclosed only by catheters with small electrodes and shorter interelectrode distance, which allow for higher near-field resolution [81] (see Section 8.1).

Most of the studies concerning substrate mapping were performed on patients after MI, and significant differences exist with respect to the arrhythmogenic substrate characteristics between patients with ICM and NICM (see Section 9).

There is currently no standardized approach for substrate-guided ablation. A number of substrate-based ablation approaches have been developed, with the main purpose of targeting and eliminating areas of slow conduction within the abnormal substrate defined during sinus rhythm [36, 38, 39, 46, 50, 51, 684, 687, 688] or evoked by pacing [127, 685, 686] (see Section 9.5).

### Intraprocedural imaging: Intracardiac echocardiography, fluoroscopy, cardiac magnetic resonance imaging

#### Intraprocedural imaging during catheter ablation of ventricular arrhythmias

**Table:**
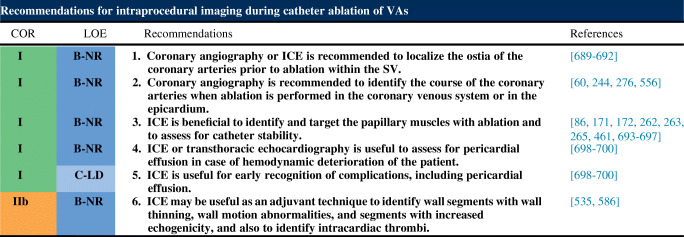

##### Recommendation-specific supportive text

1. For the coronary ostia, a distance of ≥1 cm of the catheter tip from a coronary ostium is considered a safe distance [691], although ablations have been reported to be as close as 7.3 mm [689] and 7.5 mm [692] from the coronary ostium, without resulting complications.
2. In the epicardial space, coronary angiography should be considered prior to ablation to ensure that there is no coronary artery in the proximity of the ablation catheter [60, 556]; a minimal distance of >5 mm from the ablation catheter tip to an epicardial coronary artery has been considered safe [556].
3. No randomized studies have shown improved outcomes if ICE is used to target papillary muscle arrhythmias. Several case series, however, have demonstrated that ICE is useful for targeting papillary muscle arrhythmias with real-time localization of the papillary muscle apparatus and the ablation catheter tip [86, 171, 172, 262, 263, 265, 461, 693–697]. Catheter stability on the papillary muscles or other parts of the myocardium can be assessed with ICE.
4. ICE further can help to identify complications such as pericardial effusions, allowing for early intervention [698–700]. It also can be used to assess intracardiac structures, including wall thickness, wall motion abnormalities, increased echogenicity to identify scarring, and intracardiac thrombi [535, 586, 681]. Furthermore, ICE is beneficial in monitoring catheter contact during ablation for arrhythmias not related to papillary muscles and could be useful to assess for tissue changes preceding steam pops [701].

#### Summary

For intraprocedural imaging during catheter ablation of VAs, several technologies have been used: fluoroscopy (including coronary angiography), echocardiography (intracardiac, transthoracic, or transesophageal), and real-time CMR. Although no randomized studies have demonstrated the benefit of intraprocedural imaging, there are data supporting the use of coronary angiography or ICE to enhance procedural safety for selected cases, allowing for real-time imaging while mapping and ablation are performed [60, 86, 171, 172, 244, 262, 263, 265, 276, 461, 556, 689–697].

Fluoroscopy is routinely used for catheter placement and catheter manipulations during mapping and ablation procedures. With the development and improvement of EAM systems, the need for fluoroscopy can be minimized [702, 703].

Integration of fluoroscopic cine loops into electroanatomical maps has further helped to shorten fluoroscopy times [704–706].

With respect to the coronary arteries, the use of coronary angiography or ICE to localize the ostia of the coronary arteries has been found useful when ablation is directed toward the SV [689–691]. Although data are limited, a minimal distance of ≥10 mm from the coronary ostia has been considered to be safe for RF ablation [461]. The coronary ostia can also be identified by ICE and can be marked in the electroanatomical map. This could obviate the need for coronary angiography. Coronary angiography is the preferred technique to identify the course of the coronary arteries when ablation is performed in the coronary venous system or the epicardium. A distance of >5 mm of the ablation catheter to an epicardial coronary artery is considered safe [691]. A coronary angiogram is often repeated at the conclusion of the procedure to document patency of the coronary arteries.

ICE is beneficial to identify and target VAs originating from the papillary muscles [86, 171, 172, 262, 263, 265, 693–698]. No randomized studies, however, have shown improved outcomes when ICE was used. Several case series, however, have demonstrated that ICE is useful for targeting papillary muscle arrhythmias. The main benefit is the real-time localization of the papillary muscle apparatus in conjunction with the ablation catheter tip being visualized in the EAM system. Integration of ICE images into the EAM system together with the mapping or ablation catheter is particularly helpful [171]. ICE helps to define the level of the pulmonary valve when arrhythmias are targeted in the RVOT or in the pulmonary artery. This is of importance when ablation needs to be performed in the pulmonary artery, which can be in close proximity to the coronary arteries. Furthermore, ICE can be used as an adjuvant technique to identify scarring by identifying wall segments with wall thinning, wall motion abnormalities, excessive trabeculations [700], segments with increased echogenicity [586, 681], and also to identify intracardiac thrombi [535]. Furthermore, predictors for steam pops can be identified with ICE [701], which might help to prevent complications.

ICE further can help to identify complications such as pericardial effusions, allowing for early intervention [698–700]. Transthoracic echocardiography, as well as ICE, can be useful to assess for pericardial effusion in case of intraprocedural hemodynamic deterioration of the patient [707].

Real-time CMR can enable visualization of lesion formation. It has the advantage of avoiding ionizing radiation and of imaging the whole heart in real time, and it has been used to target typical atrial flutter [708, 709]. However, real-time imaging during VT ablation in humans has not yet been reported and has thus far been limited to postablation image acquisition [710]. A laboratory with real-time CMR capabilities requires a major financial investment, adaptation, and commitment to a work flow that is very different from that of a standard electrophysiology laboratory. At this time, real-time CMR is used only in a few centers on an investigational basis. Other investigational imaging techniques include the use of endoscopy in the epicardial space for visualization of catheters, epicardial structures, and ablation lesions [711].

### Electroanatomical mapping systems and robotic navigation

**Table:**
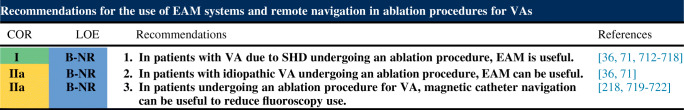

#### Recommendation-specific supportive text

1. EAM systems combine cardiac electrical information obtained from catheter-mounted electrodes and 3D spatial location information to reconstruct an image that represents the targeted cardiac chamber [723]. Several EAM systems are commonly used in clinical practice. The CARTO mapping system (current version CARTO 3, Biosense-Webster, Diamond Bar, CA) makes use of magnetic field differences for accurate localization of proprietary mapping and ablation catheters. This system also uses an impedance-based algorithm to visualize the electrodes and shafts of various diagnostic catheters. A proprietary intracardiac ultrasound catheter can interface with the mapping system to further define cardiac geometry within the mapping field [712]. The EnSite Precision system (Abbott Laboratories, Abbott Park, IL; formerly EnSite Velocity and EnSite NavX, St. Jude Medical, St. Paul, MN) uses voltage and impedance measurements to localize diagnostic and ablation catheters. With the use of proprietary catheters, the EnSite Precision system can now also provide magnetic-based navigation. The Rhythmia HDx mapping system (Boston Scientific, Marlborough, MA) uses both magnetic- and impedance-based methods for catheter tracking. A prominent feature of this system is the proprietary 64-pole mini basket catheter with closely spaced electrodes. The Topera mapping system (Abbott Laboratories, Abbott Park, IL) combines unipolar electrogram data from an intracavitary basket catheter with proprietary electrogram analysis software algorithms to identify focal and rotational activity during cardiac fibrillation. Though it is marketed for atrial mapping, it has been used in cases of idiopathic VF [724]. Access to EAM systems worldwide is limited, largely due to the high cost of systems, necessary peripherals, and proprietary catheters.
  Regardless of the type of EAM available, knowledge of the first principles of cardiac electrophysiology remains essential to create maps that accurately represent the physiology and anatomy. Maps created using EAM systems are subject to considerable variability depending on a number of factors, including (but not limited to) accurate annotation of electrogram qualities, consistent catheter contact with tissue, distributed sampling of the entire structure of interest, density of location “points” in the map, type of rhythm being mapped, direction of activation wavefront propagation, and the size and spacing of the electrodes used to acquire the data. Numerous technologies have been developed or are in development to improve mapping quality and user experience. These include automated chamber segmentation, imported scar delineation from alternative cardiac imaging, such as CMR, CT, and echocardiography, and automated electrogram analysis tools. Ultra high-density mapping catheters have significantly changed the resolution of scar features and, as a result, our understanding of VT circuit physiology.
  EAM has proven to be a versatile technology to guide treatment of a wide range of arrhythmias. Prospective randomized trials with and without EAM in patients with SVT demonstrate similar acute procedural success rates and substantial reduction in fluoroscopy use [713, 714]. No such randomized trials with and without EAM exist for VAs, and no prospective trial has demonstrated superior outcomes with the use of EAM. It is generally accepted that the use of EAM can reduce fluoroscopy time and allow for more precise mapping, with comparative analyses of nearby locations during VA ablation procedures. Particularly for more complex strategies of mapping ventricular scar, EAM enables the user to perform the necessary mapping to achieve a successful result, which would otherwise be impossible without EAM [36].
  In patients with SHD undergoing catheter ablation for VA, EAM is particularly helpful. EAM is especially versatile in this setting, offering the operator the possibility of using various mapping strategies, including activation maps, entrainment maps, pace maps, electrogram amplitude (voltage) maps, and tagging location of specific electrograms of interest. A prospective multicenter trial for VA ablation in patients with IHD has used EAM [71]. Several retrospective reports have used EAM for a wide range of SHDs and locations within the heart [38, 42, 54, 349, 716, 717, 725–729].
2. In patients with idiopathic VA undergoing catheter ablation, EAM can be useful. It is technically feasible to perform successful ablation for idiopathic VA without EAM, using 12-lead ECG, fluoroscopy, and careful analysis of intracardiac electrograms. Common examples include ablation of BBRVT and idiopathic VT originating from RVOT or LV fascicular system locations. For most centers, when EAM is available, it has become a mainstay in the procedure workflow for VA ablation procedures. EAM systems allow activation mapping of idiopathic VA to support catheter ablation in the RV, LV, aortic sinuses, and coronary venous system [36, 373, 715]. Reasons to not choose EAM for ablation of idiopathic VA include increased cost and limited access.
3. Catheter-based ablation of VA requires significant demands on the skill and experience of the operator. The concept of remote or robotic catheter navigation is appealing to reduce the physical demands of the procedure while developing more advanced abilities to achieve stable and precise catheter location than traditional catheters can afford. Several technologies have been developed to achieve these goals. The Niobe magnetic navigation system (Stereotaxis, St. Louis, MO) remotely controls the tip of a proprietary ablation catheter using changes in magnetic field direction from large rotating earth magnets [730]. This system can be combined with robotic catheter manipulation components (Vdrive system) to control various diagnostic catheters remotely as well. Previous technologies include a second magnetic navigation system, the Catheter Guidance Control and Imaging system (Magnetecs, Inglewood, CA), which used 8 electromagnets to guide a proprietary magnetically tipped catheter. The Sensei robotic system (Hansen Medical, Mountain View, CA) robotically steered a deflectable sheath. A stand-alone robotic catheter manipulation system (Amigo, Catheter Precision, Ledgewood, NJ), interacted with a wide range of diagnostic and ablation catheter handles via a handheld remote control. Use of such technologies is a matter of operator preference: the potential advantages are offset by additional costs for the navigation systems, disposables, and maintenance contracts.
  For patients undergoing ablation for VA, the use of an approved magnetic navigation system can be useful to reduce fluoroscopy exposure. One small prospective randomized trial comparing magnetic navigation to manual ablation demonstrated reduced use of fluoroscopy without clear differences in procedure outcome [721]. A retrospective case series supported similar conclusions, with a low rate for procedural complication [731]. A multicenter, single-arm study using remote navigation for patients with ischemic VT has demonstrated favorable procedural outcomes of VT noninducibility and longer-term VT freedom rates that are comparable to published results for manual catheter ablation [732]. Several retrospective comparison studies and single-arm series in patients with SHD consistently support reduced fluoroscopy times during the procedure and low rates of procedural complication [722, 733–739]. None of the remote navigation systems are specifically indicated to facilitate the catheter ablation procedure for VAs, though a prospective clinical trial using the Niobe remote magnetic navigation system is underway [740].

## Mapping and ablation

### Ablation power sources and techniques

**Key Points**

• An impedance drop ≥10 Ω or a contact force ≥10 g is commonly used as a target for RF energy delivery.

• The use of half normal saline generates larger ablation lesions but can result in steam pops.

• Simultaneous bipolar or unipolar ablation can result in larger ablation lesions.

• Cryoablation can be beneficial for achieving more stable contact on the papillary muscles.

• Ethanol ablation can generate lesions in areas where the arrhythmogenic substrate cannot be otherwise reached, provided that suitable target vessels are present.

• Stereotactic radiotherapy is an emerging alternative to ablation, requiring identification of a region of interest that can be targeted prior to the radiation treatment.

#### Introduction

Successful ablation requires the creation of durable lesions of adequate size [741]. When successful ablation cannot be achieved from the endocardium, percutaneous access of the pericardial space (discussed in Section 6.3) that permits contact mapping and ablation of the arrhythmogenic substrate, which is close to the epicardial surface, can be effective but is limited when there are pericardial adhesions, overlying fat [742], or critical structures nearby [562, 743], or when the substrate is located deeper within the myocardium [338]. Innovative techniques intended to reach deeper arrhythmogenic substrates have been developed.

#### Unipolar radiofrequency catheter ablation

Unipolar RF energy has been the mainstay of catheter ablation technologies since the early 1990s. This technology was refined with the addition of temperature sensing and larger tip catheters and further with catheter tip cooling. Lesion depth is limited by the amount of power that can be safely delivered: too much power will result in overheating at the tip-tissue interface and consequent protein denaturation with thrombus formation [744, 745]. Irrigation of the ablation electrode reduces temperature at this interface and allows greater power delivery and thus the creation of larger lesions [746–748]. Several catheter tip designs and irrigation rates have been made available, each of which generates similar myocardial lesion depth and volume [749]. Some degree of surface sparing is observed with irrigation, more so at higher flow rates and with tip designs that direct more irrigant at the surface [749, 750]. Power delivery, however, remains limited by the risk of deep tissue overheating and steam pops [747]. Catheter tip designs with greater irrigant dispersion might permit lower irrigation flow rates and less volume load for patients with reduced ventricular function. Ablation lesion size increases with the duration of current application [751], and although short duration applications are sufficient for ablation in thin structures, 60–90 s or longer applications are usually employed for VT. The optimal duration has not yet been defined.

Steam pops can occur when excessive heating of myocardial tissue occurs and can occasionally result in harmful tissue disruption [752]. However, the methods for avoiding steam pops are limited. Catheter tip irrigation partially attenuates the correlation between tip temperature and tissue temperature. Steam pops can still occur when tip temperatures rise excessively, although higher flow catheters with more irrigation ports could reduce the incidence of steam pops [753]. Monitoring the impedance could be useful in preventing steam pops, most of which occur when the impedance decreases more than 18 Ω from baseline or when greater power is delivered for longer durations [754]. A typical initial power setting for ablation with an open irrigated catheter in the LV is 30 watts and can be adjusted up to 50 watts to achieve an impedance drop of 10 Ω.

#### Contact force sensing

Lesion size is critically dependent on the contact of the ablating electrode with the tissue [755]. Catheters with contact force sensors have become common and can help to ensure lesion creation at intended sites [596]. Although a clear improvement in clinical outcomes for catheter ablation for VA is difficult to demonstrate [756], RF applications with a mean contact force >10 g are more likely to result in electrical unexcitability in scar areas [757], and contact force sensing has become an integral part of RF catheter ablation [758].

#### Hypotonic external irrigation

Most ablation catheter tips are 3.5–4 mm in length and are typically irrigated with 0.9% sodium chloride solution. The efficiency of delivering current to myocardial tissue can be increased by using smaller electrode tip sizes [759] and less conductive irrigant, such as 0.45% sodium chloride [76, 760]. These techniques deliver greater current to myocardial tissue for any given applied power but remain limited by the potential for tissue steam pops. The effectiveness and safety of using a higher impedance irrigant has been supported by a multicenter, prospective, observational human study; however, direct clinical comparisons are still pending [761]. The use of hypotonic saline irrigant can have the additional advantage of a reduced salt load for patients with compromised ventricular function.

#### Simultaneous unipolar or simultaneous bipolar radiofrequency delivery

Conventional unipolar RF catheter ablation delivers current between the catheter tip and a dispersive skin electrode. Two catheters placed on either side of the target tissue, delivering simultaneous unipolar RF energy (using two RF generators), have been employed to reach deeper myocardial substrates, which could create larger lesions than sequential ablations at each of these sites by raising the temperature of tissue deep to either ablation site, limiting its heat sink effect, and increasing the conductive heating zone [762]. This method provides independent control and monitoring of RF delivery from each ablating catheter but can pose technical challenges with device interactions. The efficacy and risks of this procedure have not been completely characterized.

Bipolar ablation is performed by replacing the dispersive skin electrode with a second catheter that is closely apposed to tissue with culprit substrate between the catheters. This technique concentrates current density between the catheters. Lesion creation extends from both catheters, and lesions are larger and more likely to be confluent than those achieved with sequential unipolar energy delivery [763]. The efficacy of this technique has been demonstrated in both preclinical models [764, 765] and in humans [75, 766]. Bipolar ablation can be delivered between catheters that are positioned across the interventricular septum from one another, on either side of a papillary muscle, or with catheters in the endocardial and epicardial spaces. Bipolar ablation is limited by the requirement for custom cabling and the technical challenges of visualizing and placing two catheters sufficiently close with target tissue between them. The catheter tip temperature cannot be monitored simultaneously for both catheters, and impedance will be influenced by both tip-tissue interfaces. Optimal power and current settings have not yet been defined, and the risk of coagulation has not yet been fully characterized. An investigational system is under evaluation.

#### Needle ablation

Infusion needle ablation has been reported as a means to achieve intramural ablation lesions. A catheter with an extendable/retractable needle at the tip can function as an intramural electrode for the temperature-controlled delivery of RF energy [767], which can create large, deep lesions during saline irrigation [768, 769]. This approach has been demonstrated to be effective for select patients with treatment-refractory VA in small series [77]. The efficacy and procedural risks of this technique have not been fully characterized. Investigational systems are under evaluation [770].

#### Cryoablation

Catheter cryoablation has been infrequently employed for treating VA. Compared with RF ablation, focal cryoablation lesions are smaller and take longer to develop. Larger lesions can be created with larger tip cryoablation catheters [771], but the clinical effectiveness and recurrence rates are less favorable than those achieved with RF ablation [772, 773], likely because of the smaller lesion size. Cryoablation catheters become adherent to the tissue during lesion creation, which enhances catheter stability and can provide additional utility in the catheter ablation for VA arising from highly mobile papillary muscles, which can be difficult to ablate using RF ablation catheters [774]. Although surgical cryoablation for VA has been used for decades [775, 776], currently available technology will likely be limited to specialized circumstances unless further innovation enables larger cryoablation lesion delivery from catheter platforms [777].

#### Transvascular ethanol ablation

Creating an arterial occlusion and controlled infarction has long been used to create ventricular ablation lesions [778]. Early experiences had moderate success but with a higher risk of complications [779]. The refinement of mapping strategies and percutaneous coronary interventional technology encouraged further development, and several series have reported on treatment-refractory patients who underwent transcoronary ethanol ablation [74, 780]. Larger series have demonstrated that this technique’s efficacy can be limited by the availability of suitable target vessels, its procedural risk, and the risk of collateral injury [742]. Heart block is a frequent risk when a septal substrate is targeted [73].

Although experience is limited, ventricular ablation can be performed by injecting alcohol in a retrograde manner into the coronary venous system, which can lead to a lower risk than in the coronary arterial system. Reports from small series have been promising, although collateral myocardial injury and recurrence are still a concern [781–783]. Further study could help to refine the technique.

#### Stereotactic radiotherapy

The use of stereotactic motion-gated external beam radiation for ablation has been explored in animal models [784], and its feasibility has been demonstrated in humans [785–788]. Tissue injury is likely the result of a combination of cell nuclear damage and vascular damage, the results of which can manifest within days or weeks [785]. This method requires the accurate preprocedure identification of the culprit arrhythmogenic substrate, using either intracardiac mapping, imaging, or noninvasive ECGI [785]. Limiting the radiation to the target is complicated by cardiac and respiratory motion. Further study is required to quantify the risk of injury to nontarget myocardial and adjacent structures and to further define the methods and efficacy.

### Idiopathic outflow tract ventricular arrhythmia

**Key Points**

• The RVOT, pulmonary arteries, SVs, LV epicardium and endocardium contain most of the OT arrhythmias.

• Activation mapping and pace mapping can be used to guide ablation in the RVOT.

• Imaging of coronary artery ostia is essential before ablation in the aortic SVs.

• The LV summit is a challenging SOO, often requiring mapping and/or ablation from the RVOT, LVOT, SVs, coronary venous system, and sometimes the epicardial space.

• Deep intraseptal VA origins can be challenging to reach.

#### Introduction

Approximately two-thirds of idiopathic VAs originate from the ventricular OTs, accounting for 10% of all patients referred for VA ablation [789]. OT VAs comprise a wide spectrum of VAs, ranging from premature ventricular beats to sustained monomorphic VT. Approximately three-quarters originate from the RVOT, with the remaining from the LVOT and adjacent structures, including the pulmonary artery, the area surrounding the His bundle, the aortic SVs, the epicardium and GCV region, LV summit, AMC, and the superior mitral and tricuspid valve annuli [87, 96, 268, 286, 789–795]. OT VAs usually exhibit either an RBBB or LBBB morphology with an inferior axis. OTs and adjacent areas have a broad and complex 3D anatomical structure, accounting for some of the unique challenges in arrhythmia localization and ablation [790, 792–794].

Most idiopathic OT VAs have a focal origin and are thought to be due to adenosine-sensitive, cAMP-mediated triggered activity [624, 796].

#### General approach

The success of ablation depends largely on the presence of VA at the time of the procedure. All AADs should be discontinued for at least 5 half-lives before the procedure. Sedation with long-acting sedatives can decrease spontaneous or inducible VA and should be avoided if possible. If spontaneous VA is absent, limited physical activities, including hand grip and leg-rising exercises, programmed stimulation, burst pacing, and the administration of isoproterenol, epinephrine, or phenylephrine, can be helpful in eliciting the arrhythmia (Section 7).

Ablation is based on activation mapping and pace mapping [792, 797], the latter of which is helpful when the VA is infrequent. Activation mapping is, however, more accurate [664], given a similar paced QRS morphology can be observed over a relatively large area [797] and can be misleading in the SV [797, 798].

The most frequent SOO remains the RVOT [789], but failure of catheter ablation at the earliest RVOT site should raise concerns that the RVOT breakthrough is not sufficiently close to the focus, warranting further mapping, including adjacent structures. A stepwise approach to the detailed and careful mapping of the region of interest, avoiding futile ablation at inappropriate sites, shortens the procedure and enhances success [460, 792].

Detailed knowledge of OT anatomy is required, and the use of fluoroscopy alone is often sufficient for guiding catheter position. However, preprocedure CT or CMR can help to exclude myocardial disease and define the anatomy that can be registered in the mapping system. ICE can help to precisely determine the catheter location in relation to the valves and the adjacent coronary arteries at risk of injury from ablation in the SV or pulmonary artery.

For VA with LBBB morphology, mapping should start at the RVOT and pulmonary artery. When mapping indicates a focus outside the RVOT and pulmonary artery, mapping of the GCV via the CS provides useful information prior to left heart catheterization. Mapping can then include the AIV and its septal branches followed by the SV and endocardial LVOT.

#### Right ventricular outflow tract and pulmonary artery

VAs ablated at the RVOT typically display LBBB morphology and transition at or > V3, without broad initial r waves in the right precordial leads. Most VAs arise from the septal anterior aspect of the RVOT. ECG algorithms can help to suggest the specific region [799] and identify those with this morphology that can arise outside the RVOT or in the pulmonary artery [460, 462, 800]. Given that the posterior RVOT abuts the RSV, mapping of this structure is useful when ablation in the RVOT fails [793]. Some RVOT VAs originate from a conus papillary muscle that can be identified by ICE [801].

Myocardial fibers extend along the pulmonary artery and can give rise to VAs that can be ablated from within the pulmonary artery. ECG and electrophysiological characteristics of pulmonary artery VAs are not well delineated [791]. Mapping at the pulmonary artery should be considered while performing catheter ablation at the RVOT. ICE or angiography is required to know with certainty whether the catheter is above or below the valve.

Acute procedural success is in the range of 90% [217, 237, 797], with more than 80% free of recurrent VA during follow-up. Serious complications occur in approximately 1% of patients and are usually related to cardiac perforation [797]. The left main coronary artery is directly posterior to the distal RVOT and is at risk of injury when ablating in the distal posterior RVOT [802] or the pulmonary artery. The left anterior descending artery can be damaged when ablation is performed at the insertion of the anterior free wall to the septum of the RVOT, especially when high power (>30 watts) is used for ablation.

#### Aortic sinuses of Valsalva

The aortic root lies posterior and rightward of the RVOT and is comprised of the SVs and commissures. Both the RSV and the anterior part of the LSV are connected to the adjacent LVOT, while both the posterior LSV and the NCSV are in fibrous continuity with the mitral annulus at the AMC [793, 794, 803]. Although this is a fibrous structure, rare VAs can arise from this region. Myocardial sleeves can cross the aortic valve plane and extend a few millimeters above the basal valvular attachment, most commonly adjacent to the RSV and sometimes at the LSV. Myocardial sleeves are rarely observed adjacent to the NCSV [793, 794, 803].

The ECG characteristics of VAs from the SV vary, but relatively common features that are not usually encountered in RVOT VAs include a QRS transition <V3, prominent tall or broad R waves in V1 or V2 [792, 795, 803–806], qrS or notched complexes in V1–V2 [239, 242], and a large R wave in lead I [807]. A single focus can result in VAs with different QRS morphologies (RBBB and LBBB), suggesting a fiber with multiple OT connections [808].

Initial mapping should include the LVOT and SVs [809]. ICE is useful for confirming the catheter position and the relation to adjacent structures. Local electrograms are helpful indications of catheter location: in NCSV, the atrial electrograms are larger than the ventricular electrogram, whereas the ventricular electrogram is larger in the other SVs, particularly in the RSV [692, 793, 803, 810]. SV foci are typically characterized by a small pre-QRS fragmented electrogram that precedes a large far-field ventricular electrogram during the VA and follows the QRS and far-field ventricular potential during sinus rhythm [242, 793, 803].

Prior to ablation, ICE imaging or coronary angiography is required to ensure that the distance between the ablation catheter and the nearest coronary ostia exceeds 10 mm [803, 804, 811]. Coronary angiography can be repeated after the ablation to confirm the absence of injury or spasm. Injury to the aortic valve is theoretically possible. An RF power of 25–35 W and a duration of 30–60 s for each RF energy application are commonly employed for irrigated or nonirrigated RF ablation catheters [803].

The compact AV node and His bundle are potentially at risk during ablation from the NCSV or commissure between the NCSV and RSV and can be identified by the presence of a His potential [794, 803]. AV conduction should be monitored during ablation, which might necessitate the delivery of RF energy during sinus rhythm rather than during tachycardia, as well as gradually increasing power [803].

Ablation for VAs at the aortic root is usually successful [664, 692, 795]. In rare cases, earliest activation is identified at a coronary artery ostium or even within the proximal coronary artery, precluding ablation directly on the focus [692, 804]. Potential complications include aortic valve injury, coronary artery injury and thromboemboli; with appropriate precautions, however, these complications appear to be rare.

#### Left ventricular outflow tract and left ventricular summit

Endocardial left OT VAs can originate from the superior basal regions of the interventricular septum or the LV free wall, near the aortic annulus, including the area adjacent to the His-bundle region where ablation carries a risk of AV block. The QRS typically has an LBBB inferior axis configuration, with R waves in V1, V2 and dominant R waves in these leads for those originating in the more leftward aspect of the LVOT near the AMC.

**Fig. 7:**
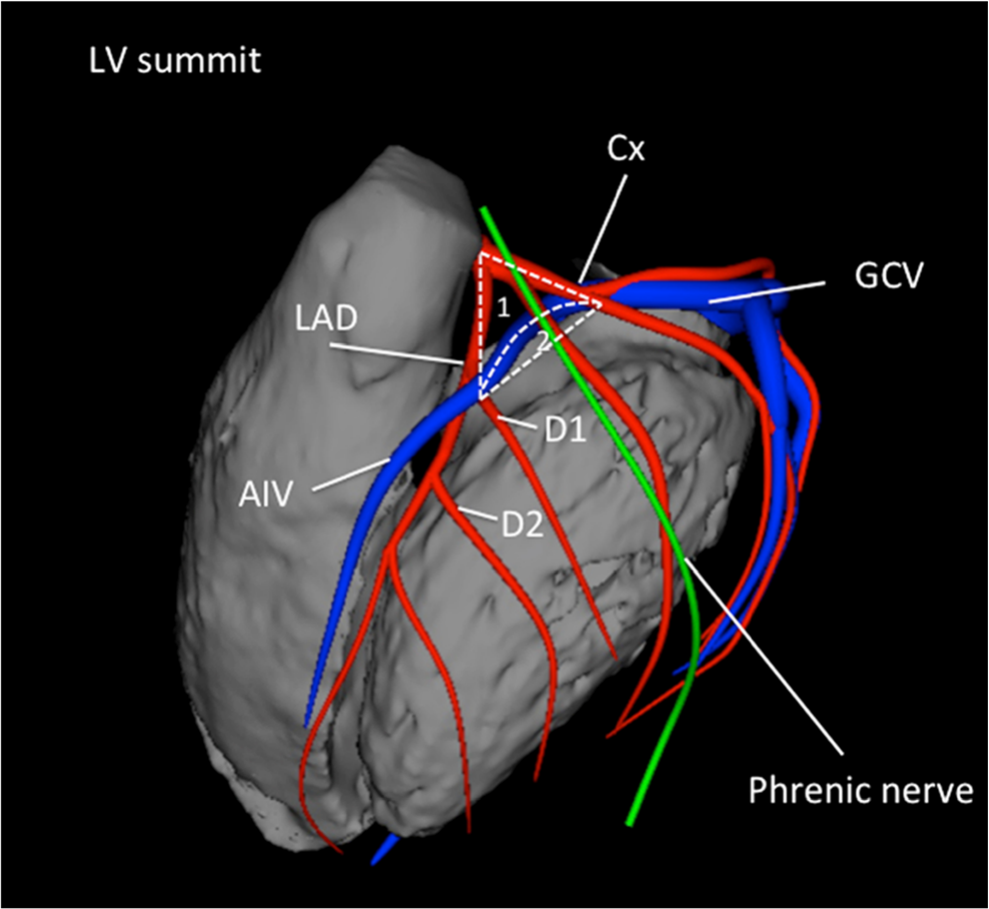
Anatomical boundaries of the LV summit, with the inaccessible [1] and accessible [2] parts. Shown are the left anterior descending artery (LAD), the circumflex artery (Cx), the great cardiac vein (GCV), the anterior interventricular vein (AIV) and the first and second diagonal branch of the LAD (D1, D2)

The epicardial area bounded by the left anterior descending and circumflex coronary arteries has been termed the LV summit (Fig. 7) [792]. Ablation for VAs in this region can be challenging and less successful than other OT VAs. The GCV divides the LV summit into two parts: the inferior part can be accessible to epicardial catheter ablation, while the superior one—the triangle of Brocq and Mouchet [812]—is inaccessible due to the close proximity of the coronary arteries and overlying epicardial fat [246]. One group has reported success in mapping and identifying origins of VA from the summit region within communicating veins between the aortic cusp and the pulmonary artery in half of the patients allowing for anatomic targeting of these VAs [813]. Ablation in the adjacent endocardium is occasionally successful even when activation is later than 10 ms prior to the QRS onset at that site [247, 275, 814]. VAs originating from the apical summit are rightward of those in the basal part and tend to have a longer QRS duration, more rightward axis, and more often an RBBB configuration compared with those from the basal part of the summit [275]. Ablation from within the GCV or epicardium can be successful for VAs arising from the inferior part of the summit.

Epicardial VAs often cluster at perivascular sites, with the majority at the junction of the GCV/AIV or at the proximal AIV [244, 804, 806, 815]. ECG characteristics can be similar to those of VAs originating from the SVs. Mapping within the GCV/AIV can be performed using standard RF catheters or small-diameter (2-Fr) multipolar electrode catheters [804, 806]. RF ablation in the coronary veins has a risk of perforation or injury to adjacent coronary arteries, or, in some patients, injury to the left phrenic nerve. Some physicians perform ablation in the GCV only after failure of RF energy application at the adjacent LVOT endocardium and SV, even if activation at these sites occurs later. Coronary angiography prior to ablation in the vein is essential, and a distance of 5 mm from the adjacent artery is recommended for ablation to be considered [804, 815]. An irrigated catheter is needed due to the low flow in the vein, and the impedance cutoff is usually disabled given that impedance in the vein might exceed the set parameters. Some physicians limit the maximum power to 20 W, the maximum temperature to 43 °C, and the RF duration to 60 s [804]. Cryoablation has been considered when the target site is very close to the artery, but arterial injury is also possible with cryoablation [247, 804]. To avoid phrenic nerve injury, ablation should not be attempted if there is diaphragmatic capture while pacing at the site at 20 mA.

In some cases, VAs in the apical portion of the LV summit are sufficiently leftward to be accessible from the pericardial space [246, 275, 804]. Proximity to major coronary arteries and overlying epicardial fat can limit ablation [246]. When proximity to the epicardial coronary artery precludes catheter ablation, a direct surgical approach can be an option [816–818].

#### Para-Hisian ventricular arrhythmias

The His bundle is located at the most proximal and rightward part of the RVOT in the region of the perimembranous septum. VAs originating from this area often have distinctive ECG characteristics, including a QS in V1–V2, relatively narrow QRS, and tall R waves in the lateral leads [286]. Ablation risks include damage to the His bundle or RBB and the possibility of complete AV block. When earliest activation is recorded near the His bundle, mapping at the RSV and NCSV should be performed, given that earlier activation can be identified at these sites [692, 810, 819]. Ablation with careful monitoring of AV conduction can be successful. Cryoablation can be an option to avoid AV block [595].

#### Deep intraseptal sites

Often, idiopathic OT VAs can arise from deep intraseptal sites and share LBBB morphology with inferior axis [278]; however, no specific ECG pattern has been described indicating an intramural origin. Identification of the SOO is often only possible in retrospect after several structures (including the RVOT, LVOT, cusps, and coronary venous system) have been carefully mapped, and timing has been found to be equally early in several anatomical structures without matching pace maps. Activation mapping from within a vascular perforator branch within the interventricular septum can be helpful in identifying the SOO that can be targeted from an adjacent endocardial site [278]. Temporary suppression of VAs by cold saline infused into the distal coronary venous system and perforator veins can also suggest an intramural septal focus [820]. Body surface mapping and multisite pacing have been found to help in indicating a deep septal origin [821, 822]. Ablation is attempted by RF application at the earliest endocardial breakthrough sites or as close as possible to the site identified by intraseptal mapping. If this is ineffective, simultaneous unipolar [762] or simultaneous bipolar RF ablation (investigational) between two RF catheters positioned at both breakthrough sites [804], or between RVOT and SV [227], or RF ablation directly within a septal perforator vein [278] have been reported. Intracoronary ethanol infusion [792] and the use of an investigation needle ablation catheter have been reported [77].

### Idiopathic nonoutflow tract ventricular arrhythmia

**Key Points**

• VAs originating from the papillary muscles can be challenging due to multiple morphologies of the VA and the difficulty in achieving and maintaining sufficient contact during ablation.

• VAs originate in LV papillary muscles more often than in RV papillary muscles; they more often originate from the posteromedial than the anterolateral papillary muscle and occur more often at the tip than at the base.

• Pace mapping is less accurate than in other focal VAs.

• ICE is particularly useful for assessing contact and stability.

• Cryoablation can also aid in catheter stability during lesion delivery.

#### Ventricular arrhythmias from the tricuspid and mitral annuli

Arrhythmias arising from the mitral annulus occur in approximately 5% of patients presenting for ablation for idiopathic VAs and can arise from anywhere around the mitral annulus, with a slight predilection for the superior aspect [95, 268, 269]. In the surface ECG, VAs from the mitral annulus have an RBBB morphology, with dominant R waves from V1 to V5, and can have an S wave in V6 [95, 267, 269]. Successful ablation has been reported in 80%–100% of cases [95, 268, 269]. Some cases have an epicardial origin and can be approached from the CS or GCV, in which case the use of coronary angiography to verify a sufficient distance from the adjacent coronary arteries is warranted.

VAs from the tricuspid annulus are encountered in 8%–10% [823] of patients with idiopathic VA referred for ablation [96]. VAs from the tricuspid annulus have an LBBB morphology, positive polarity in leads I, V5, and V6, and a QS or rS pattern in aVR. Compared with RVOT arrhythmias, lead I has a taller R wave. A precordial R wave transition >V3 favors a free wall location, and a QS pattern in V1 favors a septal location [96, 823].

Outcomes from ablation for VAs from the free wall of the tricuspid annulus are favorable, with success rates of approximately 90% [96, 823]. Ablation for VAs from the septal side of the tricuspid annulus are more challenging due to difficult catheter contact, proximity to the conduction system, and a possible intramural origin for the arrhythmias [96]. Use of a deflectable sheath can help to improve catheter contact, and exploration of the LV or SV and the coronary venous system should be considered when ablation at the RV septum is unsuccessful [139]. The risk of AV block should be considered, and careful mapping of the conduction system prior to ablation at the septal tricuspid annulus is warranted.

#### Mapping and ablation of ventricular arrhythmia from the papillary muscles

Ablation of papillary muscle arrhythmias presents unique anatomical challenges that can result in long procedural, fluoroscopy, and RF times [237]. Idiopathic VAs from the papillary muscles comprise up to 5% of patients presenting for PVC ablation [237], typically have a focal origin consistent with triggered activity or abnormal automaticity as the mechanism, and are more likely to be provoked by catecholamine administration than by programmed stimulation [261, 824, 825]. Postinfarction VAs from the papillary muscle can arise from an area of scar tissue and can have a reentrant mechanism [826]. Preprocedural imaging including CMR is helpful for identifying areas of scar tissue or fibrosis that are potential sources of arrhythmia.

Although most idiopathic PVCs from the papillary muscles are benign, they have the potential to cause cardiomyopathy [697, 827], and rarely they are triggers for VF [828]. Patients with frequent papillary muscle arrhythmias are also more likely to have mitral valve prolapse [697, 829]. While the large majority of these patients have benign outcomes, sudden death can occasionally occur, and this condition has been associated with the female sex, bileaflet prolapse, complex VAs, inverted or biphasic T waves in the inferior ECG leads of sinus beats, and LGE-CMR, typically in the inferior LV [830, 831]. Further studies on risk stratification are therefore needed.

Papillary muscle arrhythmias can arise from either ventricle, but the LV is the most common origin, with the posteromedial papillary muscle being the most frequent [832]. Papillary muscle arrhythmias originate more frequently toward the tip of the muscle, in its distal third [696]. QRS morphology can suggest the origin, but variable papillary muscle anatomy and exit sites into the myocardium limit ECG accuracy. PVCs from the posteromedial papillary muscles have RBBB QRS morphology with a superior axis and a transition in leads V3–V5. PVCs from the anterolateral papillary muscles have RBBB QRS morphology, a rightward axis, and inferior leads, demonstrating an inferior or discordant axis [267, 825]. A small initial q wave in V1 is common. Papillary muscle PVCs have a longer QRS duration compared with fascicular PVCs (153 ± 27 ms vs 127 ± 25 ms, *P* < .05) [267], and fascicular PVCs frequently exhibit an rsR′ morphology in V_1_, which is more consistent with a typical RBBB morphology.

Papillary muscle VAs in the same patient often exhibit variability in QRS morphology, which can be due to variable exits from the same origin [262]. Pacing can produce the same QRS over a relatively large area on the papillary muscle, and ablation at a site with a pace map identical to the VA might not eliminate the arrhythmia, leading to ablation over larger areas [262]. In some patients, the VA can originate in a deep intramural location, and the electrograms with the earliest activation time can have a far-field appearance or be difficult to define. In these patients, ablation at sites with a matching pace map and prolonged S-QRS might be successful, although longer and higher-powered RF application might be necessary [833].

**Fig. 8:**
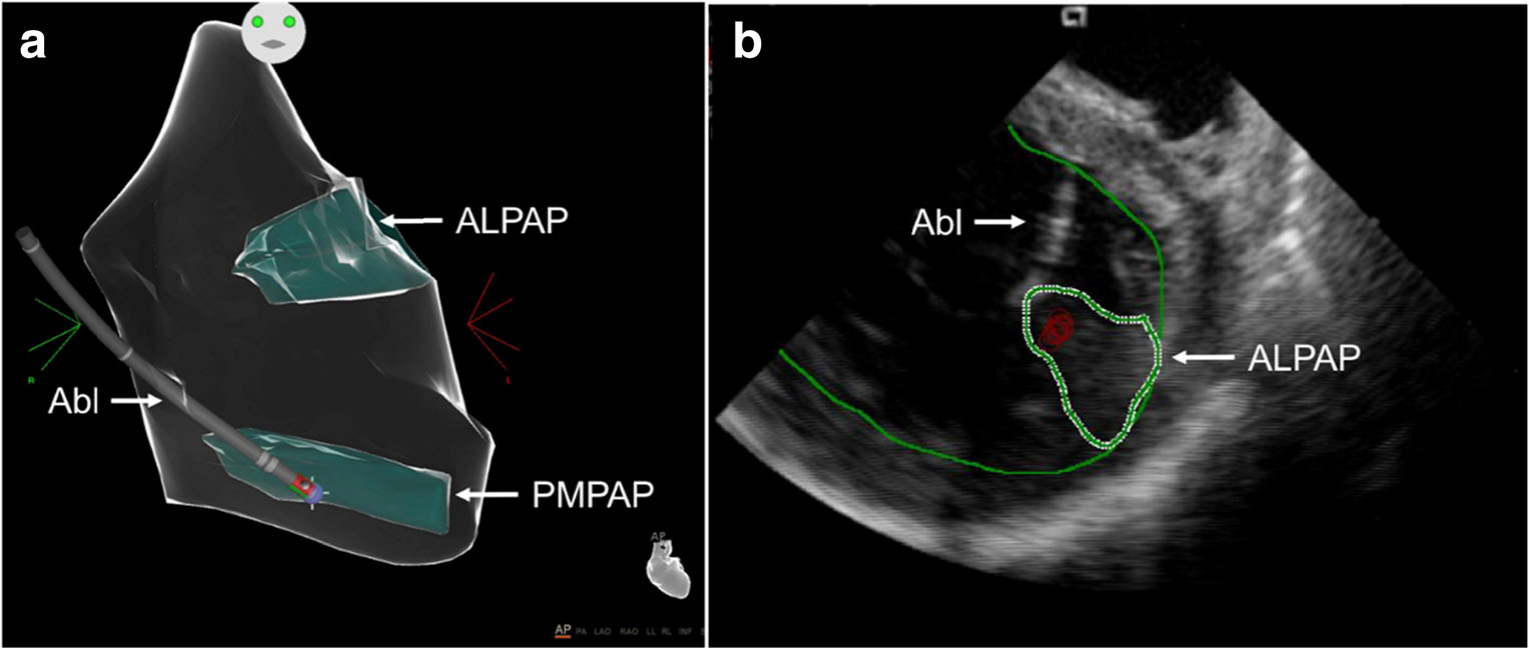
Intraprocedural imaging during ablation of papillary muscle arrhythmias. (A): Anatomical map of the left ventricle (CARTO, Biosense Webster) showing contact of the ablation catheter (Abl) with the posteromedial papillary muscle (PMPAP). (B): Intracardiac echocardiogram showing real-time visualization of the ablation catheter during ablation on the anterolateral papillary muscle (ALPAP)

Defining the anatomy and maintaining consistent catheter contact and stability during ablation delivery are significant challenges. The use of ICE integrated with EAM systems can help to identify papillary muscle surfaces within the ventricle and provide real-time visualization of catheter contact on the papillary muscle during ablation (Fig. 8) [265, 825, 832]. Catheter instability often induces PVCs that complicate mapping. Steerable sheaths can be useful when a transseptal approach is employed, although there is a lack of comparative data. Cryoablation has the advantage of achieving stable contact with the papillary muscle during freezing and can be effective; however, data are limited [694].

Lower success rates and higher recurrence rates have been reported for the catheter ablation of papillary muscle arrhythmias, compared with other idiopathic VAs, with one study citing a 60% long-term success rate for ablation in patients with idiopathic papillary muscle PVCs [237]. Although worsening mitral regurgitation following ablation of the papillary muscles has been reported, this is a rare occurrence, and complications from ablation of papillary muscle arrhythmias are generally not significantly different from ablation at other LV locations [825, 834, 835].

### Bundle branch reentrant ventricular tachycardia and fascicular ventricular tachycardia

**Key Points**

• BBR can occur in a variety of patients in whom the conduction system can be affected, including patients with DCM, valvular heart disease, MI, myotonic dystrophy, Brugada syndrome, and ARVC, among others.

• Ablation of either the RBB or LBB eliminates BBRVT but does not eliminate other arrhythmic substrates.

• A correct diagnosis of BBRVT is crucial and should employ established criteria prior to ablation of either of the bundle branches.

• Ablation of the AV node does not cure BBRVT.

• Ablation of either bundle branch does not cure interfascicular VT.

• For posterior fascicular VTs, the P1 potential is targeted during VT; if P1 cannot be identified or VT is not tolerated, an anatomical approach can be used.

• Purkinje fibers can extend to the papillary muscles, and these can be part of the VT circuit.

• For anterior fascicular VTs, the P1 potential is targeted with ablation.

• Focal nonreentrant fascicular VT is infrequent and can occur in patients with IHD; however, it cannot be induced with programmed stimulation, and the target is the earliest Purkinje potential during VT.

#### Introduction

There are 4 distinct groups of Purkinje-related monomorphic VTs: 1) BBR including interfascicular reentry VTs; 2) idiopathic fascicular reentrant VT; 3) fascicular reentrant VT post infarction; and 4) focal nonreentrant fascicular VT. The mechanism of focal nonreentrant fascicular VT is abnormal automaticity from the distal Purkinje system, and the ablation target is the earliest Purkinje activation during the VT. The other VTs are caused by macroreentry and usually occur in specific locations with specific QRS morphologies.

#### Bundle branch reentrant ventricular tachycardia

##### 9.4.2.1. Recognition

BBRVT is a unique, typically fast (200–300 bpm), monomorphic tachycardia, which is usually associated with hemodynamic collapse, syncope, and/or cardiac arrest [836]. BBRVT is caused by a macroreentry circuit involving the RBB and LBB, and septal ventricular muscle [364, 836–839]. BBR is often associated with advanced heart disease and has been observed in DCM [364], coronary artery disease [364], valvular heart disease [114, 364, 840], muscular dystrophy [841–843], CHD [844], LV noncompaction (LVNC) [845], ARVC [846], and Brugada syndrome [847]. BBRVT can also occur in association with His-Purkinje system disease in the absence of SHD [370, 848, 849]. Although classically associated with DCM, BBRVT can also occur in the setting of isolated, unexplained conduction system disease [370, 850, 851]. Recently, 6 cases of idiopathic BBRVT were identified in patients with normal biventricular size and function. All patients were noted to have His-Purkinje system disease, with prolonged HV at baseline (mean 69.2 ms), and underwent successful ablation targeting the RBB. Mutations were identified in 3 of the 6 patients: 2 in the *SCN5A* gene and 1 in the *LMNA* gene [851]. Idiopathic BBRVT can be a genetic conduction disease, manifested by isolated conduction system disease and curable with catheter ablation.

The reported incidence of BBRVT is 3.5%, representing 6% of VTs in separate series and 20% in a series of patients with NICM alone undergoing evaluation for ablation [131, 348, 852]. The incidence of BBRVT is likely underestimated.

BBRVT can have a typical LBBB [853] or RBBB pattern, depending on which bundle forms the anterograde limb (Table 5); however, both morphologies can occur in the same patient [854]. Tchou and Mehdirad [855] described 3 categories of BBRVT (Table 5). Type A and type C are the classic counterclockwise and clockwise BBRVT circuits, respectively. Type B is reentry within the LBB fascicles (interfascicular reentry). Counterclockwise BBR (type A) accounts for 98% of BBRVTs and has a typical LBBB pattern with an R-wave transition between leads V_4_ and V_5_ [856]. Alternatively, activation can proceed in an anterograde manner via the LBB and then through the septum, with retrograde activation via the RBB, creating an RBBB morphology (“clockwise” reentry, type C) [131, 857]. Interfascicular reentry tachycardia often has an RBBB and left hemiblock QRS pattern.

**Table 5:** Types of bundle branch reentrant tachycardia

	Type A	Type B (Interfascicular tachycardia)	Type C
ECG morphology	LBBB pattern	RBBB pattern	RBBB pattern
Anterograde limb	RBB	LAF or LPF	LBB
Retrograde limb	LBB	LPF or LAF	RBB

*LAF* left anterior fascicle; *LBB* left bundle branch; *LBBB* left bundle branch block; *LPF* left posterior fascicle; *RBB* right bundle branch; *RBBB* right bundle branch block

Patients with BBR generally have a prolonged PR interval, QRS duration, and H-V interval. LBBB, or less commonly RBBB, on the sinus rhythm ECG, is due to conduction slowing in the bundle rather than complete block.

Single BBR beats can occur in patients who have apparently normal His-Purkinje conduction during sinus rhythm but who have functional conduction impairment [368]. This condition is observed as a normal response to premature ventricular extrastimuli in patients undergoing PES and has been called a V3 phenomenon [837, 858].

In the electrophysiology laboratory, ventricular extrastimuli delivered with short–long–short coupling intervals (pacing train of 400 ms with a delay of 600 or 800 ms before the short-coupled premature coupling intervals are introduced) tend to cause unidirectional block in the bundle branches facilitating the initiation of BBR [859]. Atrial pacing and/or isoproterenol might be required [850].

##### 9.4.2.2. Specific considerations

- Several criteria help in diagnosing BBRVT:
  1. The typical 12-lead ECG morphology during tachycardia is LBBB or RBBB.
  2. A critical delay in His-Purkinje system conduction is needed to initiate tachycardia.
  3. Although AV dissociation can be present, there is usually persistent 1:1 His-bundle or QRS activation, with the H-V interval during the tachycardia equal to or longer than the H-V interval in sinus rhythm (and no H-V dissociation) [860].
  4. Tachycardia stops and cannot be reinitiated if conduction in one of the bundle branches is interrupted.
  5. During BBR, the His-bundle, RBB, and LBB activation sequences during an LBBB morphology tachycardia (or the His-bundle, LBB, and RBB activation sequences during an RBBB morphology tachycardia) remain stable.
  6. If the CL fluctuates, then the H-H interval oscillations precede the V-V interval oscillations. The H-V timing remains constant.
  7. Entrainment with constant fusion (manifest entrainment) is present during pacing from the RV apex [861]. However, it results in a PPI within 30 ms of the tachycardia cycle [862].
  8. Entrainment with concealed fusion is present during atrial pacing faster than the tachycardia, provided that AV nodal conduction allows 1:1 conduction.

In type A and C BBRVTs, the onset of ventricular depolarization is preceded by His-bundle, RBB, or LBB potentials with an appropriate sequence of His-bundle>RBB > LBB activation. Spontaneous variations in V-V intervals are preceded by similar changes in H-H/RBB-RBB/LBB-LBB intervals. Recording from both sides of the septum can help to identify the BBR mechanism.

Interfascicular tachycardia is an uncommon type [105, 132, 364, 850, 863, 864] in which one of the fascicles serves as the anterograde limb while the other serves as the retrograde circuit. The distal link between fascicles occurs through the ventricular myocardium. The LAF is usually the anterograde limb and the LPF is the retrograde limb [105, 848]. BBRVT and interfascicular tachycardia can be present in the same patient [363, 837, 863]. In contrast to BBR, the H-V interval during interfascicular tachycardia is usually shorter by more than 40 ms than that recorded in sinus rhythm [865]. This condition occurs because the upper turnaround point of the circuit (the LBB point) is relatively far from the His bundle activated in the retrograde direction.

It is important to recognize BBRVT and interfascicular tachycardia because they can be cured with catheter ablation. The differential diagnosis for BBR includes 1) VT due to myocardial reentry, 2) idiopathic left intrafascicular VT, 3) SVT with aberrant conduction, and 4) atriofascicular reentry.

With myocardial reentry, the His potential usually does not precede the QRS complex, and variations in the H-H interval follow changes in the V-V interval because there is retrograde passive activation of the His-Purkinje system. The PPI after entrainment of tachycardia from the RV apex will usually be long, unless the myocardial reentrant VT involves the apex [364, 862].

The possibilities of other Purkinje-related VTs [866], such as idiopathic fascicular VT [867], fascicular VT post-MI [106, 392, 868], automatic His-Purkinje VT, focal Purkinje VT [389, 869], and AV node/His-Purkinje reentry [870], should also be considered (see below).

##### 9.4.2.3. Catheter ablation

Pharmacological antiarrhythmic therapy is usually ineffective; however, RF catheter ablation of a bundle branch can cure BBRVTs and is currently regarded as first-line therapy [131, 364, 871]. Given that significant underlying structural disease is usually present, concomitant placement of an ICD should be strongly considered.

The technique of choice is ablation of the RBB [363–365]. BBRVT can be prevented by ablating the right or left main bundle branch [848, 855]. Although most patients demonstrate conduction system disease in the LBB, the RBB is typically the target for ablation because of the technical ease and desire to avoid complete LBBB. In patients with a complete LBBB pattern during sinus rhythm, anterograde ventricular activation occurs solely via the RBB due to slow anterograde conduction over the LBB, potentially with retrograde LBB activation due to transseptal concealed conduction [369, 848]. Complete AV block can develop with RBB ablation, and the LBB may be targeted in such patients [369, 848].

Complete RBB or LBB develops with successful ablation, although QRS changes can be subtle in patients with preexisting conduction abnormalities. Elimination of retrograde V-H conduction has been used as a marker of successful ablation.

In interfascicular reentry, RBB ablation will not cure the tachycardia because the RBB is a bystander. Similarly, ablation of the main LBB would not be expected to terminate the tachycardia because the circuit is distal to this point. Catheter ablation of the LAF or LPF will result in termination of the tachycardia [105, 363, 864]. A change of electrical axis might be the only manifestation during sinus rhythm after fascicular ablation.

##### 9.4.2.4. Outcomes and risks

In the 2 largest reported series, acute success rates for BBRVT and interfascicular reentry were 100% [364, 366]. After ablation, BBRVT recurrence is uncommon but has not been thoroughly assessed with follow-up testing [105, 364, 366].

The reported incidence of clinically significant conduction system impairment requiring implantation of a permanent pacemaker varies from 0% to 30% [364, 840, 841, 858–860, 865].

Despite the success of BBRVT ablation, patients with cardiomyopathy and heart failure continue to have a high mortality rate. Despite the impressive success of ablation for BBRVT, progressive heart failure is a common cause of death [131, 363–365, 368, 840, 856, 871]. Therefore, a CRT defibrillator should be considered after ablation based on the status of the residual conduction system and the severity of the underlying SHD. Furthermore, VT of myocardial origin can be induced in 36% to 60% of patients after successful ablation for BBR [105, 856].

#### Idiopathic fascicular reentrant ventricular tachycardia

##### 9.4.3.1. Recognition

Verapamil-sensitive fascicular reentrant VT is the most common form of idiopathic left-sided VT and was first recognized as an electrocardiographic entity in 1979 by Zipes et al. [872], who identified the following characteristic diagnostic triad: 1) induction with atrial pacing, 2) RBBB and left-axis configuration, and 3) manifestation in patients without SHD. In 1981, Belhassen et al. [873] were the first to demonstrate the verapamil sensitivity of the tachycardia, a fourth identifying feature. In 1988, Ohe et al. [167] reported another type of this tachycardia, with RBBB and a right-axis configuration. Nogami [867] and Talib et al. [874] subsequently reported on upper septal fascicular tachycardia. According to the QRS morphology, verapamil-sensitive left fascicular reentrant VT can be divided into 3 subgroups [867]: 1) left posterior fascicular reentrant VT, in which the QRS morphology exhibits a RBBB configuration and a superior axis; 2) left anterior fascicular reentrant VT, in which the QRS morphology exhibits an RBBB configuration and inferior axis; and 3) upper septal fascicular VT, in which the QRS morphology exhibits a narrow QRS configuration and normal or right-axis deviation [867, 874, 875]. The left posterior type is most common, the left anterior type is uncommon, and left upper fascicular VT is very rare but sometimes occurs after catheter ablation of other fascicular reentrant VTs.

The reentrant circuit of verapamil-sensitive fascicular reentrant VT can involve the Purkinje network around the papillary muscles [876]. In addition to the current classification with 3 subtypes, papillary muscle fascicular VT is another identifiable verapamil-sensitive fascicular reentrant VT. Finally, verapamil-sensitive left fascicular reentrant VT can be classified into 5 subgroups (Table 6). Papillary muscle VTs originating from the Purkinje fiber system and VT originating from myocardial tissue of the papillary muscle appear to be different entities, although there can be some overlap.

**Table 6:** Fascicular ventricular tachycardias

I. Verapamil-sensitive fascicular reentrant VT	
1. Left posterior type	
i. Left posterior septal fascicular reentrant VT	
ii. Left posterior papillary muscle fascicular reentrant VT	
2. Left anterior type	
i. Left anterior septal fascicular reentrant VT	
ii. Left anterior papillary muscle fascicular reentrant VT	
3. Upper septal type	
II. Nonreentrant fascicular VT	

*VT* ventricular tachycardia

##### 9.4.3.2. Specific considerations

The anatomical basis of this tachycardia has provoked considerable interest, with data suggesting that the tachycardia can originate from a false tendon or fibromuscular band in the LV [877–880]. Using ICE and 3D mapping systems, the successful ablation site appears to be at the connection of a false tendon and ventricular wall in some cases. The Purkinje networks in these small anatomical structures can be important parts of the circuit. In papillary muscle fascicular VTs, fibromuscular bands near papillary muscles can potentially be the substrate of the circuit.

During tachycardia, 2 distinct groups of potentials, P1 and P2, are typically recorded from a catheter lying along the midseptum [378, 881, 882]. The mid-diastolic potentials (P1) show proximal to distal activation along the septum, and the fused presystolic Purkinje potential (P2) shows distal to proximal activation. During sinus rhythm, the P2 potentials are later than the His-bundle potential and earlier than the onset of the QRS, which is consistent with an origin in the LPF. P1 represents the activation from tissue that has decremental properties and verapamil sensitivity that constitutes the anterograde limb of the circuit in VT. P2 represents the activation of the LPF or a Purkinje fiber near the LPF and is a bystander during VT. The LV septal muscle is likely the retrograde limb.

##### 9.4.3.3. Catheter ablation

VT can be initiated with atrial extrastimuli or burst pacing, or ventricular extrastimuli or burst pacing, facilitated by an isoproterenol infusion, if necessary. When evaluating these tachycardias, a multipolar catheter placed along the left septum can be helpful in delineating parts of the reentry circuits [378, 881, 882].

Left posterior septal fascicular reentrant VT mapping along the LV septum identifies 2 distinct groups of potentials (P1 and P2) during the VT [867, 881]. The apical third of the septum with a P1 potential is usually targeted to avoid creating LBBB or AV block. If the VT or ventricular echo beats are not inducible, an empirical anatomical approach can be effective [378]. First, the VT exit site is determined by pace mapping during sinus rhythm, and RF energy is delivered to that site. Second, a linear lesion is placed at the midseptum, perpendicular to the long axis of the LV, approximately 10–15 mm proximal to the VT exit. This anatomical approach is also useful in patients in whom diastolic Purkinje potentials cannot be recorded during VT.

Reported cases of left posterior papillary muscle fascicular VT presented an RBBB configuration and right-axis deviation QRS configuration. A diastolic Purkinje potential (P1) is recorded at the papillary muscle with the location confirmed by ICE imaging. Ablation at this site is highly effective for suppressing this VT [876].

Left anterior septal fascicular VT exhibits an RBBB QRS morphology with Rs pattern in V5–V6. A Purkinje potential is recorded in the diastolic phase during the VT at the midanterior LV septum [882]. In this circuit, P1 potentials represent activation in the proximal portion of the specialized Purkinje tissue, which has decremental conduction properties. During VT, the anterograde limb is the Purkinje tissue exhibiting P1, and the retrograde limb is the LV muscle. The circuits of the left anterior and posterior fascicular reentrant VTs are mirror images.

Left anterior papillary muscle fascicular VTs have RBBB with deep S waves in V5–V6. LV endocardial mapping during left anterior fascicular reentrant VT identifies the earliest ventricular activation in the anterolateral wall of the LV, where ablation suppresses VT [876, 882].

Upper septal fascicular VT has a narrow QRS and inferiorly directed frontal plane axis. P1 represents the activation potential of the specialized Purkinje tissue at the LV septum [874]. P2 represents the activation of the left anterior and posterior fascicles. Both the left anterior and posterior fascicles are the anterograde limbs of the reentrant circuit in VT, producing the narrow QRS configuration and inferior axis. This VT is successfully ablated at the LV midseptum.

##### 9.4.3.4. Outcomes and risks

In the largest series of reentrant fascicular VTs, which included 160 patients with left posterior septal fascicular VT, 30 patients with left anterior septal fascicular VT, and 8 patients with left upper septal fascicular VT [883], the success and recurrence rates were 97% and 4%, respectively, for left posterior septal fascicular VT; 90% and 11%, respectively, for left anterior septal fascicular VT; and 100% and 2.5%, respectively, for left upper septal fascicular VT. Recurrence of papillary muscle fascicular VT after ablation was high [876], with 3 of 8 patients (38%) with posterior papillary muscle fascicular VT and 1 of 5 patients (20%) with anterior papillary muscle fascicular VT requiring a second ablation session for VT recurrences.

Aside from the complications that can result from any LV electrophysiological procedure, the only complications specifically associated with catheter ablation of idiopathic left fascicular VT have been LBBB and AV block. Tsuchiya et al. [380] have reported that 2 patients in their series of 16 patients (12.5%) had transient LBBB after ablation. The authors targeted the left basal septum, and the LBBB disappeared within 10 min without VT recurrence. In a larger series [883], 1 (0.5%) of 198 patients had a transient AV block. This patient presented with left posterior fascicular VT, and the diastolic potential (P1) at the midseptum was targeted for ablation. Before the ablation, the patient had catheter-induced RBBB. The AV block disappeared immediately after discontinuing the RF energy delivery.

#### Focal nonreentrant fascicular ventricular tachycardia and premature ventricular complex

##### 9.4.4.1. Recognition

Focal nonreentrant fascicular VT is classified as propranolol-sensitive automatic VT [389, 884] and is usually observed in patients with IHD [105], although it has been observed in patients with structurally normal hearts [390, 885]. VT can be induced by exercise and catecholamines (eg, isoproterenol and phenylephrine); however, it cannot be induced or terminated by PES [389]. VT is transiently suppressed by adenosine and with overdrive pacing. Although this VT is responsive to lidocaine and beta blockers, it is usually not responsive to verapamil. These features can be employed to distinguish it from reentrant fascicular VT. The clinical and electrophysiological characteristics of this VT have not yet been well defined. Gonzalez et al. [390] have reported the electrophysiological spectrum of Purkinje-related monomorphic VT in 8 patients and have shown the mechanism to be consistent with abnormal automaticity or triggered activity in 5 patients. Talib et al. [389] have reported on 11 patients (2.8%) with idiopathic nonreentrant fascicular VT among 530 patients with idiopathic VT without SHD. All patients had monomorphic VT with a relatively narrow QRS (123 ± 12 ms) and did not respond to verapamil. The VT exhibited RBBB and superior-axis configuration in 11 patients (73%), inferior axis in 3 (20%), and LBBB and superior-axis configuration in 1 (7%). The VT could not be entrained.

##### 9.4.4.2. Catheter ablation

The ablation target of nonreentrant fascicular VT is the earliest Purkinje activation during VT. If the VT was not induced by ventricular stimulation and catecholamines, isolated PVCs with a similar QRS morphology to that observed during the VT can be targeted. At the earliest Purkinje activation during VT/PVC, a Purkinje potential is also recorded during sinus rhythm [389].

##### 9.4.4.3. Outcomes and risks

Although VT and PVC had been suppressed by catheter ablation in 1 series, the acute success rate is unclear because this VT is difficult to induce [389]. VT recurrence was observed in 4 patients (27%), 3 of whom underwent pace map–guided ablation during the first session. A second ablation with activation mapping guidance eliminated the VT during the 88 ± 8–month follow-up. Catheter ablation guided by activation mapping appears effective, whereas the pace map–guided approach is less efficacious.

LBBB and AV block have been associated with catheter ablation of nonreentrant fascicular VT. In verapamil-sensitive reentrant fascicular VTs, the creation of LBBB or AV block is rare because the ablation target is the diastolic abnormal Purkinje potential (P1) during VT, and the abolition of the normal Purkinje or fascicle potential (P2) is not needed to suppress the VT. By contrast, abolition of a portion of the Purkinje network is usually necessary to suppress the nonreentrant fascicular VT. After successful ablation, the amplitude of the local myocardium is diminished, and the Purkinje potential appears after the myocardial potential. When the ablation site is located at the distal portion of the left fascicle, there is no change in the surface QRS morphology or H-V interval after ablation. If the VT arises from a more proximal portion of the fascicle, there is a potential risk for causing LBBB or AV block by the ablation. Rodriguez et al. [885] have reported cases of nonreentrant fascicular VT with an RBBB configuration and right-axis deviation in patients who presented left anterior fascicular block after the ablation. Lopera et al. [105] have reported on 2 patients with nonreentrant fascicular VT and IHD in whom complete AV block occurred after successful ablation for VT.

### Postinfarction ventricular tachycardia

**Key Points**

• In cases of multiple inducible VTs, the clinical VT should be preferentially targeted.

• Elimination of all inducible VTs reduces VT recurrence and is associated with prolonged arrhythmia-free survival.

• For tolerated VTs, entrainment mapping allows for focal ablation of the critical isthmus.

• For nontolerated VTs, various ablation strategies have been described, including targeting abnormal potentials, matching pace mapping sites, areas of slow conduction, linear lesions, and scar homogenization.

• Imaging can be beneficial in identifying the arrhythmogenic substrate.

• Epicardial ablation is infrequently required, but epicardial substrate is an important reason for VT recurrence after VT ablation in patients with prior infarcts.

#### General considerations

The mechanism for most VTs after MI is macroreentry involving ventricular scar areas, and initial events commonly occur several years after the acute MI [578, 886–888]. Focal nonreentrant mechanisms have been described in up to 9% of cases [889]. The chronic scar pattern observed after coronary occlusion has been most widely described as compact in architecture, extending from the subendocardium to the epicardium, with increasing ischemia duration, typically sparing the endocardial rim close to the cavity. Along the border of the scar, viable myocardium is interspersed with fibrous tissue, providing the substrate for slow conduction [122, 125, 886, 887]. Although most reentry circuits appear to involve the subendocardium, intraoperative mapping studies have demonstrated that a substantial number of VT circuits involve or are confined to the subepicardial layer [578].

Early reperfusion therapy, which has been increasingly adopted for effective management of acute MI, has not only resulted in myocardial salvage but also less LV aneurysm formation, scar transmurality, and wall thinning. Less confluent histological and electroanatomical scars [890] are associated with the induction of faster VTs [890, 891] and pose additional challenges for mapping and ablation for post-MI VT.

#### Clinical, unknown clinical, and nonclinical ventricular tachycardia

The first goal of VT ablation is to abolish the clinical VT, which can be accomplished in 90%–100% of patients inducible at baseline [330, 619, 629, 634]. However, determining which VT is clinical can often be challenging. Although a 12-lead VT ECG is not available for most patients, the stored ICD electrograms, if available, can act as an accurate surrogate for the 12-lead ECG [628]. CL of both spontaneous and induced VTs can vary, and CL alone is often insufficient for recognizing clinical VT [628]. Adding to the challenge of determining the area of interest and the endpoint for ablation is the lack of up-front inducibility of any VT in 7%–24% of patients [629–631] and specifically that of clinical VT in 13%–30% of patients [628, 629, 632]. It is important to note that most patients are inducible for multiple previously undocumented VTs, with separate circuits and variable exit sites from the scar [70, 71, 309, 629]. The clinical relevance of VTs induced in the electrophysiology laboratory without prior documentation is unclear; numerous laboratories have only targeted induced VTs with CL equal to or longer than that of clinically observed VT(s) but not those with comparatively shorter CLs [71, 309]. In these multicenter trials, the VT rate could be markedly reduced in a substantial number of patients; however, approximately 50% of patients experienced VT recurrence within 6 months [70, 71, 309].

The association between noninducibility following ablation for any VT, including nonclinical VTs and the subsequent greater freedom from VT recurrence, supports the clinical relevance of nonclinical VTs. However, as many as 41% of the patients who remain inducible for nonclinical VT do not present with recurrence during short-term follow-up (see Section 9.17) [629, 633]. A potentially useful electrophysiological characteristic in distinguishing relevant from nonrelevant induced VTs is the similarity between VT CL and the baseline ventricular refractory period, given that VTs with a CL within 30 ms of the ventricular refractory period CL (fast VT based on the individual ventricular refractory period) rarely occur spontaneously [892]. The presence of only the latter VTs has been associated with low VT recurrence, comparable to that of patients who were rendered noninducible by ablation [892].

#### Mapping and ablation strategy

**Fig. 9:**
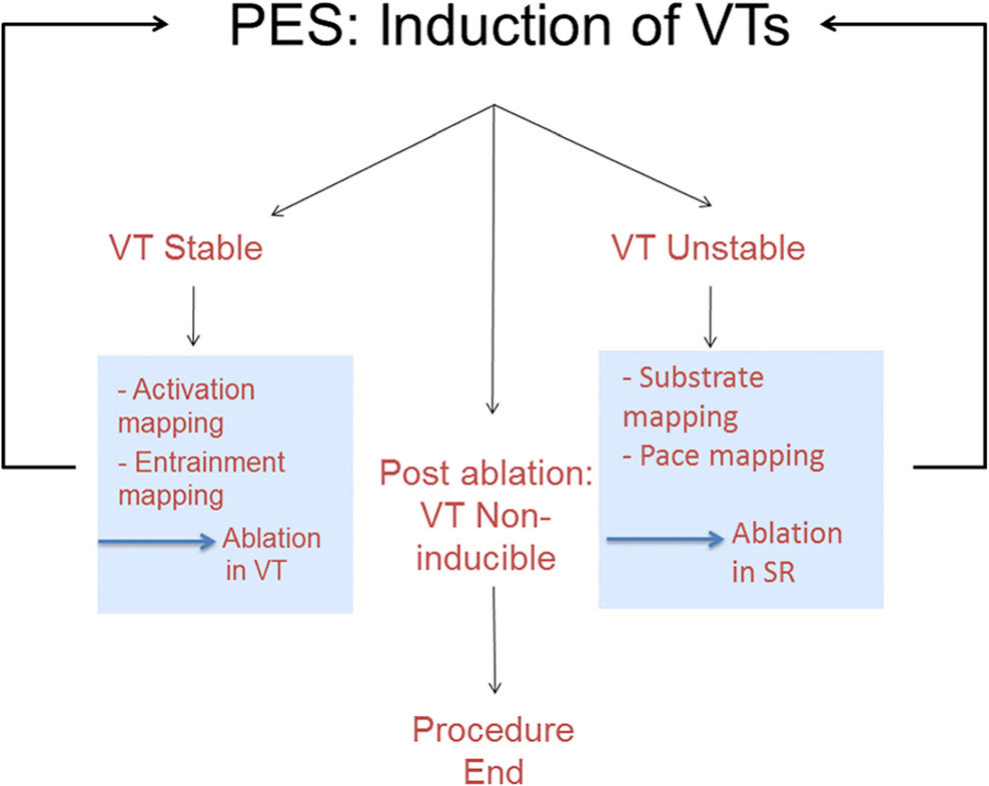
Overview of the workflow for catheter ablation of VT in patients with IHD. Not all of these steps might be required, and steps can be performed in a different sequence. For instance, repeat VT induction can be deferred in patients with hemodynamic instability. In addition, the operator might have to adapt to events that arise during the case, for instance, to take advantage of spontaneous initiation of stable VT during substrate mapping and switch to activation mapping. IHD = ischemic heart disease; PES = programmed electrical stimulation; SR = sinus rhythm; VT = ventricular tachycardia

Mapping and ablation strategies are determined by the type of VT and substrate. After MI, a minority of patients have only mappable VTs (hemodynamically tolerated, reproducibly inducible, with stable morphology) that allow for extended activation and entrainment mapping [71, 309, 629]. Hence, for most patients, additional mapping methods need to be employed in conjunction with entrainment mapping to identify critical components of the reentry circuit. Although there is no single gold standard approach, a reasonable workflow is presented in Fig. 9.

Voltage mapping facilitated by 3D EAM systems (Section 8) is considered the standard for invasive identification of scar tissue during sinus rhythm and has been validated by histology in postinfarct animal models [36, 728, 893, 894]. For electroanatomically confluent and dense scars typical of nonreperfused infarcts [890], VT-related sites are often but not exclusively located in low-voltage areas [583, 895]. To provide additional guidance in identifying putative isthmus sites in low-voltage areas, especially for unmappable VTs, several additional strategies have been suggested focusing on electrogram characteristics (split, late, fractionated potentials) and pace mapping [36, 71, 72, 309, 316, 728]. Mapping within regions of low voltage while pacing with extrastimuli at sites remote from low-voltage regions has also been demonstrated to be useful for exposing abnormal conduction regions that can be critical for VT [47, 127, 685, 686].

Various RF lesion delivery strategies have been described, ranging from focal ablations guided by entrainment mapping to ablation of the entire scar [71, 619]. Suggested strategies include short linear lesions from the putative VT exit sites toward the center of the substrate, across the presumed critical isthmus and/or along the scar border zone [36, 316, 728]. However, the endpoint for these short linear lesions is often arbitrary. Circumferential isolation of the identified scar (bipolar voltage <1.5 mV) [688] or the “core” containing potential VT isthmus elements (regions with bipolar voltage <0.5–1 mV and potentially important sites identified by entrainment, activation, and pace mapping) [50], confirmed by demonstrating exit block from the core, has been suggested as an incrementally useful ablation endpoint beyond VT noninducibility. Other methods incorporate the goal of demonstrating functional entrance block into regions of scar tissue, as demonstrated by the elimination of all previously identified LPs or local abnormal ventricular activities, either by targeting channels of conduction or by direct ablation at all abnormal sites [38, 46, 619, 687]. These approaches often involve more comprehensive substrate modification than the initial strategy of transecting potential circuits with linear lesion sets.

Although substrate-based ablation for clinical and induced VTs remains a probabilistic approach to VT ablation, the technique has been successfully performed after a presumed first VT episode and in patients with recurrent VT despite AAD therapy [71, 72, 316, 317].

After substrate modification guided by clinical and induced nonclinical VT, a significant number of patients experience recurrence of symptomatic VT, including 30% of patients who have been rendered noninducible after ablation [633]. The number of induced VTs is a potential surrogate for a larger and complex substrate [71, 629], and larger scars have been associated with VT recurrence [629]. Recurrent VT can be due to lesion recovery or incomplete lesions with local modification of the substrate [631, 896]. Although some of these VTs originate from a previously targeted area, they can have a different morphology based on 12-lead ECGs or ICD electrograms [631, 896]. However, recurrent VT can also originate from unmapped areas or from those areas not considered relevant during the index ablation [896].

#### Substrate-based ablation strategies without upfront ventricular tachycardia induction

Substrate-based approaches that target some or all abnormal electrograms, irrespective of clinical and/or induced VTs, have been suggested (Table 7), which can also be performed if VT is not inducible or if VT induction is not desired because of safety concerns [46, 47, 619, 630]. These strategies have targeted sites with abnormal electrograms, either of the entire scar in the endocardium and epicardium [619] or, more selectively, target sites with LPs [630] anywhere within the scar or sites with fractionated potentials predominantly located in the border zone, which are markers for conducting channels [46], or that have targeted sites with abnormal potentials that are poorly coupled to the myocardium (ie, LAVA) [47]. These techniques have been demonstrated to be superior to those employed in the control groups in the previously cited studies. There is, however, no generally accepted control group, and it is therefore difficult to compare the benefit of the various approaches with each other.

**Table 7:** Select recent radiofrequency catheter ablation studies in patients post myocardial infarction with a focus on substrate-based ablation strategies

Study	N	EF (%)	Prior CABG (%)	Inclusion	Access mapping catheter	Mapping strategy	Ablation strategy	Procedural endpoint	RF time procedural duration complications	VT recurrence and burden (follow-up)
Jais et al. (2012) [47] Two centers observational	70	35 ± 10	NR	1) Sustained VT resistant to AAD therapy and requiring external cardioversion or ICD therapies 2) SHD with ischemic or nonischemic dilated cardiomyopathy Exclusions: 1) VA attributable to an acute or reversible cause 2) Repetitive PVCs or nonsustained VT without sustained VT	Retrograde in 61 pts. (87%) Transseptal in 32 pts. (46%); epicardial access in 21 pts. (31%) Dual access encouraged 3.5-mm external irrigated ablation catheter; multielectrode mapping catheter in 50% endocardial procedures and in all epicardial procedures	1) PES and activation mapping of induced stable VTs 2) Substrate mapping for LAVAs — sharp high-frequency electrograms often of low amplitude, occurring during or after the far-field ventricular electrogram, sometimes fractionated or multicomponent, poorly coupled to the rest of the myocardium	1) Ablation of LAVA in SR 2) Ablation of tolerated VTs guided by entrainment and activation mapping 3) Remapping (in stable patients) with further ablation if residual LAVA or persistent inducibility	1) Complete LAVA elimination — achieved in 47 of 67 pts. with LAVA (70.1%) 2) Noninducibility — achieved in 70%, similar if LAVA eliminated or not	RF time 23 ± 11 min Procedure time 148 ± 73 min Complications 6 pts. (8.6%): tamponade or bleeding managed conservatively (3), RV perforation requiring surgical repair (1); 3 pts. died within 24 h due to low-flow state (2) plus arrhythmia recurrence (1), PEA (1)	Combined endpoint of VT recurrence or death occurred in 39 pts. (55.7%); 45% of pts. with LAVA elimination and 80% of those without VT recurrence in 32 (46%); 32% of pts. with LAVA elimination and 75% of those without 7 cardiac deaths (10%) over 22 months of median follow-up
Di Biase et al. (2015) [619] VISTA trial Multicenter RCT	118	Group 1 33 ± 14 Group 2 32 ± 10	34%	1) Post-MI 2) Recurrent stable AAD refractory VT (symptomatic or requiring ICD therapy) Exclusion: syncope, cardiac arrest, prior failed ablation, renal failure, end-stage heart failure	Endocardial Epicardial when clinical VTs were inducible after endocardial ablation + no CABG Group 1: 11.7% Group 2: 10.3% 3.5-mm tip	1) Substrate mapping (BV ≤1.5 mV) + Group 1 2) PES and activation mapping/pace mapping for clinical and stable nonclinical VT (unstable VT not targeted)	Group 1: Clinical VT ablation, linear lesion to transect VT isthmus Group 2: Extensive substrate ablation targeting any abnormal potential (=fractionated and/or LP)	Group 1: Noninducibility of clinical VT — achieved in 100% Group 2: 1) Elimination of abnormal potentials 2) No capture from within the scar (20 mA) 3) Noninducibility of clinical VT — achieved in 100%	Group 1: RF time 35 ± 27 min Procedural time 4.6 ± 1.6 h Group 2: RF time 68 ± 27 min (*P* < .001) Procedural time 24.2 ± 1.3 h (*P* = .13) Complications 5%	VT recurrence at 12 months Group 1: 48.3% Group 2: 15.5% *P* < .001 Mortality at 12 months Group 1: 15% Group 2: 8.6% *P* = .21
Tilz et al. (2014) [688] Single center observational	12 12/117 pts. with post-MI VT	32 ± 13	–	1) Presence of a circumscribed dense scar (BV <1.5 mV, area < 100 cm^2^) 2) Recurrent unmappable VT 3) Post-MI Exclusion: patchy scar/multiple scars	Endocardial 3.5-mm tip	1) PES 2) Substrate mapping: area of BV <1.5 mV + double, fractionated or LP 3) PES after ablation	Circumferential linear lesion along BZ (BV <1.5 mV) to isolate substrate	1) Lack of abnormal EGMs within area 2) No capture within area — achieved in 50% 3) Max. 40 RF lesion Noninducibility of any VT (no predefined endpoint) —observed in 92%	RF time 53 ± 15 min Procedure time 195 ± 64 min No complication	VT recurrence 33% Median follow-up 497 days
Tzou et al. (2015) [50] Two centers observational	44 Post-MI 32 44/566 pts. with SHD	31 ± 13	–	1) SHD 2) AAD refractory VT 3) Intention to achieve core isolation	Endocardial Epicardial post-MI 6% 3.5-mm tip Selected patients: multi-electrode catheters for exit block evaluation	1) BV mapping 2) PES 3) Activation mapping 4) Substrate mapping Dense scar BV <0.5 mV; BZ BV 0.5–1.5 mV/voltage channels/ fractionated/LP; pace-match, S-QRS >40 ms 5) PES after core isolation	1) Circumferential linear lesion to isolate core (=confluent area of BV <0.5 mV area and regions with BV <1 mV harboring VT-related sites 2) Targeting fractionated and LP within core 3) Targeting VT-related sites outside core (2 and 3 in 59%)	1) No capture of the ventricle during pacing inside core 2) Dissociation of isolated potentials — core isolation achieved in 70% post-MI 3) Noninducibility —achieved in 84%	RF lesions 111 ± 91 Procedure time 326 ± 121 min Complications 2.2% No death	VT recurrence 14% Follow-up 17.5 ± 9 months
Silberbauer et al. (2014) [630] One center observational	160	28 ± 9.5 inducible after RFCA 34 ± 9.2 endpoint reached	22.5%	1) Post-MI 2) AAD refractory VT 3) First VT ablation at the center	Endocardial Combined endoepicardial (20%) — Clinical findings — Prior ablation — Research protocol 3.5-mm tip/4-mm tip	1) Substrate mapping: BV <1.5 mV + LP (=continuous, fragmented bridging to components after QRS offset/inscribing after QRS, no voltage cutoff) + early potentials (EP = fragmented <1.5 mV) Pace-match 2) PES 3) Activation mapping 4) PES after substrate ablation	1) Ablation mappable VT 2) Ablation of all LP LP present at baseline Endocardium 100/160 pts. Epicardium 19/32 pts	1) Abolition of all LP — achieved at endocardium in 79 pts. (49%), at epicardium 12/32 pts. (37%) 2) Noninducibility of any VT — achieved in 88%	RF time endocardial median ≈25 min epicardial ≈6 min Procedure time Median 210–270 min Complications 3.1% In-hospital mortality 2.5%	VT recurrence 32% after median 82 (16–192) days VT recurrence according to endpoint 1 + 2 achieved (16.4%) Endpoint 2 achieved (46%) No endpoint achieved (47.4%)
Wolf et al. (2018) [631] One center observational	159	34 ± 11	25%	1) Post-MI 2) First VT ablation 3) Recurrent, AAD refractory episodes VT	Endocardial Combined endoepicardial 27% — Epicardial access was encouraged — Epicardial ablation 27/46 pts. 3.5-mm tip (70 pts) Multielectrode catheters (89 pts)	1) PES 2) Activation mapping 3) Substrate mapping: BV mapping (<1.5 mV) + LAVA (=sharp high-frequency EGMs, possibly of low amplitude, distinct from the far-field EGM occurring anytime during or after the far-field EGM 4) PES	1) Ablation of mappable VT 2) Ablation of LAVA (until local no capture) LAVA present at baseline Endocardium 141/157 pts. Epicardium 36/46 pts	1) Abolition of LAVA — achieved in 93/146 pts. (64%) 2) Noninducibility — achieved in 94/110 tested pts	RF time 36 ± 20 min Procedure time 250 ± 78 min Complications 7.5% (4 surgical interventions) Procedure-related mortality 1.3%	VT-free survival 55% during 47 months (33–82) Outcome according to endpoints: LAVA abolished vs not abolished 63% vs 44% VT-free survival at 1 year 73%
Berruezo et al. [46] One center observational	101 Post-MI 75	36 ± 13	–	1) Scar-related VT	Endocardial Combined endoepicardial (27/101 pts., among post-MI not provided) — Endo no substrate/suggestive epi — CE-MRI — VT ECG 3.5-mm tip	1) Substrate mapping: BV (<1.5 mV) + EGMs with delayed components: identification of entrance (shortest delay) of conducting channels 2) PES 3) Activation mapping + pace-match	1) Scar dechanneling targeting entrance 2) Short linear lesions (eg, between scar and mitral annulus) 3) Ablation of VT-related sites — performed in 45%	1) Scar dechanneling — Achieved in 85 pts. (84.2%) — Noninducible after 1) 55 pts. (54.5%) 2) Noninducibility —achieved in 78%	RF time 24 ± 10 min only scar dechanneling (31 ± 18 min + additional RFCA) Procedure time 227 ± 69 min Complications 6.9% No death	VT recurrence 27% after a median follow-up of 21 months (11–29) 1-year VT-free survival according to endpoint: scar dechanneling complete vs incomplete (≈82% vs ≈ 65%)
Porta-Sánchez et al. (2018) [127] Multicenter observational	20	33 ± 11	–	1) Post-MI 2) Recurrent VT	Endocardial 3.5-mm tip 4 pts. Multielectrode catheters 16 pts	1) Substrate mapping: annotation of LP (=fractionated/isolated after QRS offset) and assessment if LP showed additional delay of >10 ms after RV extrastimuli (S1 600 ms, S2 VERP +20 ms) defined as DEEP 2) PES 3) Additional mapping	1) Targeting areas with DEEP 2) Ablation of VT-related sites discretion of operator	1) Noninducibility— achieved in 80% after DEEP ablation — Remains 80% after additional ablation in those inducible	RF time 30.6 ± 21.4 min Procedure time and complications not reported	VT recurrence 25% at 6-month follow-up
de Riva et al. (2018) [686] One center observational	60	33 ± 12	30%	1) Post-MI 2) Sustained VT	Endocardial Epicardial 10% — Endocardial failure — Epicardial substrate suspected 3.5-mm tip catheter	1) PES 2) Substrate mapping: systematic assessment of presumed infarct area independent of BV during SR and RV extrastimuli. Pacing (S1 500 ms, S2 VRP + 50 ms): EDP (evoked delayed potentials) = low voltage (<1.5 mV) EGM with conduction delay >10 ms or block in response to S2 3) Activation and pace mapping	1) Targeting EDPs only 2) Ablation of VT-related sites based on activation/pace mapping	1) Elimination of EDPs — achieved in all 2) Noninducibility of targeted VT (fast VT with VTCL≈VERP not targeted) — Achieved in 67% after EDP ablation — Achieved in 90% after additional ablation	RF time 15 min (10–21) Procedure time 173 min (150–205) Complications 3.3% One procedure-related death	VT recurrence 22% at median follow-up of 16 months (8–23) Subgroup of patients with EDPs in normal-voltage areas at baseline (hidden substrate) compared to historical matched group without EDP mapping VT-free survival at 1 year 89% vs 73%

Included studies: post myocardial infarction (or data for patients post myocardial infarction provided)

*AAD* antiarrhythmic drug, *BV* bipolar voltage, *BZ* border zone, *CABG* coronary artery bypass grafting, *CE-MRI* contrast-enhanced magnetic resonance imaging, *DC* delayed component, *DEEP* decremental evoked potential, *ECG* electrocardiogram, *EDP* evoked delayed potential, *EF* ejection fraction, *EGM* electrogram, *ICD* implantable cardioverter defibrillator, *LAVA* local abnormal ventricular activity, *MI* myocardial infarction, *PEA* pulseless electrical activity, *PES* programmed electrical stimulation, *pts* patients, *PVC* premature ventricular complex, *RCT* randomized controlled trial, *RF* radiofrequency, *RFCA* radiofrequency catheter ablation, *RV* right ventricle, *SHD* structural heart disease, *SR* sinus rhythm, *VT* ventricular tachycardia

There is currently no standardized approach for substrate-guided ablation, and definitive comparisons between different techniques and endpoints are not available. The procedural outcomes (eg, noninducibility, elimination of LPs or LAVA, substrate or core isolation, scar dechanneling) are acute endpoints that cannot be easily randomized and/or applied to all patients with VT. The association between a procedure’s endpoint and favorable VT-free survival might simply reflect a less complex substrate in patients for whom the desired endpoint can be achieved. However, success with more comprehensive ablation strategies has been consistently observed [676].

It is important to note that studies have suggested various definitions for abnormal electrograms as surrogates for the VT substrate targeted by ablation [38, 47, 51, 81, 127, 630, 631, 685–687]. Validation is challenging and usually limited to mappable VTs [38]. The specificity and sensitivity of these abnormal electrograms for identifying the VT substrate throughout the range of observed post-MI scars recorded with different catheters, electrode sizes, and interelectrode spacings remain to be determined.

High-resolution mapping of post-MI reentrant VT in an animal model of reperfused MI indicates that functional conduction block during VT can play an important role [643], and these areas might not be detectable during sinus rhythm or pacing at slow rates. Targeting sites that show delayed conduction after delivering extrastimuli has been shown to be beneficial, with a smaller target area and lower VT recurrence than when other sites were targeted [127, 686].

This result might be particularly relevant for patients with small, less confluent or nontransmural scars after MI, in whom parts of the VT substrate might be functional, and a systematic application of an RV short-coupled extrastimuli can be useful for identifying hidden substrate components.

High-density multielectrode mapping with smaller electrode sizes and spacings can be helpful in obtaining more complete and rapid characterization of the arrhythmogenic substrate (Section 8), allowing for superior characterization of low-amplitude local electrograms from thin endocardial myocardium, which can be obscured by large far-field potentials from adjacent myocardium if catheters with large electrodes with distant interelectrode spacing are used [639–641, 682]. Further studies are required to assess the effect of these mapping techniques on ablation outcomes.

Finally, imaging is of major importance for identifying scars and the critical arrhythmogenic substrate within the scar. The use of various imaging modalities has been detailed in Section 5. Numerous studies have demonstrated a good correlation between CMR-defined scars and EAM-defined scars. The same is true for CT in which the wall thickness has been employed to identify scars in patients post infarction. Increased tissue heterogeneity within the scar demonstrated by CMR has helped to identify critical sites that can be selectively targeted [492], resulting in decreased VT recurrences. Likewise, CT-defined scar topography helps to identify critical VT target sites [501]. Critical areas are often located in myocardial ridges that are separated by areas of thinning [501].

#### Epicardial mapping and ablation

The role of postinfarction epicardial mapping and ablation is not clearly defined.

Despite a predominant subendocardial substrate in most patients, reentry circuits can be confined to the subepicardial layer [578]. The current incidence of epicardial substrate in patients referred for catheter ablation is unknown. ECG features are not reliable for predicting epicardial LV-VT exit in postinfarction VT [585]. Epicardial circuits might also be interrupted from the endocardium, particularly in areas with wall thinning, and evidence of this phenomenon is more frequently observed in post-MI patients than in patients with DCM [897]. Some 42%–57% of all post-MI patients included in multicenter studies [71, 72, 307, 317] and 23%–41% of patients from single-center series [629–631] had previously undergone cardiac surgery. In most patients, the presence of substantial pericardial adhesions from surgery or postinfarct pericarditis requires the creation of a surgical window in the electrophysiology lab or operating room to access the pericardial space. Even after epicardial access has been achieved, mapping can be particularly limited over the anterior wall [332, 562]. Continued oral anticoagulation and dual antiplatelet therapy raise the concern for bleeding risks associated with pericardial puncture [631].

In experienced referral centers, percutaneous or surgical epicardial access has been considered appropriate in 9%–29% of patients [332, 629–631]. At least 6% of post-MI patients referred for VT ablation benefit from an epicardial procedure [332]. A previously failed endocardial ablation preceded most of the epicardial procedures. Caution is advised, however, given the often associated comorbidities and the large proportion of patients with insufficient target structures that can be safely ablated in the epicardium [332]. Furthermore, procedural failure can be related to reasons other than an epicardial VT origin.

Based on the available data, the relative contribution of first-line epicardial ablation to a patient’s outcome remains unclear. For a large number of patients, accessible epicardial ablation target sites cannot be identified, and these patients are exposed to additional procedural risks and discomfort. Considering the higher risk for procedural complications with an epicardial approach [60], careful patient selection is warranted. Further study is required to determine whether preprocedural imaging can help to identify post-MI patients who will benefit from an epicardial approach [68, 334].

### Dilated cardiomyopathy

**Key Points**

• Identifying the location and extent of scarring on CMR is beneficial in procedural planning and has improved the outcomes of ablation in patients with DCM.

• The ablation strategy is similar to postinfarction VT.

• An intramural substrate is more frequently encountered in DCM than in postinfarction patients and requires a different ablation strategy than for patients with either epicardial or endocardial scarring.

• Epicardial ablation is beneficial if the scar is located in the epicardium of the LV free wall.

• For intramural circuits involving the septum, epicardial ablation is not beneficial.

• In the absence of CMR, unipolar voltage mapping has been described as a method to indicate a deeper-seated scar.

In nonischemic DCM, ablation may be considered for sustained monomorphic VT, recurrent polymorphic VT, or VF initiated by triggering PVCs (see Section 9.9), and for frequent nonsustained VT and PVCs that are suspected of contributing to ventricular systolic dysfunction or interfering with effective delivery of cardiac resynchronization pacing (see Section 4.3).

The prevalence of DCM is estimated to be in the range of 37 cases per 100,000 population [898]. Although the etiology is often clinically obscure, approximately 40% of patients have a genetic cause [898]. Although uncommon, sustained monomorphic VT occurs at an annual rate of approximately 3%–4% in patients with an ICD, a LVEF ≤0.35, nonsustained VT, and no prior history of sustained VAs [899]. In the DANISH trial, fewer than 3% of the patients with class II or III heart failure symptoms and an LVEF ≤0.35 had sustained VT during a median follow-up of 5.6 years [900]. In a series of 158 patients with DCM, a mean LVEF of 0.31, and no history of sustained VT, 13% of the patients were found to have inducible sustained monomorphic VT [901]. VT due to DCM is occasionally encountered in patients with atherosclerotic coronary artery disease and is suggested by the presence of multiple basal origin VTs and scar regions extending beyond the distribution expected from an infarct [902].

Three VT mechanisms have been identified in patients undergoing ablation [105, 348, 903], the most common of which is scar-related reentry, accounting for more than 80% of sustained monomorphic VTs. Reentry involving the bundle branches or fascicles (see Section 9.4) is encountered in up to 19% of patients, whereas focal VT mechanisms occur less frequently.

CMR with gadolinium contrast identifies areas of delayed hyperenhancement consistent with myocardial fibrosis in 30%–50% of patients with DCM, and the presence of this scarring is associated with an increased risk of sudden death and VT [904–906]. In patients with monomorphic VT, CMR also identifies scar areas that are a valuable guide to potential ablation target regions, given that the reentry circuit sites are usually associated with >25% scar transmurality [190, 192, 470, 907–910]. CMR is therefore particularly helpful in planning and performing ablation procedures in these patients. Knowledge of the scar location has improved ablation outcomes in patients with DCM [490].

The approaches to mapping and ablation for scar-related VTs in DCM are similar to those for postinfarction VTs; however, intramural substrates that are not easily identifiable or accessible for ablation are more common than in the postinfarct population and contribute to procedure failures and recurrences [192, 338, 471, 903]. The relevant scar area is identified from imaging and/or substrate mapping. When VT can be induced and is sustained and hemodynamically tolerated, a combination of activation mapping and entrainment mapping can be used to select the ablation region in or near the scar area [335, 471]. When VT is not mappable and an area of low voltage (bipolar voltage <1.5 mV) can be identified that is consistent in location with the VT QRS morphology, a substrate type of approach can be used in the endocardium and/or epicardium [336, 350, 360, 911]. Although isolated LPs and fractionated potentials are encountered less frequently than in postinfarct VT scars, these electrogram targets can still be used to guide ablation [41, 360]. Pace mapping can suggest the VT exit region; however, as with other scar-related VTs, critical parts of the reentry circuit where ablation may also be successful can be several centimeters distant from the exit region. One study found that when a low-voltage area is present, extensive ablation over the region (both endocardially and epicardially) was associated with a lower risk of VT recurrence compared with targeting only inducible VTs [358]. Absence of inducible VT after ablation is associated with a lower risk of VT recurrence [313, 343, 912]. Another study found that the presence of only rapid VTs after ablation (CL <30 ms plus the RV effective refractory period measured at a pacing CL of 400 ms) was not associated with a greater risk of recurrence [892].

Scar-related VTs tend to originate from scar regions along the mitral annulus or within the interventricular septum [55, 192, 335, 471]. Multiple morphologies of inducible VT and poorly tolerated VTs are common. The VT circuit can involve the endocardial, epicardial, or intramural regions of the LV. The ECG morphology of the VT is a useful guide to the likely target region and can inform the initial approach. The risk, outcomes, and approach to ablation also vary with the substrate location.

VTs that originate from an LV scar in the free wall along the mitral annulus typically have an RBBB configuration and dominant R waves in the midprecordial leads (V3, V4), with an axis directed inferiorly (if the VT exit is superior) or superiorly (if the VT exit is inferiorly located) [192, 471]. A qS or QS configuration in lead I often indicates an epicardial exit, consistent with initial forces directed from the epicardium to the endocardium. The QRS morphology can be misleading, however, particularly for fast VTs [177, 470]. Epicardial ablation is often helpful, although some of these VTs can be successfully ablated from the endocardium. With LV free wall VTs, some laboratories start with mapping in the epicardium, obtaining epicardial access before anticoagulation is administered. Other laboratories always start on the endocardium to avoid the risks of epicardial access and obtain this if the endocardial approach fails. In reported series from experienced centers, epicardial mapping is performed in 29%–74% of patients [192, 321, 335, 339]. The approach to epicardial ablation is reviewed in Section 6.3. Recognizing that low voltage can be recorded over epicardial fat and avoiding the left phrenic nerve and coronary artery injury are important considerations [913].

VTs that originate from the anteroseptal region typically have a dominant S wave in V1, with an inferior axis and prominent R waves by V3 to V4 [192, 471]. The frontal plane axis can be inferiorly or superiorly directed. If the scar extends leftward along the aortic root and toward the AMC region, the VT can have a dominant R wave in V1 with an inferior axis. If the scar extends inferiorly in the septum, VTs with a superiorly directed axis can be present. Ablation for VTs that originate in scars at the anteroseptum has a lower success rate compared with those from the inferolateral basal LV in a number of studies [192, 471]. Access from the epicardial aspect is limited by the overlying RVOT, the epicardial fat pad, and the left coronary arteries. In the septum, ablation can be limited by proximity to the AV conduction system. Determining the risk of heart block and managing its consequences are important considerations. Implementation of pacing for CRT might be warranted if ablation results in AV block.

Intramural VTs can occur in any location but are common in the periaortic region and septum [55, 192, 471]. Preprocedural CMR is the gold standard for identifying scarring and is particularly helpful in identifying intramural scars (Section 5). Identification of intramural scars is best accomplished by a preprocedural CMR. In the absence of CMR data, intramural scarring is suspected when the endocardial bipolar voltage is >1.5 mV (with 3.5-mm electrode mapping catheters) over the endocardial region that is closest to the VT. If located at the LV free wall, an epicardial origin can be excluded when epicardial mapping fails to identify a VT substrate in the overlying epicardium. The unipolar endocardial voltage can be helpful for identifying deeper seated scar tissue. An LV unipolar voltage <8.3 mV over an area with an endocardial bipolar voltage >1.5 mV is consistent with intramural or epicardial scars; however, electrogram amplitude is also dependent on wall thickness, and there is substantial variability [55, 65, 914, 915]. As indicated in Section 8.5.2, different voltage cutoff values have been reported, and validation studies with CMR-defined scars have demonstrated that there is a substantial overlap of unipolar low voltage between scar zones and regions without scars. These cutoff values should therefore be used with caution in the absence of available CMR data. Septal intramural scars are suggested by a conduction time of >40 ms when pacing is performed on the RV side of the septum, and recording from the adjacent LV septum [56]. Use of intracardiac ultrasound has also been suggested for detecting scars, but data are limited [586]. The optimal approach to ablation for these VTs is not defined; however, ablation targeting the closest overlying endocardial and epicardial sites is effective in some patients.

Overall outcomes are largely from case series published by experienced centers, a number of which have included results from multiple procedures [192, 335, 336, 338, 339, 350, 358, 360, 471, 912, 916]. Ablation abolishes at least 1 inducible VT in 60%–74% of patients and all inducible VTs in 43%–72% of patients. Programmed stimulation is not performed after ablation in 10%–15% of patients, largely due to hemodynamic concerns. Persistent inducibility of sustained monomorphic VT is associated with increased recurrences [313, 343, 912]. During median/mean follow-ups ranging from 15 to 48 months, 31%–61% of patients experience at least 1 VT recurrence. Approximately half of those with recurrent VT experience fewer VT episodes than prior to ablation [192, 335]. When directly compared in the same center, recurrent VT is more frequent after ablation for DCM VT than for postinfarction VT [338]. Transplant-free survival ranges from 76% to 89%. Patients with large scar areas associated with larger areas of low voltage and VTs that have LV apical exits, often associated with scar extending from base to near the apex, have increased mortality during follow-up [916, 917].

Complications, including vascular access bleeding, tamponade, and volume overload, are reported in 4%–11% of patients [335, 336, 912]. Serious complications, including tamponade requiring surgery, phrenic nerve injury, and coronary artery injury, are more frequent when percutaneous epicardial access is performed [321, 335]. Thromboembolic complications appear to be rare, but pulmonary embolism can occur. In-hospital mortality is as high as 3% and has been reported due to uncontrollable VT, refractory heart failure, and tamponade sequelae [336]. When LV systolic function is severely depressed, the option of cardiac transplantation or an LVAD is an important consideration if the arrhythmia cannot be controlled or if the procedure is complicated by hemodynamic deterioration. Mechanical support has also been employed during ablation procedures to prevent hemodynamic deterioration and to facilitate mapping (Section 6). Ablation that results in LBBB or AV block can lead to hemodynamic deterioration due to cardiac dyssynchrony that can warrant implementation of biventricular pacing. Extensive ablation of areas of normal myocardium has the potential to further impair ventricular function, although this risk has not been clearly defined. It seems prudent to avoid empiric ablation over large regions that are not known to contain scars.

### Ventricular tachycardia ablation in hypertrophic cardiomyopathy

**Key Points**

• Polymorphic VT and VF are the most common VAs in HCM; monomorphic VT is less common.

• The arrhythmogenic substrate in HCM often involves the septum but can extend to the epicardium, often necessitating combined endocardial and epicardial ablation procedures to eliminate the VT.

• VT associated with apical aneurysms is often ablated endocardially.

Malignant VA contributes to sudden death in patients with HCM. ICDs are therefore the mainstay of prevention. The substrate for VAs in HCM is complex. The combination of myofibrillar disarray and fibrosis likely generates heterogeneous conduction properties and, together with the vulnerability of the hypertrophied myocardium to supply-demand ischemia, creates a potentially arrhythmogenic milieu [918–920]. Interstitial-type fibrosis (compared with replacement fibrosis), postmyectomy scars, alcohol ablation, and the presence of an apical aneurysm can also influence arrhythmogenesis [921]. The fibrotic areas visualized on CMR that characterize HCM can result in a similar substrate to scars from ischemic disease, in which case similar techniques can be applied during VT ablation [922–925]. However, increased myocardial thickness and intramyocardial or epicardial sites of fibrosis limit the reach of classical mapping and ablation methods. The varying mechanisms (eg, reentrant vs triggered) and morphology (monomorphic vs polymorphic) of VAs encountered in HCM represent further challenges. ICD records indicate that polymorphic VT degenerating to VF is the most common event [926–928].

Ablation is generally performed for monomorphic VT that recurs despite antiarrhythmic therapy, particularly with ICD shocks. These VAs are only a small proportion of VAs in HCM, and the ablation experience is limited to case reports and small case series of highly selected patients. In a series of 10 patients with preserved LVEF (LVEF of 57% ± 13%), programmed stimulation induced clinical VT in 7 patients [446]. A mean of 2 VTs per patient (including nonclinical VTs) were induced. VTs were sometimes stable and permitted activation and entrainment mapping. Voltage mapping identified a combination of epicardial and endocardial scars in most patients. Isolated endocardial or epicardial scarring rarely occurred. The ablation strategy entailed targeting late or fractionated electrograms and pace mapping and/or substrate modification targeting low bipolar voltage (≤1.5 mV) regions. It is worth noting that epicardial ablation was required in most patients. During a 3-year follow-up, 30% of the patients underwent recurrent shocks, with repeat procedures required for 2 patients.

A similar approach was used in another series of 22 patients with more advanced disease (mean LVEF of 35%) [447]. Scar-related VTs occurred most often (60%) from the LV-RV junctions, either at the basal (42%) or apical (18%) LV segment level (coinciding with the anatomical regions frequently associated with fibrosis). Epicardial ablation was required in almost two-thirds of the patients, and 73% remained arrhythmia-free at 20 months. In another report of 5 patients with the dilated phase of HCM (LVEF of approximately 35%), VT circuits were predominantly distributed in the basal septum and the basal anterior to anterolateral LV. In addition to the endocardial ablation, intracoronary ethanol ablation and surgical cryoablation were required in a number of patients [448].

Patients with HCM and an LV apical aneurysm have a greater risk of sudden death, embolic stroke, and progressive heart failure than the general population with HCM and warrant special consideration [929]. The thin-walled dyskinetic or akinetic segment can be composed of dense scars with channels of viable myocardium (resembling ischemic substrate). Both sustained monomorphic VTs and VTs with an RBBB or LBBB pattern in lead V1 can occur, the latter consistent with an exit from the septal aspect of the aneurysm. Careful imaging is important for defining the aneurysm and ruling out thrombi prior to the procedure. Transthoracic contrast echocardiography, left ventriculography, and real-time visualization with ICE can be helpful. Anticoagulation therapy is a reasonable postprocedure treatment. Ablation strategies can include those techniques used for ICM [930]. In a recent series, endocardial ablation was successful in most patients, and epicardial ablation was required in only 1 patient [450]. In a number of cases, however, the aneurysm does not have low-voltage endocardial or epicardial scars. When ablation is not possible or is ineffective, surgical approaches could be successful [931, 932].

Common themes emerge from these reports on ablation for monomorphic VT in HCM. ECG characteristics might not be helpful for locating epicardial or intramural circuits [930]. Preprocedural imaging is recommended when deciding on the endocardial vs combined endocardial-epicardial approach, as in other VT substrates (see Section 5). If unavailable, epicardial access can be obtained at the beginning of the case in anticipation of the need for combined endocardial-epicardial ablation [446]. Intramural reentry might be difficult to target because the myocardium is thick. Mapping methods can include a combination of voltage-based substrate mapping (using conventional voltage ranges) and activation, entrainment, and late or fractionated potential mapping. VTs associated with apical aneurysms are often ablated endocardially. Although the arrhythmia mechanisms for monomorphic VTs in HCM appear to be mostly scar-related reentry, reports have also mentioned ablation of BBR, focal RVOT [446, 930], papillary muscle VTs, and left posterior fascicular VT [933]. Reports have not suggested an increased incidence of major complications compared with VT ablation in other populations, although cardiac tamponade can be more of a concern in patients with apical aneurysms. The results of ablation are encouraging, acutely eliminating VT in 80% of patients, with more than two-thirds of patients free of VT in the long-term follow-up. If catheter ablation is ineffective, other methods, including transcoronary alchohol and surgical resection and ablation, may be considered. Methods enhancing lesion size in order to reach the deep arrhythmia substrate will likely facilitate treatment.

### Brugada syndrome

**Key Points**

• PVC-triggered VF or polymorphic VT are the most prevalent VAs that motivate device therapy in patients with Brugada syndrome.

• Monomorphic VT is less frequent but can be caused by BBRVT in patients with Brugada syndrome.

• The arrhythmogenic substrate is located in the RV epicardium and can be demonstrated by sodium channel blockers.

• Ablation targets include fractionated prolonged electrograms on the epicardial aspect of the RV.

#### Introduction

Catheter ablation for VA in Brugada syndrome may be considered for VF, polymorphic VT, sustained monomorphic VT or PVCs triggering VF or polymorphic VT. Ablation strategies have evolved from initially only targeting the PVC triggers for VA storms to the current complete elimination of the arrhythmogenic substrate that is usually located on the epicardial aspect of the RVOT [102, 934].

Although quinidine can be effective for VA suppression in Brugada syndrome, its use is complicated by its limited availability, its difficult pharmacokinetics, and its adverse effects in two-thirds of patients, leading to discontinuation in a quarter of patients in a recent randomized double-blind trial [935]. The clinical indications for catheter ablation in Brugada syndrome are detailed in Section 4.

The worldwide prevalence of Brugada syndrome ranges from 0.5 to 35.5 per 1000 inhabitants [936]. Brugada syndrome was first systematically described in 1992 in a series of 8 patients with VF arrest. Brugada syndrome is an inherited arrhythmogenic condition defined on the basis of its characteristic ECG phenotype [937]. An RBBB-like pattern with ≥2 mm coved ST segment elevation in the right precordial leads constitutes the so-called type I Brugada pattern necessary for diagnosis [938]. This pattern is dynamic and can fluctuate according to variations in autonomic tone, body temperature, diurnal influences, electrolyte levels, and drug exposure, especially drugs with sodium channel blocking properties [939–941]. Additionally, the ECG pattern can be concealed unless V1 and V2 are recorded at the higher second and third intercostal spaces [942, 943]. Multiple conditions can present as a Brugada phenocopy and need to be ruled out before a diagnosis of Brugada syndrome can be reached [944]. The inheritance pattern for Brugada syndrome is usually autosomal dominant. Although its genetic basis was initially identified as a loss-of-function mutation in the *SCN5A* gene encoding the α-subunit of the cardiac sodium channel *Na*_*V*_*1.5*, numerous genetic culprits have since been recognized [945].

The mechanism generating the Brugada syndrome phenotype has been the focus of significant research, and considerable recent evidence has implicated depolarization abnormalities in the pathogenesis of Brugada syndrome [946]. However, repolarization abnormalities have also been described, with disproportionate shortening of the epicardial action potential leading to the potential for phase 2 reentry and the induction of polymorphic VT or VF [947, 948]. Subtle but significant cardiac ultrastructural alterations, especially on the epicardial aspect of the RVOT, have also been found in numerous patients [949]. Electrophysiological and electroanatomical abnormalities have also been described in this location, present both spontaneously and upon provocation with sodium channel blockers or the pericardial instillation of warm water [950–952]. High-frequency, low-amplitude, long-duration fractionated electrograms are routinely recorded from this region and are the target of substrate-based ablation procedures [950]. Complete elimination of all spontaneous and provoked epicardial fractionation is associated with normalization of the Brugada ECG phenotype and with elimination of inducible and spontaneous VAs in most patients at the short-term follow-up [436, 950, 952–954].

#### Approach to triggering premature ventricular complexes

For patients with recurrent PVC-triggered VF or polymorphic VT with ongoing spontaneous PVCs, localization of these focal triggers by activation mapping or pace mapping has been the dominant procedural strategy [102]. Patients with Brugada syndrome, however, generally have little ambient ectopy in between phases of arrhythmic instability, and ablation targeting VF triggers is rarely reported. Moreover, isoproterenol administration can suppress VF storms and prevent VF inducibility and is not likely to be useful for provoking triggering PVCs during an ablation procedure [955, 956]. In most reported cases, foci have been mapped to endocardial sites in the RVOT [957, 958].

#### Approach to sustained monomorphic ventricular tachycardia

Sustained monomorphic VT can occur but is unusual in Brugada syndrome. Rodriguez-Manero et al. [959] observed Brugada syndrome in 4.2% of patients with appropriate ICD interventions, with approximately half of these patients able to be pace terminated. Most of these tachycardias were mapped to and ablated in the RVOT, although BBR was observed in 2 of the 8 patients who underwent ablation. Sustained monomorphic VT ablation is approached similarly to other conditions with reentrant VTs, and CMR imaging is beneficial for assessing scarring that might be due to another disease process. The effectiveness of empiric targeting of the epicardial RVOT substrate when sustained monomorphic VT is not inducible is not known.

#### Approach to polymorphic ventricular tachycardia/ventricular fibrillation

Polymorphic VT and VF causing recurrent ICD shocks or electrical storms are the most pressing arrhythmic indications for catheter ablation in Brugada syndrome. In the absence of a triggering PVC, the arrhythmogenic substrate in the RVOT is the only possible ablation target. The preponderance of experience finds this substrate located on the epicardial aspect of the RVOT, with only 10% of patients having some potential abnormal substrate identified from endocardial mapping [576]. For this reason, catheter ablation for Brugada syndrome should be performed at centers with experience in the specialized techniques of percutaneous pericardial access and epicardial mapping (see Section 6).

Percutaneous epicardial access can be obtained in accordance with the operator’s usual technique. The anterior pericardial access approach provides direct access to the epicardial RVOT, and the best access to this region via a posterior pericardial access approach can be achieved with the catheter passing through the transverse sinus. Double-wiring the initial access sheath allows for a second pericardial sheath to be placed, through which a multipolar mapping catheter can be introduced. The ablation catheter is usually deployed through a deflectable sheath to aid in obtaining optimal tissue contact.

**Fig. 10:**
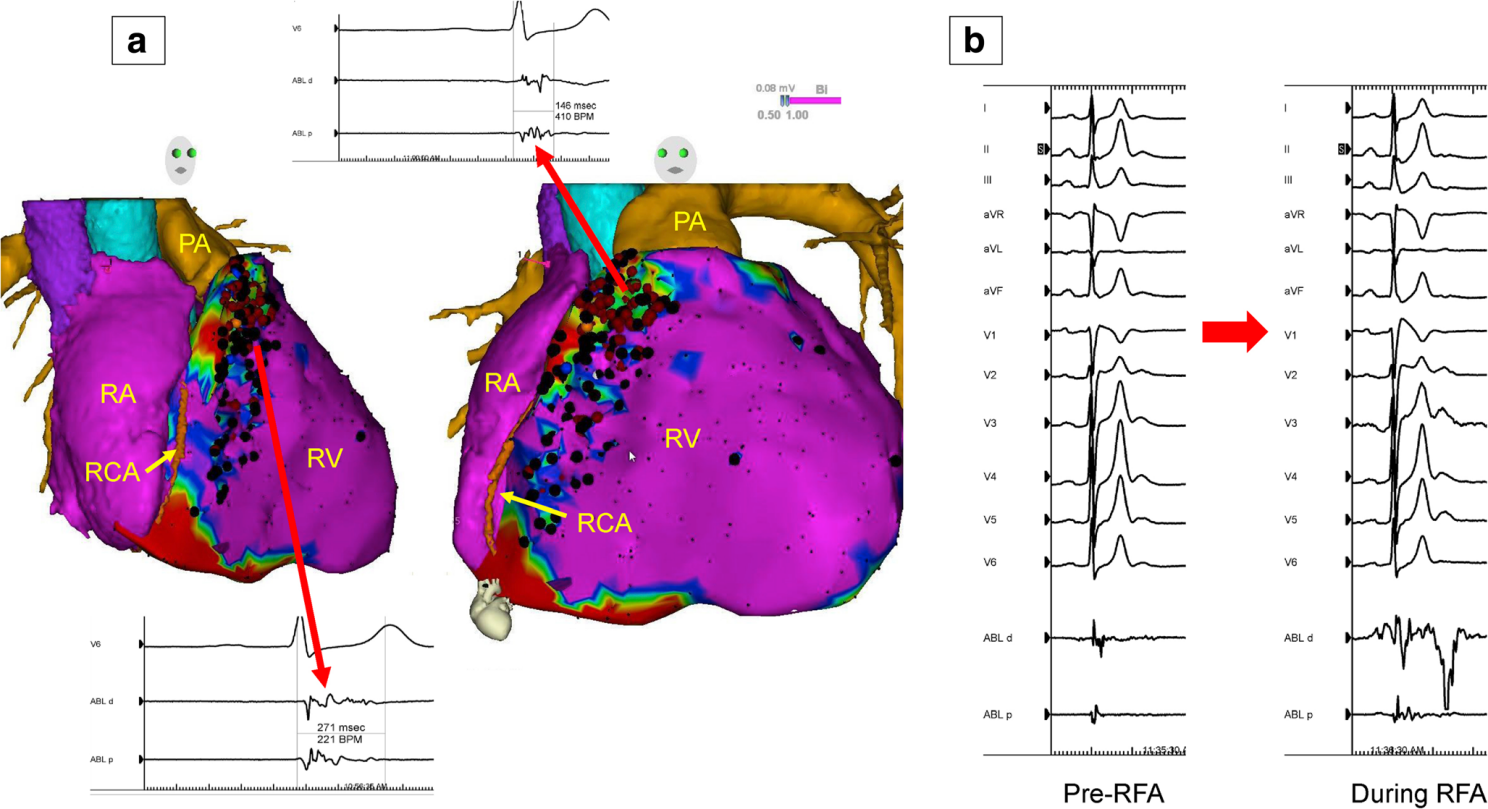
Epicardial substrate ablation in a patient with Brugada syndrome and appropriate ICD shocks for VF. Image integration of a preacquired CT with the electroanatomical epicardial substrate map is shown in (A). Purple represents bipolar voltage >1.5 mV. Fractionated potentials (arrows) are tagged with black dots, and a representative example is displayed. Widespread fractionated potentials were recorded from the epicardial aspect of the RVOT extending down into the basal RV body. Ablation lesions are tagged with red dots. Some fractionated potentials could not be ablated due to the proximity of the acute marginal branches of the right coronary artery. Panel (B) shows the significant transient accentuation of the Brugada ECG pattern during the application of radiofrequency energy at one of these sites. CT = computed tomography; ECG = electrocardiogram; ICD = implantable cardioverter defibrillator; PA = pulmonary artery; RA = right atrium; RCA = right coronary artery; RFA = radiofrequency ablation; RV = right ventricle; RVOT = right ventricular outflow tract; VF = ventricular fibrillation

Once the appropriate catheter set has been deployed, the relevant anatomy should be defined. Integration of a preacquired CT into the EAM, particularly with the assistance of ICE, is an efficient means for defining anatomy (Fig. 10a). Virtual epicardial (and/or endocardial RV) chamber geometry in the EAM system can also be acquired directly with a roving catheter.

Mapping efforts are then concentrated on the region of interest in the epicardial aspect of the RVOT. Multielectrode catheters with small electrodes can provide better definition of high-frequency fractionation than ablation catheters. Although mapping can commence in the basal state if the Brugada syndrome ECG pattern is present at baseline, several studies have shown an increase in the area of arrhythmogenic substrate after administration of a sodium channel blocker [435, 436, 953]. A similar effect has been observed with the pericardial instillation of warm water [952].

All high-frequency, long-duration, multicomponent, low-amplitude fractionated or isolated late electrograms are tagged on the substrate map (Fig. 10a). Given the potential presence of significant epicardial fat, low voltage in isolation is not indicative of underlying substrate. Caudal extension of the substrate has been associated with the presence of concomitant inferolateral J waves and early repolarization syndrome [960, 961].

After a complete map has defined the region and extent of the arrhythmogenic Brugada syndrome substrate, an ablation strategy can be devised. The most important factor in this regard is the relationship of the surrounding at-risk structures. Although the phrenic nerve is not usually a consideration, the proximal large subdivisions of the right coronary artery are potentially at risk, particularly the conus branch and the RV marginal arteries [961]. Ablation-induced acute occlusion of the former could potentially result in VF, and injury to the latter risks infarction of an otherwise normally contractile RV. Coronary angiography prior to lesion delivery is therefore strongly recommended. Coronary angiography of the left coronary arteries should also be performed because the disease process can also involve the LV epicardium.

An irrigated RF ablation catheter is used for epicardial lesion delivery, targeting the previously defined fractionated electrograms. Contact force sensing helps to confirm an optimal catheter contact vector toward the epicardium rather than outward toward the parietal pericardium. Characteristic accentuation of Brugada syndrome-type ST elevation in the right precordial leads is generally observed with RF application (Fig. 10b) and usually resolves after RF delivery ceases. However, acute occlusion of an RV marginal coronary artery needs to be ruled out if it persists. Ablation continues until all electrogram fractionation in the arrhythmogenic substrate region has been eliminated, both at rest and with repeat provocation with sodium channel blockers. When this has been achieved, the Brugada syndrome ECG phenotype has usually normalized (both in the standard and higher interspace recordings), and polymorphic VAs are no longer inducible.

#### Outcomes

There have been no randomized trials of catheter ablation in Brugada syndrome, and all reported clinical outcomes are from short-term follow-ups of largely single-center observational studies. These initial data are encouraging, with most investigators reporting 73%–100% freedom from recurrent VT/VF during follow-up [434–436, 950]. A systematic review of all published cases to 2018 found an overall 96.7% freedom from recurrent VT/VF [576]. However, given that recurrent VF was observed in several patients despite an apparently successful procedure, catheter ablation cannot be recommended as a replacement for ICD insertion to mitigate the risk of sudden death in symptomatic Brugada syndrome. Additionally, most of the reported patients had undergone ablation for symptomatic VA, and there are no controlled outcome data to support a role for catheter ablation in asymptomatic individuals with the Brugada syndrome ECG phenotype.

#### Risks

Catheter ablation for Brugada syndrome is an invasive and complex procedure with significant potential for acute and delayed complications. These events include those related to epicardial access and epicardial ablation and the general risks related to invasive catheter ablation, which are detailed elsewhere in this document (see Sections 6.3 and 10.2). The most frequent reported complications are isolated pericardial effusions and pericarditis [434–436, 950]. No procedural deaths, strokes, MIs, or tamponades have been reported to date, but the early published worldwide experience includes only approximately 200 cases. It should be noted that these reports are from high-volume centers, and complication rates with more widespread deployment could be higher.

### Polymorphic ventricular tachycardia/ventricular fibrillation triggers

**Key Points**

• Recurrent PVC-induced VF is most often triggered by PVCs originating from Purkinje fibers, located in the RVOT, the moderator band, or the LV.

• Patients with a single triggering PVC are better ablation candidates; however, there are often multiple triggers.

• Patients with healed MI often require extensive ablation of the Purkinje fiber system within or at the scar border.

• Ischemia should be ruled out as a trigger for VF prior to ablation.

Although relatively rare, polymorphic VT and VF can be triggered by PVCs in patients with or without structural and/or electrical heart disease (long QT syndrome, catecholaminergic polymorphic VT, Brugada syndrome) [82, 102, 135, 299–301, 962–964]. When this occurs, the clinical presentation is often a polymorphic VT/VF storm [82, 300, 301, 962, 963]. The syndrome is recognized when short-coupled, unifocal PVCs (typically from the RVOT or Purkinje fiber system, including the RV and LV papillary muscles) [135, 299, 300] trigger polymorphic VT or VF episodes. Ruling out acute myocardial ischemia as the cause is important. Several considerations have emerged from a number of small studies on catheter ablation in patients with polymorphic VT or VF refractory to antiarrhythmic therapy [82, 102, 135, 299–301, 962–964]: 1) mapping and ablation is facilitated when the index PVCs are frequent; procedural planning includes withdrawal of AADs where feasible and minimal sedation; 2) ablation sites are often associated with presystolic Purkinje activation; in some instances, there can be slight variation in PVC morphology and Purkinje potential to local ventricular activation time, suggesting different exits from the same source [299]; 3) in the setting of MI, successful ablation sites are related to Purkinje activation in the infarct border zone [301, 962–964]; 4) multiple ablation lesions are typically delivered, given the relative imprecision of mapping and the serious consequences of failed ablation; and 5) although acute procedural success in experienced centers is high, late recurrence of PVC-triggered polymorphic VT/VF is observed in 5%–15% of patients, emphasizing the need for defibrillator therapy. Multipolar mapping and ICE imaging, especially for PVCs originating from papillary muscles, is helpful.

As discussed in Section 4.3, catheter ablation is indicated for patients with drug-refractory, recurrent, monomorphic PVCs triggering polymorphic VT/VF in the absence of SHD and for patients with remote MI. In the acute phase of MI or in the early phase following coronary revascularization, PVC-triggered polymorphic VT/VF is initially best treated conservatively, given that these arrhythmias often resolve spontaneously in a relatively short period.

### Arrhythmogenic right ventricular cardiomyopathy

**Key Points**

• The arrhythmogenic substrate in ARVC is located in the epicardium and can involve the endocardium in advanced stages.

• The most commonly affected areas are the subtricuspid and RV outflow regions.

• LV involvement is not uncommon.

• Endocardial-epicardial ablation is often required and results in higher acute success and lower recurrence rates compared with endocardial ablation alone.

• Conventional mapping and ablation techniques, including entrainment mapping of tolerated VT, pace mapping, and substrate ablation, are used.

#### Introduction to the specific disease substrate characteristics

ARVC is a genetically determined myocardial disease characterized by progressive RV fibrofatty replacement, VAs, heart failure, and sudden cardiac death. Fibrofatty replacement starts at the epicardium or midmyocardium and extends until becoming transmural [965]. The scar distribution predominantly affects the subtricuspid and OT regions and, less frequently, the apex [966–968]. LV involvement is present in more than half of all cases and is more frequent in advanced stages [423], affecting the posterolateral subepicardium [423, 969]. ARVC is typically diagnosed through the established Task Force Criteria, although there is increasing evidence supporting a role for invasive EAM to diagnose the early stages of ARVC when Task Force Criteria are inconclusive [155, 970, 971]. In the early stages of the disease, VT reentry circuits are located nearly exclusively in the epicardium [54, 423, 425, 968]. In more advanced disease, the VT substrate reaches the endocardium, and areas in the epicardium can be fibrotic and lack reentry circuits [54, 423, 425, 968].

#### General management

ICDs are recommended to prevent death in patients with ARVC and VAs and can also be considered in those with recognized risk factors who have not yet had VT [4]. Neither AADs nor catheter ablation provides sufficient protection against sudden cardiac death. AADs (sotalol and amiodarone) are frequently used to improve symptoms and to prevent recurrent VT episodes, but have limited efficacy. Sotalol has been shown to reduce VT inducibility in an electrophysiology study [972]; however, data on its long-term efficacy are limited. Limited evidence suggests that amiodarone is the most effective drug for preventing VAs [973]. Flecainide combined with metoprolol or sotalol has also been used to suppress arrhythmias [974]. Catheter ablation is commonly used to reduce the frequency of arrhythmia episodes.

#### General approach for ablation

Given that ARVC affects the epicardium first, endocardial VT ablation alone is often insufficient [54, 62, 427, 429, 430] and can result in VT recurrence. A combined endoepicardial approach as first-line therapy [64, 423, 568, 968] or adjuvant epicardial ablation after unsuccessful endocardial ablation or after VT recurrence results in improved acute and long-term outcomes [63, 425]. After combined or adjuvant epicardial ablation, VT reoccurs in 16%–29% of patients after approximately 3 years of follow-up, with varying proportions of patients in these studies taking AADs [63, 64, 423, 425, 568]. Predominant LV involvement is an independent predictor of recurrence after ablation [423].

**Fig. 11:**
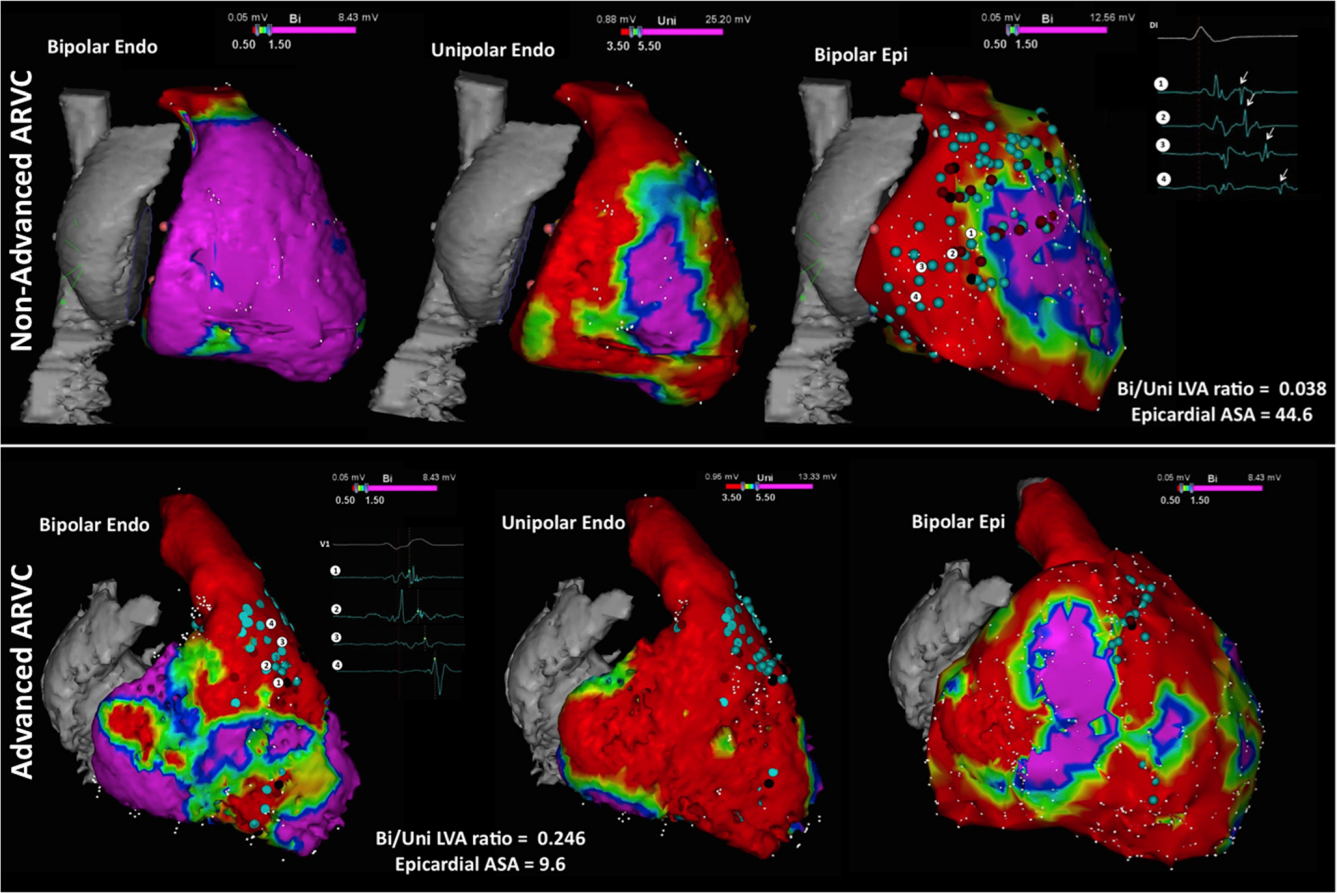
Right ventricular voltage maps from cases of moderate (upper row) and advanced (lower row) arrhythmogenic right ventricular cardiomyopathy (ARVC) are shown. Purple represents a voltage >1.5 mV in the bipolar maps (left and right) and > 5.5 mV in the unipolar maps (center); red represents a voltage <0.5 mV in the bipolar maps and < 3.5 mV in the unipolar maps. Moderate ARVC is defined as having a bipolar/unipolar low-voltage area ratio of <0.23 and is associated with epicardial arrhythmogenic substrate area (ASA) (defined by the presence of electrograms with delayed components of >10 cm^2^). Advanced ARVC displays a bipolar/unipolar endocardial low-voltage area of ≥0.23, which is associated with an epicardial arrhythmogenic substrate area of ≤10 cm^2^ [423]. Adapted with permission from Oxford University Press [423]

Given the variable endocardium-to-epicardium disease involvement, the benefit of epicardial ablation likely varies among patients. The presence of an epicardial arrhythmic substrate in patients with limited or no endocardial VT substrate can be identified by endocardial unipolar voltage mapping [66]. Areas of endocardial unipolar voltage <5.5 mV are associated with epicardial low-voltage scar regions. Isolated epicardial involvement with a completely normal endocardial bipolar map has been observed in 26%–40% of patients [423, 425, 427, 968], and these patients might require epicardial ablation. The low-voltage areas are typically adjacent to the tricuspid valve annulus and in the free wall of the RVOT. In contrast, patients with advanced stage ARVC have more extensive involvement of the RV endocardium (wider areas of low voltage in the bipolar map) and can have less arrhythmic substrate in the epicardium, which is likely due to the progressive fibrofatty replacement of the subepicardial myocardium, such that the surviving myocardium supporting reentry is closer to the endocardium [423]. These patients constituted approximately 20% of patients with ARVC in a recent multicenter study and can be recognized by an endocardial bipolar vs unipolar low-voltage area ratio of ≥0.23 (Fig. 11) [423].

One strategy for guiding the selective use of epicardial mapping and ablation in ARVC is based on initial endocardial mapping. Epicardial access is obtained for patients whose endocardial voltage map shows limited or no endocardial substrate [eg, a bipolar vs unipolar low-voltage endocardial area ratio of <0.23 [423]], which is characteristic of early disease. Initial endocardial ablation is performed for patients with more extensive endocardial low-voltage substrate characteristic of advanced disease, followed by epicardial access for epicardial ablation if the VTs that are considered clinically relevant are still inducible. Alternatively, Santangeli et al. [63] found that a strategy of epicardial mapping and ablation only when VTs are still inducible or recur after endocardial ablation achieved good long-term outcomes.

Conventional mapping and ablation techniques used for other scar-related VT substrates are also employed in ARVC. Ablation can be performed during sinus rhythm, targeting the substrate based on electrogram characteristics and pace mapping (see Section 8) and/or during mappable monomorphic VTs. These ablation strategies have not been directly compared in patients with ARVC and are complementary and often combined. The procedure can be started during sinus rhythm with the hope of reducing radiation exposure and the need for electrical cardioversion [975]. Complete elimination of all substrate characterized by electrograms with delayed components by targeting the conducting channel entrances has been shown to be a feasible and efficient strategy, needing only a small amount of RF delivery in some patients [64, 423, 968]. Complete substrate elimination and noninducibility of any sustained monomorphic VT as procedural endpoints are associated with good long-term results.

Some VTs are catecholamine induced. A recent study found that high-dose isoproterenol infusions could induce PVCs that had the same morphology as sustained VTs, and focal ablation of the PVCs eliminated these catecholamine-mediated VTs [976].

#### Risks

The additional benefit in arrhythmia control obtained with epicardial mapping and ablation in ARVC should be carefully weighed against the risks associated with obtaining epicardial access. The risk of inadvertent RV puncture with pericardial bleeding can be increased with RV enlargement or advanced stage disease [423]. The incidence of major complications related to epicardial access has been reported to be as high as 8% (see Section 6.3) [59, 60, 63, 64, 425, 568]. Although the risk of coronary injury from epicardial ablation appears to be low, MI from an injury to an anomalous RV coronary branch has been reported [977].

### Mapping and ablation in congenital heart disease

**Key Points**

• Patients with a VT substrate after congenital heart defect surgery include those with repaired tetralogy of Fallot, repaired VSD, and repaired d-transposition of the great arteries (D-TGA), as well as Ebstein’s anomaly among other disease processes.

• VT isthmuses are often located between anatomical barriers and surgical incisions or patch material.

• An AI can be identified and targeted during sinus rhythm.

• For tolerated VTs, entrainment mapping is the method of choice for identifying critical components of the reentry circuit.

#### Introduction

As the population of patients with repaired CHD continues to grow, management of recurrent VT in this population becomes increasingly important (Fig. 2). Monomorphic sustained VT approachable by catheter or surgical ablation typically occurs in patients with ventricular incisions, surgical scars, and patch materials. Accordingly, for patients with CHD included in mapping and ablation studies, repair of tetralogy of Fallot is the most commonly performed procedure, followed by VSD closure and complex D-TGA repair [410–413, 415, 416, 421]. As in other patients with SHD who present with VT, a complete evaluation of factors that promote arrhythmias is necessary. Cardiac imaging to identify residual or new lesions is also important. Hemodynamic abnormalities resulting in increased wall stress and ischemia can serve as important triggers for VAs [978]. In particular, significant pulmonary regurgitation after prior transannular patching with subsequent RV enlargement is a common finding in repaired tetralogy of Fallot [979] and might require surgery or transcutaneous valve replacement. Treatment of the underlying hemodynamic abnormalities can reduce the incidence of recurrent VAs by eliminating the triggers; however, the underlying substrate for monomorphic VT in repaired tetralogy of Fallot remains [422]. Accordingly, concomitant intraoperative cryoablation with [402, 979, 980] or without [409, 418] intraoperative mapping has been successfully performed in select patients with tetralogy of Fallot, VT, and an indication for reoperation.

#### Mapping and ablation

**Fig. 12:**
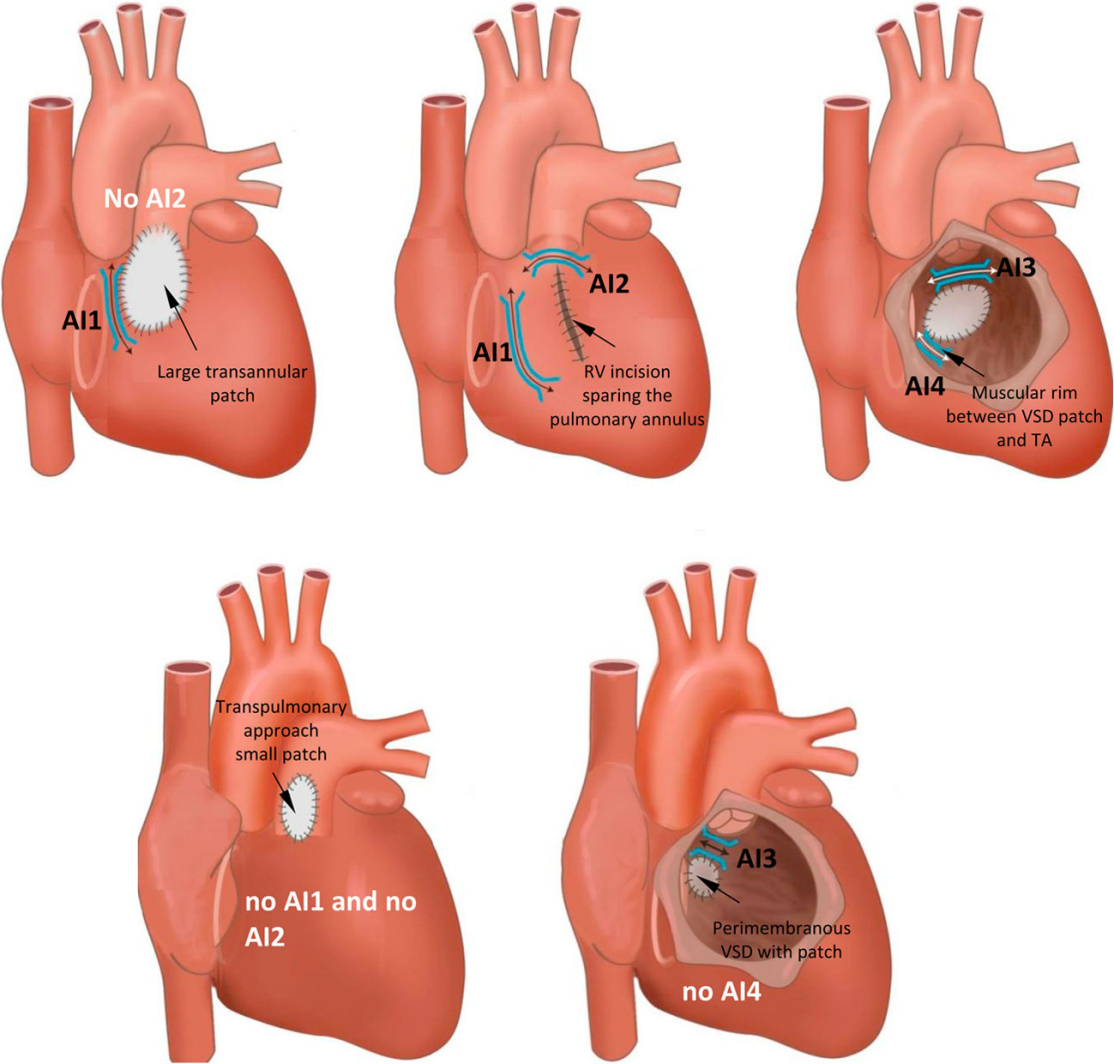
Anatomical isthmuses (AI) in repaired tetralogy of Fallot according to the surgical approach and variation of the malformation. RV = right ventricle; TA = tricuspid annulus; VSD = ventricular septal defect

Most spontaneous and induced monomorphic VTs in patients with CHD referred for ablation are due to macroreentry, with a critical isthmus defined by anatomical barriers, bordered by unexcitable tissue [410, 412, 413, 415, 416, 421]; however, focal mechanisms are occasionally encountered [413, 415, 416]. The boundaries of AIs are the valve annuli and (unlike most other acquired heart diseases) patch material and surgical incisions. Four VT-related AIs in tetralogy of Fallot have been identified: isthmus 1, bordered by the tricuspid annulus and the scar or patch in the anterior RVOT; isthmus 2, between the pulmonary annulus and the RV free wall incision or RVOT patch, sparing the pulmonary valve annulus; isthmus 3, between the pulmonary annulus and the VSD patch or septal scar; and isthmus 4 (which is rarely encountered), between the VSD patch or septal scar and the tricuspid annulus in case of an inferior muscular rim [412] (Fig. 12). Additional AIs bordered by surgical scars and valve annuli have been described after surgery for other CHDs, including complex D-TGA, VSD closure, and surgery for Ebstein’s anomaly [415, 416, 981]. Substrate formation might depend on the coincidence of pathological myocardial remodeling and anatomical boundaries determined by the type and timing of prior corrective surgery. Changes in surgical approaches over the past decades (eg, a combined transatrial-transpulmonary approach avoiding ventriculotomies in tetralogy of Fallot) are likely to affect the incidence and the potential substrate for arrhythmias [982]. Therefore, knowledge of the malformation and careful review of all operation records before ablation is important.

AIs can be reconstructed during sinus rhythm by noncontact mapping [413] or by electroanatomical bipolar voltage mapping combined with high-output pacing (10 mA, 2 ms) at low-voltage sites (<1.5 mV) to identify unexcitable tissue [412, 415]. Noncapture despite good catheter contact is indicative of patch material or surgical scars. VT induction and activation mapping for hemodynamically tolerated VT or pace mapping within anatomically defined isthmuses can be performed to confirm that the AI is critical for sustaining VT [412, 415, 416]. Transection of the AI by connecting the adjoining anatomical boundaries by linear RF lesions can be performed during sinus rhythm [412, 415, 416, 421]. Demonstration of conduction block after transection of the VT isthmus provides a defined procedural endpoint similar to that for achieving block in the cavotricuspid isthmus for atrial flutter and is a valuable acute procedural endpoint combined with noninducibility (see below) [412, 415, 416].

Isthmus dimension and conduction properties likely determine the susceptibility to arrhythmias. In a study that included 24 patients with tetralogy of Fallot, electroanatomical voltage mapping combined with activation mapping during sinus rhythm demonstrated that relatively narrow and slowly conducting AIs (calculated conduction velocity < 0.5 m/s) were the substrate for all 37 documented and induced VTs in patients with preserved cardiac function [113]. These slowly conducting isthmuses can be identified and ablated during sinus rhythm; inducibility of the clinical arrhythmia and hemodynamic tolerance is no longer a prerequisite for successful ablation in patients with AI-dependent macroreentrant VT [113]. Isthmus 3 between the pulmonary annulus and the VSD patch or septal scar is the most common cause of VT and occasionally requires ablation from both the RV and LV sides of the septum or SV for transection [414].

#### Outcome after ablation

Four recent series have reported on ablation outcomes, combining activation mapping with a substrate-based ablation approach and including a total of 99 patients with CHD (82 of 99 had tetralogy of Fallot) [413, 415, 416, 421]. VTs were typically fast, with a median CL of 295–300 ms, requiring a mapping and ablation approach during the underlying baseline rhythm. The definition of complete acute success (noninducibility with or without isthmus block) differed among the studies or changed during the study period [421] but was achieved in 73%–82% of the patients. During a mean follow-up ranging from 33 months to 9.5 years, VT recurred in 12%–32% of the patients after a single procedure and in 5%–11% after repeat ablation [416, 421].

In 2 series of adults with CHD (most with tetralogy of Fallot), successful AI ablation (combined endpoint noninducibility and isthmus block) was achieved in 25 of 34 patients [415] and in 8 of 14 patients [416], respectively. No patients with confirmed conduction block had a recurrence of monomorphic VT during follow-ups of 46 ± 29 [415] and 33 ± 7 months [416], respectively. Accordingly, noninducibility with conduction block across the targeted isthmus is a useful ablation procedure endpoint [412, 413, 415, 416, 421].

Serious procedure-related complications are rare [415, 416, 421]; however, high-risk ablation target sites, including the para-Hisian region with a risk of AV block, need to be considered [113, 411]. Ablation failure can result from withholding ablation due to proximity to the conduction system and coronary arteries, myocardial hypertrophy, and the protection of portions of the AIs by patch material [411, 413, 414, 416]. In particular, a pulmonary homograft can cover parts of the infundibular septum in patients with tetralogy of Fallot, preventing isthmus transection from an RV approach [414]. Although a septal AI can be successfully approached from the LV or aorta after right-sided ablation failure, proximity to the coronary arteries and potential damage to the aortic valve can increase the procedural risk [414]. Accordingly, surgical ablation concomitant with repair of residual hemodynamic abnormalities should be considered in these patients with spontaneous VT, especially if new patch material will prevent postsurgical access to the substrate. For these patients, a preoperative electrophysiology study and EAM can be helpful in guiding surgical ablation [113]. The use of empirical intraoperative linear cryoablation without preoperative or intraoperative mapping, transecting AI III and AI I, has been reported in patients with and without clinical VT, with mixed results [409]. However, empiric linear lesions might not be sufficient. After intraoperative isthmus ablation was performed in 31 patients who were inducible for VA prior to pulmonary valve replacement, 47% remained inducible after surgery [418]. Similar to catheter ablation, preoperative mapping, tailored cryoablation, and intraoperative confirmation of bidirectional conduction block across linear lesions can improve outcomes.

The strong link between a slowly conducting AI and sustained VT [113, 414] in tetralogy of Fallot is intriguing, and preventive ablation of these potential isthmus areas with a demonstration of conduction block may be considered, especially if future surgical procedures might affect the accessibility of these potential VT isthmus areas. Whether this would be beneficial for patients with tetralogy of Fallot remains to be determined and will need to be demonstrated by randomized studies.

### Sarcoidosis

**Key Points**

• The arrhythmogenic substrate in cardiac sarcoidosis is often intramurally located but can include the endocardium and epicardium.

• A CMR is beneficial in planning an ablation procedure in cardiac sarcoidosis.

• The arrhythmogenic substrate can be complex and can include areas of active inflammation and chronic scarring.

• The VT recurrence rate after ablation is high.

Cardiac sarcoidosis is a dynamic infiltrative noncaseating granulomatous disease with periods of inflammation that often culminate in fibrosis [157, 983–985]. Patients with cardiac symptoms and low LVEF fare the poorest among patients with cardiac sarcoidosis in terms of prognosis and mortality. In contrast, patients with asymptomatic cardiac sarcoidosis and normal LVEF have a 10-year survival rate of 89%–100%. Although only approximately 5% of patients with systemic sarcoidosis have symptomatic cardiac involvement, autopsy and imaging studies suggest that asymptomatic cardiac involvement occurs in 25%–92% of patients [157, 986, 987]. Isolated cardiac involvement has been increasingly recognized; a longitudinal study in Finland found that nearly two-thirds of 110 patients with histologically confirmed cardiac sarcoidosis had isolated cardiac involvement. The initial presentation was associated with AV block in 48% of cases and with VT or VF in 38% [986]. The 5-year survival probability for patients with cardiac sarcoidosis treated with immunosuppression has been reported to be as high as 95%, with the greatest benefits seemingly gained by those without severe LV systolic dysfunction (1327, 1331–1333). For patients with cardiac symptoms and low LVEF, the 10-year survival rate is 19%–27% and the cause of death is often heart failure or arrhythmia [157, 986, 987]. These findings suggest that early recognition and treatment could be of value, although there are no randomized trials on this issue. For patients presenting with VT, consideration of cardiac sarcoidosis is important, given the differences in both acute and long-term management, specifically regarding therapy with immunosuppressive drugs [157]. A CMR can be particularly helpful in raising the suspicion of an infiltrative disease process such as cardiac sarcoidosis [988], and obtaining this imaging is recommended in current heart failure guidelines (COR IIa, LOE B) [518]. Importantly, cardiac sarcoidosis can mimic ARVC, and its involvement can be localized to the RV [157, 344, 989–993]. There is substantial overlap in the clinical diagnostic criteria for ARVC and cardiac sarcoidosis, including imaging characteristics, identification of epsilon waves, surface ECG characteristics, including signal-averaged ECG, scar distribution, VT morphologies, and other substrate characteristics identified in intracardiac mapping [157, 344, 989–993]. There are a number of clinically useful findings that can help to differentiate the 2 conditions. In contrast to cardiac sarcoidosis, ARVC rarely involves the septum, and therefore the conduction system is rarely involved in ARVC [992]. The disease process in ARVC also originates in the epicardium, whereas the granulomas in the inflammatory process of sarcoidosis are located within the myocardium and reach the endocardium/epicardium by extension of the intramural lesions [994]. Reaching a cardiac tissue diagnosis is often difficult because granulomatous infiltration can be patchy, and the yield of blind endomyocardial biopsy is <25% [157]. Endomyocardial biopsy targeting areas of abnormal bipolar or unipolar voltage has shown promise for providing greater diagnostic yield [157, 995].

The mechanisms of VT in patients with cardiac sarcoidosis include abnormal automaticity and triggered activity during the inflammatory phase and predominantly scar-related reentry in the chronic phase. Multiple VT mechanisms can be encountered in the same patient [111, 344–346, 570, 996–1001].

When PET scanning identifies active inflammation at the time the VT presents, acute treatment with immunosuppressive agents and AADs may be considered over ablation [157, 570, 983, 992, 1000]. There are limited data supporting the use of steroids or immunosuppressive therapy alone for managing cardiac sarcoidosis-related VT [345, 570, 991]. Regimens have included corticosteroids (prednisolone 1 mg/kg/day or equivalent), along with steroid-sparing agents (including methotrexate, cyclophosphamide, cyclosporine, mycophenolate, and infliximab), for a minimum of 3 months, after which the medication may be tapered based on treatment response [570, 1002]. A number of studies have suggested that immunosuppression reduces the burden of VAs [345, 570, 992, 997], whereas others have either failed to show a benefit or have demonstrated a worsening of VAs [77, 766, 1003]. Data suggest that immunosuppression could be more beneficial for VAs in early disease with preserved LVEF [157]. Nevertheless, the current expert consensus recommendation is to consider assessing the patients for active inflammation using PET scanning and administering therapy with immunosuppressants and AADs if active inflammation is present [157].

Medical therapy, including immunosuppression therapy, has failed for most patients in reports on VT ablation in cardiac sarcoidosis (Table 8) [344, 570, 991]. If urgent imaging is unavailable or the VT persists despite empiric medical treatment, ablation may also be considered [157].

**Table 8:** Catheter ablation of ventricular arrhythmias in cardiac sarcoidosis

Study	N	LVEF, %	Concurrent immunosuppressive therapy, n (%)	VTs induced, mean ± SD	Mapping, Endo n/Epi n	Ablation, Endo n/Epi n	Patients undergoing repeated procedures, n (%)	VT recurrence, n (%)	VT burden decrease, n (%)	Major complications	Follow-up, months
Koplan et al. [570]	8	35 ± 15	5 (63)	4 ± 2	6/2	8/2	1 (13)	6 (75)	4 (44)	NR	6
Jefic et al. [344]	9	42 ± 14	8 (89)	5 ± 7	8/1	NR	3 (33)	4 (44)	9 (100)	NR	20
Naruse et al. [345]	14	40 ± 12	12 (86)	3 ± 1	14/0	14/0	4 (29)	6 (43)	NR	NR	33
Dechering et al. [991]	8	36 ± 19	NR	4 ± 2	NR	NR	NR	1 (13)	7 (88)	NR	6
Kumar et al. [111]	21	36 ± 14	12 (57)	Median 3 (range 1–8)	21/8	21/5	11 (52)	15 (71)	16 (76)	4.7%	24
Muser et al. [346]	31	42 ± 15	22 (71)	Median 3 (range 1–5)	31/11	31/8	9 (29)	16 (52)	28 (90)	4.5%	30

*LVEF* left ventricular ejection fraction, *N* number, *NR* not reported, *VT* ventricular tachycardia

The ablation approach to scar-related macroreentrant VT in cardiac sarcoidosis is similar to that employed in other scar-related VTs (see Sections 8 and 9) and is largely based on identifying the abnormal substrate during sinus rhythm. Activation and/or entrainment mapping during VT is often limited due to hemodynamic instability, multiple VT morphologies, or noninducibility [344–346, 570, 991, 1001]. Substrate-based ablation targets areas with abnormal electrograms (see Section 8) and favorable pace maps (see Section 8) [111, 344–346, 570, 991, 1001]. A feature common to other nonischemic VT substrates is the predilection for involvement of the epicardium (22%–26%) [111, 344, 346, 570] and midmyocardium, the latter of which can be difficult to define in the absence of a CMR or with standard mapping techniques. Granulomatous infiltration can affect any part of the myocardium; cardiac imaging studies with CMR, nuclear perfusion scanning, and PET scanning can therefore be helpful for locating and assessing the burden of the complex substrate [111, 157, 346, 990, 992]. The frequent involvement of the basal septum results in a high prevalence of right septal VTs or VTs involving the Purkinje fiber system [345], and the predominance of a basal substrate gives rise to peritricuspid and perimitral VTs [344, 345]. Nonstandard and investigational adjunctive ablation techniques (see Section 9.1) designed to create larger and deeper lesions have been used to attempt the ablation of intramural substrate when standard ablation fails, including bipolar ablation, needle-facilitated ablation, the use of half normal saline RF electrode irrigation, and transcoronary arterial or venous ethanol ablation [77, 766, 781, 1003].

A number of observational studies have investigated the utility of catheter ablation in patients with cardiac sarcoidosis (Table 8). Outcomes have varied widely, as have the differences in patient characteristics, the concomitant use of immunosuppression, and follow-up times [111, 344–346, 570, 991, 1004]. In general, freedom from any recurrent VT is achieved in 45.8% of cases for up to 2 years of follow-up, with multiple procedures required in 12.5%–43% of patients [111, 344–346, 570, 1004]; however, the overall VT burden has been reduced by 88% [1004]. Major procedural complications have been observed in up to 5% of cases [111, 346, 1004].

### Chagas disease

**Key Points**

• The pathogenesis of ChD is poorly understood but often results in an inferolateral LV aneurysm.

• The arrhythmogenic substrate is located intramurally and on the epicardial surface, often necessitating an epicardial ablation procedure.

#### Chagas disease

ChD is a chronic parasitosis affecting the heart and other organs and is caused by the protozoan *Trypanosoma cruzi*. ChD is transmitted to humans mainly through parasite-laden feces from a hematophagous insect vector found only in the Americas, where the disease in considered endemic. The World Health Organization estimates a prevalence of ChD ranging from 1%–6% in endemic areas, with 10 million people infected worldwide (mostly in Latin American countries), 100 million at risk of infection, and 300,000 new cases reported each year. The World Health Organization estimates that 50,000 CCM-related deaths occur annually, with 60%, 25%, and 15% related to sudden cardiac death, progressive heart failure, and stroke, respectively [1005, 1006].

Cardiac involvement is the most frequent and serious manifestation of chronic ChD. Although the pathogenesis of cardiac damage is complex and not completely understood, at least 4 possible mechanisms have been suggested: cardiac parasympathetic neuronal depopulation, immune-mediated myocardial injury, parasite persistence in cardiac tissue with secondary antigenic stimulation, and coronary microvascular abnormalities causing myocardial ischemia. Diffuse arteriolar dilatation found in CCM can result in a coronary steal phenomenon of blood flow from epicardial coronary arteries to dilated arterioles, producing low perfusion pressure in the distal microvasculature supplying susceptible areas distal to the coronary branches. The watershed areas between main coronary artery branches would be the most susceptible to this steal phenomenon, causing low perfusion pressure, secondary ischemia, microinfarctions, and reparative fibrosis. The most frequently observed areas of myocardial scarring in CCM are the posterolateral LV and the LV apex in the watershed zones between the right coronary and circumflex arteries, and the anterior descending and posterior descending coronary arteries, respectively. VT is typically related to an inferolateral LV aneurysm [1006].

#### Ventricular tachycardia in Chagas cardiomyopathy

Sustained VT is usually due to reentry, associated with an inferolateral LV scar in over 70% of patients. Occasionally, endocardial radiofrequency ablation can result in transmural injury, effectively treating all portions of the myocardium involved in the arrhythmia circuit. However, scars commonly exist intramyocardially and/or subepicardially in an area with an associated thick layer of subendocardial myocardium. Mapping and ablation from the epicardial surface is performed in up to 40% of patients [571, 1007]. A myocardial isthmus of surviving tissue between the inferolateral LV scar and the mitral valve annulus can be involved in a macroreentrant submitral circuit, as has been described for postinfarction VTs [1008]. BBR can occur but is unusual [1009].

In CCM that presents with VT storms, cardiac sympathetic denervation (CSD) can reduce VT episodes and is a potentially valuable treatment option [1010].

#### Epicardial ablation of sustained ventricular tachycardia in Chagas heart disease

The prevalence of epicardial VT origins in patients with CCM is high (approximately 37%). Electrograms obtained during epicardial mapping from the areas giving rise to VT are similar to those observed for other scar-related VTs, including delayed potentials, mid-diastolic potentials, and continuous electrical activity during VT. The critical isthmus of the reentrant circuit can be confirmed by entrainment maneuvers or interruption of VT with RF ablation. With the progressive nature of the disease, VT recurrences are common [1007, 1011].

### Miscellaneous diseases and clinical scenarios with ventricular tachycardia

**Key Points**

• Lamin cardiomyopathy often has a poor prognosis, progressing to end-stage heart failure.

• VT ablation is challenging due to intramural substrates.

• VT recurrence rate is high after ablations.

• VT in patients with noncompaction tends to originate from regions of noncompacted myocardium where scar can be identified in the midapical LV.

• VT ablation in patients with LVAD can be challenging due to the limitation of preprocedural imaging, and the electromagnetic noise generated by the LVAD.

#### Lamin cardiomyopathy

The cardiac phenotype associated with mutations in the *LMNA* gene includes a familial form of DCM with autosomal dominant inheritance and a variety of clinical manifestations ranging from progressive heart failure, AV block to supraventricular block, and VAs [1012–1014]. Malignant VAs are particularly prevalent in these patients and can precede the development of overt DCM [362]. Risk factors for developing VAs include the presence of AV conduction abnormalities, the male sex, and the presence of nonmissense mutations in the *LMNA* gene [362, 1015, 1016]. LV dysfunction has also been reported as a risk factor for developing VAs, although malignant VAs can occur in up to one-third of cases despite the presence of normal LV function [362]. Longitudinal studies assessing the natural history of patients with *LMNA* DCM have consistently described a poor long-term prognosis regardless of the initial clinical presentation, with most patients eventually progressing to end-stage heart failure and the need for advanced therapies including heart transplantation [362, 1012].

The management of recurrent VAs in patients with *LMNA* DCM is challenging owing to the progressive nature of the disease, the variable response to AAD therapy, and the predominant anteroseptal location of the underlying arrhythmogenic substrates, with a high prevalence of intramural substrates. The role of catheter ablation in managing recurrent VT in patients with *LMNA* cardiomyopathy has been recently evaluated in a multicenter registry [110]. Of the 25 patients included in the registry, an anteroseptal substrate was found in 82%, with all patients having multiple inducible VT morphologies. Ablation was challenging, with most patients having residual VT inducible at the end of the ablation procedure. Over a relatively short-term follow-up (median of 7 months), the mortality rate was 26%, 44% of patients were considered for mechanical circulatory support or heart transplantation, and the cumulative recurrence rate was 91%. Of note, a procedure-related complication occurred in 25% of cases, including anticipated complete heart block (in the process of ablating septal substrate), asystole, cardiogenic shock, and thromboembolic events. Given the overall poor success in achieving lasting VT control, the high risk of procedural complications, and the rapid progressive nature of the disease, catheter ablation has only a palliative role for patients with *LMNA* cardiomyopathy and recurrent drug-refractory VAs.

#### Left ventricular noncompaction

LVNC is a rare primary cardiomyopathy likely caused by an arrest of the normal embryogenesis of the endocardium and mesocardium, leading to the formation of prominent trabeculations and deep intertrabecular recesses within the LV wall communicating with the cavity [1017, 1018]. The inferior and lateral walls of the LV from the mid-cavity to the apex are the most commonly involved regions. The clinical presentation of LVNC is highly variable, ranging from completely asymptomatic to end-stage heart failure, and is frequently associated with VAs and thromboembolic events [1017, 1019]. The substrate underlying VAs in these patients is complex and involves pathological myocardial changes consisting of fibrosis, disruption of cellular architecture, and noncompacted myocardium. Reentrant VAs tend to colocalize with the regions of noncompacted myocardium in the midapical LV cavity, and patients can have a unique substrate distribution compared with other forms of NICM, with isolated involvement of the mid to apical LV segments and sparing the perivalvular regions [1020]. However, significant heterogeneity in the VA substrates has been described, and VAs can also arise from regions remote from the noncompacted myocardial segments [1020, 1021]. Small observational studies and isolated reports of patients with drug-refractory VA have shown that catheter ablation can be performed safely and can achieve long-term arrhythmia control [1020–1025]. Owing to the high thromboembolic risk associated with LVNC, long-term oral anticoagulation is usually warranted, and it is important that patients undergo a careful preprocedural imaging evaluation to rule out the presence of intracavitary thrombi, which can be challenging to identify due to the presence of dense trabeculations. Catheter ablation can be performed on uninterrupted oral anticoagulation or with efforts to minimize the time off of oral anticoagulation using periprocedural bridging with heparin [1020].

#### Congenital left ventricular aneurysms

Congenital LV aneurysms and diverticula are rare and believed to be the result of the disrupted embryogenesis of portions of the ventricular myocardium [1026, 1027]. The diagnosis of congenital ventricular aneurysms and diverticula is typically reached through noninvasive imaging studies after ruling out secondary aneurysmal evolution from myocardial insults such as coronary artery disease, inflammatory cardiomyopathies, and trauma. Congenital ventricular aneurysms and diverticula can be asymptomatic or can present with thromboembolic complications, heart failure, myocardial rupture, or malignant VAs and sudden cardiac death [1026–1028]. Monomorphic reentrant VTs can originate from congenital ventricular aneurysms or diverticula [1029–1031], and isolated reports have demonstrated that catheter ablation can achieve arrhythmia control [1029, 1032, 1033].

#### Left ventricular assist devices

Patients with permanent LVADs have a high incidence of recurrent VT, with an estimated incidence of 22%–53%, with the highest incidence in patients with a history of sustained VT prior to LVAD placement [1034–1036]. Recurrent sustained VT has been associated with increased mortality in a select number of studies [1036–1038]. Sustained VT has negative hemodynamic consequences in some patients, possibly due to the deterioration of RV function. Whenever a patient with an LVAD presents with new-onset VA, it is important to rule out mechanical triggers such as mechanical interaction between the inflow cannula and the adjacent apical-septal myocardium, “suction events,” and mechanical pump failure resulting in worsening heart failure [1039, 1040]. These issues can typically be ruled out with a transthoracic echocardiogram, LVAD interrogation, and blood tests for hemolysis, which can be a marker of pump thrombosis. However, most patients presenting with repetitive sustained VAs, particularly those with a history of VAs before the LVAD implantation [1038, 1041], have no identifiable mechanical trigger and usually require therapy to achieve VA suppression. Although antiarrhythmic therapies have not been well studied, an observational study by Raasch et al. [1041] of 61 patients undergoing LVAD implantation reported that 9 of 15 (60%) patients in whom amiodarone was initiated after the occurrence of VAs remained free of recurrent arrhythmias; however, the follow-up duration in this subgroup was not specified. In the patients experiencing recurrent VAs despite therapy with amiodarone, the authors reported no convincing benefits for adjuvant therapy with other antiarrhythmic agents, including sotalol, lidocaine, mexiletine, and procainamide. In particular, mexiletine was added to amiodarone in 4 patients but was effective in only 1.

For recurrent VAs despite AAD therapy, catheter ablation should be considered. Multiple observational studies and a large multicenter registry have shown that catheter ablation can be performed safely and effectively in patients with LVADs [1042, 1043], although there are several important considerations. The preprocedural definition of the substrate can be challenging due to significant imaging artifacts from ICD devices and the LVAD cannula. LVADs that have a magnetically levitated impeller introduce substantial electromagnetic noise that can render the QRS morphology of VT from the body surface ECG uninterpretable. The aortic valve often has minimal or no motion in patients with LVADs, cusp fusion can develop, and, in some instances, the aortic valve is surgically oversewn to abolish aortic insufficiency. The SVs can be sources of thrombi. A transseptal approach to access the LV is usually preferred, and is the only option in some cases [1042], although the resulting atrial septal communication can potentially cause clinically significant right to left shunting, with systemic oxygen desaturation in some patients who have concomitant RV failure and increased right atrial pressure.

Sacher et al. [1042] have reported on a multicenter study of catheter ablation in 34 patients with LVADs who underwent 39 procedures (25 with a transseptal and 14 with a retrograde aortic approach). Of note, the targeted VT was related to the LVAD cannula in only 9% of the cases, whereas the remaining VAs were associated with the underlying disease substrate. After a mean follow-up of 25 ± 15 months, 7 patients underwent heart transplantation, 10 died, and 13 (76%) of the remaining 17 patients remained free from recurrent VT. These results are in line with other single-center observational series [852, 1043–1047] and support the benefits of catheter ablation as an important treatment option for patients with LVADs and recurrent VT.

### Surgical therapy

**Key Points**

• Surgery-facilitated access to the epicardium via a limited subxiphoid incision can be helpful in the case of adhesions.

• Cryoablation via thoracotomy is possible for posterolateral substrates and via sternotomy for anterior substrates.

In some patients, standard endocardial approaches combined with percutaneous epicardial catheter ablation remain ineffective in providing VA control due to deep intramural circuits [743]. Percutaneous epicardial access might not be feasible for patients with prior pericarditis or cardiac surgery, and epicardial VTs might not be easily targeted using standard ablation approaches [559]. Alternative treatment methods need to be considered for patients with failed endocardial and epicardial ablation procedures [742]. The threshold for a surgical approach might be lower if a concomitant surgical procedure is indicated. For patients with an inaccessible epicardial space, percutaneous catheter ablation in the electrophysiology laboratory can be facilitated by a surgically created epicardial window allowing entry into the pericardial space [562]. This approach is most straightforward in patients with an apical or inferior VA substrate, because the area of the heart closest to the window is most easily accessed, and pericardial adhesions can limit extensive mapping. Surgical cryoablation can also be conducted in the operating room utilizing a lateral thoracotomy or sternotomy [332, 1048]. Thoracotomy is the preferred approach to lateral and posterior substrates, whereas sternotomy is often needed for anterior substrates. Portable EAM can be utilized to help to localize the substrate, and VT can be inducible when the patient is off the cardiopulmonary bypass. When possible, preprocedural planning should include CT imaging of grafts for patients with prior bypass surgery to prevent injury to these structures. The use of surgical VT ablation has also been described in patients undergoing percutaneous LVAD implantation [1049, 1050]. Recently, there has been growing concern regarding the increased rates of LVAD thrombosis among patients who undergo both endocardial and epicardial surgical cryoablation at the time of LVAD implantation [1051]. In highly selected patients with epicardial LVOT VAs refractory to standard techniques due to overlying epicardial fat and/or proximity to coronary arteries, open [816] or minimally invasive [817, 818] approaches have been used. The full characterization of the efficacy and safety of these approaches is pending.

### Sympathetic modulation

**Key Points**

• Sympathetic modulation targeting the stellate ganglia by video-assisted thoracoscopy may be considered for failed VT ablation procedures or VF storms.

• A temporary effect can be obtained with the percutaneous injection or infusion of local anesthetics.

The autonomic nervous system (ANS) has a central role in regulating heart rate and cardiac function and contributes to the pathogenesis of VA in structurally normal hearts, abnormal hearts, and channelopathies [1052]. The ANS includes the parasympathetic component mediated by the vagus nerve and a sympathetic component mediated by cervicothoracic paravertebral sympathetic ganglia. The ANS undergoes remodeling and becomes dysfunctional in SHD [1052]. In most situations, ANS imbalances contribute to the onset and/or maintenance of VAs, especially through the activation of efferent sympathetic pathways. Interventions that decrease sympathetic tone are often beneficial.

Sympathetic activation and vagal withdrawal are associated with diminished respiratory sinus arrhythmia and heart rate variability. Iodine-123 metaiodobenzylguanidine and 11C-meta-hydroxyepinephrine imaging provide an indication of sympathetic nerve distribution and local noradrenaline reuptake that reflects sympathetic activity [1052]. Although iodine-123 metaiodobenzylguanidine and 11C-meta-hydroxyepinephrine [1053] imaging findings are associated with VA in various settings, the clinical utility of using these findings for risk stratification and to guide therapy has yet to be proven [1052].

Beta blockers are a first-line therapy for VA sensitive to ANS influences. Beta blockers often decrease idiopathic RVOT PVCs in healthy individuals [1054] and reduce the rate of sudden cardiac death after MI [1055, 1056]. Conversely, in electrical storms due to Brugada syndrome or idiopathic VF, beta-adrenergic stimulation by isoproterenol infusion can abolish recurrent arrhythmia [1057, 1058].

Transient CSD achieved by high thoracic epidural anesthesia with bupivacaine administered at the T1–T2 or T2–T3 level into the epidural space can be employed as a bridge to more definitive therapy [1059]. An 80% reduction in VA episodes was observed in 6 of 8 patients undergoing thoracic epidural anesthesia, with infusion durations ranging from 48 to 96 h [1059]. Partial denervation can be achieved by percutaneous stellate ganglion block and can also quiet post-MI electrical storms [1056]. A recent meta-analysis of nonrandomized studies found that temporary percutaneous stellate ganglion block was associated with a major acute reduction in the burden and supports its potential use as a bridge to more definitive therapy [1060].

Persistent CSD is achieved at open surgery (or by video-assisted thoracoscopy) by resecting the lower third to half of the stellate ganglia and the T2 to T4 or T5 thoracic ganglia, as well as transecting the nerve of Kuntz when present [1061]. Potential complications include Horner syndrome, Harlequin syndrome, dyshydrosis, and regional temperature changes [1062, 1063]. In a series of heterogeneous patients with drug-refractory VA storms undergoing unilateral or bilateral surgical CSD, ICD shocks were reduced by 90% in 90% of the patients, with left CSD less efficient (50% shock free) than bilateral CSD [1061, 1064, 1065]. CSD has also been shown to be effective in CCM [1010]. However, denervation through stellate ganglion ablation surgery is not always complete, and VA can recur. Nonetheless, in patients with VA storms for whom beta blockers, antiarrhythmic medications, and catheter ablation are ineffective or not tolerated, CSD can be considered a reasonable option, provided the treating physicians have expertise with these techniques [1010, 1055, 1064].

Surgical CSD can be considered for high-risk patients with contraindications for beta blockers, for patients with symptomatic long QT syndrome when beta blockers are not effective, or when ICD therapy is contraindicated or declined [1062, 1063, 1066–1068]. CSD is associated with a significant reduction of events in patients with high-risk long QT syndrome, with a reported 50%–80% long-term success rate [1067–1071]. A significant decrease in QTc duration is usually observed after CSD ([387, 1067].

CSD can also be considered for patients with catecholaminergic polymorphic VT and recurrent symptomatic VA (syncope or ICD shocks) while undergoing optimal drug therapy with beta blockers and flecainide and for patients with a contraindication for beta blockers [1063, 1072, 1073]. In catecholaminergic polymorphic VT with recurrent VA despite optimal therapy, CSD achieved 80% event-free survival at 2 years and has been advocated as an alternative to an ICD [1071].

Spinal cord stimulation of T1 to T5 with an external stimulator appears to modulate autonomic activity, possibly via both sympathetic inhibition and an increase in vagal activity [1053]. Spinal cord stimulation reduced VA episodes 75%–100% in a small number of patients with cardiomyopathy and high VA burden [1067]. The spinal cord stimulation mechanism is not completely understood, and the long-term clinical effects and safety have not been studied in humans. Low-level stimulation of the cervical vagosympathetic trunks or carotid body can antagonize proarrhythmic sympathetic surges and decrease VAs in animal models, but there is no experience for this with humans [1053].

Renal denervation decreases both central sympathetic activity and systemic catecholamine excretion, possibly mediated through either afferent or efferent renal sympathetic nerves and independent of the effect on blood pressure [1053, 1074]. In small series or isolated cases with various cardiomyopathies and refractory VA, catheter-based percutaneous renal denervation was observed to reduce VAs and appeared safe [1075–1078]. Techniques and acute procedural endpoints need further evaluation, and randomized trials are needed to assess the efficacy and safety.

### Endpoints of catheter ablation of ventricular tachycardia

**Key Points**

• Noninducibility of VT by PES after ablation is a reasonable endpoint and predictor for VT recurrence after VT ablation in patients with SHD.

• Due to the limitations of programmed stimulation, endpoints other than noninducibility have been described, including elimination of excitability, elimination of LPs or LAVA, dechanneling, substrate homogenization, core isolation, image-guided ablation, and anatomically fixed substrate ablation.

#### Historical perspective

In the early days of electrophysiology testing to study arrhythmias, PES was recognized as a tool for evaluating the efficacy of treatments (at that time, drugs and surgical ablation). For both supraventricular arrhythmia and VA, elimination of inducible sustained tachycardia following drug or surgical intervention, when the arrhythmia had been reproducibly induced with PES prior to intervention, correlated with successful treatment at least by the short-term follow-up [19, 1079]. PES was known to be a somewhat unreliable assessment tool in that 1) it did not perfectly predict outcomes (sensitivity or specificity) and 2) significant day-to-day variability in the results was demonstrable in the absence of an intervention [1080]. In addition, varying definitions of “noninducibility” have been used, including noninducible VA of any type using up to triple extrastimuli from 2 RV sites and 2 drive CLs; initiation of only “nonclinical” VTs that had morphologies and/or CLs that had not been previously observed spontaneously; and rendering a previously easily inducible VT no longer inducible at that step of the PES protocol but without performing the complete PES protocol. Despite these shortcomings, VA noninducibility was regarded for over 2 decades as the standard means for assessing the efficacy of therapeutic interventions, including catheter ablation [629, 633].

#### Programmed electrical stimulation

PES at the end of the ablation procedure is still employed and remains a reasonable predictor of VT recurrence. In particular, patients in whom relatively slow VTs (CL >300 ms) remain inducible at the end of the procedure are more likely to experience recurrence than those in whom no VT (or only rapid, unmappable VT) is inducible [1081]. In a recent study, inducible nonclinical VT that has a CL longer than the RV effective refractory period plus 30 ms was associated with recurrent VTs, whereas faster inducible nonclinical VTs were not associated with recurrence [892]. A meta-analysis of post-MI VT ablation studies found that the absence of inducible VT was associated with a substantially lower risk of recurrent VT compared with the inducibility of nonclinical VT (odds ratio [OR] 0.49) and clinical VT (OR 0.10) [633].

The predictive capacity of immediate postablation PES was recently challenged by Frankel et al. [636], who have shown that NIPS using the patient’s ICD a few days after the ablation procedure yielded a more accurate picture of the likelihood of VT recurrence after ablation than the acute end-of-procedure stimulation protocol. In this study, 18% of patients without inducible VT at the end of the ablation procedure had inducible clinical VT using subsequent NIPS, and another 37% had inducible nonclinical VT. Each group had shorter recurrence-free survival than those without inducible VT at delayed NIPS. Similar results were recently reported by Oloriz et al. [1082]. Numerous potential causes of postablation recurrent VT episodes have been proposed, including ablation lesion healing, development of new circuits, withdrawal of AADs that had suppressed some VTs, and emergence of VTs at the periphery of ablation zones [896]. The optimal management of patients found to have inducible VT has not yet been defined.

#### Current ablation strategies and assessment of results

For focal arrhythmias (such as nonsustained or sustained VT or PVCs, typically observed in individuals without SHD), activation and/or pace mapping are generally adequate guides for ablation (assuming the arrhythmia was present spontaneously or inducible prior to ablation). Assessment of efficacy is termination of VT, or elimination of PVCs, and subsequent noninducibility of VT or PVCs by catecholamine infusion or electrical stimulation that had reliably provoked episodes prior to ablation.

For patients with SHD in whom reentry is the primary VT mechanism, the effectiveness of ablation has usually been assessed by PES (typically through triple ventricular extrastimuli at 1 or 2 RV sites and 1–2 drive CLs), with or without an adjunctive catecholamine infusion. In addition to these mapping tools, a variety of substrate-based strategies are currently in use for ablation for VA, each with their own procedural endpoints (almost all combined with end-of-procedure PES). A short description of these strategies is provided below [683, 1083]. In many of these studies, substrate ablation was combined with ablation guided by activation and entrainment mapping when a hemodynamically tolerated VT was inducible. For all of these approaches, data are largely limited to small series from single centers or groups of investigators.

1. Elimination of electrical excitability [42]: Targeting VT isthmuses between areas of electrically unexcitable scar and ablation to the extent that isthmus tissue cannot be stimulated with unipolar pacing output of 10 mA (the endpoint of this strategy) has been shown to result in fewer VT recurrences in follow-up. This approach has the drawbacks of contact dependency and time expense, especially in cases with more extensive areas of ablation in which repeated cycles of stimulation and ablation can be tedious and time-consuming.
2. Elimination of LPs [38]: Elimination of all recordable LPs (high-frequency electrogram components inscribed after the end of the surface QRS complex that reflect slowed conduction or block) has been shown to improve freedom from recurrent VT compared with cases in which LPs were not eliminated [1084]. This approach is limited by sampling density and the time required for remapping to assess the absence of LPs.
3. Elimination of LAVAs [684]: LAVAs are defined as electrograms with 2 or more distinct components that might be evident in the resting rhythm or can be revealed by a stimulation wavefront arriving from a different direction. LAVAs thus include standard LPs but also the split potentials (those with an isoelectric interval between components of at least 30 ms) that are not late and even some normal-appearing electrograms for which stimulation might reveal previously unobserved abnormalities. Whereas elimination of LPs has generally been performed on the endocardium, LAVA mapping and ablation requires endocardial and (when possible) epicardial mapping and ablation. Elimination of all LAVAs (the endpoint of this strategy) has been correlated with high rates of freedom from spontaneous recurrence of VT. Problems with this approach include the lack of standard programmed stimulation methods to detect LAVA at sites without these potentials during sinus rhythm, the extent of ablation needed to treat all LAVAs (endocardial and epicardial ablation, the latter might not always be feasible), and the inability to know when all LAVAs had been ablated successfully (sampling bias). Additionally, the mapping catheter used can affect LAVA detection, with small, closely spaced electrodes detecting LAVA and LP more readily than standard ablation electrodes. However, this might necessitate double access to the chamber in question or repeated catheter exchanges.
4. Dechanneling: Using this method, EAM is used to identify sites with high-frequency delayed (not necessarily “late”) potentials inscribed after a far-field component in sinus rhythm and targets those with relatively short intervals from potential entry sites into channels within scars that can participate in reentry. Endpoints of this “dechanneling” approach are the elimination of delayed potentials or reversal of the activation sequence of delayed potentials when recorded with multipolar catheters. VT-free outcomes were better when complete dechanneling could be affected [46].
5. Substrate homogenization [619]: This strategy is similar in principle to LP and LAVA ablation but more broadly targets the entire region in which low-amplitude (<1.5 mV bipolar) abnormal electrograms are observed with very extensive ablation on both the endocardium and epicardium when abnormal epicardial substrate is identified. The endpoint is electrogram elimination (amplitude reduction to the background noise level or below) and noninducibility of VT. In a small randomized trial of patients with prior MI and a nonrandomized series of patients with post-MI and NICM, VT recurrence was less likely with extensive substrate ablation than with ablation guided by activation and entrainment mapping targeting only the clinical VT [358, 619]. However, it is not known whether this technique would be superior to the targeting of clinical VT(s) plus more limited substrate ablation using the other described techniques. This approach necessarily requires extensive ablation (with more fluid volume administered if irrigated ablation is employed) and potentially longer procedural times. The technique is not applicable to diseases that do not have identifiable low-voltage scar regions.
6. Core isolation [50, 688]: The principle underlying this strategy is that endocardial VT circuits can be somewhat “compartmentalized” by layers of midmyocardial scar tissue, such that conduction is largely spread on the endocardial surface. The encircling of the region with abnormal electrograms that contain the VT circuits need only reach the midmyocardial barrier to isolate the area (ie, damage need not be transmural). In the study by Tzou et al. [50], core isolation was usually achievable and was associated with improved freedom from VT recurrence compared with a nonrandomized reference group. This approach is not always feasible and requires extensive ablation in some patients. The extent to which these core elements reconnect with healing of the ablation lesions and resolution of the edema is unknown.
7. Imaging-guided lesion assessment: Estimating the acute extent (particularly depth) of ablation-related damage, particularly in normal ventricular tissue, has been shown to be feasible with intracardiac ultrasound [1085], CMR [1086], near-infrared spectroscopy [1087], and by elevations in the pacing threshold [1088], as well as with other methods. Lesion size assessment has promise for future applications as an indicator of the adequacy of ablation but has not been studied in detail as a procedural endpoint.
8. Identification of a largely anatomically fixed substrate during baseline rhythm by pace mapping [36] and by locating isolated potentials [38] has been beneficial in identifying a critical isthmus. Targeting the arrhythmogenic substrate by combining pace mapping and electrogram mapping has further demonstrated a reduction in VT recurrence in patients post infarction [39].

As noted, most strategies also use PES to assess for VT inducibility at the end of the procedure. It is important to note that the assessment of efficacy of these ablation strategies is predicated on the assumption that ablation lesions have permanent effects, which cannot be known.

#### Summary

The preponderance of experience for assessing the results of VT ablation is with PES, despite its inherent limitations. Multiple methods of substrate ablation have been described, each with its own procedural endpoint (but almost always with PES assessment at the end of the procedure). Several observations can be made based on available data:

1. Some method of assessing success (prespecified endpoints) should be employed at the end of any ablation procedure for treating VA, insofar as possible (constrained by patient safety considerations). Scar-based reentrant VT usually incorporates PES using 3 extrastimuli at 2 drive CLs from 1 or more RV stimulation sites or at least as vigorous a stimulation protocol as was required to initiate arrhythmia prior to ablation, as well as achieving endpoints particular to whichever substrate-based ablation strategy is used.
2. If a “clinical” (spontaneously occurring) VT remains inducible at the end of the procedure, the likelihood of postprocedure recurrence is high.
3. Nonclinical VTs induced at the end of an ablation procedure have a recurrence rate during follow-up lower than persistently inducible clinical VTs but higher than if no VT is induced at the end of the ablation procedure. Clinicians should carefully consider the risks and benefits of targeting these nonclinical VTs, which entail longer procedure time and potential risk.
4. It is reasonable to consider using NIPS 2–3 days after ablation to refine the prognosis (see Section 10.4). If clinical VT can be initiated at that time, repeat ablation may be considered, although the risks and benefits of this approach have not been defined.

## Postprocedural care

### Postprocedural care: Access, anticoagulation, disposition

#### Postprocedural care: Access

**Table:**
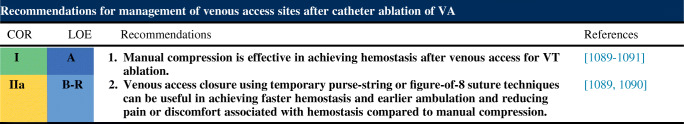

##### Recommendation-specific supportive text

1. Although little has been written on postprocedural management of access sites specifically after catheter VT ablation, practices have been adapted by extrapolating results from other procedures requiring femoral venous or arterial access. Manual compression has been the standard of care for achievement of hemostasis after venous sheath removal. In randomized studies of other electrophysiology procedures comparing manual compression with temporary suture closures or a vascular closure device, comparable high rates of hemostasis had been achieved [1089–1091]. Extrapolating these results to ventricular ablation procedures, manual compression is effective in achieving hemostasis, though time to hemostasis can be expected to be shorter with temporary suture techniques compared with manual compression [1089–1091].
2. The use of a temporary purse-string or figure-of-8 suture that can be removed after achieving hemostasis has been studied after venous access in other ablation procedures, such as for AF, in which procedures are routinely performed on uninterrupted anticoagulation and/or with large-bore catheters [1092–1096]. Compared with manual compression, temporary suture techniques can yield faster hemostasis and ambulation times and reduce the pain or discomfort associated with hemostasis. Two randomized studies have shown shorter times to hemostasis and ambulation, with one reporting reduced rates of access complications compared with manual compression [1089, 1090].

Vascular closure device-based methods for venous closure have not been specifically studied for electrophysiology procedures [1097–1099]. One randomized study compared manual compression to an extravascular closure system that delivers resorbable polyethylene glycol sealant external to the vessel at the sheath access point in 208 patients with a 5, 6, or 7 Fr sheath in the common femoral vein [1099]. There were no vascular complications in either arm. Time to hemostasis was significantly shorter in the device arm (0.12 ± 0.89 vs 7.6 ± 5.7 min; *P* < .001).

When the writing committee was surveyed regarding venous access hemostasis, all the respondents used manual compression, approximately half (53%) used temporary figure-of-8 or purse-string sutures some of the time, and only 1 (6%) had used a vascular closure device. Given that venous access complications are driven primarily by insertion difficulties or concomitant arterial access, reduction in venous access complication outcomes might be best achieved with methods such as use of ultrasound at insertion, rather than by device- or suture-based methods.

**Table:**
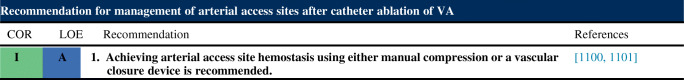

##### Recommendation-specific supportive text

1. Arterial access after VT ablation is typically closed by manual compression or use of vascular closure devices, with comparably high success rates in achieving hemostasis. Though not specifically studied for VT ablation access closure, manual compression and vascular closure device use have been extensively studied for coronary or structural intervention procedures. Several systematic reviews and randomized trials have demonstrated shorter time to hemostasis, with little need for compression, shorter time to mobilization, and lower hematoma rates with use of vascular closure devices for arterial closure compared with manual compression, with no differences in vascular injury, thrombosis, or infection [1100, 1101]. Extrapolating these results to arterial closure after VT ablation, vascular closure device use could reduce the time to hemostasis and ambulation and reduce the risk of hematoma, compared with manual compression. Vascular injury rates for pseudoaneurysm, dissection, or arteriovenous fistula might be more reflective of insertion difficulties rather than of closure methods.
  Use of vascular closure devices is generally avoided if the arteriotomy site is at or distal to the common femoral bifurcation, if contamination of the sheath is suspected, or if the posterior wall of the artery is suspected to have been punctured.
  When the writing committee was surveyed regarding arterial access hemostasis, 42% of the respondents had used manual compression 100% of the time, 29% had used temporary figure-of-8 or purse-string sutures 1%–25% of the time, and 67% had used a vascular closure device at least some of the time (24% had used a vascular closure device over half the time).

**Table:**
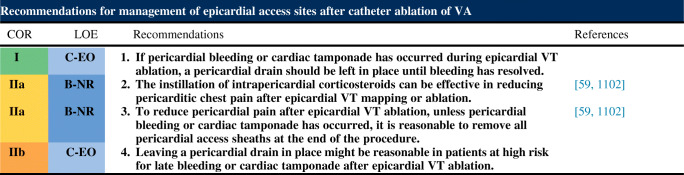

##### Recommendation-specific supportive text

1. If pericardial bleeding or cardiac tamponade has occurred during the procedure, the pericardial drain should ideally be left in place until there is minimal drainage output. This often requires observation in an intensive care unit and follow-up echocardiograms to assess for residual or loculation of fluid.
2. Epicardial access for VT ablation can be associated with postprocedure pericarditic pain and acute pericarditis. The use of steroids can reduce the incidence of postprocedure pericardial chest pain. In an animal model, triamcinolone 2 mg/kg significantly attenuated inflammation and postprocedure inflammatory adhesion formation after epicardial mapping and ablation [1103]. Della Bella et al. [59] compiled the epicardial VT ablation experience of 6 European high-volume VT ablation centers. Of 218 patients, postprocedural precordial pain occurred in 21% and was considered severe in half of these. Oral steroids were used by 1 center routinely after 2007; 1 center routinely used intrapericardial steroids; and 4 centers did not use steroids. Dyrda et al. [1102] retrospectively evaluated the use of 3 therapeutic approaches on the incidence of pericarditis and AF after epicardial mapping and ablation for VT in 85 cases. The approach evolved over time from no steroids to systemic oral or intravenous steroids (1 mg/kg/day for 3 days) to intrapericardial steroids (triamcinolone acetate 2 mg/kg, injected into the pericardial space via a pigtail catheter and left in place by capping of the pigtail). Compared with no steroids, the incidence of pericarditic chest pain was lower with intrapericardial steroids (21.1% vs 58.8%; *P* = .006), but not with intravenous or oral steroids (43.4% vs 58.8%, *P* = 0.31). No difference was found in the occurrence of ECG findings for pericarditis with steroid therapy (36.8%, 30.0%, and 41.2% for intrapericardial steroids, intravenous or oral steroids, or no steroids, respectively), and a nonsignificant reduced incidence of chest pain with ECG changes was found with steroid use (13.2%, 10.0%, and 29.4% for intrapericardial steroids, intravenous or oral steroids, or no steroids, respectively).
  Among the writing committee members, 75% reported instilling steroids in the pericardial space after epicardial mapping/ablation, with 2 reporting this practice only after extensive epicardial ablation. Methylprednisolone, triamcinolone, or triamcinolone acetate were used by 40%–50%, and 9% had used dexamethasone. Only 8% instilled lidocaine into the pericardial space after ablation. In the absence of bleeding or tamponade, 38% leave a pericardial drain in place after epicardial ablation, usually for 8–24 h (89%) or > 24 h (11%).
3. Whether or not to leave a pericardial drain in place after epicardial ablation can be a difficult decision and is based on concerns for late cardiac tamponade. However, Della Bella et al. [59] noted that precordial pain could be due to the pigtail left in the pericardium for continuous drainage. In the Dyrda et al. [1102] study the pigtail catheter was left in place <3 h in 8% and ≥ 24 h in 44%. More chest pain occurred if the pigtail was left in place ≥24 h (51% vs 25%; *P* = .012); however, ECG changes were noted less frequently (19% vs 48%; *P* = .006).
4. In deciding whether to remove the pericardial drain, leaving a guidewire in the pericardial space after pericardial sheath removal and observing several minutes with intracardiac or transthoracic echo might be useful. This can exclude a “through and through” puncture of the RV, which will present with early significant hemorrhage after sheath removal.
  A pericardial drain may be left in place after epicardial ablation based on concerns for late cardiac tamponade. Della Bella et al. [59] noted that 4 of 8 patients with cardiac tamponade occurred late, which provided the rationale for leaving a pericardial drain in place. In the study by Dyrda et al. [1102], postprocedure pericardial effusion or tamponade occurred in 13 patients, observed 18 ± 14 h after the procedure. There were 4 cases of severe pericardial bleeding, occurring acutely in 2 and delayed in 2. The delayed cases were given low-molecular-weight heparin or heparin plus dual antiplatelet therapy.
  If the drain is left in place, longer duration can be associated with pericarditic chest pain (see above), and removal of the drain within 24 h might reduce the incidence of pericarditic chest pain.

#### Atrial fibrillation after epicardial ventricular arrhythmia ablation

The use of amiodarone might be reasonable to lower the risk of new-onset AF after epicardial VT ablation in patients with evidence of acute pericarditis. AF is reported to occur in 4.1%–19.5% [1103–1105] of patients after epicardial VT ablation and tends to occur more commonly in those with signs of pericarditis. In the study by Dyrda et al. [1102], AF occurred in 8.3% of patients with no prior history of it. Median time to new-onset AF was 36 h. Patients with pericarditic ECGs tended to be at greater risk of AF (16.7 vs 3.6%; *P* = .091). Mahapatra et al. [1105] reported a new AF incidence of 19.5%, and all had clinical symptoms of pericarditis. New AF was associated with younger age, longer epicardial ablation time (424 ± 169 vs 867 ± 450 s; *P* < .001), longer epicardial mapping time (103 ± 28 vs 135 ± 51 min; *P* = .02), RV puncture (9.1% vs 50.0%; *P* = .02), and pericarditis pain score at 24 h (1.58 ± 0.79 vs 2.25 ± 0.16; *P* = .03). Prolonged drainage >24 h was not associated with AF incidence (*P* = .28). The occurrence of pericarditis renders anticoagulation decisions challenging; however, the use of steroids was not associated with a lower incidence of AF after epicardial VT ablation in these studies [1103, 1105]. In contrast, the use of amiodarone was associated with lower rates of AF in this population (87.9% vs 12.5%; *P* < .001 in the study by Mahapatra et al.) [1103, 1105]. New-onset AF tends to be paroxysmal and self-limited after epicardial VT ablation. Amiodarone was used by only 1 writing committee member (4%) for AF prophylaxis after epicardial ablation. The writing committee did not feel there was sufficient evidence to make a recommendation about amiodarone use in this setting.

#### Postprocedural care: Anticoagulation

**Table:**
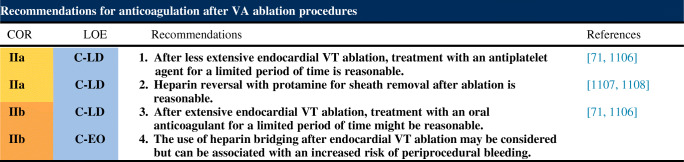

##### Recommendation-specific supportive text

1. Antiplatelet therapy for less extensive endocardial VT ablation appears to be safe, with no significant bleeding nor thromboembolism risks. The Multicenter Thermocool Ventricular Tachycardia Ablation Trial has recommended antiplatelet therapy with aspirin 325 mg/day or anticoagulation with warfarin for 3 months after ablation, if ablation has been performed over an area with >3 cm between ablation sites [71]. No procedure-related thromboembolic complication or stroke had been detected by neurological examination. In the study by Siontis et al. [1106], in patients with less extensive ablation, antiplatelet agents with full-dose aspirin or clopidogrel plus aspirin were used at the physician’s discretion instead of therapeutic anticoagulation. Among 24 patients discharged on only antiplatelet agents (4 low-dose aspirin, 9 full-dose aspirin, 1 clopidogrel, 10 combination of aspirin and clopidogrel), no definite or possible thromboembolic events and no bleeding events were documented in the first 3 months. Among the writing committee, over 90% prescribe either antiplatelets or anticoagulants after endocardial VA ablation, with various criteria used to decide which. When antiplatelets were used (almost exclusively aspirin), the most common duration was 4 to 6 weeks.
2. Anticoagulation during left-sided VT ablation procedures is generally reversed for sheath removal, typically with protamine. In a retrospective cohort study of 158 patients undergoing RF catheter ablation, including 11 for VAs, 116 received protamine and 42 did not [1107]. No significant difference in thrombotic events was observed between groups (one pulmonary embolism in the protamine group and 0 thrombotic events in the control group). Among 150 patients undergoing AF ablation, a randomized trial reported that compared to control, protamine reversal of heparin led to a trend toward shorter duration of manual compression (20 ± 9 vs 24 ± 16 min; *P* = .06) and a shorter time to ambulation (316 ± 80 vs 480 ± 92 min; *P* < .001), with no differences in vascular access complications or thromboembolic events [1108].
3. After extensive endocardial VT ablation, use of anticoagulation for a period of time has frequently been included as part of postprocedural practice, although there are no comparison studies. In the Multicenter Thermocool Ventricular Tachycardia Ablation Trial, antiplatelet therapy with aspirin 325 mg/day or anticoagulation with warfarin was administered for 3 months after ablation if ablation was performed over an area with >3 cm between ablation sites [71]. No procedure-related thromboembolic complication or stroke was detected. The anticoagulation regimens used in other major prospective clinical trials are described in Table 9. Siontis et al. [1106] evaluated an anticoagulation protocol after VT ablation of large LV endocardial ablation areas (>3 cm between ablation sites) in 217 patients with infarct-related VT without evidence of procedure-related pericardial effusion. Starting 8 h after access site hemostasis, an infusion of low-dose, slowly escalating unfractionated heparin 600–900 U/h was administered for 6 h, along with initiation of warfarin. This was followed by 3 months of anticoagulation, or longer if another indication was present. If a direct oral anticoagulant had been administered prior to the procedure, it was resumed as early as 48 h after sheath removal if there was no bleeding. With this regimen, in-hospital bleeding occurred in 6% and an arterial thromboembolic event occurred in 1 (0.6%) patient who had received bridging anticoagulation. Among the 214 patients discharged, 89% were prescribed systemic anticoagulation, and no definite or possible thromboembolic events were documented in the first 3 months; 1 patient had a major bleeding event. Of the writing committee members, approximately two-thirds initiate anticoagulation after LV endocardial ablation in some patients, generally after more extensive lesions. The postprocedural anticoagulation protocols in the major prospective studies of VT catheter ablation are outlined in Table 9.
4. Heparin bridging to oral anticoagulation or ambulation has been practiced but can be associated with a small risk of periprocedural bleeding after VT ablation. Among patients bridged with low-molecular-weight heparin to oral anticoagulation after VT ablation, Siontis et al. [1106] reported in-hospital bleeding in 6% of the patients. Of the two-thirds of writing committee members who initiate anticoagulation after endocardial LV ablation, 60% initiate heparin until this has been established, and a further 20% if starting warfarin until the international normalized ratio is >2, but not if starting a direct oral anticoagulant. See Section 5.5 for further discussion of this topic, including the writing committee’s practices.

**Table 9:** Postprocedural care in prospective studies of ventricular tachycardia catheter ablation

Study	Postprocedure NIPS	AAD type	AAD duration	Follow-up	ICD programming	Anticoagulation post ablation	Bleeding and thromboembolic events (ablation arm)
Calkins 2000 [70]	No	Patients were continued on the type of antiarrhythmic therapy they had received before ablation.	At least the first 3 months after hospital discharge	Evaluation at 1, 3, 6, 9, 12, and 24 months after ablation	Not specified	Not specified	Four of 146 (2.7%) stroke or TIA, 4 (2.7%) episodes of pericardial tamponade
SMASH-VT 2007 [316]	No	No patient received an AAD (other than beta blockers) before the primary endpoint was reached.	N/A	Followed in the ICD clinic at 3, 6, 9, 12, 18, and 24 months; echocardiography at 3 and 12 months	Not specified	Oral anticoagulation 4–6 weeks, aspirin if fewer than 5 ablation lesions	One pericardial effusion without tamponade, managed conservatively; 1 deep venous thrombosis
Stevenson 2008 [71]	No	The previously ineffective AAD was continued for the first 6 months, after which time drug therapy was left to the discretion of the investigator.	Six months, after which time drug therapy was left to the discretion of the investigator	Echocardiogram and neurologist examination before and after ablation; office visit at 2 and 6 months, with ICD interrogation where applicable	Not specified	Three months with either 325 mg/day aspirin or warfarin if ablation had been performed over an area over 3 cm in length.	Vascular access complications in 4.7%; no thromboembolic complications
Euro-VT 2010 [309]	No	Drug management during follow-up was at the discretion of the investigator.	Drug management during follow-up was at the discretion of the investigator.	At 2, 6, and 12 months, with ICD interrogation where applicable	Investigators were encouraged to program ICD detection for slow VT for at least 20 beats or 10 s to allow nonsustained VT to terminate before therapy is triggered.	Not specified	No major bleeding or thromboembolic complications
VTACH 2010 [317]	No	Discouraged	Discouraged	Every 3 months from ICD implantation until completion of the study	VF zone with a cutoff rate of 200–220 bpm and a VT zone with a cutoff CL of 60 ms above the slowest documented VT and ATP followed by shock.	Not specified	One transient ischemic ST segment elevation; 1 TIA
CALYPSO 2015 [318]	No	Discouraged	Discouraged	At 3 and 6 months	Investigators were required to ensure that VT detection in the ICD is programmed at least 10 beats below the rate of the slowest documented VT.	At the discretion of the treating physician, anticoagulation recommended with aspirin or warfarin for 6–12 weeks	
Marchlinski 2016 [72]	Not required	Not dictated by the study protocol	Not dictated by the study protocol	At 6 months and at 1, 2, and 3 years	Not dictated by the study protocol	Per clinical conditions and physician preference	Cardiac perforation (*n* = 1), pericardial effusion (*n* = 3)
VANISH 2016 [307]	No	Continued preprocedure antiarrhythmic medications	Not specified	A 3-month office visit, echo, ICD check; a 6-month office visit, ICD check; every 6 months thereafter, an office visit, ICD check	VT detection at 150 bpm or with a 10–20 bpm margin if the patient was known to have a slower VT. ATP was recommended in all zones. The protocol was modified to recommend prolonged arrhythmia detection duration for all patients.	Intravenous heparin (without bolus) 6 h after sheath removal, then warfarin if substrate-mapping approach used or if more than 10 min of RF time	Major bleeding in 3 patients; vascular injury in 3 patients; cardiac perforation in 2 patients
SMS 2017 [319]	No	At the discretion of the investigator	At the discretion of the investigator	At 3, 6, 9, and 12 months, and at 3- or 6-month intervals until completion of the study or until 33-month follow-up was reached	VF zone at 200–220 bpm, detection 18 of 24 beats, shock only; VT zone detection at least 16 consecutive beats, ATP, and shocks. Where VT rates were exclusively >220 bpm, VT zone at 160–180 bpm was recommended; where VT rates were < 220 bpm, VT zone with a CL 60 ms above the slowest VT was recommended	Aspirin (250 mg/day) or warfarin as necessitated by the underlying heart disease	Two tamponades requiring pericardiocentesis

*AAD* antiarrhythmic dug, *ATP* antitachycardia pacing, *CL* cycle length, *ICD* implantable cardioverter defibrillator, *NIPS* noninvasive programmed stimulation, *RF* radiofrequency, *TIA* transient ischemic attack, *VF* ventricular fibrillation, *VT* ventricular tachycardia

#### Postprocedural care: Disposition

After completion of VT ablation, patients are generally monitored on telemetry in the hospital for at least one day, and often longer for VT ablation in cases of SHD or heart failure. Patients who are hemodynamically unstable, who require hemodynamic or ventilator support, or who have had pericardial bleeding, cardiac tamponade, or a pericardial drain left in place are usually monitored and stabilized in an intensive care unit prior to transfer to a regular telemetry floor. Selected right-sided ablation patients may be discharged the same day, if stable, but patients with significant comorbidities, any instability, or left-sided VA ablation are typically monitored >24 h. Transtelephonic or CIED remote monitoring is often used to facilitate follow-up (see Section 10.4). Outpatient follow-up generally occurs by 1–4 months after the procedure.

A survey of the writing committee showed that 42% routinely have patients monitored in an intensive care unit after ventricular ablation. Stable patients are kept in the hospital for 1 day (overnight) by 35%, 2 days by 52%, 3 days by 9%, and > 3 days by 4% of the writing committee members.

### Incidence and Management of Complications

#### Introduction

Ablation of VAs is an invasive procedure that can be performed in patients with or without SHD. Despite new technologies and progress in techniques in recent years, complications are expected, especially in patients with more severe disease. In fact, the incidence of complications related to VT ablation is higher in patients with SHD than in idiopathic VT [237, 326, 1109].

For the purpose of this document, major complications are defined as those that result in prolongation of hospital stay or another hospitalization, those that require additional intervention for treatment, and/or those that result in significant injury or death. All other complications, such as small hematomas not requiring intervention, are defined as minor. A recent meta-analysis reported major complication rates of 8%–10% after VT ablations [1110]. Slightly higher complication rates have been reported in administrative and registry real-world studies compared with clinical trials (9.39% vs 7.97%) [1110]. The most common complication reported is vascular damage, followed by pericardial complications (cardiac tamponade, hemopericardium, pericarditis). Although new technologies and techniques have been incorporated for VT ablations, the rate of complications has not decreased, perhaps because more patients at high risk have been scheduled for ablation [1109]. It is also notable that in one recent study, the complication rates were independently related to operators and to timing of the procedure, with significantly higher complication rates when the ablation was begun after 2 pm (10% vs 5%; *P* < .0001) [616].

#### Mortality

Interpretation of mortality in the setting of VT ablations in patients with SHD is difficult, given mortality can be a consequence of procedure-related complications but can also be related to procedural failure (incessant or recurrent VT). Table 10 shows the incidence of in-hospital or early mortality in patients who underwent VT ablation. Some clinical trials have shown no mortality, whereas other studies have reported rates of in-hospital or early mortality as high as 3% [71, 316, 317, 326, 451, 614, 1109]. Some of the following predictors of in-hospital or early mortality have been reported: smoking, hypothyroidism, fluid and electrolyte disturbances, chronic renal failure, peripheral vascular disease, lower LVEF, history of AF, ICM, and multiple slow VTs [71, 326, 614]. It is also important to note that VT ablation in low- or medium-volume centers has been shown to be an independent predictor of mortality in these complex ablations [326].

**Table 10:** Major complications of ventricular arrhythmia ablation in patients with structural heart disease

Complication	Incidence	Mechanisms	Presentation	Prevention	Treatment	Ref.
In-hospital mortality	0%–3%	VT recurrence, heart failure, complications of catheter ablation	Not applicable	Correct electrolyte disturbances and optimize medical status before ablation	–	[71, 316, 317, 326, 614]
Long-term mortality	3%–35% (12–39 months of follow-up)	VT recurrence and progression of heart failure	Cardiac nonarrhythmic death (heart failure) and VT recurrence	Identification of patients with indication for heart transplantation	–	[71, 316, 317, 614]
Neurological complication (stroke, TIA, cerebral hemorrhage)	0%–2.7%	Emboli from left ventricle, aortic valve, or aorta; cerebral bleeding	Focal or global neurological deficits	Careful anticoagulation control; ICE can help detection of thrombus formation, and of aortic valve calcification; TEE to assess aortic arch	Thrombolytic therapy	[71, 316, 317, 326, 614]
Pericardial complications: cardiac tamponade, hemopericardium, pericarditis	0%–2.7%	Catheter manipulation, RF delivery, epicardial perforation	Abrupt or gradual fall in blood pressure; arterial line is recommended in ablation of complex VT	Contact force can be useful, careful in RF delivery in perivenous foci and RVOT	Pericardiocentesis; if necessary, surgical drainage, reversal heparin; steroids and colchicine in pericarditis	[71, 316, 317, 326, 614]
AV block	0%–1.4%	Energy delivery near the conduction system	Fall in blood pressure and ECG changes	Careful monitoring when ablation is performed near the conduction system; consider cryoablation	Pacemaker; upgrade to a biventricular pacing device might be necessary	[71, 316, 326, 614]
Coronary artery damage/MI	0.4%–1.9%	Ablation near coronary artery, unintended coronary damage during catheter manipulation in the aortic root or crossing the aortic valve	Acute coronary syndrome; confirmation with coronary catheterization	Limit power near coronary arteries and avoid energy delivery <5 mm from coronary vessel; ICE is useful to visualize the coronary ostium	Percutaneous coronary intervention	[71, 316, 317, 326, 614]
Heart failure/pulmonary edema	0%–3%	External irrigation, sympathetic response due to ablation, and VT induction	Heart failure symptoms	Urinary catheter and careful attention to fluid balance and diuresis, optimize clinical status before ablation, reduce irrigation volume if possible (decrease flow rates or use closed irrigation catheters)	New/increased diuretics	[71, 316, 317, 614]
Valvular injury	0%–0.7%	Catheter manipulation, especially retrograde crossing the aortic valve and entrapment in the mitral valve; energy delivery to subvalvular structures, including papillary muscle	Acute cardiovascular collapse, new murmurs, progressive heart failure symptoms	Careful catheter manipulation; ICE can be useful for identification of precise location of energy delivery	Echocardiography is essential in the diagnosis; medical therapy, including vasodilators and dobutamine before surgery; IABP is useful in acute mitral regurgitation and is contraindicated in aortic regurgitation	[71, 316, 317, 614]
Acute periprocedural hemodynamic decompensation, cardiogenic shock	0%–11%	Fluid overloading, general anesthesia, sustained VT	Sustained hypotension despite optimized therapy	Close monitoring of fluid infusion and hemodynamic status -Optimize medical status before ablation -pLVAD -Substrate mapping preferred, avoid VT induction in higher-risk patients	Mechanical HS	[71, 316, 317, 451, 614]
Vascular injury: hematomas, pseudoaneurysm, AV fistulae	0%–6.9%	Access to femoral arterial and catheter manipulation	Groin hematomas, groin pain, fall in hemoglobin	Ultrasound-guided access	Ultrasound-guided compression, thrombin injection, and surgical closure	[71, 316, 317, 326, 614]
Overall major complications with SHD	3.8%–11.24%					[71, 316, 317, 326, 614]
Overall all complications	7%–14.7%					[452, 614, 1109]

*AV* atrioventricular, *ECG* electrocardiogram, *HS* hemodynamic support, *IABP* intra-aortic balloon pump, *ICE* intracardiac echocardiography, *MI* myocardial infarction, *pLVAD* percutaneous left ventricular assist device, *RF* radiofrequency, *RVOT* right ventricular outflow tract, *SHD* structural heart disease, *TEE* transesophageal echocardiography, *TIA* transient ischemic attack, *VT* ventricular tachycardia

#### Acute periprocedural hemodynamic decompensation and cardiogenic shock

Periprocedural AHD, defined as sustained systolic hypotension despite optimized doses of vasopressors or requiring mechanical HS and procedure discontinuation, can occur in up to 11% of VT ablations in patients with SHD [451]. The occurrence of AHD is associated with increased in-hospital and long-term mortality, higher recurrence of VT, and procedure failure [451, 613, 1111, 1112]. Some of the following predictors of AHD have been reported, although they were tested only in a univariate analysis: older age, diabetes mellitus, ICM, NYHA class III/IV, VT storm, lower EF, prolonged procedure duration, and general anesthesia. Ablation should be carefully planned in these patients [451]. A score was developed to identify higher-risk patients, who might benefit from periprocedural HS. The PAAINESD score is further discussed in Section 5.1.

Some measures can help to prevent these complications: close monitoring of fluid balance and diuresis, careful attention to hemodynamic status, optimization of medical status before ablation, avoidance of VT induction in higher-risk patients, preferring substrate mapping ablation, and avoidance of general anesthesia and certain drugs that can result in myocardial depression, including propofol. Use of a pLVAD, as discussed in Section 6.4, might be helpful in specific cases [452, 613, 1111, 1112].

The pLVAD has been used in two scenarios: prophylactic, when higher-risk patients are identified, and for rescue during an acute complication. When used prophylactically, the pLVAD has been shown to prevent AHD and has a lower 30-day mortality (4.2% vs 58%). Mathuria et al. [455] showed a similar 30-day mortality among patients with prophylactic use compared to the non-pLVAD group (4.2% vs 3.1%), although the first group showed a higher PAAINESD score (16.5 vs 13.5; *P* = .02). Other studies have failed to demonstrate the benefit of pLVAD, although the fact that these devices are selected for patients with more severe disease is a bias that is not possible to rule out [452, 455, 611, 613, 1112]. Furthermore, few randomized, prospective, controlled trials exist to identify the appropriate utility of this device in the setting of VA ablation.

#### Neurological complications

Neurological complication is a rare but devastating event due to cerebral emboli or intracerebral hemorrhage that can occur with VA ablation, with a reported incidence of 0% to 2.7% [71, 316, 317, 326, 614]. It can occur during or shortly after the ablation procedure, usually in the first 24 h, although in the 2 subsequent weeks following the procedure the risk is still present. Possible mechanisms for thromboembolic complications include thrombus formation on the catheter or on the tissue, air embolism, plaque disruption in the aortic arch, displacement of endocardial thrombus adhered to LV prior to ablation, char formation and/or tissue disruption during ablation, or generation of calcific emboli while passing a catheter through a heavily calcified aortic valve. Optimal anticoagulation during the procedure targeting ACT >300 s and adequate control of RF energy delivery parameters are important practices that could help to prevent thrombus formation during ablation. Given that air embolism can also occur, careful attention to sheath management is crucial. In the presence of specific neurological symptoms suggesting stroke or transient ischemic attack after ablation, brain MRI, CT, or cerebral angiography should be performed. If stroke is confirmed, thrombolytic therapy or endovascular mechanical or pharmacological therapy might be useful.

A possible neurological complication is asymptomatic microembolism that results from thrombus formation, gas, tissue and/or fat dislodgement, and/or air embolism [1113]. The long-term consequences of this phenomenon are unknown.

#### Pericardial complications: Cardiac tamponade, hemopericardium, and pericarditis

Pericardial complication is the second most common reported complication of VT ablation in patients with SHD, being reported in 0% to 4.5% of procedures, although it can be higher in the subgroup of procedures in which pLVAD is used [71, 316, 317, 326, 455, 611]. In one study by Turagam et al. [613], however, the incidence of cardiac tamponade was 5% vs 1.8% when HS was used in VT ablation procedures. A greater incidence of pericardial effusion is expected in epicardial compared with endocardial approaches [1114]. Possible mechanisms of this complication are cardiac perforation due to overheating, direct trauma due to catheter manipulation, transseptal accident when this approach is chosen, or accident when using a pericardial approach. The level of anticoagulation can also influence the possibility of hemopericardium, facilitating the perpetuation of bleeding. Cardiac tamponade must be suspected if there is a drop in blood pressure; thus, an arterial line for continuous blood pressure monitoring is important, and previous recordings of cardiac silhouette in the left anterior oblique view can be helpful. ICE can help to prevent or quickly detect this complication, allowing earlier initiation of appropriate treatment that might prevent more serious and irreversible consequences. The use of ICE in this setting is discussed in Section 8.6. When pericardial drainage is needed, anticoagulation therapy should be reevaluated, and reversing heparin effects with protamine might be necessary. Percutaneous pericardiocentesis and placement of a pericardial drain are frequently mandatory in cardiac tamponade. The aspirated pericardial blood can be returned to the central vein when the cardiac tamponade is diagnosed during the procedure, although this approach has not been systematically evaluated. The ablation procedure is usually interrupted unless bleeding stops shortly after drainage. In some cases, surgical repair might be necessary if the bleeding is not controlled. After drainage and clinical stability, a pericardial drain should be maintained during the first 24 h, and echocardiography should be used for further evaluation and decisions regarding the timing of removing the pericardial drain. It is also important to carefully evaluate the appropriate timing of reintroducing anticoagulation therapy as needed.

Another pericardial complication that can be found after VT ablation is pericarditis. It can be the sole complication or it might follow pericardial drainage due to cardiac tamponade. Symptoms of chest pain and low-grade fever, leukocytosis, and elevated C-reactive protein levels are commonly observed; diagnosis can be confirmed with ECG or echocardiogram. Pericarditis is also typically more frequent when an epicardial approach is used. Steroid administration in the pericardial space might be useful to prevent pericarditis (see Section 10.1). Treatment with nonsteroidal anti-inflammatory drugs, colchicine, and/or steroids is useful.

#### Vascular injury

The incidence of vascular injury after VT ablation ranges from 0% to 8.6% and is the most common major complication reported after VT ablation procedures [1110]. Vascular complications after VT ablation are typically related to vascular access and include hematoma, retroperitoneal hematoma, pseudoaneurysm, arteriovenous fistula, and dissection or occlusion of the artery accessed. Prior to sheath deployment, confirmation that the guide wire used for arterial access is indeed in the lumen and not in the arterial wall is important to avoid retrograde dissection of the iliac artery and aorta. This can be accomplished with techniques such as ultrasound or angiography. Furthermore, longer sheaths are preferable in the setting of tortuous peripheral vessels to avoid damage of the vessel wall with the catheter tip. Imaging of the aortic arch (with transesophageal echocardiography or CT) prior to ablation using a retrograde approach could help to identify patients at risk for thromboembolic events in the presence of mobile atheromas or thick plaque material. ICE can also be beneficial to identify plaque material in the ascending aorta prior to a planned retrograde approach. Many electrophysiologists curve the ablation catheter to a “J” shape in the descending aorta to minimize trauma to the aortic valve when mapping the LV with a retrograde approach. The catheter is then prolapsed into the LV.

In higher-risk patients, such as those with high body mass index, body surface area < 1.6 m^2^, hypertension, age > 70 years, baseline anemia, and known peripheral vascular disease, ultrasound-guided percutaneous access and careful management of anticoagulation status should be strongly considered [553]. Reversal of heparin with protamine before sheath removal can also be useful to prevent groin hematoma. There are no conclusive data demonstrating that the use of vascular closure devices decreases this complication compared with manual compression [1115].

Large hematomas after sheath removal are the most common complication and are usually self-limited, but they can be large enough to result in blood transfusion. When femoral arterial pseudoaneurysm and arteriovenous fistulas are suspected, ultrasound Doppler or CT scan are useful imaging tools for diagnosis. A CT scan can also be useful in patients with back pain and acute anemia after ablation to evaluate for retroperitoneal hematoma. Percutaneous thrombin injection, surgical repair of pseudoaneurysm and arteriovenous fistula, and surgical evacuation of large hematomas are possible interventions for these vascular complications.

#### Myocardial ischemia, coronary artery damage

Myocardial ischemia can occur during VT ablation due to hypotension after VT induction or other causes. MI was reported in 1.7% in a large retrospective database study [326]. Caution is crucial especially in patients with known nontreatable ischemia, possibly with avoidance of VT induction. Coronary artery damage can also cause myocardial ischemia. Although injury to coronary arteries is rare, it can occur while crossing the aortic root with the catheter when using the retrograde aortic approach, with ablation in the coronary cusps, or with dislodgement of the ablation catheter in the LVOT. ICE, as discussed in Section 8.6, can be useful for continuous visualization of the ablation catheter and the coronary artery ostium. Coronary angiography and continuous protection using a coronary angioplasty wire can be useful when the ablation site is close to a coronary ostium [1116].

#### Valve injury

Valve injury is a rare but potentially fatal complication of VT ablation (Table 10). The incidence of valve injury is reported in up to 0.7% of cases [71, 316, 317, 614]; when using the retrograde aortic approach, it can occur while crossing the aortic valve with the ablation catheter. Entrapment of the ablation catheter in the mitral or tricuspid valve can also occur using transseptal or retrograde approaches. Energy delivery direct to the valve apparatus can lead to valve injury. Ablation of the papillary muscle could result in this complication, and it can occur with both RF energy and cryoablation [835, 1117].

Clinical presentations of this complication include acute cardiovascular collapse, new murmurs, and symptoms of worsening congestive heart failure. It is important to note that this complication can occur immediately after the ablation when resulting from entrapment or mechanical damage, but when resulting from direct energy delivery to the papillary muscle, symptoms can appear weeks after the ablation procedure.

To prevent valve damage, one should be careful whenever manipulating the catheter when crossing the aortic valve and mapping near the mitral valve. ICE is useful to continuously visualize catheter position and its relationship with the valve apparatus and papillary muscle (see details of ICE in Section 8.6). Surgical intervention might be needed for removal of an entangled catheter or for repair of a damaged valve. Given the acute damage of the valve, repair instead of valve replacement is always preferable whenever possible.

#### Atrioventricular Block

AV block is reported in up to 1.4% of the VT ablations [71, 316, 317, 326, 614], and it can occur when ablation is performed in the septal region near the conduction system. It can be anticipated in patients with a known diseased conduction system, especially in patients with previous complete bundle branch block (either right or left) and energy delivered to the basal septum (either left or right sided). If the ablation is performed during VT, monitoring of AV conduction is not possible, and ablation during sinus rhythm might be preferable. Although many VT ablations are performed in patients with a CIED, the need for RV pacing could worsen LVEF, and upgrade to CRT might be required.

### Hemodynamic deterioration and proarrhythmia

**Table:**
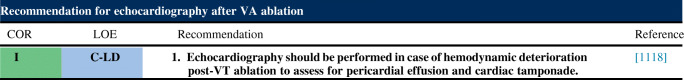

#### Recommendation-specific supportive text

1. Cardiac tamponade is a serious and potentially reversible cause of hemodynamic deterioration after VA ablation. It can present intraprocedure, postprocedure, or late. When suspected, immediate transthoracic, transesophageal, or ICE should be performed to assess for the presence of a pericardial effusion or thrombus [1118].

#### Synopsis

Extensive ablation in viable myocardium and repeated VT episodes with hypotension that can cause myocardial ischemia can lead to worsening of heart failure or cardiogenic shock in patients with impaired ventricular function. In patients with scar-related VT, particularly after MI, heart failure accounts for more than one-third of mortality during follow-up late after ablation and exceeds 10% per year in some studies [35, 70, 71, 330, 452, 1119–1122]. In the Multicenter Thermocool Ventricular Tachycardia Ablation Trial, 35% of 1-year mortality was due to heart failure [71]. In another study, despite the lack of a difference in the EF for the entire group, 14 of 62 (22.5%) patients did have a decline in EF when a repeat echocardiogram was performed within 7 days of the ablation [1122]. However, some observations suggest that VT ablation might not be the sole nor major cause of the heart failure, given several studies that assessed pre- and postablation LV function did not report adverse effects on LVEF before and after ablation [36, 1123]. Clinical pre- and intraprocedural variables indicating poorer clinical status, such as low LVEF, chronic kidney disease, VT storm, and unmappable VTs can predict mortality after VT ablation. The International VT Ablation Center Collaborative Group Study reported that patients with more severe illness who have multiple comorbidities who received HS during ablation had higher mortality [613]. Therefore, the reported heart failure mortality could be in line with expectations for these patients. Of 100 early mortality cases, 48 (48%) patients had early recurrent VT preceding death, although the time course from time of first VT recurrence to death was highly variable. Refractory VT was the cause of death in 22% of cases, with another 39% dying of other cardiac causes (most commonly advanced heart failure) [452].

Nevertheless, hemodynamic deterioration and early mortality from heart failure after ablation remain concerns, especially in patients with fragile hemodynamic status, patients with evident hemodynamic deterioration, or patients who required extensive ablation in areas of normal ventricular myocardium or near coronary arteries. Close monitoring of LV function and potential need for continued hemodynamic monitoring and/or support might be necessary after such ventricular ablation procedures.

Nontransmural and incomplete lesions can form a new or modified substrate, promoting arrhythmia after ablation; indeed, the majority of VTs recurring after ablation of postinfarction VT are new VTs, as determined by analysis of ICD electrograms and repeat ablation procedures [896].

### Follow-up of patients post catheter ablation of ventricular tachycardia

**Table:**
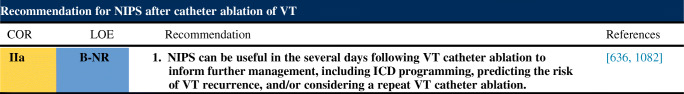

#### Recommendation-specific supportive text

1. Some advocate for routine PES, largely NIPS, shortly after VT catheter ablation and before discharging patients. However, there are no data from RCTs on the clinical usefulness of such a practice. Data on NIPS after VT catheter ablation were generated by two observational studies. In one such study, 132 patients with SHD underwent NIPS an average of 3 days after VT catheter ablation; 59 (44.7%) patients had no inducible VT, 49 (37.1%) had inducible nonclinical VT, and 24 (18.2%) had inducible clinical VT. At 1 year, patients with inducible clinical VT had a significantly lower VT-free survival than patients with no inducible VT (<30% vs >80%; *P* < .001). The authors concluded that when patients with VT and SHD have no VT or have only inducible nonclinical VT at the end of ablation or if they are too unstable to undergo final programmed stimulation during ablation, NIPS can be useful in the subsequent several days to further define the risk of VT recurrence, and if clinical VT is inducible during NIPS, to consider repeating VT catheter ablation due to the high risk of recurrence during follow-up (1541]. In another study, 218 PESs (186 noninvasive and 32 invasive) were performed an average of 6 days after ablation on beta-blocker therapy in 210 consecutive patients. The positive and negative predictive values of PES for VT recurrence over 1 year of follow-up were 53% and 88%, respectively, and the negative predictive value was highest among patients with IHD and those with an LVEF >35% [1082]. However, these studies were single-center, retrospective studies that did not include a large number of patients and that did not subject all patients undergoing VT catheter ablation to PES [636, 1082]. As such, the routine performance of NIPS is currently not standard of care, and decisions regarding NIPS should be individualized until more data from prospective studies, preferably RCTs, emerge on the clinical usefulness of NIPS post-VT catheter ablation.

#### Summary

Close follow-up of patients after VT catheter ablation is critically important because complications can be detected and addressed during this period, and decisions regarding postprocedure management are made. These decisions relate to whether to perform NIPS, whether to maintain the patient on an AAD, and how to best program and follow ICDs in patients with such devices.

Another important decision after VT catheter ablation is whether to maintain a patient on an AAD, and if so, what medication to choose and for what duration. No RCTs have specifically addressed these issues, and RCTs of VT catheter ablation implemented different protocols related to postprocedure use of AADs (Table 9). In 3 clinical trials of VT catheter ablation, preablation AADs (mostly amiodarone) were continued either for 3 to 6 months or for an unspecified period of time [70–72, 307]. In 3 other clinical trials of VT ablation, the use of AADs post-VT catheter ablation was either discouraged or left to the discretion of the treating physician [309, 317, 318]. In the SMASH-VT trial, no patient received an AAD (other than beta blockers) before the primary endpoint was reached [1123]. Given this variability in the use of AADs post-VT ablation in prior RCTs, there is no standard of care for their use in this scenario; as such, decisions in this regard should be individualized based on patient characteristics and findings of the PES at the end of the VT catheter ablation (or later), keeping in mind the toxicities of these medications and their potential detrimental effects on survival [4]. In particular, given its toxicities, many favor discontinuing amiodarone after ablation. In fact, amiodarone use had decreased in several prospective cohort studies of VT ablation [71, 72, 309], and dose reduction or discontinuation had been feasible in patients who were not inducible post ablation in 1 single-center experience [1124].

Data on the best programming parameters of ICDs post-VT catheter ablation are scarce, given that studies of optimal ICD programming were conducted largely in patients with no prior VT [1125–1127]. In a few clinical trials of VT catheter ablation, information on ICD programming was not provided [70, 71, 1123]. In the clinical trials that provided information on ICD programming, programming instructions to the sites were variable (Table 9). However, based on current practice, ICDs may be programmed with a 10–20 bpm margin if the patient has slower VT and, where appropriate, with ATP and prolonged arrhythmia detection durations and higher detection rates for VF. Although not specific to post-VT catheter ablation programming, the HRS document on optimal programming of ICDs can be a useful resource [1127]. Given the risk of VT recurrence after VT ablation, it is imperative to follow ICDs closely, preferably with remote monitoring that allows earlier detection and treatment of VT recurrences [1128].

### Assessing the outcomes of catheter ablation

#### Introduction

**Fig. 13:**
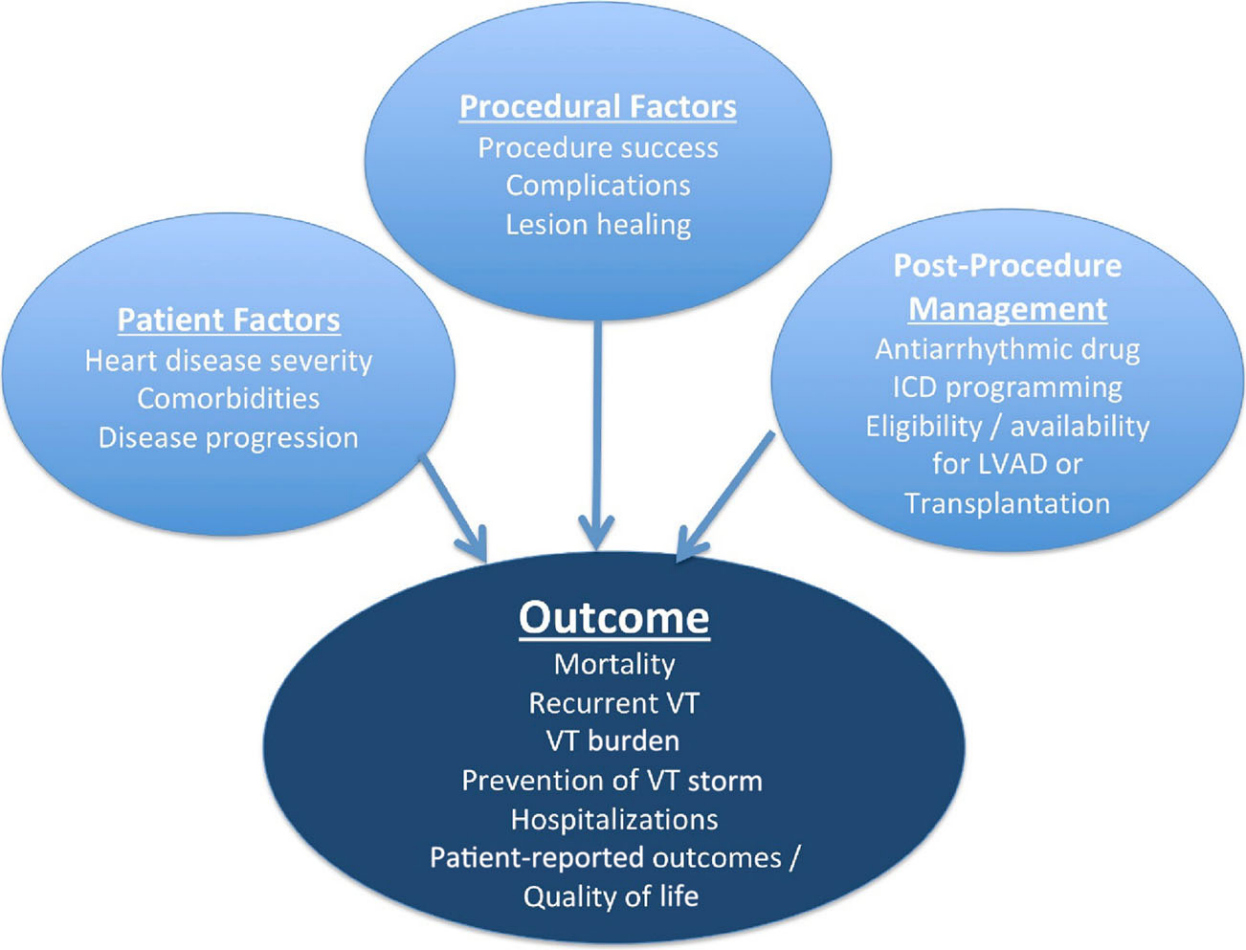
Factors influencing outcomes post VA ablation. ICD = implantable cardioverter defibrillator; LVAD = left ventricular assist device; VA = ventricular arrhythmia; VT = ventricular tachycardia

Important outcomes of clinical trials for VA catheter ablation include procedure-related complications and mortality, recurrent VA, long-term mortality, and quality of life. It is also important to recognize that outcomes are also impacted by the many factors following ablation that have the potential to influence the frequency and nature of arrhythmia recurrences (Fig. 13).

#### Recurrent arrhythmias

Recurrence of any sustained VT is an important endpoint for patients who have ICDs. In patients with SHD and ICDs, the QRS morphology of recurrent VT compared with preablation VT is frequently difficult to assess and is not usually reported in clinical trials. The ECG morphology of spontaneous VTs terminated by ICDs is often unknown, and the QRS morphology of VTs can be altered by changes in the arrhythmia substrate and AAD therapy after ablation. Any spontaneous sustained VT might be of clinical significance, whether it was observed previously or not. Clinical trials assessing the effect of catheter ablation on recurrent sustained VT have reported the time from the ablation procedure to the first recurrence of VT in Kaplan–Meier curves [307, 316–318]. These curves provide important information and are widely understood. They fail, however, to capture other potential benefits of the procedure, including reduction in VT burden, prevention of VT storm, conversion of symptomatic episodes causing ICD shocks to asymptomatic episodes terminated by ATP, and reductions in medications.

For patients with nonsustained VAs and PVCs, outcome assessment is determined by ambulatory monitoring of a duration sufficient to detect recurrences, based on the frequency of the arrhythmia prior to treatment, and considering the goals of therapy.

#### Arrhythmia burden

Several studies have reported arrhythmia burden using the patient as their own reference during a specified time prior to ablation and comparing the arrhythmia frequency after ablation in patients who have ICDs [71, 72, 1129]. This type of analysis can be challenging. The number of VT episodes before and after ablation can be influenced by programmed ICD detection criteria and AAD management. It is also difficult to know how to assign a “frequency” to preablation incessant VT or VT storm for comparison with single VT recurrences post ablation. Prior ICD interrogations are not always available to confirm the arrhythmia burden. The VT burden that has clinical relevance has not been defined and likely would vary depending on other patient factors. Further work on methods by which arrhythmia burden can best be assessed and compared in patients undergoing VT ablation is needed.

#### Ventricular tachycardia storm

VT storm has been defined as 3 or more separate episodes of sustained VT in a 24-h period, and it has been associated with poorer outcomes [1130]. Prevention of VT storm is an important goal. Again, ICD programming has an important influence on detection and counting of these events.

#### Hospitalizations

Hospitalizations for cardiovascular disease and recurrent VA are also potentially important outcomes. Recurrent arrhythmias can be precipitated by intercurrent illness. Whether a patient requires hospitalization for a recurrence is sometimes a subjective assessment.

#### Patient-reported outcomes

Assessing the impact of therapies on quality of life and measuring patient-reported outcomes is endorsed by the AHA and the European Society of Cardiology [1131, 1132]. In the United States, the Food and Drug Administration has also recently implemented the Medical Device Development Tools program in which patient-reported outcome tools can be submitted for evaluation and potential certification allowing their use for clinical outcome assessment in trials of medical devices [1133].

Assessment of the impact of ablation on quality of life is likely to reflect not only the outcome of ablation therapy, but also the severity of the arrhythmia’s impact on the patient prior to ablation. For example, a recent trial of left ventricular assist devices (LVADs) found that LVADs improved health status in patients with heart failure who had low self-reported quality of life, but not in those with acceptable quality of life at the time of LVAD implantation [1134]. The measure of quality of life prior to ablation is potentially useful information for defining who benefits from ablation.

Although a number of validated tools are available, the interpretation of findings can also be challenging. The VANISH trial assessed quality of life using 4 validated instruments (Short Form 36 Health Survey, the Implantable Cardioverter Defibrillator Concerns Questionnaire, the Hospital Anxiety and Depression Scale, and the EuroQol five dimensions questionnaire) [1135]. Among 16 different measures of quality of life at 6 months and 1 year that were compared with baseline, persistent improvements were observed in some (energy and fatigue), transient improvements that were no longer present at 1 year in others (eg, social functioning), and no improvement was found in yet others (eg, general health perceptions scale). How to integrate multiple measures to assess net benefit of the procedure could be a challenge.

#### Mortality

Assessing mortality after ablation is important. Although mortality is relatively high in many VA ablation populations, and VA recurrence has been associated with increased mortality, no trial has yet shown a mortality benefit from catheter ablation. Efforts to enroll a sufficient number of patients will be key to conclusively establishing the impact of ablation on mortality in a randomized trial [318, 1136]. Recently, risk scores for predicting mortality in patients with SHD undergoing VT ablation have been developed, and await further validation [451, 1137].

## Training and institutional requirements and competencies

### Training requirements and competencies for catheter ablation of ventricular arrhythmias

**Table:**
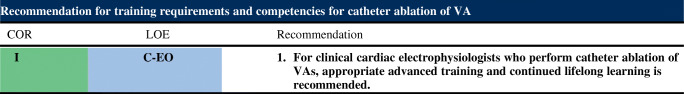

#### Recommendation-specific supportive text

1. Catheter ablation of VAs requires advanced skills in clinical cardiac electrophysiology. Physicians performing these procedures should be trained at electrophysiology programs with expertise in complex VA ablations and should meet the general and advanced training requirements for clinical cardiac electrophysiologists during training or with an experienced mentor if novel procedures and approaches have developed after training.

#### Training requirements

For adult electrophysiologists in the United States, these training competencies are outlined in the *2015 ACC COCATS 4: Task Force 11: Training in Arrhythmia Diagnosis and Management, Cardiac Pacing, and Electrophysiology report*, the *2015 ACC/AHA/HRS Advanced Training Statement on Clinical Cardiology Electrophysiology*, and the *2009 EHRA/HRS Expert Consensus on Catheter Ablation of Ventricular Arrhythmias* [1, 1138, 1139]. For pediatric electrophysiologists in the United States, these training competencies are outlined in the *2015 SPCTD/ACC/AAP/AHA Task Force 4: Pediatric Cardiology Fellowship Training in Electrophysiology* and the *2013 Recommendations for Advanced Fellowship Training in Clinical Pediatric and Congenital Electrophysiology: A Report from the Training and Credentialing Committee of the Pediatric and Congenital Electrophysiology Society* [1140, 1141]. Although these requirements mainly reflect fellowship training in the United States, other countries have requirements that are similar, including the Working Group of Pacing, Electrophysiology of the French Society of Cardiology [1142]. Training requirements vary from country to country, but irrespective of local differences, appropriate advanced training and continued lifelong learning are strongly recommended for all clinical cardiac electrophysiologists who perform catheter ablation for VAs.

As the field of catheter ablation for VAs continues to evolve with introduction of new approaches, technology, and application in higher-risk and more complex patients (ie, adults with VADs and CHD), trainees and practicing operators specializing in these ablations are expected to continue to maintain their core and specialized competencies as outlined in the *2017 ACC/HRS Lifelong Learning Statement of Clinical Cardiac Electrophysiology Specialists* [1143]. These procedures should be performed by select qualified cardiac electrophysiologists with a practice focus on VAs at programs with expertise.

Physicians performing catheter ablation for VAs should achieve proficiency in 6 core competency domains promulgated by the Accreditation Council of Graduate Medical Education and American Board of Medical Specialties. These 6 core competency domains include Medical Knowledge, Patient Care and Procedural Skills, Systems-Based Practices, Practice-Based Learning and Improvement, Professionalism, and Interpersonal and Communications Skills [1144].

#### Medical knowledge

A detailed understanding of cardiac anatomy is required, including the conduction system, the coronary arterial and venous systems, and anatomical variations due to underlying heart disease. Knowledge is necessary of the mechanisms and pathophysiology of VAs, with a particular focus on the relationship between these arrhythmias and acquired, inherited, or SHD; sympathetic and parasympathetic tone; and drugs. This understanding, along with proficiency in electrocardiographic interpretation, helps guide appropriate mapping and ablation approaches. In addition, knowledge of patient-specific factors such as disease type, severity, and comorbidities; procedural risks; and alternative therapies such as AADs, surgery, or device therapy should be used to guide appropriate patient selection.

#### Patient care and procedural skills

A comprehensive and structured approach to the clinical evaluation and management of patients with VAs undergoing catheter ablation is necessary across all settings (inpatient and outpatient) and time points (preprocedural planning, periprocedural and intraprocedural care, and postprocedural follow-up). Trainees should demonstrate and maintain these proficiencies as pertains to their practice area of focus. A clear understanding of the various approaches and systems used (mapping, ablation, and imaging) and proficient demonstration of the skills to perform VA ablations are required. Additional advanced technical skills are needed for those seeking to perform epicardial ablations or ablations in CHD. Given patients undergoing complex VA ablations often have underlying medical conditions and comorbidities, recognition of the potential need for a multidisciplinary approach and coordination with other specialists (anesthesiologists, surgeons, interventional cardiologists, cardiologists, heart failure specialists, and/or intensivists) will ensure optimal procedural outcomes.

#### Systems-based practice

Complications can occur in catheter ablation procedures for VAs that can range from minimal to fatal. These can include vascular injury, thromboembolic events, cardiac tamponade, malignant VA including electrical storm, acute MI, and hemodynamic instability or collapse. A careful understanding of the potential procedural complications that might arise from either the patient’s underlying medical conditions or the electrophysiology procedure itself is necessary. When appropriate, the use of a multidisciplinary team for the management of high-risk patients should be in place for prevention, prompt detection, timely intervention, and optimal management of complications. Functional systems should be in place to promptly care for patients in case of unanticipated complications.

#### Practice-based learning and improvement

All trainees should continue to participate in lifelong learning to maintain and enhance skills and knowledge through self-assessment, regular literature review, updated practice guidelines, consensus document and appropriate use criteria, and attending appropriate scholarly meetings with a focus on VAs.

#### Professionalism

On the basis of personal expertise and technical skills, practicing clinical cardiac electrophysiologists and trainees should practice within their scope as pertains to the specialized field of catheter ablation for VAs.

#### Interpersonal and communications skills

Catheter ablation for VAs can be challenging due to increased procedural risk and complexity necessitating additional technical expertise. Practicing clinical cardiac electrophysiologists and trainees should engage patients, families, and interprofessional teams in a shared decision-making approach. These physicians must develop the skills to communicate and counsel effectively regarding the diagnosis, risks, and benefits of catheter ablation, and alternative management options.

#### Ionizing radiation

Awareness is rising of the detrimental effects on patients, the electrophysiology staff, and the operator of ionizing radiation during imaging. The use of low-frame rate fluoroscopy equipment, pre- and intraprocedural imaging with ICE, as well as 3D EAM, all contribute to reduce radiation exposure. Appropriate education of all personnel involved in the procedure on the risks and benefits of radiation is key for safer imaging. Societal recommendations have emphasized the importance of awareness of the principles of radiation safety and their implementation on a day-by-day basis while using radiation for medical imaging [1145, 1146].

### Institutional requirements for catheter ablation of ventricular tachycardia

**Table:**
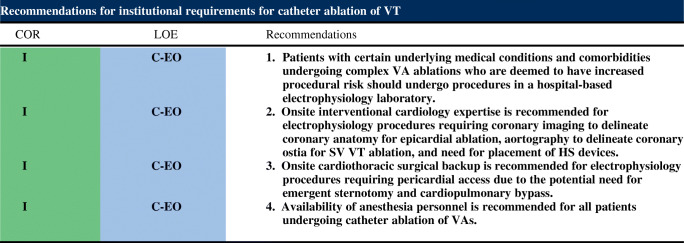

#### Recommendation-specific supportive text

1. High-risk patients include those with advanced heart failure, severe ventricular dysfunction, severe valvular dysfunction, prosthetic heart valves, CHD, inherited arrhythmia disorders, recent MI, recent stroke, chronic kidney disease, severe obstructive pulmonary disease, pulmonary hypertension, severe/morbid obesity, active oral anticoagulation, and advanced or pediatric age groups. Due to the potential risks associated with catheter ablation in close proximity to the coronary arteries and epicardial ablation, these procedures should be performed in a hospital-based electrophysiology lab with immediate availability of interventional cardiology or cardiothoracic surgical support [478].
2. Interventional procedures, including coronary angiography, aortography, and deployment of percutaneous HS devices (pLVAD, IABP), requires advanced skills and should be performed by appropriately trained personnel.
3. Epicardial ablation and mapping requires pericardial access that carries the risk of ventricular laceration or perforation. Inadvertent injury to the ventricle can result in life-threatening tamponade and hemodynamic collapse, which might require emergent surgical intervention via sternotomy. Immediate surgical intervention is critical and dependent on designated surgical backup (in-room or onsite with a clear notification protocol in place) and immediate access to an emergency surgical tray [478].
4. Due to procedural complexities and patient factors, including underlying medical conditions and comorbidities, the use of anesthesia services (anesthesiologist, nurse anesthetist, or equivalent trained personnel) provides optimal periprocedural patient care and additional expertise in the setting of life-threatening complications [478].

### Ventricular tachycardia network and ventricular tachycardia unit

VT ablation is a domain of specialized centers with high operational expertise. Within these centers, logistics for optimizing patient flow and referral are crucial. Therefore, many tertiary referral centers for VT ablation have established their network of regional referring centers to improve patient admission and early VT ablation. Few studies have documented their experience with this concept [312, 359].

Conceptionally, “VT networks” centralize around highly experienced centers capable of all techniques associated with VT and electrical storm ablation, including epicardial ablation and HS. Discussion with a multidisciplinary team and immediate transfer should be possible. Electrophysiology staff should be available on a 24/7 basis. Although acute ablation, defined as ablation for rhythm stabilization within 8 h after onset, is rare, there is a potential need for catheter ablation on a 24/7 basis [312, 359, 1147]. However, very few electrophysiology labs worldwide have this capacity.

Dedicated VT units have been established in some tertiary referral centers to expedite the transfer and management of patients with VT in a specialized intensive care unit. The optimal technical set-up of a dedicated VT unit has not been defined, but 12-lead ECG monitoring, online device telemetry, and intensive care unit facilities are helpful. The organization of VT units and networks would likely vary substantially across different countries according to the local health care delivery organization and resources. The ability to provide 24/7 catheter ablation might be useful for effective early rhythm stabilization, specifically in unstable patients with recurrent VA; however, comparisons with standards of care, including pharmacological suppression, HS, and neuromodulation, have not been made [312, 359]. Less than half of the writing committee members’ institutions have a formal capacity to perform VA ablations outside of regular working hours (necessitating personnel to be called into the hospital overnight or on weekends), although two-thirds had performed such a procedure after hours in exceptional circumstances, almost all between <1 and 5 times per year. Opinions were divided about whether a formal on-call schedule for VA ablation was necessary.

## Future directions

### Clinical trials of catheter ablation of ventricular tachycardia

#### Introduction

The development of high-quality randomized trial evidence to establish an optimal therapy for VT has been challenging, despite the clinical importance of the question. Relatively few RCTs have been published comparing catheter ablation with noninterventional therapies. Recent consensus and guideline documents have consistently stressed the importance of further research in the field [1, 4, 156], particularly the need for prospective randomized trials. Important clinical questions remain regarding optimal techniques for catheter ablation, its optimal role and timing in ischemic and nonischemic cardiomyopathies, as well as the relative merits of catheter ablation in comparison with antiarrhythmic or other pharmacological therapy. Published randomized trials completed thus far have included patients with ICM. Three trials compared catheter ablation to standard care in the setting of initial presentations with VAs [316], with presentations with tolerated VT [317], and with nontolerated VT [319]. One trial compared ablation to more aggressive AAD therapy for patients with VT despite first-line AAD treatment [307]. At least 8 trials of catheter ablation for VT have been initiated but were terminated because of insufficient enrollment (STAR-VT, NCT02130765; VeTAMed, NCT01798277; INTERVENE, NCT02301390; AVATAR, NCT02114528; ASPIRE, NCT01557842; CEASE-VT, NCT01097330; BERLIN-VT, NCT01045668; CALYPSO, NCT01576042), of which only CALYPSO has published results available. Trials comparing various methods of VT ablation or treatment have been reported [VISTA [619]; Ultra High Density mapping, NCT02083016 [677]] or are listed as ongoing on trial registry sites [MAGNETIC VT [740]; Impress, NCT03531502; ZFOVA zero fluoro trial, NCT03041519; Ripple Mapping, NCT02216760]. Barriers to completion of research in this area are significant, and include patient and provider biases and preferences (particularly for randomized trials of procedures compared to pharmacotherapy), challenges to standardization of procedural techniques and endpoints, as well as trial funding and reimbursement [1136].

#### Ongoing randomized controlled trials

Several randomized trials comparing catheter ablation with medical therapy are ongoing. The PARTITA trial (NCT01547208) is enrolling and following 586 patients with ICD implantation, and randomly allocating 176 who present with first ICD shocks to early catheter ablation vs withholding ablation until electrical storm occurs, with a primary composite endpoint of heart failure hospitalization and death from any cause. The PAUSE-SCD trial (NCT02848781) is enrolling 120 patients with ICM or NICM and randomly allocating them to ICD implant followed by catheter ablation or medical therapy with a composite endpoint of recurrent VT, cardiovascular re-hospitalization, and all-cause mortality. The IMPRESS TRIAL (NCT03531502) is enrolling 75 patients with cardiomyopathy who experienced a first ICD shock and is performing a NIPS procedure with a noninvasive mapping system (Cardioinsight, Medtronic). If the NIPS procedure is positive, then patients will be randomized to catheter ablation guided by noninvasive mapping or standard medical therapy with a primary endpoint of ICD shocks. The PREVENTIVE VT Trial (NCT03421834) is a randomized trial of 60 patients with a subset of ICM (chronic total occlusion of infarct-related area). Patients will be randomized to ICD alone or ICD plus catheter ablation, with an endpoint of time to first ICD therapy or VT-related hospitalization. The VANISH2 trial (NCT02830360) is enrolling 366 patients with prior MI who present with sustained monomorphic VT and is randomizing them to receive either catheter ablation or AAD therapy, with a primary composite endpoint of death, appropriate shock, or VT storm.

#### Endpoints for prospective clinical trials of ventricular tachycardia ablation

Although observational studies have demonstrated an association between recurrent VAs and mortality [311, 340, 634, 1148], no prospective study thus far has demonstrated a significant beneficial effect of VT ablation on survival. Successful completion of a prospective trial of VT ablation with a primary outcome of mortality is challenging due to competing mortality risks in patients with VT. In the VANISH trial, there was no clear effect of ablation on mortality in comparison with escalated AAD therapy [307, 1149], likely because arrhythmia was an infrequent cause of death in the trial, and because additional interventions were undertaken when patients experienced clinical deterioration or recurrent arrhythmia. For randomized trials enrolling patients with low-frequency VT events, the use of time-to-event endpoints (rather than measures of arrhythmia burden) has traditionally been preferred, given clinicians and patients might not tolerate large burdens of recurrent arrhythmia events without considering treatment alternatives to assigned therapy. For randomized trials specifically enrolling patients with high VT burden, the use of reduction in VT burden provides a meaningful endpoint for both patient satisfaction and scientific inquiry.

#### Future clinical studies

Further research is required to identify optimal methods for arrhythmia suppression and to understand the influences of each method on arrhythmia outcomes, as well as on cardiac function, symptoms, quality of life [1135], cost-effectiveness [1150, 1151], and long-term outcomes. The best first-line therapy for VT, beyond the use of ICDs, remains undetermined, as does the most appropriate time to intervene. Ablation outcomes in nonischemic cardiomyopathies, specific disease states, and patient subgroups also require further study. Developing, funding, and executing patient-centered, prospective, and randomized trials are strongly encouraged to move the field of VT ablation forward in ways that promote patient health and minimize confounding biases.

### Future directions in the treatment of patients with ventricular arrhythmias

#### Introduction

Awareness of the importance of VAs as a frequent and potentially modifiable cause of death has led to increased development of novel methods to improve VT mapping (both inside and outside the body), VT treatment (inside and outside the body), and identification of an individual patient’s status, along the spectrum of cardiac illness.

#### Advances in mapping

Cardiac mapping has made an important advance with the development of tools that employ multiple small, closely spaced electrodes. These tools provide higher resolution mapping during sustained VT and sinus rhythm (see Section 8.1).

Outside of catheter mapping, tools have been created to take advantage of coronary vasculature access to intramyocardial structures. The most common example involves mapping the perforator veins [278] facilitated by the use of electrically protected wires into septal perforating branches [825], the use of cold saline [820], and the use of dual site pace mapping [822] to identify intramural VA origins.

Advances in cardiac imaging are enabling a more comprehensive understanding of the 3D nature of cardiac scarring and the dynamic nature of ventricular electrophysiology. Preprocedural imaging with CMR and CT is increasingly being used to identify abnormal myocardium, but its value as a stand-alone ablation guide has not yet been proven. Studies are underway to address this possibility.

The synthesis of cardiac imaging with detailed cardiac mapping is furthering the understanding of the relationship between the VT circuit and myocardial scarring. Important observations have been made from mapping several surfaces of the heart simultaneously [1152]. Additional observations can be made by comparing maps and electrograms in sinus rhythm, RV pacing, LV pacing and sustained VT [119]. However, these detailed maps are time-consuming to create, potentially exposing patients to additional procedural risk. The translation of these observations into tangible value for he operator or for improved patient outcomes remains to be proven and is an area of active investigation.

Entirely noninvasive electrophysiological mapping has been developed with ECGI. This technology combines body surface unipolar electrograms obtained from a vest of electrodes with patient-specific heart-torso geometry. Local ECGs are reconstructed on the surface of the heart geometry, requiring a single beat of arrhythmia. ECGI has been shown to localize the site of VT origin [477, 1153], ventricular scar locations [1154], and risk assessment for future VT events in a small cohort of patients with IHD and ICDs [476]. Compared with catheter-based mapping, noninvasive ECGI mapping of VT does not provide similar local bipolar electrogram detail and ECGI does not image during ventricular diastole. Unipolar ECG reconstructions on the ventricular surface can infer activation patterns from deeper structures, such as septum or papillary muscles, but further refinement is needed to reliably map these structures. Advantages include the ability to map both ventricles simultaneously in a single beat, obtaining an immediate sense of VT entrance and exit locations, as well as mapping unstable VT/VF. Outside of the electrophysiology lab, ECGI offers possibilities for clinical VT risk stratification, such as was recently demonstrated with Brugada syndrome [1155], long QT syndrome [1156], and early repolarization syndrome [1157].

#### Advances in ablation

A better understanding of biophysical properties of RF energy to create ablative heat in both normal and abnormal ventricular myocardium has driven important innovations. A change in the catheter irrigant to half normal saline alters the local ionic content, which creates a larger ablation lesion [760]. In prospective clinical use, this approach has translated into high rates of successful ablations in patients who failed traditional ablation [761], although direct comparisons with conventional irrigant are lacking. Caution should be exercised, given the authors reported a 12% rate of steam pop with this technique.

Alternate forms of delivering RF energy have been developed. The most prominent of such technologies is a catheter with a needle that can be deployed into ventricular myocardium to both map and ablate [77]. Clinical trials in patients with VA are underway (NCT01791543; NCT03204981). Advantages include more precise intramyocardial mapping, injection of dye to assess location of potential ablation using fluoroscopy, and deeper ablation lesions than standard catheter ablation. Disadvantages include the difficulty ensuring safe use and unpredictable energy distribution. A warm saline-infused needle ablation catheter, capable of creating large lesions, is currently being studied in patients with ICM and VT (NCT02994446) [770]. Alternative methods for RF delivery to larger areas are under development, including expandable catheter tips that form spheres or round lattice structures.

Beyond RF energy to create thermal ablation, alternative energies have been studied to control VT. These alternatives include localized intracoronary alcohol injection, locally applied pulsed field direct current electroporation, and noninvasive focused stereotactic radiation. Coronary ethanol infusion has been performed in both an antegrade [779] and retrograde fashion [781] on patients for the treatment of refractory VT. Electroporation using localized direct current is a technology in rapid development with significant advantages of speed (seconds) and a tissue-selective ablation effect in preclinical models. Its safety and efficacy in humans has yet to be established. Stereotactic radioablation is a noninvasive and rapid (minutes) method for delivery of photons (X-rays), protons, or heavy ions (carbon) into selected cardiac tissue. The use of noninvasive photon ablation in humans was first reported in 2015 [786]. The development of an entirely noninvasive process for mapping and ablating VT, combining ECGI, cardiac imaging, and stereotactic cardiac ablation, represents a promising new option in the field [785].

Outside of a direct ablative effect on the diseased myocardium, modulation of the nervous system can alter the likelihood that VT will occur. Neuromodulation is largely designed to decrease sympathetic tone and enhance parasympathetic tone. There are many locations at which to manipulate the nervous system, including cervical vagal stimulation, transcutaneous auricular vagal stimulation, baroreceptor activation therapy, spinal cord stimulation, ganglionated plexus ablation, renal sympathetic denervation, and left CSD.

#### Advances in patient evaluation

As we progress into a future where artificial intelligence becomes increasingly used for predictive analytics, the field of VT ablation is ripe for such advances. In silico multiscale cardiac modeling of patients with ICM has provided an exciting possibility for the development of a patient-specific a priori ablation strategy [1158]. Beyond this approach, the use of predictive tools can further help physicians to identify the right patients, the proper timing of the procedure, and the expected outcomes with various forms of ablation techniques to ultimately offer the right patient the right ablation at the right cardiac location at the right time in the course of the disease.

### Electronic supplementary material

ESM 1 (PDF 2569 kb)

## Appendix 1

**Table 11:** Author disclosure table

Writing group member	Employment	Honoraria/Speaking/Consulting	Speakers’ bureau	Research∗	Fellowship support∗	Ownership/Partnership/Principal/Majority stockholder	Stock or stock options	Intellectual property/Royalties	Other
Edmond M. Cronin, MB, BCh, BAO, FHRS, CCDS, CEPS-A (Chair)	Hartford Hospital, Hartford, Connecticut	None	None	None	None	None	None	None	None
Frank M. Bogun, MD (Vice-Chair)	University of Michigan, Ann Arbor, Michigan	None	None	None	None	None	None	None	None
Philippe Maury, MD (EHRA Chair)	University Hospital Rangueil, Toulouse, France	None	None	None	None	None	None	None	None
Petr Peichl, MD, PhD (EHRA Vice-Chair)	Institute for Clinical and Experimental Medicine, Prague, Czech Republic	1: Abbott; 1: Biosense Webster; 1: BIOTRONIK	None	None	None	None	None	None	None
Minglong Chen, MD, PhD, FHRS (APHRS Chair)	Jiangsu Province Hospital, The First Affiliated Hospital of Nanjing Medical University, Nanjing, China	None	None	None	None	None	None	None	0: APHRS Board Member
Narayanan Namboodiri, MBBS, MD (APHRS Vice-Chair)	Sree Chitra Institute for Medical Sciences and Technology, Thiruvananthapuram, India	None	None	None	None	None	None	None	None
Luis Aguinaga, MD, PhD, FESC, FACC (LAHRS Chair)	Centro Privado de Cardiología, Tucuman, Argentina	None	None	None	None	None	None	None	None
Luiz Roberto Leite, MD, PhD, FHRS (LAHRS Vice-Chair)	Instituto Brasília de Arritmia, Brasília, Brazil	None	1: Boehringer Ingelheim	None	None	None	None	None	None
Sana M. Al-Khatib, MD, MHS, FHRS, CCDS	Duke University Medical Center, Durham, North Carolina	None	None	None	None	None	None	None	None
Elad Anter, MD	Beth Israel Deaconess Medical Center, Boston, Massachusetts	2: Itamar Medical; 3: Boston Scientific; 4: Biosense Webster	None	None	None	None	None	None	None
Antonio Berruezo, MD, PhD	Heart Institute, Teknon Medical Center, Barcelona, Spain	1: Biosense Webster	1: Biosense Webster	2: Biosense Webster	None	None	None	None	None
David J. Callans, MD, FHRS, CCDS	University of Pennsylvania, Philadelphia, Pennsylvania	1: Abbott Laboratories; 1: BIOTRONIK; 1: Medtronic; 1: Wolters Kluwer; 2: Boston Scientific	None	4: NIH	2: BIOTRONIK; 2: Boston Scientific; 4: Abbott; 4: Biosense Webster; 4: Medtronic	None	None	None	1: Acutus Medical; 1: AtriCure; 1: Impulse Dynamics; 1: Thermedical; 3: Bayer
Mina K. Chung, MD, FHRS	Cleveland Clinic, Cleveland, Ohio	2: ABIM	None	5: AHA; 5: NIH	None	None	None	1: Elsevier; 1: Up to Date	0: ACC (EP Section Leadership Council member); 0: AHA (Chair, ECG & Arrhythmias Committee; Member, Clinical Cardiology Leadership Committee; Member, Committee on Scientific Sessions Programming); 0: Amarin (Data monitoring committee member); 0: BIOTRONIK; 2: AHA (Associate Editor, *Circulation Arrhythmia & Electrophysiology*)
Phillip Cuculich, MD	Washington University School of Medicine, St. Louis, Missouri	2: Medtronic	None	None	None	None	None	None	None
Andre d’Avila, MD, PhD	Hospital Cardiologico SOS Cardio, Florianopolis, Brazil	None	None	None	None	None	None	None	None
Barbara J. Deal, MD, FACC	Northwestern University Feinberg School of Medicine, Chicago, Illinois	None	None	None	None	None	None	None	None
Paolo Della Bella, MD	Ospedale San Raffaele, Milan, Italy	2: Biosense Webster; 2: Boston Scientific; 3: Abbott	None	None	2: Medtronic; 3: Abbott Vascular; 3: Biosense Webster; 3: BIOTRONIK; 4: Boston Scientific	None	None	None	None
Thomas Deneke, MD, PhD, FHRS	Herz- und Gefäß-Klinik, Bad Neustadt, Germany	None	None	None	None	None	None	None	None
Timm-Michael Dickfeld, MD, PhD, FACC, FHRS	University of Maryland, Baltimore, Maryland	1: Abbott Laboratories; 1: Biosense Webster; 1: Impulse Dynamics; 1: Philips	None	1: GE Healthcare	None	None	None	None	None
Claudio Hadid, MD	Hospital General de Agudos Cosme Argerich, Buenos Aires, Argentina	None	None	None	None	None	None	None	None
Haris M. Haqqani, MBBS, PhD, FHRS	University of Queensland, The Prince Charles Hospital, Chermside, Australia	0: Abbott Laboratories; 0: Boston Scientific; 0: Medtronic	None	3: Biosense Webster	None	None	None	None	None
G. Neal Kay, MD, CCDS	University of Alabama at Birmingham, Birmingham, Alabama	None	None	None	None	None	None	None	None
Rakesh Latchamsetty, MD, FHRS	University of Michigan, Ann Arbor, Michigan	None	1: BIOTRONIK	None	None	None	None	None	None
Francis Marchlinski, MD, FHRS	University of Pennsylvania, Philadelphia, Pennsylvania	1: Abbott; 1: Biosense Webster; 1: Boston Scientific; 1: Medtronic; 2: BIOTRONIK	None	1: Abbott; 4: Biosense Webster	2: BIOTRONIK; 2: Boston Scientific; 4: Abbott; 4: Biosense Webster; 4: Medtronic	None	None	None	None
John M. Miller, MD, FHRS	Indiana University School of Medicine, Krannert Institute of Cardiology, Indianapolis, Indiana	1: Abbott Laboratories; 1: Biosense Webster; 1: Boston Scientific; 1: BIOTRONIK; 1: Medtronic	None	None	3: Biosense Webster; 3: Boston Scientific; 3: Medtronic	None	None	1: Elsevier	None
Akihiko Nogami, MD, PhD	University of Tsukuba, Ibaraki, Japan	1: Abbott Laboratories	None	4: Medtronic	None	None	None	None	None
Akash R. Patel, MD, FHRS, CEPS-P	University of California San Francisco Benioff Children’s Hospital, San Francisco, California	None	None	None	None	None	None	None	None
Rajeev Kumar Pathak, MBBS, PhD, FHRS	Australian National University, Canberra Hospital, Canberra, Australia	1: BIOTRONIK	1: Medtronic	None	None	None	None	None	None
Luis C. Saenz Morales, MD	CardioInfantil Foundation, Cardiac Institute, Bogota, Columbia	None	None	None	None	None	None	None	None
Pasquale Santangeli, MD, PhD	University of Pennsylvania, Philadelphia, Pennsylvania	1: Abiome; 1: Baylis Medical; 1: Biosense Webster; 1: Medtronic; 1: Stereotaxis; 2: Abbott Laboratories	None	None	None	None	None	None	None
John L. Sapp, Jr., MD, FHRS	Queen Elizabeth II Health Sciences Centre, Halifax, Canada	1: Abbott Vascular; 1: Biosense Webster; 1: Medtronic	None	4: Abbott Vascular; 5: Biosense Webster	None	None	None	1: Biosense Webster	None
Andrea Sarkozy, MD, PhD, FEHRA	University Hospital Antwerp, University of Antwerp, Antwerp, Belgium	1: Biosense Webster; 1: BIOTRONIK	None	None	None	None	None	None	0: EHRA Board member
Kyoko Soejima, MD	Kyorin University School of Medicine, Tokyo, Japan	2: Abbott Laboratories; 2: Boehringer Ingelheim	3: Medtronic	None	None	None	None	None	None
William G. Stevenson, MD, FHRS	Vanderbilt University Heart and Vascular Center, Nashville, Tennessee	1: Abbott; 1: BIOTRONIK; 1: Boston Scientific; 1: Medtronic	None	None	None	None	None	0: Biosense Webster; 0: Brigham and Women’s Hospital	None
Usha B. Tedrow, MD, MS, FHRS	Brigham and Women’s Hospital, Boston, Massachusetts	1: Abbott; 1: Biosense Webster; 1: Medtronic	None	None	None	None	None	None	None
Wendy S. Tzou, MD, FHRS	University of Colorado Denver, Aurora, Colorado	1: Abbott; 1: Biosense Webster; 1: BIOTRONIK; 1: Boston Scientific; 1: Medtronic	None	None	None	None	None	None	None
Niraj Varma, MD, PhD	Cleveland Clinic, Cleveland, Ohio	1: BIOTRONIK; 1: Medtronic	None	3: Abbott	None	None	None	None	None
Katja Zeppenfeld, MD, PhD, FESC, FEHRA	Leiden University Medical Center, Leiden, the Netherlands	1: Abbott	None	5: Biosense Webster	3: Biosense Webster	None	None	None	None

Number value: **0** = $0; **1** = ≤ $10,000; **2** = > $10,000 to ≤ $25,000; **3** = > $25,000 to ≤ $50,000; **4** = > $50,000 to ≤ $100,000; **5** = > $100,000

*ABIM* American Board of Internal Medicine, *ACC* American College of Cardiology, *AHA* American Heart Association, *APHRS* Asia Pacific Heart Rhythm Society, *EHRA* European Heart Rhythm Association, *LAHRS* Latin American Heart Rhythm Society, *NIH* National Institutes of Health

∗ Research and fellowship support are classed as programmatic support. Sources of programmatic support are disclosed but are not regarded as a relevant relationship with industry for writing group members or reviewers

## Appendix 2

**Table 12:** Reviewer disclosure table

Peer reviewer	Employment	Honoraria/Speaking/Consulting	Speakers’ bureau	Research∗	Fellowship support∗	Ownership/Partnership/Principal/Majority stockholder	Stock or stock options	Intellectual property/Royalties	Other
Samuel J. Asirvatham, MD, FHRS	Mayo Clinic College of Medicine, Rochester, Minnesota	1: Abbott; 1: BIOTRONIK; 1: Boston Scientific; 1: Medtronic	None	None	None	None	None	1: AliveCor	None
Eduardo Back Sternick, MD, PhD	Faculdade Ciências Médicas de Minas, Gerais, Brazil	None	None	None	None	None	None	None	None
Janice Chyou, MD	Northport VA Medical Center, Northport, New York; Agile Health, New York, New York	None	None	None	None	None	None	None	None
Sabine Ernst, MD, PhD	Royal Brompton and Harefield Hospitals, London, England	2: Biosense Webster; 2: Stereotaxis	None	3: Catheter Precision; 4: Baylis; 4: Spectrum Dynamics	None	None	None	None	None
Guilherme Fenelon, MD, PhD	Hospital Israelita Albert Einstein, Sao Paulo, Sao Paulo, Brazil	1: Libbs	None	None	None	None	None	None	None
Edward P. Gerstenfeld, MD, MS, FACC	University of California, San Francisco, San Francisco, California	1: Abbott Vascular; 1: Biosense Webster; 1: Boston Scientific; 1: Medtronic	None	4: Abbott Vascular; 4: Biosense Webster	None	None	None	None	None
Gerhard Hindricks, MD	Heart Center Leipzig at the University of Leipzig, Leipzig, Germany	None	None	4: Abbott Vascular	None	None	None	None	None
Koichi Inoue, MD, PhD	Sakurabashi-Watanabe Hospital, Osaka, Japan	2: Biosense Webster; 2: Medtronic Japan	None	None	None	None	None	None	None
Jeffrey J. Kim, MD	Baylor College of Medicine, Texas Children’s Hospital, Houston, Texas	None	None	None	3: Medtronic	None	None	None	None
Kousik Krishnan, MD, FHRS, FACC	Rush University Medical Center, Chicago, Illinois	1: ZOLL	None	None	None	None	None	None	None
Karl-Heinz Kuck, MD, FHRS	Asklepios Klinik St. Georg, Hamburg, Germany	1: Abbott Vascular; 1: Biosense Webster; 1: Boston Scientific; 1: Edwards Lifesciences; 1: Medtronic	None	None	None	None	None	None	None
Martin Ortiz Avalos, MD	Hospital San Angel Inn Universidad, Mexico City, Mexico	None	None	None	None	None	None	None	None
Thomas Paul, MD, FACC, FHRS	Georg August University Medical Center, Gottingen, Germany	None	None	None	None	None	None	None	None
Mauricio I. Scanavacca, MD, PhD	Instituto Do Coracao, Sao Paulo, Brazil	None	None	None	None	None	None	None	None
Roderick Tung, MD, FHRS	The University of Chicago Medicine, Center for Arrhythmia Care, Heart and Vascular Center, Chicago, Illinois	2: Abbott	None	2: Abbott	3: Abbott; 3: Medtronic; 3: Boston Scientific	None	None	None	None
Jamie Voss, MBChB	Middlemore Hospital, Auckland, New Zealand	None	None	None	None	None	None	None	None
Takumi Yamada, MD	University of Alabama at Birmingham, Birmingham, Alabama	1: Nihon Kohden; 2: Abbott; 2: Japan Lifeline	None	None	None	None	None	None	None
Teiichi Yamane, MD, PhD, FHRS	Jikei University School of Medicine, Tokyo, Japan	1: Boehringer Ingelheim; 1: Bristol-Myers Squibb; 2: Abbott Laboratories; 2: Medtronic Japan	None	None	None	None	None	None	None

Number value: 0 = $0; 1 = ≤ $10,000; 2 = > $10,000 to ≤ $25,000; 3 = > $25,000 to ≤ $50,000; 4 = > $50,000 to ≤ $100,000; 5 = > $100,000

∗ Research and fellowship support are classed as programmatic support. Sources of programmatic support are disclosed but are not regarded as a relevant relationship with industry for writing group members or reviewers

## Abbreviations

AAD: antiarrhythmic drug
ACT: activated clotting time
AF: atrial fibrillation
AHD: acute hemodynamic decompensation
AI: anatomical isthmus
AIV: anterior interventricular vein
AMC: aortomitral continuity
ANS: autonomic nervous system
ARVC: arrhythmogenic right ventricular cardiomyopathy
ATP: antitachycardia pacing
AV: atrioventricular
BBR: bundle branch reentry
BBRVT: bundle branch reentrant ventricular tachycardia
cAMP: cyclic adenosine monophosphate
CCM: Chagas cardiomyopathy
CHD: congenital heart disease
ChD: Chagas disease
CIED: cardiovascular implantable electronic device
CL: cycle length
CMR: cardiac magnetic resonance imaging
COR: class of recommendation
CRT: cardiac resynchronization therapy
CS: coronary sinus
CSD: cardiac sympathetic denervation
CT: computed tomography
DCM: dilated cardiomyopathy
D-TGA: d-transposition of the great arteries
EAM: electroanatomical mapping
ECG: electrocardiogram
ECGI: electrocardiographic imaging
EF: ejection fraction
GCV: great cardiac vein
HCM: hypertrophic cardiomyopathy
HR: hazard ratio
HS: hemodynamic support
IABP: intra-aortic balloon pump
ICD: implantable cardioverter defibrillator
ICE: intracardiac echocardiography
ICM: ischemic cardiomyopathy
IHD: ischemic heart disease
LAF: left anterior fascicle
LAVA: local abnormal ventricular activity
LBB: left bundle branch
LBBB: left bundle branch block
LGE: late gadolinium enhancement
LMNA: lamin A/C
LOE: level of evidence
LP: late potential
LPF: left posterior fascicle
LSV: left sinus of Valsalva
LV: left ventricle
LVAD: left ventricular assist device
LVEF: left ventricular ejection fraction
LVNC: left ventricular noncompaction
LVOT: left ventricular outflow tract
MDCT: multidetector cardiac computed tomography
MI: myocardial infarction
MRI: magnetic resonance imaging
NCSV: noncoronary sinus of Valsalva
NICM: nonischemic cardiomyopathy
NIPS: noninvasive programmed stimulation
NYHA: New York Heart Association
OR: odds ratio
OT: outflow tract
PES: programmed electrical stimulation
PET: positron emission tomography
pLVAD: percutaneous left ventricular assist device
PPI: postpacing interval
PVC: premature ventricular complex
RBB: right bundle branch
RBBB: right bundle branch block
RCT: randomized controlled trial
RF: radiofrequency
RSV: right sinus of Valsalva
RV: right ventricle
RVOT: right ventricular outflow tract
RWI: relationship with industry and other entities
SD: standard deviation
SHD: structural heart disease
SHFM: Seattle Heart Failure Model
SOO: site of origin
SPECT: single-photon emission computerized tomography
SV: sinus of Valsalva
SVT: supraventricular tachycardia
3D: three-dimensional
VA: ventricular arrhythmia
VAD: ventricular assist device
VF: ventricular fibrillation
VSD: ventricular septal defect
VT: ventricular tachycardia
